# *O*-Aminoalkyl-*O*-Trimethyl-2,3-Dehydrosilybins: Synthesis and In Vitro Effects Towards Prostate Cancer Cells

**DOI:** 10.3390/molecules23123142

**Published:** 2018-11-29

**Authors:** Bao Vue, Sheng Zhang, Andre Vignau, Guanglin Chen, Xiaojie Zhang, William Diaz, Qiang Zhang, Shilong Zheng, Guangdi Wang, Qiao-Hong Chen

**Affiliations:** 1 Department of Chemistry, California State University, Fresno, 2555 E. San Ramon Avenues, M/S SB70, Fresno, CA 93740, USA; baov@csufresno.edu (B.V.); shengzhang2014@mail.fresnostate.edu (S.Z.); andre1357@mail.fresnostate.edu (A.V.); chen.bmc@gmail.com (G.C.); zhangxiaojie87@gmail.com (X.Z.); diazwill101@mail.fresnostate.edu (W.D.); 2 Department of Chemistry and RCMI Cancer Research Center, Xavier University of Louisiana, 1 Drexel Drive, New Orleans, LA 70125, USA; qzhang1@xula.edu (Q.Z.); szheng@xula.edu (S.Z.); gwang@xula.edu (G.W.)

**Keywords:** silybin, prostate cancer, 2,3-dehydrosilybin, cell proliferation, cell apoptosis

## Abstract

As part of our ongoing silybin project, this study aims to introduce a basic nitrogen-containing group to 7-OH of 3,5,20-*O*-trimethyl-2,3-dehydrosilybin or 3-OH of 5,7,20-*O*-trimethyl-2,3-dehydrosilybin via an appropriate linker for in vitro evaluation as potential anti-prostate cancer agents. The synthetic approaches to 7-*O*-substituted-3,5,20-*O*-trimethyl-2,3-dehydrosilybins through a five-step procedure and to 3-*O*-substituted-5,7,20-*O*-trimethyl-2,3- dehydrosilybins via a four-step transformation have been developed. Thirty-two nitrogen-containing derivatives of silybin have been achieved through these synthetic methods for the evaluation of their antiproliferative activities towards both androgen-sensitive (LNCaP) and androgen-insensitive prostate cancer cell lines (PC-3 and DU145) using the WST-1 cell proliferation assay. These derivatives exhibited greater in vitro antiproliferative potency than silibinin. Among them, **11**, **29**, **31**, **37**, and **40** were identified as five optimal derivatives with IC_50_ values in the range of 1.40–3.06 µM, representing a 17- to 52-fold improvement in potency compared to silibinin. All these five optimal derivatives can arrest the PC-3 cell cycle in the G_0_/G_1_ phase and promote PC-3 cell apoptosis. Derivatives **11**, **37**, and **40** are more effective than **29** and **31** in activating PC-3 cell apoptosis.

## 1. Introduction

**Figure 1:**
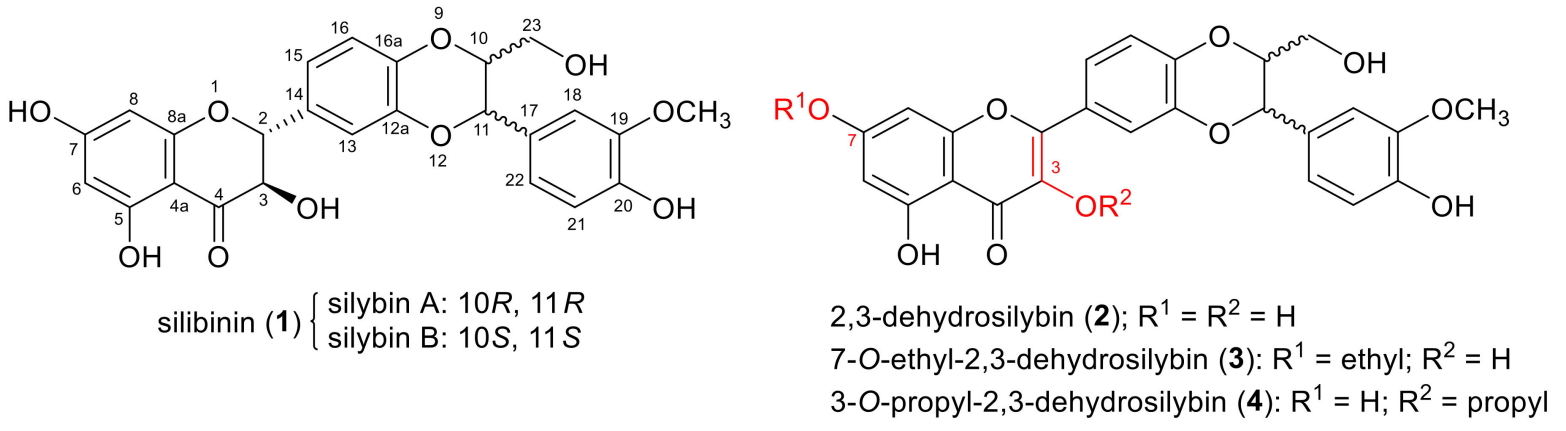
Structures of silibinin and derivatives.

Silybin ((**1**), Figure 1), also known as silibinin, exists in nature as an approximately equimolar mixture of two diastereomers of silybin A and silybin B with opposite configurations at C-10 and C-11 [1]. They are hard to separate by conventional purification methods, but can be separated by the HPLC method [2]. Silybin A and silybin B biogenetically originate from a taxifolin moiety (flavonoid) and a coniferyl alcohol unit (lignan), which was presumed to follow a non-stereoselective radical coupling reaction [3]. The mixture of diastereoisomeric silybin A and silybin B was originally thought to be a pure compound and named silybin [4]. Later, it was re-termed as silibinin to emphasize that it is a mixture [5]. In this article, silibinin was thus used to represent the mixture of silybin A and silybin B. Silibinin (**1**) is the first and well-studied member of flavonolignans [4] and the key chemical and medicinal component of milk thistle (*Silybum marianum* L. Gaertner, Asteraceae) [6]. The earliest record on the medicinal merit of Milk thistle for preventing and treating various hepatotoxicity is Hieronymus Bock’s book published in 1539 [7,8]. Milk thistle and silibinin are now attractive to scientists, not only for their well-known chemotherapeutic use for hepatotoxicity in Europe [9], but also for their potential in treating and preventing cancers [5,10,11,12]. Silymarin (crude extract of milk thistle), silibinin (diastereomeric mixture), silybin A (optically pure), and silybin B (optically pure) have been demonstrated by various in vitro cell-based and in vivo animal-based assays to possess therapeutic potential in treating prostate cancer [12,13,14]. The detailed mechanism of silibinin and structurally-related flavonolignans as anti-prostate cancer agents has previously been summarized by us in a review article [15]. Briefly, this group of natural products can arrest the prostate cancer cell cycle at G1 phase and induce cancer cell apoptosis. Also, they have been demonstrated to inhibit the secretion of prostate cancer specific antigen (PSA) and androgen receptor (AR). They have been revealed to possess anti-angiogenesis properties by decreasing the density of prostate tumor microvessels and VEGF immunoreactivity. Additionally, silibinin and structurally-related compounds can suppress prostate cancer cell migration and invasion through down-regulating the vimentin protein and MMP-2 mRNA, up-regulating E-cadherin expression, and reversing the epithelial-to-mesenchymal transition (EMT). Additionally, the non-toxic profiles of silibinin have been confirmed by its long-term use as a dietary supplement and a Phase I clinical trial of silybin-phytosome, a formula of silibinin, at a dose of 13 g/day [16]. However, the development of silibinin as an anti-prostate cancer drug is hindered, at least partly, by its moderate potency, with its IC_50_ values of 40–106 µM in prostate cancer cell models [12,13,15,17] and by its poor pharmacokinetic profiles [16]. Structural modification of silibinin can serve as a viable strategy to enhance its potency. Methylated silybins have been reported to be capable of increasing antiproliferative activities towards prostate cancer cells [18]. Additionally, 2,3-dehydrosilybin has been shown to be a significantly better anticancer agent than silibinin [19]. Structural manipulations on the phenolic and alcoholic hydroxyl groups of silibinin and 2,3-dehydrosilybin have been applied to overcome their pharmacokinetic limitations [20,21,22].

Our previous studies on the structure-activity relationships of silibinin revealed that the antiproliferative potency of 2,3-dehydrosilybin in three prostate cancer cell models could be further improved through introducing a suitable alkyl group on 7-OH and 3-OH, as exemplified by 7-*O*-ethyl-2,3-dehydrosilybin (**3**) and 3-*O*-propyl-2,3-dehydrosilybin (**4**) (Figure 1) [23,24]. This encouraged us to further investigate the effects of nitrogen-containing groups on 7-OH and 3-OH of 2,3-dehydrosilybin on the biological profiles in prostate cancer cell models. Consequently, this study started with the development of general synthetic approaches to 7-*O*-substituted-3,5,20-*O*-trimethyl-2,3-dehydrosilybins and 3-*O*-substituted-5,7,20-*O*-trimethyl-2,3- dehydrosilybins, followed by the synthesis of thirty-two new derivatives of silibinin, including six 7-*O*-aminoalkyl-3,5,20-*O*-trimethyl-2,3-dehydrosilybins and 26 3-*O*-aminoalkyl-5,7,20-*O*- trimethyl-2,3-dehydrosilybins. Additionally, the phenolic hydroxyl groups in all synthetic derivatives were converted to methoxyl groups. This conversion was expected to overcome, to some degree, pharmacokinetic limitations caused by the phenolic hydroxyl groups and to pave an avenue to the selective incorporation of a basic nitrogen-containing group into the phenolic hydroxyl group at either C-7 or C-3. The in vitro anticancer activities of these derivatives have been evaluated in three prostate cancer cell models. The design, synthesis, antiproliferative activity, and structure-activity relationships of these silibinin derivatives are presented in this paper. The cell apoptosis induction and cell cycle regulation by five representative derivatives are also reported.

## 2. Results and Discussion

### 2.1. Chemistry

#### 2.1.1. Synthesis of 7-*O*-Aminoalkyl-3,5,20-*O*-Trimethyl-2,3-Dehydrosilybins (**9**–**14**)

**Scheme 1:**
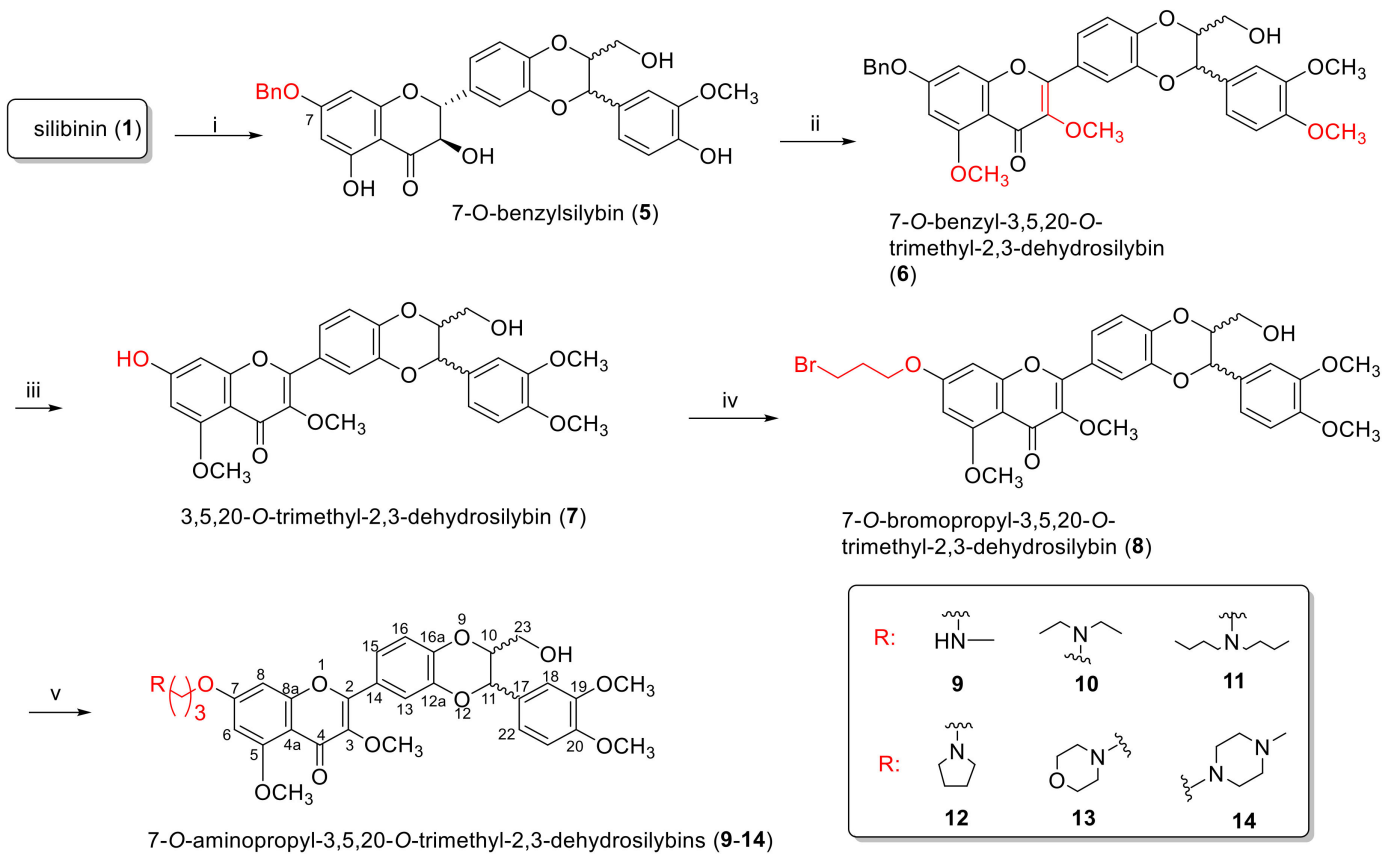
Synthesis of 7-*O*-aminopropyl-3,5,20-*O*-trimethyl-2,3-dehydrosilybins (**9**–**14**). Reagents and conditions: i. K_2_CO_3_ (4 equiv.), BnBr (1 equiv.), acetone (0.1 M), reflux overnight, 80%; ii. (a) K_2_CO_3_ (3 equiv.), DMF (0.5 M), rt, 3 h; (b) MeI (6 equiv.), rt, overnight, 48%; iii. HCOONH_4_ (10 equiv.), Pd/C (10%, *w*/*w*), MeOH (0.2 M), reflux, overnight, 67%; iv. 1,3-dibromopropane (4 equiv.), K_2_CO_3_ (4 equiv.), DMF (1 M), 60 °C, overnight; v. amine (3 equiv.), K_2_CO_3_ (3 equiv.), acetone (0.1 M), reflux, overnight, 40%.

Some oxidative conversions of silibinin to 2,3-dehydrosilybin have been reported [25,26,27]. As illustrated in Scheme 1, the synthesis of 7-*O*-substituted-2,3-dehydrosilybins (**9**–**14**) started with selective benzylation (81%) of the C-7 phenolic hydroxyl group of silibinin, according to the procedure reported by Kren et al. and us [23,28]. It is worth noting that anaerobic conditions are essential to achieving high yields for the selective benzylation. This is because the simultaneous presence of a base and air led to the aerobic oxidation of silibinin to 2,3-dehydrosilybin [8,24] and the 3-OH in 2,3-dehydrosilybin is readily benzylated or alkylated [8,24,28], which has been rationalized by the electrochemistry measurements and bond dissociation energy calculations [29]. 7-*O*-Benzyl-3,5,20-*O*-trimethyl-2,3-dehydrosilybin (**6**) was then achieved by the one-pot reaction of the base-mediated oxidation of 7-*O*-benzylsilybin (**5**), followed by trimethylation of the corresponding 7-*O*-benzyl-2,3-dehydrosilybin. 3,5,20-*O*-Trimethyl-2,3-dehydrosiliybin (**7**) was obtained by debenzylation of aryl benzyl ether **6** using ammonium formate as the hydrogen source, catalyzed by palladium on carbon. 7-*O*-bromopropyl-3,5,20-*O*-trimethyl-2,3-dehydrosilybin (**8**) was prepared by *O*-alkylating **7** with 1,3-dibromopropane mediated by potassium carbonate. 7-*O*-Aminoalkyl-3,5,20-*O*-trimethyl-2,3-dehydrosilybins (**9**–**14**) were achieved by *N*-alkylation of the bromoalkyl side chain of **8** with the appropriate amine.

#### 2.1.2. Synthesis of 3-*O*-Aminoalkyl-5,7,20-*O*-Trimethyl-2,3-Dehydrosilybins (**20**–**45**)

**Scheme 2:**
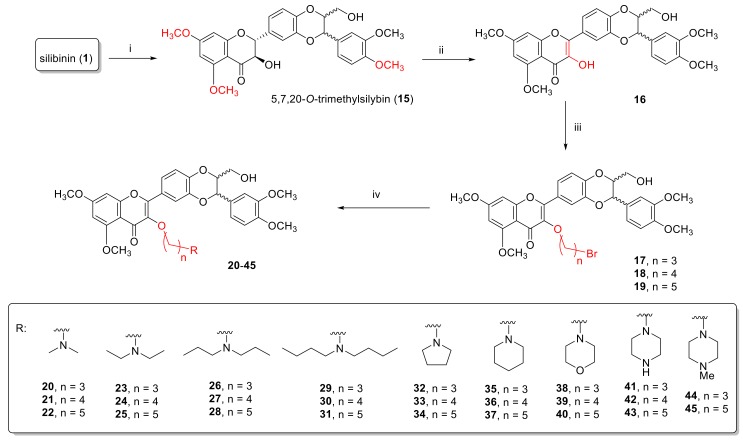
Synthesis of 3-*O*-aminoalkyl-5,7,20-*O*-trimethyl-2,3-dehydrosilybins (**20**–**45**). Reagents and conditions: i. dimethyl sulfate (8 equiv.), K_2_CO_3_ (8 equiv.), acetone, argon, reflux 4 h; ii. H_2_O_2_ (30%), NaOH (16%), methanol/THF, rt overnight; iii. Dibromoalkane (10 equiv.), K_2_CO_3_ (6 equiv.), DMF, rt 24–48 h; iv. Amine (16 equiv.), K_2_CO_3_ (6 equiv.), acetone, rt 48 h.

3-*O*-Substituted-2,3-dehydrosilybins (**20**–**45**) were synthesized following a four-step procedure, as shown in Scheme 2. Specifically, 5,7,20-*O*-trimethylsilybin (**15**) was achieved by treating silibinin (**1**) with dimethylsulfate in the presence of potassium carbonate under strictly anaerobic conditions. Note that a small amount of 3,5,7,20-*O*-tetramethyl-2,3-dehydrosilybin can be formed if anaerobic conditions are not well-controlled, which would complicate the purification process and decrease the yield. Even though 5,7,20-*O*-tribenzylsilybin was much easier to aerobically oxidize than that in silibinin [30], the oxidation of 5,7,20-*O*-trimethylsilybin (**15**) under the same conditions led to a mixture of products instead of the desired oxidation product. After several trials with different oxidation conditions, 5,7,20-*O*-trimethyl-2,3-dehydrosilybin (**16**) was eventually obtained by the oxidation of **15** with sodium hydroxide and hydrogen peroxide. A 10–14 h reaction time serves as a critical factor for the optimal yield (40–55%) of this oxidation reaction. We also found that this oxidation cannot be quenched with hydrochloric acid because it selectively demethylated the 5-OMe of the product. 5,7,20-*O*-Trimethyl-2,3-dehydrosilybin (**16**) was then converted to 3-*O*-bromoalkyl-5,7,20-*O*-trimethyl-2,3-dehydrosilybins (**17**–**19**) via *O*-alkylation with the appropriate dibromoalkanes, using potassium carbonate as the base and DMF as the aprotic solvent. The subsequent *N*-alkylation of the 3-*O*-bromoalkyl-5,7,20-*O*-trimethyl-2,3-dehydrosilybins (**17**–**19**) with the corresponding amine furnished the respective 3-*O*-aminoalkyl-5,7,20-*O*-trimethyl-2,3-dehydrosilybin (**20**–**45**).

#### 2.1.3. Structure Determination

The ^1^H-NMR and ^13^C-NMR data of **5** are consistent with those reported in the literature [28]. The structure of **6** was characterized by the presence of three single signals at 3.94, 3.924, and 3.916 in its ^1^H-NMR spectrum and at 60.1, 56.6, and 56.2 in its ^13^C-NMR spectrum for three additional methoxyl groups when compared with **5** and the absence of the ^1^H-NMR signal at 4.52 for the H-3 of **5**. The structure of **7** was established based on the absence of the ^1^H-NMR signals at 7.48–7.34 (m, 5H) and 5.12 (s, 2H) for the benzyl group of **6**. The signals in the ^1^H-NMR spectrum [4.17 (t, *J* = 5.7 Hz, 2H), 3.61 (t, *J* = 6.3 Hz, 2H), 2.36 (quin, *J* = 6.0 Hz, 2H)] of compound **8** confirmed the addition of a 3-bromopropyl group when compared with **7**. The structures of the six 7-*O*-aminoalkyl-3,5,20-*O*-trimethyl-2,3-dehydrosilybins (**9**–**14**) were characterized by interpreting their NMR, HRMS, and FTIR data (for details see Experimental Section and Supporting Information). Specifically, the presence of the ^1^H-NMR and ^13^C-NMR signals for each alkylamine group suggests the incorporation of the corresponding alkylamine group, which was corroborated by the HRMS data for each compound. ^1^H-NMR and ^13^C-NMR data for **9**–**14** were assigned (Experimental Section and Supporting Information) by comparing them with the NMR data of 7-*O*-propyl-2,3-dehydrosilybin because derivatives **9**–**14** and 7-*O*-propyl-2,3-dehydrosilybin share an identical core structure and all NMR signals of 7-*O*-propyl-2,3-dehydrosilybin have been fully assigned by us, according to the interpretation of its COSY, HMQC, and HMBC data [23].

The structure of **15** was determined by the existence of three single signals for three additional methoxyl groups at 3.91, 3.90, and 3.90 in its ^1^H-NMR spectrum when compared with silibinin. The structure of **16** was confirmed by the absence of the ^1^H-NMR signal at 4.42 (1H) for H-3 of **15**. The signals in the ^1^H-NMR spectra [4.14 (t, 2H), 3.60 (t, 2H), 2.31–2.26 (m, 2H) of **17**; 4.14 (t, 2H), 3.46 (t, 2H), 2.09–2.00 (m, 2H), 1.90–1.81 (m, 2H) of **18**; 4.02 (t, 2H), 3.34 (t, 2H), 1.85 (quin, 2H), 1.74 (quin, 2H), 1.59–1.49 (m, 2H) of **19**] confirmed the addition of an appropriate bromoalkyl group to each of **17**–**19** when compared with **16**. The structures of the 26 3-*O*-aminoalkyl-5,7,20-*O*-trimethyl-2,3-dehydrosilybins (**20**–**45**) were characterized by interpreting their NMR, HRMS, and FTIR data (for details, see Experimental Section and Supporting Information). Specifically, the presence of the ^1^H-NMR and ^13^C-NMR signals for each alkylamine group suggests the incorporation of the corresponding alkylamine group, which was further supported by the HRMS data for each compound. ^1^H-NMR and ^13^C-NMR data for **20**–**45** were assigned (Experimental Section and Supporting Information) by comparing them with the NMR data of 3-*O*-propyl-2,3-dehydrosilybin (**4**), due to the fact that derivatives **20**–**45** and compound **4** possess an identical core structure and all NMR signals of compound **4** have been fully assigned by us, according to the interpretation of its COSY, HMQC, and HMBC data [24].

### 2.2. Antiproliferative Activity towards Prostate Cancer Cell Lines and Structure-Activity Relationships

The in vitro antiproliferative activities of six 7-*O*-substituted and 26 3-*O*-substituted silybin derivatives were evaluated using the WST-1 cell proliferation assay, according to the procedure as described in the Experimental Section in both androgen-sensitive (LNCaP) and androgen-insensitive (PC-3 and DU145) human prostate cancer cell lines. Silibinin and docetaxel were used as a positive control for comparison in the parallel experiments and the IC_50_ values calculated from the dose-response curves are summarized in Table 1. Clearly, 7-*O*-aminoalkyl-3,5,20-*O*-trimethyl-2,3-dehydrosilybins and 3-*O*-aminoalkyl-5,7,20-*O*-trimethyl-2,3-dehydrosilybins are more potent in suppressing both androgen-sensitive and androgen-insensitive prostate cancer cell proliferation than silibinin. This conclusion is supported by the following data: i) the optimal 7-*O*-substituted derivative (**11**) is 52-, 51-, and 24-fold more potent than silibinin toward PC-3, DU145, and LNCaP prostate cancer cell lines, respectively; and ii) the optimal 3-*O*-substituted derivatives (**29**, **31**, **37**, and **40**) are 26–27, 31–37 and 17–22 times more potent than silibinin against PC-3, DU145, and LNCaP prostate cancer cell lines, respectively. Furthermore, the most potent derivatives **11**, **29**, **31**, **37**, and **40** are more active than 2,3-dehydrosilybin (**2**), 7-*O*-ethyl-2,3-dehydrosilybin (**3**), and 3-*O*-propyl-2,3-dehydrosilybin (**4**) in suppressing DU145 prostate cancer cell proliferation. Additionally, the dibutylamino group in derivatives **11**, **29** and **31**; the morpholino moiety in **37**; and the piperidino unit in **40,** are the favorable nitrogen-containing groups for the greater potency. A three-carbon linker in **11** and **29**, as well as a five-carbon linker in **31**, **37** and **40**, are beneficial to the potency.

### 2.3. Antiproliferative Activity towards MCF 10A and PWR-1E Non-Neoplastic Human Epithelial Cell Lines

Silibinin and five potent derivatives (**11**, **29**, **31**, **37**, and **40**) were selected for further evaluation against the MCF 10A non-neoplastic human mammary epithelial cell line and PWR-1E non-neoplastic human prostate epithelial cells. The five derivatives were chosen based on the following grounds: **11** is the most potent 7-*O*-aminoalkyl-3,5,20-*O*-trimethyl-2,3-dehydrosilybin considering its overall potency towards three prostate cancer cell models; **29**, **31**, **37**, and **40** are the most promising 3-*O*-aminoalkyl-5,7,20-O-trimethyl-2,3-dehydrosilybins. As shown in Table 2, silibinin demonstrates a significantly higher capability of suppressing non-neoplastic cell (MCF 10A and PWR-1E) proliferation than prostate cancer cell proliferation. Four 3-*O*-aminoalkyl-5,7,20-*O*-trimethyl-2,3-dehydrosilybins (**29**, **31**, **37**, and **40)** did not exhibit significantly different responses to prostate cancer cells and to non-neoplastic MCF 10A and PWR-1E cells. However, the 7-*O*-aminoalkyl-3,5,20-*O*-trimethyl-2,3-dehydrosilybin **11** does not demonstrate apparent antiproliferative activity towards PWR-1E and MCF 10A non-neoplastic epithelial cells up to a 50 µM concentration. Consequently, 7-*O*-aminoalkyl-3,5,20-*O*-trimethyl-2,3-dehydrosilybin **11** illustrates a good selectivity of inhibiting prostate cancer cell proliferation over non-neoplastic MCF 10A and PWR-1E human epithelial cell proliferation (Table 2), which suggests that modification at 7-OH of 3,5,20-*O*-trimethyl-2,3-dehydrosilybin only improves the antiproliferative potency towards prostate cancer cells, and not non-neoplastic epithelial cells.

### 2.4. Cell Cycle Regulation and Cell Apoptosis

Silibinin can arrest the rat (H-7 and I-8) and human prostate cancer cell (LNCaP) cycle at the G_1_ phase [31,32], and cause G_1_ and G_2_-M PC-3 prostate cancer cell cycle arrest [33]. Five optimal derivatives, consisting of **11**, **29**, **31**, **37**, and **40**, were selected for flow cytometry evaluation of their effect on PC-3 cell cycle regulation because they exhibited optimal cell proliferation inhibition on both androgen-dependent LNCaP and androgen-independent PC-3 prostate cancer cell models, with ≤3.0 µM IC_50_ values. At 20 µM, all these five derivatives can cause PC-3 cell accumulation in a G_0_/G_1_ phase by increasing the cell population in this phase at 16 h from 55.7% (control) to 66.3% (treated with **11**), from 36.2% (control) to 50.3% (treated with **29**), from 33.1% (control) to 35.9% (treated with **31**), from 33.1% (control) to 34.6% (treated with **37**), and from 33.1% (control) to 43.4% (treated with **40**).

**Figure 2:**
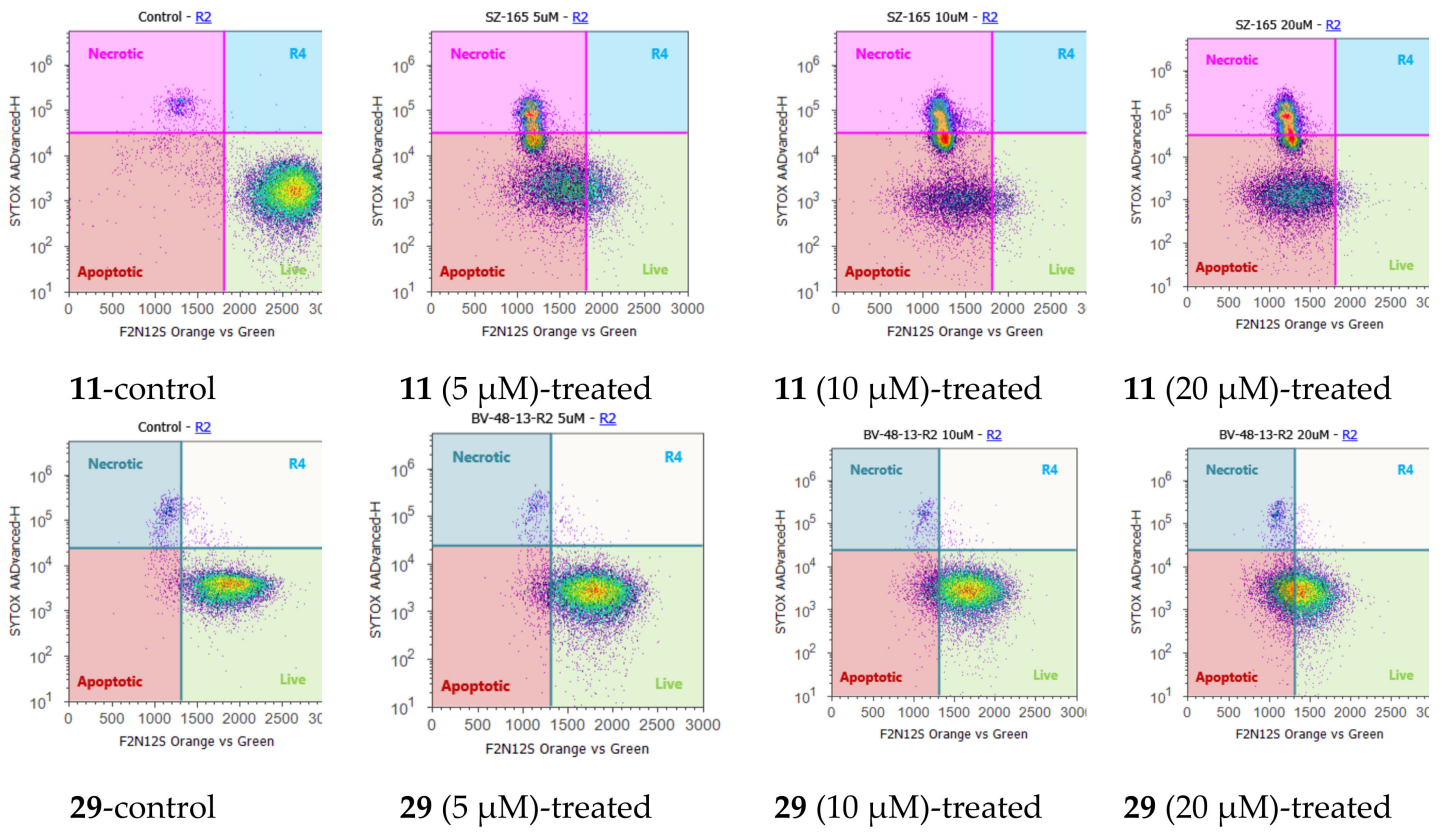

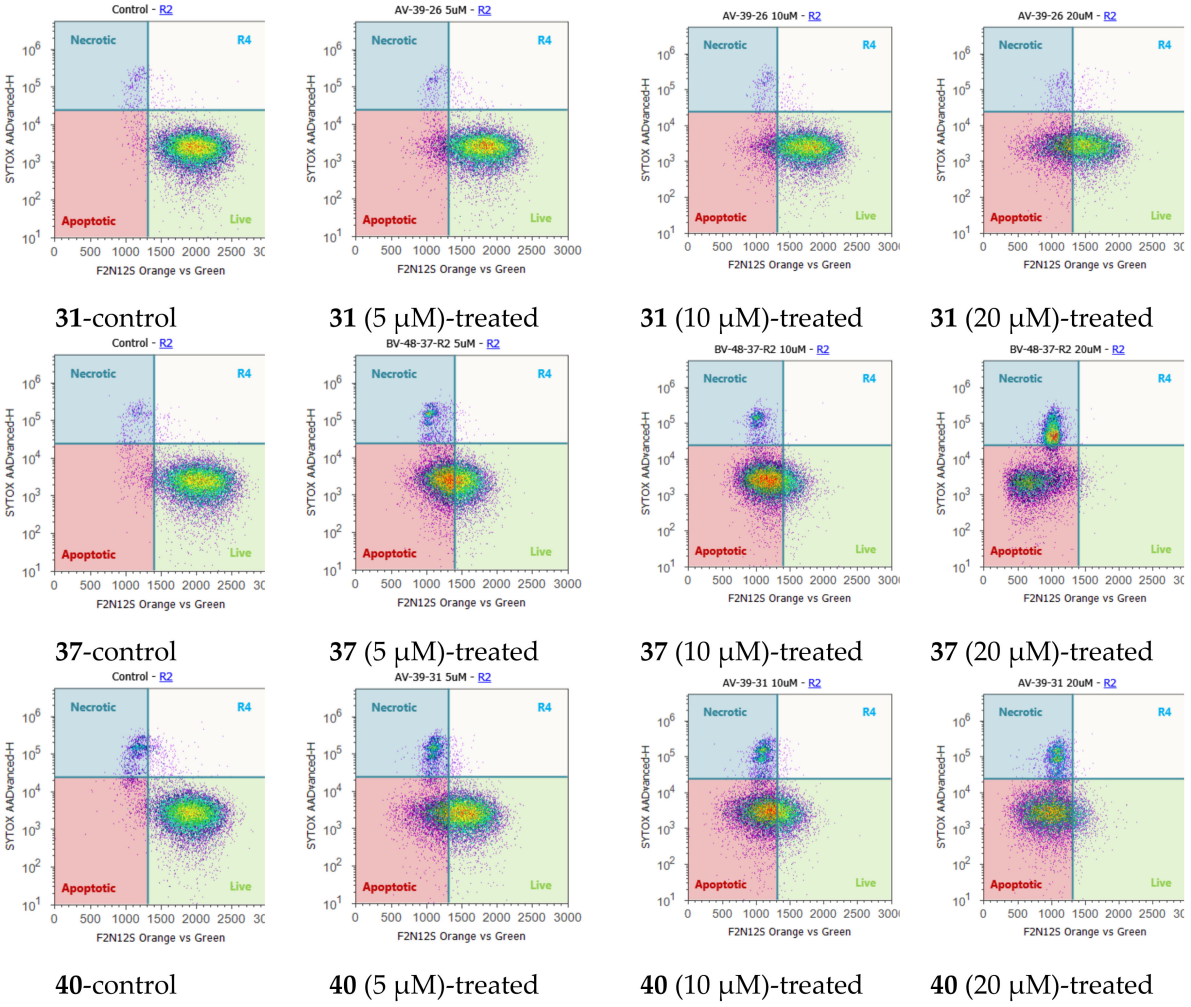
Apoptosis in PC-3 cells treated with **11**, **29**, **31**, **37**, and **40** at 5 µM, 10 µM, and 20 µM (by F2N12S and SYTOX AADvanced double staining).

Silibinin was revealed by Agarwal and co-workers to activate cell apoptosis in PC-3 tumor xenografts [34]. An F2N12S and SYTOX AADvanced double staining flow cytometry-based assay was used to discriminate PC-3 cells dying from apoptosis from those dying from necrosis in response to various concentrations of derivatives **11**, **29**, **31**, **37**, and **40**. PC-3 cells were incubated with the test compound for 16 h. As shown in Figure 2, derivatives **11**, **37**, and **40** induced appreciable levels of apoptotic cell death in the androgen-insensitive PC-3 prostate cancer cell line in a dose-responsive manner after 16 h of treatment. Specifically, 5 µM of derivatives **11**, **37**, and **40** could induce a substantial early phase of apoptosis (26–59%) in PC-3 cells compared with control cells: treatment with 10 µM of these three optimal derivatives led to 56–76% early apoptotic cells and 6–40% late apoptotic/necrotic cells; 20 µM of derivatives **11**, **37**, and **40** also activated notable apoptosis, with 54–75% early apoptotic cells and 16–44% late apoptotic/necrotic cells. The apoptotic cell population reached its maximum when PC-3 cancer cells were exposed to derivative **11**, **37**, and **40** at 5µM, 10 µM, and 30 µM, respectively. In contrast, derivatives **29** and **31** did not induce significant levels of apoptotic cell death (less than 10%) up to a 10 µM concentration. Only 50 µM of derivatives **29** and **31** resulted in the maximum apoptotic cell population (71% and 95%, respectively).

## 3. Materials and Methods

### 3.1. General Procedures

HRMS were obtained on an Orbitrap mass spectrometer with electrospray ionization (ESI) (Thermo Fisher Scientific, Waltham, MA, USA). NMR spectra were obtained on a Bruker Fourier 300 spectrometer (Billerica, MA, USA) in CDCl_3_, or DMSO-*d*_6_. The chemical shifts are given in ppm referenced to the respective solvent peak, and coupling constants are reported in Hz. Anhydrous THF and dichloromethane were purified by the PureSolv MD 7 Solvent Purification System from Innovative Technologies (MB-SPS-800) (Herndon, VA, USA). All other reagents and solvents were purchased from commercial sources and were used without further purification. Silica gel column chromatography was performed using silica gel (32–63 µm) (SiliCycle Inc, Quebect, QC, Canada). Preparative thin-layer chromatography (PTLC) separations were carried out on thin layer chromatography plates loaded with silica gel 60 GF254 (EMD Millipore Corporation, MA, USA). Silibinin (>98.0%) was purchased from Fisher Scientific (TCI America, Portland, OR, USA, Cat # 50-014-46874).

### 3.2. Synthesis of 7-O-Benzylsilybin *(**5**)*

Following the procedure described in the literature [23,28], 7-*O*-benzylsilybin (**5**) was prepared from silibinin in an 80% yield as a light yellow solid. m.p. 93–95 °C. IR (film) *ν*_max_: 3432, 2937, 1634, 1571, 1507 cm^−1^; ^1^H-NMR (300 MHz, CDCl_3_): δ 11.25 (11.24) (s, 1H), 7.43–7.34 (m, 5H), 7.19 (dd, *J* = 4.2, 1.5 Hz, 1H), 7.10–7.01 (m, 2H), 6.97–6.88 (overlapped, 3H), 6.21 (d, *J* = 1.8 Hz, 1H), 6.13 (6.12) (d, *J* = 2.4 Hz, 1H), 5.96 (br.s, 1H), 5.07 (s, 2H), 4.99 (d, *J* = 11.7 Hz, 1H), 4.93 (d, *J* = 8.4 Hz, 1H), 4.52 (dd, *J* = 11.7, 3.3 Hz, 1H), 4.09–3.99 (m, 1H), 3.89 (s, 3H), 3.80 (dd, *J* = 12.3, 2.1 Hz, 1H), 3.55 (dd, *J* = 12.3, 3.6 Hz, 1H); ^13^C-NMR (75 MHz, CDCl_3_): δ 196.2, 167.8, 163.6, 162.8, 147.0, 146.4, 144.1, 143.9, 135.6, 129.5, 128.8, 128.4, 127.9, 127.5, 121.2 (121.1), 120.8, 117.3 (117.2), 116.6, 114.9, 109.8, 101.1, 96.4, 95.4, 83.0, 78.4, 76.3, 72.4, 70.5, 61.6, 56.1; HRMS-ESI *m*/*z* [M + H]^+^ calcd for C_32_H_29_O_10_: 573.1761, found: 573.1769.

### 3.3. Synthesis of 7-O-Benzyl-3,5,20-O-Trimethyl-2,3-Dehydrosilybin *(**6**)*

Potassium carbonate (3 eq.) was added to a solution of benzylsilybin (1 eq.) in DMF (0.5 M) and the reaction mixture was exposed to air with stirring at room temperature (or 60 °C) for 3 h. When most of the 7-*O*-benzylsilybin was oxidized to 7-*O*-benzyl-2,3-dehydrobenzylsilybin, as monitored by TLC, the reaction mixture was cooled down to room temperature. Potassium carbonate (3 eq.), followed by methyl iodide (6 eq.), were added to the reaction mixture and the reaction was allowed to proceed at room temperature overnight prior to being quenched with HCl (1 M). The subsequent mixture was diluted with water and extracted with ethyl acetate. The combined organic extracts were rinsed with brine and dried over anhydrous sodium sulfate. After filtration, the volatile components were evaporated under vacuum to give the crude product, which was purified by column chromatography over silica gel or PTLC eluting with 5% methanol in dichloromethane to generate 7-*O*-benzyl-3,5,20-*O*-trimethyl-2,3-dehydrosilybin (**6**): 48% yield, yellow solid, m.p. 118–119 °C. IR (film) *ν*_max_: 3401, 2932, 1601, 1504, 1440 cm^−1^; ^1^H-NMR (300 MHz, CDCl_3_): δ 7.76 (d, *J* = 2.1 Hz, 1H), 7.73 (dd, *J* = 8.7, 2.1 Hz, 1H), 7.48–7.34 (m, 5H), 7.06 (d, *J* = 8.7 Hz,1H), 7.04 (dd, *J* = 8.4, 2.1Hz, 1H), 6.98 (d, *J* = 1.8 Hz, 1H), 6.93 (d, *J* = 8.1 Hz, 1H), 6.56 (d, *J* = 2.1 Hz, 1H), 6.41 (d, *J* = 2.4 Hz, 1H), 5.12 (s, 2H), 5.02 (d, *J* = 8.4 Hz,1H), 4.17–4.10 (m,1H), 3.94 (s, 3H), 3.924 (s, 3H), 3.916 (s, 3H), 3.89 (s, 3H), 3.85 (dd, *J* = 12.3, 2.1 Hz, 1H), 3.58 (dd, *J* = 12.6, 3.9 Hz, 1H); ^13^C-NMR (75 MHz, CDCl_3_): δ 174.2, 163.1, 161.2, 158.8, 152.1, 150.0, 149.6, 145.3, 143.8, 141.6, 135.8, 128.9, 128.6, 128.4, 127.7, 124.5, 122.4, 120.3, 117.3, 117.2, 111.5, 110.3, 109.8, 96.5, 93.4, 78.8, 76.5, 70.6, 61.8, 60.1, 56.6, 56.2, 56.1; HRMS-ESI *m*/*z* [M + H]^+^ calcd for C_35_H_33_O_10_: 613.2074, found: 613.2071.

### 3.4. Synthesis of 3,5,20-O-Trimethyl-2,3-Dehydrosilybin *(**7**)*

To the solution of 7-*O*-benzyl-3,5,20-*O*-trimethyl-2,3-dehydrosilybin (**6**, 1 eq.) in methanol (0.2 M), Pd-C (50% wet, 10% *w*/*w*) and ammonium formate (10 eq.) were sequentially added. The reaction mixture was refluxed overnight under argon. After being cooled to room temperature, the reaction mixture was filtered through silica gel pad eluting with THF. The filtrate was concentrated under vacuum to afford 3,5,20-*O*-trimethyl-2,3-dehydrosilybin (**7**) in 67% yield as a light yellow solid. M.p. 235–236 °C. IR (film) *ν*_max_: 3545, 2955, 2924, 2853, 2177, 2159, 2028, 1992, 1978, 1968, 1728, 1593, 1557, 1508 cm^−1^; ^1^H-NMR (300 MHz, DMSO-*d*_6_): *δ* 7.74–7.64 (m, 1H), 7.60–7.55 (m, 2H), 7.13–6.98 (m, 6H), 6.42 (s, 1H), 6.33 (s, 1H), 5.02 (d, *J* = 7.8 Hz, 1H), 4.36–4.28 (m, 1H), 3.78 (s, 12H), 3.76 (s, 3H), 3.57 (dd, *J* = 12.9, 2.1 Hz, 1H), 3.35 (dd, *J* = 12.9, 4.8 Hz, 1H). HRMS-ESI *m*/*z* [M + H]^+^ calcd for C_28_H_27_O_10_: 523.1604, found: 523.1598.

### 3.5. Synthesis of 7-O-(3′-Bromo)Propyl-3,5,20-O-Trimethyl-2,3-Dehydrosilybin *(**8**)*

To a solution of 3,5,20-*O*-trimethyl-2,3-dehydrosilybin in DMF (1 M), potassium carbonate (4 eq.) followed by 1,3-dibromopropane (4 eq.) were added. The reaction mixture was stirred at 60 °C overnight prior to being quenched by the addition of 1 M HCl. The subsequent reaction mixture was diluted with water and extracted with ethyl acetate. The combined organic extracts were rinsed with brine, dried over anhydrous sodium sulfate, and concentrated under vacuum to yield a crude product, which was subjected to column chromatography or PTLC purification over silica gel eluting with 5% methanol in dichloromethane to afford 7-*O*-(3′-bromo)propyl-3,5,20-*O*-trimethyl-2,3-dehydrosilybin (**8**) as a yellow solid. m.p. 115–116 °C. IR (film) *ν*_ma_x: 2932, 1604, 1506, 1463, 1441, 1345, 1264 cm^−1^; ^1^H-NMR (300 MHz, CDCl_3_): *δ* 7.76 (d, *J* = 2.1 Hz, 1H), 7.73 (dd, *J* = 8.7, 2.4 Hz, 1H), 7.07–6.97 (m, 3H), 6.92 (d, *J* = 8.4 Hz, 1H), 6.48 (d, *J* = 2.1 Hz, 1H), 6.32 (d, *J* = 2.1 Hz, 1H), 5.02 (d, *J* = 8.4 Hz, 1H), 4.20–4.11 (m, 1H), 4.17 (t, *J* = 5.7 Hz, 2H), 3.95 (s, 3H), 3.92 (s, 3H), 3.91 (s, 3H), 3.89 (s, 3H), 3.85 (dd, *J* = 12.6, 2.7 Hz, 1H), 3.66-3.56 (m, 1H), 3.61 (t, *J* = 6.3 Hz, 2H), 2.36 (quin, *J* = 6.0 Hz, 2H); ^13^C-NMR (75 MHz, CDCl_3_): δ 174.2, 163.0, 161.2, 158.8, 152.1, 149.9, 149.6, 145.3, 143.8, 141.5, 128.4, 124.4, 122.3, 120.3, 117.3, 117.2, 111.4, 110.2, 109.7, 96.1, 93.0, 78.8, 76.4, 66.0, 61.8, 60.1, 56.5, 56.2, 56.1, 32.1, 29.6; HRMS-ESI *m*/z [M + H]^+^ calcd for C_31_H_32_BrO_10_: 643.1179, 645.1158, found: 643.1173, 645.1151.

### 3.6. General Procedure for the Synthesis of 7-O-(N,N-Dialkylamino)Propyl-3,5,20-O-Trimethyl-2,3-Dehydrosilybins

Potassium carbonate (3 eq.) and the appropriate amine (3 eq.) were added to a solution of 7-*O*-(3′-bromo)propyl-3,5,20-*O*-trimethyl-2,3-dehydrosilybin (**8**) in dry acetone (0.1 M). The reaction mixture was refluxed overnight before the removal of acetone under vacuum. The residue was diluted with water and extracted with ethyl acetate. The combined organic extracts were rinsed with brine, dried over anhydrous sodium sulfate, and concentrated in vacuo to generate the crude products, which were subjected to PTLC purification eluting with 10% methanol in dichloromethane to yield the respective 7-*O*-(3′-amino)propyl-3,5,20-*O*-trimethyl-2,3-dehydrosilybin. 

*7-O-(N-Methylaminopropyl)-3,5,20-O-trimethyl-2,3-dehydrosilybin* (**9**). 40% yield, light yellow solid, m.p. 125–127 °C. IR (film) *ν*_max_: 3401, 2928, 1625, 1606, 1507 cm^−1^; ^1^H-NMR (300 MHz, CDCl_3_): *δ* 7.59 (d, *J* = 2.1 Hz, 1H, H-13), 7.53 (dd, *J* = 8.7, 2.1 Hz, 1H, H-15), 7.04 (dd, *J* = 8.1, 1.2 Hz, 1H, H-16), 6.97 (d, *J* = 1.5 Hz, 1H, H-18), 6.93 (dd, *J* = 8.7, 2.7 Hz, 2H, H-21 & H-22), 6.22 (d, *J* = 1.8 Hz, 1H, H-8), 6.18 (d, *J* = 1.8 Hz, 1H, H-6), 4.99 (d, *J* = 8.4 Hz, 1H, H-11), 4.17–4.06 (overlapped, 3H, H-10 & 7-O*CH_2_*), 3.92 (s, 3H, OCH_3_), 3.91 (s, 3H, OCH_3_), 3.90 (s, 3H, OCH_3_), 3.80 (s, 3H, OCH_3_), 3.85–3.80 (overlapped, 1H, H-23), 3.57 (dd, *J* = 12.9, 3.9 Hz, 1H, H-23), 3.25 (t, *J* = 6.3 Hz, 2H, 7-*O*-CH_2_CH_2_*CH_2_*-), 2.81 (s, 3H, NH*CH_3_*), 2.34–2.30 (m, 2H, 7-*O*-CH_2_*CH_2_*CH_2_-), 2.01 (d, *J* = 1.2 Hz, 1H, 23-OH); ^13^C-NMR (75 MHz, CDCl_3_): δ 174.4 (C-4), 162.9 (C-7), 160.4 (C-5), 158.1 (C-8a), 152.6 (C-19), 150.0 (C-20), 149.6 (C-16a), 145.6 (C-2), 143.7 (C-12a), 140.9 (C-3), 128.3 (C-17), 123.5 (C-14), 122.0 (C-15), 120.3 (C-22), 117.2 (C-16), 117.1 (C-13), 111.5 (C-21), 110.4 (C-18), 108.7 (C-4a), 96.3 (C-6), 92.9 (C-8), 78.9 (C-10), 76.3 (C-11), 65.2 (7-O*CH_2_*), 61.6 (C-23), 59.9 (O*CH_3_*), 56.8 (O*CH_3_*), 56.2 (O*CH_3_*), 56.1 (O*CH_3_*), 47.5 (7-*O*-CH_2_*CH_2_*CH_2_-), 34.2 (NH*CH_3_*), 25.7 (7-*O*-CH_2_*CH_2_*CH_2_-); HRMS-ESI *m*/*z* [M + H]^+^ calcd for C_32_H_36_NO_10_: 594.2339, found: 594.2334. The yields and spectral data for compounds **10**–**14** are included in Supporting Information.

### 3.7. Synthesis of 5,7,20-O-Trimethylsilybin *(**15**)*

A three-neck round bottom flask was charged with silibinin (2.01 g, 4.2 mmol) and potassium carbonate (3.43 g, 25.1 mmol), which was vacuumed three times under argon prior to the addition of acetone (30.0 mL). The reaction mixture was refluxed for 15 min before dimethylsulfate (3.13 mL, 33.1 mmol) was added through a needle. The reaction was continued with refluxing for an additional 4 h when the reaction was completed, as monitored by TLC. After cooling down to room temperature, saturated ammonium chloride was added to quench the reaction, and the subsequent mixture was extracted with ethyl acetatethree times. The organic layers were combined, washed with brine twice, and dried over anhydrous sodium sulfate. Purification of the crude product through column chromatography, eluting with ethyl acetate/hexane (50/50 to 70/30, *v*/*v*), gave the product (**15**) as a white crystal in 80% yield. ^1^H-NMR (300 MHz, CDCl_3_): δ 7.21 (dd, *J* = 9.9, 1.8 Hz, 1H), 7.08(dd, *J* = 8.4, 2.1 Hz, 1H), 7.04 (d, *J* = 3.3 Hz, 1H), 7.00 (dd, *J* = 8.7, 2.7 Hz, 1H), 6.96 (s, 1H), 6.90 (d, *J* = 8.4 Hz, 1H), 6.12 (d, *J* = 2.1 Hz, 1H), 6.11 (d, *J* = 1.5 Hz, 1H), 4.98 (d, *J* = 9.0 Hz, 1H), 4.94 (d, *J* = 12.3 Hz, 1H), 4.42 (dd, *J* = 12.3, 5.1 Hz, 1H), 4.06–4.03 (m, 1H), 3.91 (s, 3H), 3.90 (s, 3H), 3.90 (s, 3H), 3.81 (d, *J* = 1.8 Hz, 3H), 3.81 (dd, *J* = 12.0, 2.4 Hz, 1H), 3.55 (dd, *J* = 12.3, 3.9 Hz).

### 3.8. Synthesis of 5,7,20-O-Trimethyl-2,3-Dehydrosilybin *(**16**)*

A 10 mL round flask was charged with 5,7,20-*O*-trimethylsiybin (150.0 mg, 0.23 mmol) in methanol (2.0 mL) and tetrahydrofuran (2.0 mL). The solution was stirred for 10 min at room temperature prior to being added to hydrogen peroxide (0.85 mL, 30%) and sodium hydroxide aqueous solution (0.65 mL, 16%) at 0 °C. The reaction mixture was slowly warmed to room temperature and then stirred overnight, before being quenched with saturated ammonium chloride. The subsequent mixture was extracted with dichloromethane three times, and the combined extracts were dried over sodium sulfate and concentrated under vacuum. The crude product was obtained in 49% yield, which is pure enough for the next step of the reaction without purification. ^1^H-NMR (300 MHz, CDCl_3_): δ 7.89 (s, 1H), 7.86 (d, *J* = 8.4 Hz, 1H), 7.14 (d, *J* = 8.7 Hz, 1H), 7.04 (d, *J* = 8.4 Hz, 1H), 6.98 (s, 1H), 6.93 (d, *J* = 8.1 Hz, 1H), 6.53 (d, *J* = 1.8 Hz, 1H), 6.35 (d, *J* = 2.1 Hz, 1H), 5.03 (d, *J* = 8.1 Hz, 1H), 4.17–4.11 (m, 1H), 3.98 (s, 3H), 3.93 (s, 3H), 3.92 (s, 3H), 3.90 (s, 3H), 3.85 (d, *J* = 13.8 Hz, 1H), 3.59 (d, *J* = 10.5 Hz, 1H).

### 3.9. General Procedure for the Synthesis of 3-O-Bromoalkyl-5,7,20-O-Trimethyl-2,3-Dehydrosilybins *(**17**–**19**)*

A round bottom flask (10 mL) was charged with 5,7,20-*O*-trimethyl-2,3-dehydrosilybin (**16**, 83.2 mg, 0.16 mmol), potassium carbonate (352.0 mg, 2.55 mmol), and DMF (5.0 mL). The mixture was stirred for 10 min prior to being added to 1,3-, 1,4-, or 1,5-dibromalkane (2.56 mmol, 16 equiv.). The reaction was continued with stirring at room temperature for 24–48 h, before the reaction was quenched with water. The subsequent mixture was extracted with ethyl acetate three times, and the combined extracts were dried over anhydrous sodium sulfate and concentrated in vacuo. The crude products were subjected to PTLC purification eluting with DCM/methanol (100/5, *v*/*v*) to yield the respective 3-*O*-bromoalkyl-5,7,10-*O*-trimethyl-2,3-dehydrosilybin. *3-O-(3′-Bromo)propyl-5,7,20-O-trimethyl-2,3-dehydrosilybin* (**17**). 81% yield, ^1^H-NMR (300 MHz, CDCl_3_): δ 7.72 (s, 1H), 7.70 (d, *J* = 9.6 Hz, 1H), 7.07 (d, *J* = 7.8 Hz, 1H), 7.06 (dd, *J* = 8.1, 1.8 Hz, 1H), 7.00 (d, *J* = 1.8 Hz, 1H), 6.94 (d, *J* = 8.4 Hz, 1H), 6.49 (d, *J* = 2.1 Hz, 1H), 6.35 (d, *J* = 2.4 Hz, 1H), 5.05 (d, *J* = 8.4 Hz, 1H), 4.14 (t, *J* = 6.0 Hz, 2H), 4.16–4.12 (overlapped, 1H), 3.96 (s, 3H), 3.94 (s, 3H), 3.93 (s, 3H), 3.89 (s, 3H), 3.86 (dd, *J* = 12.6, 2.7 Hz, 1H), 3.60 (t, *J* = 6.9 Hz, 2H), 3.63–3.58 (overlapped, 1H), 2.31–2.26 (m, 2H). The yields and spectral data for compounds **18**–**19** are included in Supporting Information.

### 3.10. General Procedure for the Synthesis of 3-O-(Alkylamino)Alkyl-5,7,20-O-Trimethyl-2,3-Dehydrosilybins

A round bottom reaction flask (10 mL) was charged with 3-*O*-bromoalkyl-5,7,10-*O*-trimethyl- 2,3-dehydrosilybin (1 eq.) and potassium carbonate (10 eq.) in acetone (2.0 mL, 0.029 M). The solution was stirred for 10 min prior to being added the appropriate amine (16 eq.). The reaction was allowed to proceed with stirring at room temperature for 24–48 h, before being quenched with water. The subsequent mixture was extracted with ethyl acetate three times, and the combined extracts were dried over anhydrous sodium sulfate and concentrated in vacuo. The crude product was subjected to PTLC purification eluting with DCM/methanol (100:5, *v*/*v*). Each desired nitrogen-containing compound was retrieved from PTLC silica gel by washing with dichloromethane/methanol/ammonium hydroxide (100:10:5, *v*/*v*/*v*). 

*3-O-(N,N-Dimethylamino)propyl-5,7,20-O-trimethyl-2,3-dehydrosilybin* (**20**). 97% yield, white solid, white wax. IR (film) *ν*_max_: 3364, 2940, 2837, 1625, 1604, 1517, 1505, 1492, 1462 cm^−1^; ^1^H-NMR (300 MHz, CDCl_3_): δ 7.64 (d, *J* = 6.2 Hz, 1H, H-15), 7.63 (s, 1H, H-13), 7.10 (d, *J* = 10.1 Hz, 1H, H-16), 7.03 (d, *J* = 8.4 Hz, 1H, H-22), 6.98 (s, 1H, H-18), 6.91 (d, *J* = 8.1 Hz, 1H, H-21), 6.47 (s, 1H, H-8), 6.33 (s, 1H, H-6), 5.05 (d, *J* = 7.7 Hz, 1H, H-11), 4.19–4.16 (m, 1H, H-10), 4.00–3.97 (overlapped, 2H, 3-O*CH_2_*), 3.94 (s, 3H, OCH_3_), 3.91 (s, 3H, OCH_3_), 3.90 (s, 3H, OCH_3_), 3.86 (s, 3H, OCH_3_), 3.86–3.83 (overlapped, 1H, H-23), 3.57 (dd, *J* = 12.2, 2.9 Hz, 1H, H-23), 3.52–3.42 (m, 2H, N*CH_2_*), 2.88 (s, 6H, 2 × N*CH_3_*), 2.33–2.22 (m, 2H, 3-OCH_2_*CH_2_*); ^13^C-NMR (75 MHz, CDCl_3_): δ 174.4 (C-4), 164.5 (C-7), 160.8 (C-5), 158.9 (C-8a), 153.4 (C-16a), 149.8 (C-19), 149.4 (C-20), 145.8 (C-2), 143.9 (C-12a), 139.6 C-3), 128.2 (C-17), 123.3 (C-14), 122.2 (C-15), 120.2 (C-22), 117.5 (C-13), 117.0 (C-16), 111.4 (C-21), 110.3 (C-18), 108.9 (C-4a), 96.2 (C-6), 92.5 (C-8), 78.7 (C-10), 76.3 (C-11), 69.3 (3-O*CH_2_*), 61.4 (C-23), 56.5 (OCH_3_), 56.1 (OCH_3_), 56.0 (OCH_3_), 55.9 (OCH_3_), 43.3 (N*CH_2_*), 42.3 (N*CH_3_*), 25.4 (3-*O*-CH_2_*CH_2_*CH_2_-); HRMS-ESI *m*/*z* [M + H]^+^ calcd for C_33_H_38_NO_10_: 608.2496, found: 608.2490. The yields and spectral data for compounds **21**–**45** are included in Supporting Information.

### 3.11. Cell Culture

All cell lines were initially purchased from American Type Culture Collection (ATCC^TM^) (Manassas, VA, USA). The PC-3 and LNCaP prostate cancer cell lines were routinely cultured in RPIM-1640 medium supplemented with 10% FBS and 1% penicillin/streptomycin. The DU145 prostate cancer cells were routinely cultured in Eagle’s Minimum Essential Medium (EMEM) supplemented with 10% FBS and 1% penicillin/streptomycin. Cultures were maintained in a high humidity environment supplemented with 5% carbon dioxide at a temperature of 37 °C.

### 3.12. WST-1 Cell Proliferation Assay

PC-3, DU145, or LNCaP cells were plated in 96-well plates at a density of 3,200 per well in 200 µL of culture medium. The cells were then separately treated with silibinin, or synthesized silibinin derivatives, at five different doses for three days, while equal treatment volumes of DMSO (0.25%) were used as the vehicle control. The cells were cultured in a CO_2_ incubator at 37 °C for three days. A total of 10 µL of the premixed WST-1 cell proliferation reagent (Clontech) was added to each well. After mixing gently for one min on an orbital shaker, the cells were incubated for an additional 3 h at 37 °C. To ensure a homogeneous distribution of color, it is important to mix gently on an orbital shaker for one min. The absorbance of each well was measured using a microplate reader (Synergy HT, BioTek, Winooski, VT, USA) at a wavelength of 430 nm. The IC_50_ value is the concentration of each compound that inhibits cell proliferation by 50% under the experimental conditions and is the average of at least triplicate determinations so is reproducible and statistically significant. For calculating the IC_50_ values, a linear proliferative inhibition was made based on at least five dosages for each compound.

### 3.13. Cell Cycle Analysis

PC-3 cells were plated in 24-well plates at a density of 200,000 per well in 400 µL of culture medium. After 3 h of cell attachment, the cells were then treated with compound 30 at 5 μM for 15 h, while equal treatment volumes of DMSO were used as the vehicle control. The cells were cultured in a CO_2_ incubator at 37 °C for 22 h and 31 h, respectively. Both attached and floating cells were collected in a centrifuge tube by centrifugation at an rcf value of 450 g for 5 min. After discarding the supernatant, the collected cells were re-suspended with 500 µL 80% cold ethanol to fix them for 30 min in 4 °C. The fixed cells could be stored at −20 °C for one week. After fixation, the ethanol was removed after centrifuging and the cells were washed with PBS. The cells were then re-suspend with 100 μL of 100 mg/mL ribonuclease and were cultured at 37 °C for 30 min to degrade all RNA. The cells were stained with 200 μL of 50 μg/mLpropidium iodide stock solution for 30 min at −20 °C, and then the fluorescence intensity of PI was detected in individual PC-3 cells using an Attune flow cytometer (Life Technologies, Carlsbad, CA, USA) within 0.5 to 1 h after staining.

### 3.14. F2N12S and SYTOX AADvanced Double Staining Assay

PC-3 cells were plated in 24-well plates at a density of 200,000 per well in 400 µL of culture medium. After 3 h of cell attachment, the cells were then treated with each test compound at different concentrations for 15 h, while equal treatment volumes of DMSO were used as the vehicle control. The cells were cultured in a CO_2_ incubator at 37 °C for 15 h. Both attached and floating cells were collected in a centrifuge tube by centrifugation at an rcf value of 450 g for 5 to 6 min. The collected cells were re-suspended with 500 µL HBSS to remove proteins which may affect the flow signal and were centrifuged again. After discarding the supernatant, the collected cells were re-suspended with 300 µL HBSS and stained with 0.3 µL of F2N12S for 3–5 min, followed by 0.3 µL SytoxAAdvanced for an additional 5 min. The fluorescence intensity of the two probes was further measured in individual PC-3 cells using an Attune flow cytometer (Life Technologies) 0.5 to 1 h after staining.

### 3.15. Statistical Analysis:

All data are represented as the mean ± standard deviation (SD) for the number of experiments indicated. Other differences between treated and control groups were analyzed using the Student’s t-test. A *p*-value < 0.05 was considered statistically significant.

## 4. Conclusions

In summary, six 7-*O*-aminoalkyl-3,5-20-*O*-trimethyl-2,3-dehydrosilybins and 26 3-*O*-aminoalkyl-5,7,20-*O*-trimethyl-2,3-dehydrosilybins have been successfully synthesized through a five-step and four-step sequence, respectively. The synthetic methods can be used for the general synthesis of 7-*O*-substituted-3,5,20-*O*-trimethyl-2,3-dehydrosilybins and 3-*O*-substituted-5,7,20-O- trimethyl-2,3-dehydrosilybins. The antiproliferative activities of the 32 derivatives against three prostate cancer cell lines have been evaluated using the WST-1 cell proliferation assay. All of them showed better prostate cancer cell proliferation inhibition than silybin. Derivatives **11**, **29**, **31**, **37**, and **40** were identified as the optimal derivatives, with IC_50_ values in the range of 1.40–3.06 µM toward these three prostate cancer cell lines, representing a 17- to 52-fold improvement in potency compared to silibinin. All these five optimal derivatives can cause PC-3 cell accumulation in a G_0_/G_1_ phase by increasing the cell population in this phase at 16 h. Derivatives **11**, **37**, and **40** show a stronger ability than derivatives **29** and **31** in activating PC-3 cell apoptosis by inducing appreciable levels of apoptotic cell death at a 5 µM concentration after 16 h of treatment. In contrast, derivatives **29** and **31** did not induce significant levels of apoptotic cell death (less than 10%) up to a 10 µM concentration.

## Figures and Tables

**Table 1:** Anti-proliferative activity of dialkylaminoalkyl-2,3-dehydrosilybins.

Compound	IC_50_ (µM) ^a^			IC_50_ (Silibinin)/IC_50_(Derivative)		
	PC-3 ^b^	DU145 ^c^	LNCaP ^d^	PC-3^b^	DU145 ^c^	LNCaP ^d^
**Docetaxel**	0.0019 ± 0.0006	0.0012 ± 0.0003	0.0002 ± 0.0001	-	-	-
**Silibinin**	72.65 ± 3.15	93.34 ± 13.67	43.73 ± 10.90	-	-	-
**2** [23]	9.45 ± 0.56	11.48 ± 1.42	3.09 ± 1.30	8	8	14
**3** [24]	3.25 ± 0.31	7.59 ± 0.66	2.58 ± 0.07	22	12	17
**4** [24]	1.71 ± 0.45	11.04 ± 0.68	2.07 ± 0.18	42	8	21
**8**	26.09 ± 3.58	11.47 ± 2.38	5.71 ± 2.13	3	8	8
**9**	42.64 ± 6.61	39.64 ± 9.49	12.08 ± 1.81	2	2	4
**10**	9.92 ± 0.43	8.62 ± 0.32	7.49 ± 0.16	7	11	6
**11**	1.40 ± 0.17	1.84 ± 0.14	1.82 ± 0.14	52	51	24
**12**	25.05 ± 1.00	19.59 ± 0.47	11.00 ± 0.99	3	5	4
**13**	26.47 ± 1.00	45.10 ± 11.53	12.72 ± 6.28	3	2	3
**14**	25.65 ± 3.39	19.42 ± 0.88	16.35 ± 3.47	3	5	3
**19**	5.76 ± 1.36	8.13 ± 0.42	5.19 ± 2.08	13	11	8
**20**	9.09 ± 1.48	32.71 ± 5.32	20.69 ± 5.34	8	3	2
**21**	5.15 ± 2.13	9.97 ± 3.34	5.13 ± 0.89	14	9	9
**22**	3.47 ± 2.23	6.73 ± 0.45	5.07 ± 0.52	21	14	9
**23**	5.16 ± 0.94	9.21 ± 0.37	3.09 ± 0.15	14	10	14
**24**	5.76 ± 1.36	8.18 ± 0.42	5.19 ± 2.08	11	8	13
**25**	6.41 ± 0.40	6.64 ± 0.54	6.33 ± 0.34	11	14	7
**26**	2.03 ± 0.69	8.39 ± 1.38	3.89 ± 0.54	36	11	11
**27**	3.77 ± 0.41	5.39 ± 0.53	4.02 ± 1.55	19	17	11
**28**	3.30 ± 0.86	5.78 ± 1.36	2.70 ± 0.47	22	16	16
**29**	2.73 ± 0.12	2.51 ± 0.04	2.21 ± 0.17	27	37	20
**30**	3.77 ± 0.41	3.07 ± 0.51	3.84 ± 0.51	19	30	11
**31**	2.84 ± 0.10	2.85 ± 0.23	2.51 ± 0.31	26	33	17
**32**	2.86 ± 0.79	14.12 ± 2.79	5.89 ± 0.30	25	7	7
**33**	7.49 ± 1.98	19.84 ± 2.38	8.06 ± 1.44	10	5	5
**34**	2.95 ± 0.76	5.90 ± 1.30	3.92 ± 1.67	25	16	11
**35**	2.37 ± 0.70	7.26 ± 1.12	2.28 ± 0.42	31	13	19
**36**	24.09 ± 10.55	66.96 ± 13.65	27.30 ± 5.42	3	1	2
**37**	2.71 ± 0.23	2.69 ± 0.03	1.99 ± 0.23	27	35	22
**38**	2.94 ± 0.13	10.60 ± 0.63	1.74 ± 0.97	25	9	25
**39**	3.49 ± 0.24	6.36 ± 0.37	2.84 ± 0.22	21	15	15
**40**	2.72 ± 0.08	3.06 ± 0.13	2.23 ± 0.18	27	31	20
**41**	9.98 ± 4.94	>50	11.41 ± 5.91	7	<2	4
**42**	5.30 ± 0.66	6.85 ± 0.77	4.58 ± 1.77	14	14	10
**43**	7.87 ± 1.47	48.12 ± 16.27	14.39 ± 7.49	9	2	3
**44**	8.45 ± 2.89	18.57 ± 7.67	12.80 ± 7.80	9	5	3
**45**	4.72 ± 0.88	8.59 ± 2.23	5.66 ± 0.44	15	11	8

^a^ IC_50_ value is the compound concentration effective at inhibiting 50% of the cell viability measured by the WST-1 cell proliferation assay after three days of exposure. The data are presented as the mean ± SD from *n* = 3. ^b^ Human androgen-insensitive prostate cancer cell line derived from bone metastasis of a prostate tumor. ^c^ Human androgen-insensitive prostate cancer cell line derived from brain metastasis of a prostate tumor. ^d^ Human androgen-sensitive prostate cancer cell line derived from lymph node metastasis of a prostate tumor.

**Table 2:** Antiproliferative activity of selected derivatives against MCF 10A and PWR-1E cells.

Compound	IC_50_ (µM) ^a^				
	PC-3	DU145	LNCaP	MCF 10A	PWR-1E
**Silibinin**	72.65 ± 3.15	93.34 ± 13.67	43.73 ± 10.90	23.84 ± 0.96	20.45 ± 4.09
**11**	1.40 ± 0.17	1.84 ± 0.14	1.82 ± 0.14	>50	>50
**29**	2.73 ± 0.12	2.51 ± 0.04	2.21 ± 0.17	<5	<5
**31**	2.84 ± 0.10	2.85 ± 0.23	2.51 ± 0.31	<5	<5
**37**	2.71 ± 0.23	2.69 ± 0.03	1.99 ± 0.23	<5	<5
**40**	2.72 ± 0.08	3.06 ± 0.13	2.23 ± 0.18	<5	<5

^a^ IC_50_ is the drug concentration effective at inhibiting 50% of the cell viability measured by the WST-1 cell proliferation assay after three days of exposure. The data are presented as the mean ± SD from *n* = 3.

## Supplementary Materials

The following are available online at http://www.mdpi.com/1420-3049/23/12/3142/s1, The yields and spectral data for compounds **10**–**14**, **18**–**19**, and **21**–**45**; NMR-spectra (^1^H and ^13^C) of the new silybin derivatives.

## References

[1] Kim N.-C., Graf T.N., Sparacino C.M., Wani M.C., Wall M.E. (2003). Complete isolation and characterization of silybins and isosilybins from milk thistle (*Silybum marianum*). Org. Biomol. Chem..

[2] Fabio G.D., Romanucci V., Marino C.D., De Napoli L., Zarrelli A. (2013). A rapid and simple chromatographic separation of diastereomers of silibinin and their oxidation to produce 2,3-dehydrosilybin enantiomers in an optically pure form. Planta Med..

[3] Althagafy H.S., Meza-Avina M.E., Oberlies N.H., Croatt M.P. (2013). Mechanistic study of the biomimetic synthesis of flavonolignan diastereoisomers in milk thistle. J. Org. Chem..

[4] Pelter A., Haensel R. (1968). The structure of silybin (Silybum substance E6), the first flavonolignan. Tetrahedron Lett..

[5] Kroll D.J., Shaw H.S., Oberlies N.H. (2007). Milk thistle nomenclature: Why it matters in cancer research and pharmacokinetic studies. Integr. Cancer Ther..

[6] Carrier D.J., Crowe T., Sokhansanj S., Wahab J., Barl B. (2002). Milk thistle, *Silybum marianum* (L.) Gaertn., flower head development and associated marker compound profile. J. Herbs Spices Med. Plants.

[7] Abenavoli L., Capasso R., Milic N., Capasso F. (2010). Milk thistle in liver diseases: Past, present, future. Phytother. Res..

[8] Biedermann D., Vavrikova E., Cvak L., Kren V. (2014). Chemistry of silybin. Nat. Prod. Rep..

[9] Flora K., Hahn M., Rosen H., Benner K. (1998). Milk thistle (*Silybum marianum*) for the therapy of liver disease. Am. J. Gastroenterol..

[10] Agarwal R., Katiyar S.K., Lundgren D.W., Mukhtar H. (1994). Inhibitory effect of silymarin, an anti-hepatotoxic flavonoid, on 12-*O*-tetradecanoylphorbol-13-acetate-induced epidermal ornithine decarboxylase activity and mRNA in SENCAR mice. Carcinogenesis.

[11] Lahiri-Chatterjee M., Katiyar S.K., Mohan R.R., Agarwal R. (1999). A flavonoid antioxidant, silymarin, affords exceptionally high protection against tumor promotion in the SENCAR mouse skin tumorigenesis model. Cancer Res..

[12] Agarwal R., Agarwal C., Ichikawa H., Singh R.P., Aggarwal B.B. (2006). Anticancer potential of silymarin: From bench to bed side. Anticancer Res..

[13] Vue B., Zhang S., Chen Q.-H. (2016). Flavonoids with therapeutic potential in prostate cancer. Anticancer Agents Med. Chem..

[14] Davis-Searles P.R., Nakanishi Y., Kim N.-C., Graf T.N., Oberlies N.H., Wani M.C., Wall M.E., Agarwal R., Kroll D.J. (2005). Milk thistle and prostate cancer: Differential effects of pure flavonolignans from Silybum marianum on antiproliferative end points in human prostate carcinoma cells. Cancer Res..

[15] Vue B., Chen Q.-H. (2016). The potential of flavonolignans in prostate cancer management. Curr. Med. Chem..

[16] Flaig T.W., Gustafson D.L., Su L.J., Zirrolli J.A., Crighton F., Harrison G.S., Pierson A.S., Agarwal R., Glode L.M. (2007). A phase I and pharmacokinetic study of silybin-phytosome in prostate cancer patients. Invest. New Drugs.

[17] Zheng D., Wang Y., Zhang D., Liu Z., Duan C., Jia L., Wang F., Liu Y., Liu G., Hao L. (2011). In vitro antitumor activity of silybin nanosuspension in PC-3 cells. Cancer Lett..

[18] Sy-Cordero A.A., Graf T.N., Runyon S.P., Wani M.C., Kroll D.J., Agarwal R., Brantley S.J., Paine M.F., Polyak S.J., Oberlies N.H. (2013). Enhanced bioactivity of silybin B methylation products. Bioorg. Med. Chem..

[19] Agarwal C., Wadhwa R., Deep G., Biedermann D., Gazak R., Kren V., Agarwal R. (2013). Anti-cancer efficacy of silybin derivatives – A structure-activity relationship. PLoS ONE.

[20] Romanucci V., Agarwal C., Agarwal R., Pannecouque C., Iuliano M., De Tommaso G., Caruso T., Di Fabio G., Zarrelli A. (2018). Silibinin phosphodiester glycol-conjugates: Synthesis, redox behavior and biological investigations. Bioorg. Chem..

[21] Yang L.X., Huang K.X., Li H.B., Gong J.X., Wang F., Feng Y.B., Tao Q.F., Wu Y.H., Li X.K., Wu X.M. (2009). Design, synthesis, and examination of neuron protective properties of alkenylated and amidated dehydro-silybin derivatives. J. Med. Chem..

[22] Manivannan E., Amawi H., Hussein N., Karthikeyan C., Fetcenko A., Narayana Moorthy N.S.H., Trivedi P., Tiwari A.K. (2017). Design and discovery of silybin analogues as antiproliferative compounds using a ring disjunctive – Based, natural product lead optimization approach. Eur. J. Med. Chem..

[23] Vue B., Zhang S., Zhang X., Parisis K., Zhang Q., Zheng S., Wang G., Chen Q.-H. (2016). Silibinin derivatives as anti-prostate cancer agents: Synthesis and cell-based evaluations. Eur. J. Med. Chem..

[24] Zhang S., Vue B., Huang M., Zhang X., Lee T., Zhang Q., Zheng S., Wang G., Chen Q.-H. (2016). 3-*O*-Alkyl-2,3-dehydrosilibinins: Two synthetic approaches and in vitro effects toward prostate cancer cells. Bioorg. Med. Chem. Lett..

[25] Di Fabio G., Romanucci V., De Nisco M., Pedatella S., Di Marino C., Zarrelli A. (2013). Microwave-assisted oxidation of silibinin: A simple and preparative method for the synthesis of improved radical scavengers. Tetrahedron Lett..

[26] Gazak R., Svobodova A., Psotova J., Sedmera P., Prikrylova V., Walterova D., Kren V. (2004). Oxidized derivatives of silybin and their antiradical and antioxidant activity. Bioorg. Med. Chem..

[27] Zarrelli A., Sgambato A., Petito V., De Napoli L., Previtera L., Di Fabio G. (2011). New C-23 modified of silybin and 2,3-dehydrosilybin: Synthesis and preliminary evaluation of antioxidant properties. Bioorg. Med. Chem. Lett..

[28] Dzubak P., Hajduch M., Gazak R., Svobodova A., Psotova J., Walterova D., Sedmera P., Kren V. (2006). New derivatives of silybin and 2,3-dehydrosilybin and their cytotoxic and P-glycoprotein modulatory activity. Bioorg. Med. Chem..

[29] Psyzkova M., Biler M., Biedermann D., Valentova K., Kuzma M., Vrba J., Ulrichova J., Sokolova R., Mojovic M., Popovic-Bijelic A. (2016). Flavonolignan 2,3-dehydroderivatives: Preparation, antiradical and cytoprotective activity. Free Radic. Biol. Med..

[30] Vue B., Zhang X., Lee T., Nandini N., Zhang S., Chen G., Zhang Q., Zheng S., Wang G., Chen Q.-H. (2017). 5- or/and 20-*O*-Alkyl-2,3-dehydrosilybins: Synthesis and biological profiles on prostate cancer cell models. Bioorg. Med. Chem..

[31] Tyagi A., Bhatia N., Condon M.S., Bosland M.C., Agarwal C., Agarwal R. (2002). Antiproliferative and apoptotic effects of silibinin in rat prostate cancer cells. Prostate.

[32] Zi X., Agarwal R. (1999). Silibinin decreases prostate-specific antigen with cell growth inhibition via G1 arrest, leading to differentiation of prostate carcinoma cells: Implications for prostate cancer intervention. Proc. Natl. Acad. Sci. USA.

[33] Deep G., Singh R.P., Agarwal C., Kroll D.J., Agarwal R. (2006). Silymarin and silibinin cause G1 and G2-M cell cycle arrest via distinct circuitries in human prostate cancer PC3 cells: A comparison of flavone silibinin with flavanolignan mixture silymarin. Oncogene.

[34] Singh R.P., Deep G., Blouin M.-J., Pollak M.N., Agarwal R. (2007). Silibinin suppresses in vivo growth of human prostate carcinoma PC-3 tumor xenograft. Carcinogenesis.

