# Supplementary material for: O-Aminoalkyl-O-Trimethyl-2,3-Dehydrosilybins: Synthesis and In Vitro Effects Towards Prostate Cancer Cells
Source: Molecules. 2018 Nov 29;23(12):3142. doi: 10.3390/molecules23123142 (PMC6320956; doi:10.3390/molecules23123142)
Supplement: Supplementary file 1 [file molecules-23-03142-s001.pdf]

Supplementary Data

O-Aminoalkyl-*O*-Trimethyl-2,3-Dehydrosilybins: Synthesis and *in vitro* Effects toward Prostate Cancer Cells

Bao Vue[a], Sheng Zhang[a], Andre Vignau[a], Guanglin Chen[a], Xiaojie Zhang[a], William Diaz[a], Qiang Zhang[b,c], Shilong Zheng[b,c], Guangdi Wang[b,c], and Qiao-Hong Chen[a,]*

[a]Department of Chemistry, California State University, Fresno, 2555 E. San Ramon Avenue, M/S SB70, Fresno, CA 93740, USA
[b]Department of Chemistry and [c]RCMI Cancer Research Center, Xavier University of Louisiana, 1 Drexel Drive, New Orleans, LA 70125, USA

List of contents:

Yields and spectral data for compounds **10-14**, **18**, **19**, and **21-45**……………………S4-S13
^{1}H NMR spectrum of **9** in $CDCl_3$…………………………………………………..….S14
^{13}C NMR spectrum of **9** in $CDCl_3$………….……………………………………..….S15
^{1}H NMR spectrum of **10** in $CDCl_3$………………………………………………….…...S16
^{13}C NMR spectrum of **10** in $CDCl_3$………….………………………………..…...S17
^{1}H NMR spectrum of **11** in $CDCl_3$…………………………………………………….S18
^{13}C NMR spectrum of **11** in $CDCl_3$………….……………………………………….S19
^{1}H NMR spectrum of **12** in $CDCl_3$…………………………………………………….S20
^{13}C NMR spectrum of **12** in $CDCl_3$………….……………………………………….S21
^{1}H NMR spectrum of **13** in $CDCl_3$…………………………………………………….S22
^{13}C NMR spectrum of **13** in $CDCl_3$………….……………………………………….S23
^{1}H NMR spectrum of **14** in $CDCl_3$…………………………………………………….S24
^{13}C NMR spectrum of **14** in $CDCl_3$………….……………………………………….S25
^{1}H NMR spectrum of **20** in $CDCl_3$…………………………………………………….S26
^{13}C NMR spectrum of **20** in $CDCl_3$………….……………………………………….S27
^{1}H NMR spectrum of **21** in $CDCl_3$…………………………………………………….S28
^{13}C NMR spectrum of **21** in $CDCl_3$………….……………………………………….S29
^{1}H NMR spectrum of **22** in $CDCl_3$…………………………………………………….S30
^{13}C NMR spectrum of **22** in $CDCl_3$………….……………………………………….S31
^{1}H NMR spectrum of **23** in $CDCl_3$…………………………………………………….S32
^{13}C NMR spectrum of **23** in $CDCl_3$………….……………………………………….S33

^{1}H NMR spectrum of **24** in $CDCl_3$……S34
^{13}C NMR spectrum of **24** in $CDCl_3$……S35
^{1}H NMR spectrum of **25** in $CDCl_3$……S36
^{13}C NMR spectrum of **25** in $CDCl_3$……S37
^{1}H NMR spectrum of **26** in $CDCl_3$……S38
^{13}C NMR spectrum of **26** in $CDCl_3$……S39
^{1}H NMR spectrum of **27** in $CDCl_3$……S40
^{13}C NMR spectrum of **27** in $CDCl_3$……S41
^{1}H NMR spectrum of **28** in $CDCl_3$……S42
^{13}C NMR spectrum of **28** in $CDCl_3$……S43
^{1}H NMR spectrum of **29** in $CDCl_3$……S446
^{13}C NMR spectrum of **29** in $CDCl_3$……S45
^{1}H NMR spectrum of **30** in $CDCl_3$……S46
^{13}C NMR spectrum of **30** in $CDCl_3$……S47
^{1}H NMR spectrum of **31** in $CDCl_3$……S48
^{13}C NMR spectrum of **31** in $CDCl_3$……S49
^{1}H NMR spectrum of **32** in $CDCl_3$……S50
^{13}C NMR spectrum of **32** in $CDCl_3$……S51
^{1}H NMR spectrum of **33** in $CDCl_3$……S52
^{13}C NMR spectrum of **33** in $CDCl_3$……S53
^{1}H NMR spectrum of **34** in $CDCl_3$……S54
^{13}C NMR spectrum of **34** in $CDCl_3$……S55
^{1}H NMR spectrum of **35** in $CDCl_3$……S56
^{13}C NMR spectrum of **35** in $CDCl_3$……S57
^{1}H NMR spectrum of **36** in $CDCl_3$……S58
^{13}C NMR spectrum of **36** in $CDCl_3$……S59
^{1}H NMR spectrum of **37** in $CDCl_3$……S60
^{13}C NMR spectrum of **37** in $CDCl_3$……S61
^{1}H NMR spectrum of **38** in $CDCl_3$……S62
^{13}C NMR spectrum of **38** in $CDCl_3$……S63
^{1}H NMR spectrum of **39** in $CDCl_3$……S64
^{13}C NMR spectrum of **39** in $CDCl_3$……S65
^{1}H NMR spectrum of **40** in $CDCl_3$……S66
^{13}C NMR spectrum of **40** in $CDCl_3$……S67
^{1}H NMR spectrum of **41** in $CDCl_3$……S68

^{13}C NMR spectrum of **41** in $CDCl_3$……………...……………………………………………….S69
^{1}H NMR spectrum of **42** in $CDCl_3$……………………………………………………………….S70
^{13}C NMR spectrum of **42** in $CDCl_3$……………...……………………………………………….S71
^{1}H NMR spectrum of **43** in $CDCl_3$……………………………………………………………….S72
^{13}C NMR spectrum of **43** in $CDCl_3$……………...……………………………………………….S73
^{1}H NMR spectrum of **44** in $CDCl_3$……………………………………………………………….S74
^{13}C NMR spectrum of **44** in $CDCl_3$……………...……………………………………………….S75
^{1}H NMR spectrum of **45** in $CDCl_3$……………………………………………………………….S76
^{13}C NMR spectrum of **45** in $CDCl_3$……………...……………………………………………….S77

Corresponding author.

*E-mail: qchen@csufresno.edu. Phone: (+1)5592782394. Fax: (+1)5592784402.

7-O-(N,N-Diethylaminopropyl)-3,5,20-O-trimethyl-2,3-dehydrosilybin (**10**). 29% yield, yellow solid, m.p. 107-109 °C. IR (film) ν_{max}: 3419, 3182, 2981, 2844, 1624, 1602 cm^{-1}; ^1H NMR (300 MHz, CD_3OD): δ 7.59-7.48 (overlapped, 1H, H-15), 7.51 (s, 1H, H-13), 7.03 (s, 1H, H-18), 7.00-6.94 (overlapped, 2H, H-16 and H-22), 6.93 (d, J = 8.4 Hz, 1H, H-21), 6.36 (s, 1H, H-8), 6.23 (s, 1H, H-6), 4.89-4.86 (overlapped, 1H, H-11), 4.09-3.92 (overlapped, 3H, H-10 & 7-OCH_2), 3.84 (s, 3H, OCH_3), 3.83 (s, 3H, OCH_3), 3.78 (s, 3H, OCH_3), 3.75 (s, 3H, OCH_3), 3.69 (dd, J = 12.9, 2.4 Hz, 1H, H-23), 3.43 (dd, J = 12.3, 3.9 Hz, 1H, H-23), 2.74 (t, J = 7.5 Hz, 2H, 7-*O*-$CH_2CH_2CH_2$-), 2.70 (t, J = 7.2 Hz, 2H, NCH_2), 2.67 (t, J = 7.2 Hz, 2H, NCH_2), 2.02-1.86 (m, 2H, 7-*O*-$CH_2CH_2CH_2$-), 1.10 (t, J = 7.2 Hz, 6H, 2 x NCH_2CH_3); ^{13}C NMR (75 MHz, CD_3OD): δ 175.5 (C-4), 165.0 (C-7), 161.7 (C-5), 159.7 (C-8a), 153.9 (C-19), 151.1 (C-20), 150.6 (C-16a), 147.2 (C-2), 145.0 (C-12a), 141.9 (C-3), 130.3 (C-17), 124.4 (C-14), 123.0 (C-15), 121.6 (C-22), 118.1 (C-16), 117.9 (C-13), 112.8 (C-21), 112.3 (C-18), 109.6 (C-4a), 97.0 (C-6), 94.1 (C-8), 80.3 (C-10), 77.5 (C-11), 67.9 (7-OCH_2), 61.9 (C-23), 60.2 (OCH_3), 56.6 (OCH_3), 56.5 (OCH_3), 56.4 (OCH_3), 50.3 (NCH_2), 47.9 (7-*O*-$CH_2CH_2CH_2$-), 26.5 (7-*O*-$CH_2CH_2CH_2$-), 11.0 (NCH_2CH_3); HRMS-ESI *m/z* $[M+H]^+$ calcd for $C_{35}H_{42}NO_{10}$: 636.2808, found: 636.2801.

7-O-(N,N-Dibutylaminopropyl)-3,5,20-O-trimethyl-2,3-dehydrosilybin (**11**). 25% yield, yellow solid, mp. 69-71 °C. IR (film) ν_{max}: 3404, 2930, 2871, 1625, 1602, 1505 cm^{-1}; ^1H NMR (300 MHz, $CDCl_3$): δ 7.75 (d, J = 1.8 Hz, 1H, H-13), 7.72 (dd, J = 8.4, 2.1 Hz, 1H, H-15), 7.04 (d, J = 8.4 Hz, 1H, H-16), 7.03 (dd, J = 8.1, 1.8 Hz, 1H, H-22), 6.97 (d, J = 1.8 Hz, 1H, H-18), 6.92 (d, J = 8.4 Hz, 1H, H-21), 6.46 (d, J = 2.1 Hz, 1H, H-8), 6.31 (d, J = 2.1 Hz, 1H, H-6), 5.02 (d, J = 8.4 Hz, 1H, H-11), 4.13 (dt, J = 8.1, 2.7 Hz, 1H, H-10), 4.08 (t, J = 6.3 Hz, 2H, 7-OCH_2), 3.94 (s, 3H, OCH_3), 3.92 (s, 3H, OCH_3), 3.91 (s, 3H, OCH_3), 3.88 (s, 3H, OCH_3), 3.84 (dd, J = 12.6, 2.7 Hz, 1H, H-23), 3.57 (dd, J = 12.3, 3.6 Hz, 1H, H-23), 2.66 (t, J = 6.3 Hz, 2H, 7-*O*-$CH_2CH_2CH_2$-), 2.49 (t, J = 7.2 Hz, 4H, 2 × NCH_2), 1.98 (quin, J = 6.3 Hz, 2H, 7-*O*-$CH_2CH_2CH_2$-), 1.50-1.40 (m, 4H, 2 × $NCH_2CH_2CH_2CH_3$), 1.36-1.24 (m, 4H, 2 × $NCH_2CH_2CH_2CH_3$), 0.89 (t, J = 7.2 Hz, 6H, 2 × $NCH_2CH_2CH_2CH_3$); ^{13}C NMR (75 MHz, $CDCl_3$): δ 174.2 (C-4), 163.4 (C-7), 161.1 (C-5), 158.8 (C-8a), 152.0 (C-19), 149.9 (C-20), 149.6 (C-16a), 145.3 (C-2), 143.8 (C-12a), 141.5 (C-3), 128.5 (C-17), 124.4 (C-14), 122.3 (C-15), 120.3 (C-22), 117.3 (C-16), 117.2 (C-13), 111.5 (C-21), 110.3 (C-18), 109.5 (C-4a), 96.1 (C-6), 93.0 (C-8), 78.8 (C-10), 76.4 (C-11), 66.8 (7-OCH_2), 61.7 (C-23), 60.0 (OCH_3), 56.5 (OCH_3), 56.2 (OCH_3), 56.1 (OCH_3), 53.9 (NCH_2), 50.5 (7-*O*-$CH_2CH_2CH_2$-), 28.9 ($NCH_2CH_2CH_2CH_3$), 26.7 (7-*O*-$CH_2CH_2CH_2$-), 20.8 ($NCH_2CH_2CH_2CH_3$), 14.2 ($NCH_2CH_2CH_2CH_3$); HRMS-ESI *m/z* $[M+H]^+$ calcd for $C_{39}H_{50}NO_{10}$: 692.3434, found: 692.3431.

7-O-Pyrrolidinopropyl-3,5,20-O-trimethyl-2,3-dehydrosilybin (**12**). 29% yield, yellow solid, mp. 111-113 °C. IR (film) ν_{max}: 3415, 2933, 1625, 1605, 1578, 1506 cm^{-1}; ^1H NMR (300 MHz, $CDCl_3$): δ 7.74 (d, J = 1.8 Hz, 1H, H-13), 7.71 (dd, J = 8.7, 2.1 Hz, 1H, H-15), 7.03 (d, J = 8.7 Hz, 2H, H-16 & H-22), 6.97 (d, J = 1.5 Hz, 1H, H-18), 6.91 (d, J = 8.1 Hz, 1H, H-21), 6.46 (d, J = 2.1 Hz, 1H, H-8), 6.31 (d, J = 2.1 Hz, 1H, H-6), 5.01 (d, J = 8.1 Hz, 1H, H-11), 4.16-4.12 (m, 1H, H-10), 4.09 (t, J = 6.3 Hz, 2H, 7-OCH_2), 3.94 (s, 3H, OCH_3), 3.92 (s, 3H, OCH_3), 3.90 (s, 3H, OCH_3), 3.88 (s, 3H, OCH_3), 3.84 (dd, J = 12.9, 2.4 Hz, 1H, H-23), 3.56 (dd, J = 12.6, 3.9 Hz, 1H, H-23), 2.71 (t, J = 7.2 Hz, 2H, 7-*O*-$CH_2CH_2CH_2$-), 2.67-2.57 (m, 4H, 2 × pyrrolidino-CH_2), 2.07 (quin, J = 6.0 Hz, 2H, 7-*O*-$CH_2CH_2CH_2$-), 1.88-1.79 (m, 4H, 2 × pyrrolidino-CH_2); ^{13}C NMR (75 MHz, $CDCl_3$): δ 174.2 (C-4), 163.3 (C-7), 161.0 (C-5), 158.8 (C-8a), 152.1 (C-19), 149.9 (C-20), 149.6 (C-16a), 145.3 (C-2), 143.8 (C-12a), 141.4 (C-3), 128.5 (C-17), 124.3 (C-14), 122.2 (C-15), 120.3 (C-22), 117.3 (C-16), 117.2 (C-13), 111.4 (C-21), 110.2 (C-18), 109.5 (C-4a), 96.1 (C-6), 93.0 (C-8), 78.8 (C-10), 76.4 (C-11), 66.7 (7-OCH_2), 61.7 (C-23), 60.0 (OCH_3), 56.5 (OCH_3), 56.2 (OCH_3), 56.1 (OCH_3), 54.3 (NCH_2), 53.0 (pyrrolidino-CH_2), 28.0 (7-*O*-$CH_2CH_2CH_2$-), 23.6 (pyrrolidino-CH_2); HRMS-ESI *m/z* $[M+H]^+$ calcd for $C_{35}H_{40}NO_{10}$: 634.2652, found: 634.2650.

7-O-Mopholinopropyl-3,5,20-O-trimethyl-2,3-dehydrosilybin (**13**). 19% yield, yellow solid. mp. 132-133 °C. IR (film) ν_{max}: 3406, 2932, 1625, 1606, 1506, 1464, 1443 cm^{-1}; ^1H NMR (300 MHz, $CDCl_3$): δ 7.73 (s, 1H, H-13), 7.72 (d, J = 10.8 Hz, 1H, H-15), 7.03 (d, J = 8.4 Hz, 2H, H-16 & H-22), 6.97 (s, 1H, H-18), 6.91 (d, J = 8.1 Hz, 1H, H-21), 6.44 (s, 1H, H-8), 6.29 (s, 1H, H-6), 5.02 (d, J = 8.1 Hz, 1H, H-11), 4.18-4.04 (overlapped, 3H, H-10 & 7-OCH_2), 3.93 (s, 3H, OCH_3), 3.91 (s, 3H, OCH_3), 3.90 (s, 3H, OCH_3), 3.87 (s, 3H, OCH_3), 3.87-3.76 (overlapped, 4H, 2 × OCH_2 from mopholino), 3.57 (dd, J = 12.3, 3.3 Hz, 1H, H-23), 3.06-2.99 (m, 1H, H-23), 2.79-2.57 (overlapped, 6H, 3 × NCH_2), 2.18-2.05 (overlapped, 2H, 7-*O*-$CH_2CH_2CH_2$-); ^{13}C NMR (75 MHz, $CDCl_3$): δ 174.2 (C-4), 163.2 (C-7), 161.0 (C-

5), 158.8 (C-8a), 152.1 (C-19), 149.9 (C-20), 149.6 (C-16a), 145.3 (C-2), 143.8 (C-12a), 141.4 (C-3), 128.4 (C-17), 124.3 (C-14), 122.2 (C-15), 120.3 (C-22), 117.23 (C-16), 117.17 (C-13), 111.4 (C-21), 110.2 (C-18), 109.5 (C-4a), 96.1 (C-6), 92.9 (C-8), 78.8 (C-10), 76.4 (C-11), 66.4 (7-O*CH*$_2$), 66.2 O*CH*$_2$ from mopholino), 61.7 (C-23), 60.0 (O*CH*$_3$), 56.5 (O*CH*$_3$), 56.2 (O*CH*$_3$), 56.1 (O*CH*$_3$), 55.4 (N*CH*$_2$), 53.4 (N*CH*$_2$), 42.3 (7-*O*-CH$_2$*CH*$_2$CH$_2$-); HRMS-ESI *m/z* [M+H]$^+$ calcd for $C_{35}H_{40}NO_{11}$: 650.2601, found: 650.2598.

7-O-(4-Methylpiperazin-1-yl)propyl-3,5,20-O-trimethyl-2,3-dehydrosilybin (**14**). 10% yield, light yellow solid. Mp. 142-143 °C. IR (film) ν_{max}: 3406, 2917, 2850, 1625, 1604, 1505 cm^{-1}; ^{1}H NMR (300 MHz, CDCl$_3$): δ 7.77-7.71 (overlapped, 2H, H-13 & H-15), 7.06 (d, *J* = 9.0 Hz, 1H, H-16), 7.04 (dd, *J* = 10.8, 1.5 Hz, 1H, H-22), 6.97 (d, *J* = 1.5 Hz, 1H, H-18), 6.92 (d, *J* = 8.4 Hz, 1H, H-21), 6.44 (d, *J* = 2.1 Hz, 1H, H-8), 6.30 (d, *J* = 1.8 Hz, 1H, H-6), 5.02 (d, *J* = 8.4 Hz, 1H, H-11), 4.17-4.01 (m, 1H, H-10), 4.06 (t, *J* = 6.0 Hz, 2H, 7-O*CH*$_2$), 3.94 (s, 3H, OCH$_3$), 3.92 (s, 3H, OCH$_3$), 3.91 (s, 3H, OCH$_3$), 3.89 (s, 3H, OCH$_3$), 3.85 (dd, *J* = 12.3, 2.1 Hz, 1H, H-23), 3.57 (dd, *J* = 12.6, 3.9 Hz, 1H, H-23), 2.95-2.72 (overlapped, 8H, 4 × NC*H*$_2$ from piperazin), 2.67 (t, *J* = 7.2 Hz, 2H, N*CH*$_2$), 2.55 (s, 3H, N*CH*$_3$), 2.07-2.02 (m, 2H, 7-*O*-CH$_2$*CH*$_2$CH$_2$-); ^{13}C NMR (75 MHz, CDCl$_3$): δ 174.2 (C-4), 163.2 (C-7), 161.1 (C-5), 158.8 (C-8a), 152.0 (C-19), 150.0 (C-20), 149.6 (C-16a), 145.3 (C-2), 143.8 (C-12a), 141.5 (C-3), 128.4 (C-17), 124.4 (C-14), 122.4 (C-15), 120.3 (C-22), 117.2 (C-16), 117.2 (C-13), 111.5 (C-21), 110.3 (C-18), 109.6 (C-4a), 96.2 (C-6), 92.8 (C-8), 78.8 (C-10), 76.5 (C-11), 66.3 (7-O*CH*$_2$), 61.7 (C-23), 60.1 (O*CH*$_3$), 56.6 (O*CH*$_3$), 56.2 (O*CH*$_3$), 56.1 (O*CH*$_3$), 54.3 (N*CH*$_2$), 54.0 (NC*H*$_2$ from piperazin), 50.9 (NC*H*$_2$ from piperazin), 44.5 (N*CH*$_3$), 26.1 (7-*O*-CH$_2$*CH*$_2$CH$_2$-); HRMS-ESI *m/z* [M+H]$^+$ calcd for $C_{36}H_{43}N_2O_{10}$: 663.2917, found: 663.2917.

3-O-(4′-Bromo)butyl-5,7,20-O-trimethyl-2,3-dehydrosilybin (**18**). 62% Yield, ^{1}H NMR (300 MHz, CDCl$_3$): δ 7.71 (s, 1H), 7.69 (dd, *J* = 7.2, 1.5 Hz, 1H), 7.04 (d, *J* = 8.0 Hz, 2H), 6.97 (d, *J* = 1.8 Hz, 1H), 6.92 (d, *J* = 8.1 Hz, 1H), 6.46 (d, *J* = 2.4 Hz, 1H), 6.32 (d, *J* = 2.4 Hz, 1H), 5.03 (d, *J* = 8.1 Hz, 1H), 4.16-4.09 (m, 1H), 4.03 (t, *J* = 6.0 Hz, 2H), 3.93 (s, 3H), 3.91 (s, 3H), 3.90 (s, 3H), 3.86 (s, 3H), 3.81 (dd, *J* = 7.2, 2.4 Hz, 1H), 3.57 (dd, *J* = 12.3, 3.6 Hz, 1H), 3.46 (t, *J* = 6.6 Hz, 2H), 2.09-2.00 (m, 2H), 1.90-1.81 (m, 2H); ^{13}C NMR (75 MHz, CDCl$_3$): δ 174.2, 164.1, 161.1, 158.9, 152.6, 149.9, 149.6, 145.3, 143.8, 140.4, 128.5, 124.4, 122.4, 120.3, 117.3, 117.1, 111.4, 110.3, 109.5, 95.9, 92.5, 78.8, 76.4, 61.7, 56.5, 56.2, 56.1, 55.9, 34.1, 28.8.

3-O-(5′-Bromo)pentyl-5,7,20-O-trimethyl-2,3-dehydrosilybin (**19**). 77% yield, ^{1}H NMR (300 MHz, CDCl$_3$): δ 7.72 (s, 1H), 7.71 (dd, *J* = 7.8, 1.8 Hz, 1H), 7.03 (d, *J* = 8.1 Hz, 2H), 6.97 (d, *J* = 1.5 Hz, 1H), 6.92 (d, *J* = 8.4 Hz, 1H), 6.45 (d, *J* = 2.1 Hz, 1H), 6.31 (d, *J* = 2.1 Hz, 1H), 5.03 (d, *J* = 8.4 Hz, 1H), 4.14-4.11 (m, 1H), 4.02 (t, *J* = 6.3 Hz, 2H), 3.93 (s, 3H), 3.91 (s, 3H), 3.90 (s, 3H), 3.85 (s, 3H), 3.85 (d, *J* = 12.6 Hz, 1H), 3.57 (d, *J* = 11.7 Hz, 1H), 3.34 (t, *J* = 6.9 Hz, 2H), 1.85 (quin, *J* = 7.2 Hz, 2H), 1.74 (quin, *J* = 7.2 Hz, 2H), 1.59-1.49 (m, 2H).

3-O-(N,N-Dimethylamino)butyl-5,7,20-O-trimethyl-2,3-dehydrosilybin (**21**). 86% yield, yellow wax. IR (film) ν_{max}: 3397, 2938, 1625, 1603, 1505, 1460 cm^{-1}; ^{1}H NMR (300 MHz, CDCl$_3$): δ 7.72 (dd, *J* = 8.7 Hz, 2.1 Hz, 1H, H-15), 7.72 (d, *J* = 1.8 Hz, 1H, H-13), 7.04 (d, *J* = 9.3 Hz, 1H, H-16), 7.03 (dd, *J* = 8.1, 1.8 Hz, 1H, H-22), 6.97 (d, *J* = 1.5 Hz, 1H, H-18), 6.91 (d, *J* = 8.1 Hz, 1H, H-21), 6.45 (d, *J* = 2.1 Hz, 1H, H-8), 6.31 (d, *J* = 2.1 Hz, 1H, H-6), 5.04 (d, *J* = 8.1 Hz, 1H, H-11), 4.13 (dt, *J* = 7.8, 3.3 Hz, 1H, H-10), 4.05-401 (m, 2H, 3-O*CH*$_2$), 3.93 (s, 3H, OCH$_3$), 3.91 (s, 3H), OCH$_3$), 3.90 (s, 3H, OCH$_3$), 3.86 (s, 3H, OCH$_3$), 3.85-3.81 (overlapped, 1H, H-23), 3.57 (dd, *J* = 12.3, 3.9 Hz, 1H, H-23), 2.45 (t, *J* = 7.5 Hz, 2H, N*CH*$_2$), 2.29 (s, 6H, 2 × N*CH*$_3$), 1.77-1.65 (m, 4H, 3-OCH$_2$*CH*$_2$*CH*$_2$); ^{13}C NMR (75 MHz, CDCl$_3$): δ 174.1 (C-4), 163.9 (C-7), 161.0 (C-5), 158.8 (C-8a), 152.3 (C-16a), 149.7 (C-19), 149.4 (C-20), 145.2 (C-2), 143.6 (C-12a), 140.4 (C-3), 128.5 (C-17), 124.3 (C-14), 122.4 (C-15), 120.2 (C-22), 117.2 (C-13), 117.0 (C-16), 111.3 (C-21), 110.2 (C-18), 109.4 (C-4a), 95.8 (C-6), 92.3 (C-8), 78.7 (C-10), 76.2 (C-11), 71.8 (3-O*CH*$_2$), 61.5 (C-23), 59.1 (OCH$_3$), 56.4 (OCH$_3$), 56.1 (OCH$_3$), 56.0 (OCH$_3$), 55.7 (N*CH*$_2$), 44.8 (N*CH*$_3$), 27.9 (3-*O*-CH$_2$*CH*$_2$CH$_2$-), 23.5 (3-*O*-CH$_2$CH$_2$*CH*$_2$-); HRMS-ESI *m/z* [M+H]$^+$ calcd for $C_{34}H_{40}NO_{10}$: 622.2652, found: 622.2651.

3-O-(N,N-Dimethylamino)pentyl-5,7,20-O-trimethyl-2,3-dehydrosilybin (**22**). 76% yield, yellow solid, mp. 90-91 °C. IR (film) ν_{max}: 3398, 2919, 1625, 1608, 1509 cm^{-1}; ^1H NMR (300 MHz, $CDCl_3$): δ 7.74-7.67 (overlapped, 2H, H-15 & H-13), 7.03 (d, *J* = 8.7 Hz, 2H, H-16 & H-22), 6.98 (d, *J* = 1.5 Hz, 1H, H-18), 6.91 (d, *J* = 8.1 Hz, 1H, H-21), 6.45 (d, *J* = 2.1 Hz, 1H, H-8), 6.31 (d, *J* = 2.1 Hz, 1H, H-6), 5.06 (d, *J* = 7.8 Hz, 1H, H-11), 4.14 (dt, *J* = 7.8, 3.3 Hz, 1H, H-10), 4.03-3.98 (m, 2H, 3-OCH_2), 3.93 (s, 3H, OCH_3), 3.90 (s, 3H, OCH_3), 3.89 (s, 3H, OCH_3), 3.85 (s, 3H, OCH_3), 3.85-3.81 (overlapped, 1H, H-23), 3.57 (dd, *J* = 12.6, 3.9 Hz, 1H, H-23), 2.42 (t, *J* = 7.5 Hz, 2H, NCH_2), 2.34 (s, 6H, 2 × NCH_3), 1.72 (quin, *J* = 6.9 Hz, 2H, 3-$OCH_2\mathit{CH}_2CH_2CH_2CH_2$), 1.61-1.51 (m, 2H, 3-$OCH_2CH_2CH_2\mathit{CH}_2CH_2$), 1.47-1.37 (m, 2H, 3-$OCH_2CH_2\mathit{CH}_2CH_2CH_2$); ^{13}C NMR (75 MHz, $CDCl_3$): δ 174.1 (C-4), 163.9 (C-7), 161.0 (C-5), 158.8 (C-8a), 152.3 (C-16a), 149.7 (C-19), 149.4 (C-20), 145.1 (C-2), 143.6 (C-12a), 140.5 (C-3), 128.5 (C-17), 124.3 (C-14), 122.4 (C-15), 120.2 (C-22), 117.2 (C-13), 116.9 (C-16), 111.3 (C-21), 110.2 (C-18), 109.4 (C-4a), 95.8 (C-6), 92.3 (C-8), 78.7 (C-10), 76.1 (C-11), 72.0 (3-OCH_2), 61.5 (C-23), 59.1 (OCH_3), 56.4 (OCH_3), 56.0 (OCH_3), 55.9 (OCH_3), 55.7 (NCH_2), 44.5 (NCH_3), 29.9 (3-$OCH_2\mathit{CH}_2CH_2CH_2CH_2$), 26.1 (3-$OCH_2CH_2CH_2\mathit{CH}_2CH_2$), 23.7 (3-$OCH_2CH_2\mathit{CH}_2CH_2CH_2$); HRMS-ESI *m/z* $[M+H]^+$ calcd for $C_{35}H_{42}NO_{10}$: 636.2809, found: 636.2807.

3-O-(N,N-Diethylamino)propyl-5,7,20-O-trimethyl-2,3-dehydrosilybin (**23**). 90% yield, yellow wax. IR (film) ν_{max}: 3375, 2936, 2837, 1624, 1604, 1579, 1517, 1505, 1492, 1462 cm^{-1}; ^1H NMR (300 MHz, $CDCl_3$): δ 7.81-7.56 (overlapped, 1H, H-15), 7.68 (s, 1H, H-13), 7.06 (d, *J* = 9.0 Hz, 1H, H-16), 7.03 (d, *J* = 9.3 Hz, 1H, H-22), 6.97 (s, 1H, H-18), 6.91 (d, *J* = 8.1 Hz, 1H, H-21), 6.47 (s, 1H, H-8), 6.33 (s, 1H, H-6), 5.03 (d, *J* = 7.9 Hz, 1H, H-11), 4.16-4.13 (m, 1H, H-10), 4.04-4.01 (m, 2H, 3-OCH_2), 3.94 (s, 3H, OCH_3), 3.91 (s, 6H, 2 × OCH_3), 3.86 (s, 3H, OCH_3), 3.88-3.84 (overlapped, 1H, H-23), 3.57 (dd, *J* = 12.9, 2.7 Hz, 1H, H-23), 3.07 (t, *J* = 6.9 Hz, 2H, NCH_2), 2.90-2.83 (m, 4H, 2 × NCH_2CH_3), 2.86-2.78 (m, 2H, 3-$OCH_2\mathit{CH}_2$), 1.19 (t, *J* = 6.9 Hz, 6H, 2 × $NCH_2\mathit{CH}_3$); ^{13}C NMR (75 MHz, $CDCl_3$): δ 174.2 (C-4), 164.2 (C-7), 161.1 (C-5), 159.0 (C-8a), 152.9 (C-16a), 149.9 (C-19), 149.6 (C-20), 145.6 C-2), 143.9 (C-12a), 140.2 (C-3), 128.5 (C-17), 124.1 (C-14), 122.4 (C-15), 120.4 (C-22), 117.4 (C-13), 117.3 (C-16), 111.5 (C-21), 110.3 (C-18), 109.4 (C-4a), 96.1 (C-6), 92.5 (C-8), 78.9 C-10), 76.5 (C-11), 70.0 (3-OCH_2), 61.7 (C-23), 56.6 (OCH_3), 56.2 (OCH_3), 56.1 (OCH_3), 55.9 (OCH_3), 49.9 (NCH_2), 46.9 (NCH_2CH_3), 26.4 (3-*O*-$CH_2\mathit{CH}_2CH_2$-), 10.1 ($NCH_2\mathit{CH}_3$); HRMS-ESI *m/z* $[M+H]^+$ calcd for $C_{35}H_{42}NO_{10}$: 636.2809, found: 636.2806.

3-O-(N,N-Diethylamino)butyl-5,7,20-O-trimethyl-2,3-dehydrosilybin (**24**). 69% yield, yellow solid, mp. 64-65 °C. IR (film) ν_{max}: 3397, 2938, 1625, 1606, 1506, 1463 cm^{-1}; ^1H NMR (300 MHz, $CDCl_3$): δ 7.72 (d, *J* = 6.3 Hz, 1H, H-15), 7.71 (s, 1H, H-13), 7.03 (d, *J* = 9.0 Hz, 2H, H-16 & H-22), 6.97 (s, 1H, H-18), 6.91 (d, *J* = 8.1 Hz, 1H, H-21), 6.45 (s, 1H, H-8), 6.31 (s, 1H, H-6), 5.03 (d, *J* = 7.8 Hz, 1H, H-11), 4.15-4.10 (m, 1H, H-10), 4.02-4.98 (m, 2H, 3-OCH_2), 3.95 (s, 3H, OCH_3), 3.93 (s, 6H, 2 × OCH_3), 3.90 (s, 3H, OCH_3), 3.85-3.81 (overlapped, 1H, H-23), 3.56 (dd, *J* = 12.3, 3.3 Hz, 1H, H-23), 2.96 (br.s, 2H, NCH_2), 2.63 (q, *J* = 7.2 Hz, 4H, 2 × NCH_2CH_3), 1.80-1.59 (m, 4H, 3-$OCH_2\mathit{CH}_2\mathit{CH}_2$), 1.06 (t, *J* = 7.2 Hz, 6H, 2 × $NCH_2\mathit{CH}_3$); ^{13}C NMR (75 MHz, $CDCl_3$): δ 174.8 (C-4), 164.5 (C-7), 161.6 (C-5), 159.4 (C-8a), 153.0 (C-16a), 150.3 (C-19), 150.0 (C-20), 145.8 (C-2), 144.2 (C-12a), 141.0 (C-3), 129.1 (C-17), 124.9 (C-14), 123.0 (C-15), 120.8 (C-22), 117.7 (C-13), 117.6 (C-16), 111.9 (C-21), 110.8 (C-18), 110.0 (C-4a), 96.4 (C-6), 92.9 (C-8), 79.3 (C-10), 76.8 (C-11), 72.5 (3-OCH_2), 62.1 (C-23), 57.0 (OCH_3), 56.6 (OCH_3), 56.6 (OCH_3), 56.4 (OCH_3), 52.7 (NCH_2), 47.1 (NCH_2CH_3), 28.7 (3-*O*-$CH_2\mathit{CH}_2CH_2CH_2$-), 22.9 (3-*O*-$CH_2CH_2\mathit{CH}_2CH_2$-), 11.3 ($NCH_2\mathit{CH}_3$); HRMS-ESI *m/z* $[M+H]^+$ calcd for $C_{36}H_{44}NO_{10}$: 650.2966, found 650.2963.

3-O-(N,N-Diethylamino)pentyl-5,7,20-O-trimethyl-2,3-dehydrosilybin (**25**). 33% yield, yellow wax. IR (film) ν_{max}: 3397, 2938, 1624, 1606, 1507, 1459 cm^{-1}; ^1H NMR (300 MHz, $CDCl_3$): δ 7.74 (dd, *J* = 9.3, 2.1 Hz, 1H, H-15), 7.74 (d, *J* = 2.1 Hz, 1H, H-13), 7.05 (d, *J* = 9.3 Hz, 1H, H-16), 7.04 (dd, *J* = 8.4, 2.1 Hz, 1H, H-22), 6.98 (d, *J* = 1.8 Hz, 1H, H-18), 6.92 (d, *J* = 8.1 Hz, 1H, H-21), 6.46 (d, *J* = 2.1 Hz, 1H, H-8), 6.32 (d, *J* = 2.1 Hz, 1H, H-6), 5.07 (d, *J* = 8.1 Hz, 1H, H-11), 4.16 (dt, *J* = 7.2, 2.4 Hz, 1H, H-10), 4.05-3.98 (m, 2H, 3-OCH_2), 3.93 (s, 3H, OCH_3), 3.91 (s, 3H, OCH_3), 3.90 (s, 3H, OCH_3), 3.86 (s, 3H, OCH_3), 3.85 (dd, *J* = 12.6, 2.7 Hz, 1H, H-23), 3.58 (dd, *J* = 12.6, 3.9 Hz, 1H, H-23), 2.75 (q, *J* = 7.2 Hz, 4H, 2 × NCH_2CH_3), 2.63 (t, *J* = 8.4 Hz, 2H, NCH_2), 1.73 (quin, *J* = 6.9 Hz, 2H, 3-*O*-$CH_2\mathit{CH}_2CH_2CH_2CH_2N$-), 1.56-1.51 (m, 2H, 3-*O*-$CH_2CH_2CH_2\mathit{CH}_2CH_2N$-), 1.46-1.41 (m, 2H, 3-*O*-$CH_2CH_2\mathit{CH}_2CH_2CH_2N$-), 1.13 (t, *J* = 7.2 Hz, 6H, 2 ×

NCH$_2$*CH*$_3$); ^{13}C NMR (75 MHz, CDCl$_3$): δ 174.1 (C-4), 163.9 (C-7), 161.0 (C-5), 158.8 (C-8a), 152.5 (C-16a), 149.7 (C-19), 149.4 (C-20), 145.3 (C-2), 143.6 (C-12a), 140.3 (C-3), 128.5 (C-17), 124.2 (C-14), 122.3 (C-15), 120.2 (C-22), 117.2 (C-13), 117.0 (C-16), 111.3 (C-21), 110.2 (C-18), 109.4 (C-4a), 95.8 (C-6), 92.3 (C-8), 78.7 (C-10), 76.1 (C-11), 71.7 (3-O*CH*$_2$), 61.5 (C-23), 56.4 (OCH$_3$), 56.1 (OCH$_3$), 56.0 (OCH$_3$), 55.7 (OCH$_3$), 51.1 (N*CH*$_2$), 46.3 (N*CH*$_2$CH$_3$), 29.5 3-*O*-CH$_2$*CH*$_2$CH$_2$*CH*$_2$CH$_2$N-), 23.7 3-*O*-CH$_2$CH$_2$*CH*$_2$CH$_2$CH$_2$N-), 9.4 (NCH$_2$*CH*$_3$); HRMS-ESI *m/z* [M+H]$^+$ calcd for $C_{37}H_{46}NO_{10}$: 663.3122, found: 664.3116.

3-O-(3-(N,N-Dipropyl)propyl)-5,7,20-O-trimethyl-2,3-dehydrosilybin (**26**). 78% yield, yellow wax. IR (film) ν_{max}: 3338, 3052, 2958, 2934, 2873, 2837, 1624, 1603, 1578, 1517, 1505, 1456 cm^{-1}; ^{1}H NMR (300 MHz, CDCl$_3$): δ 7.70 (d, *J* = 7.7 Hz, 1H, H-15), 7.68 (s, 1H, H-13), 7.05 (d, *J* = 7.9 Hz, 1H, H-16), 7.03 (d, *J* = 6.8 Hz, 1H, H-22), 6.97 (s, 1H, H-18), 6.91 (d, *J* = 8.2 Hz, 1H, H-21), 6.46 (s, 1H, H-8), 6.32 (s, 1H, H-6), 5.03 (d, *J* = 8.0 Hz, 1H, H-11), 4.13 (d, *J* = 7.8 Hz, 1H, H-10), 4.08-4.00 (m, 2H, 3-O*CH*$_2$), 3.93 (s, 3H, OCH$_3$), 3.90 (s, 6H, 2 × OCH$_3$), 3.85 (s, 3H, OCH$_3$), 3.85-3.82 (overlapped, 1H, H-23), 3.57 (dd, *J* = 12.4, 3.3 Hz, 1H, H-23), 2.97-2.85 (m, 2H, 3-*O*-CH$_2$CH$_2$*CH*$_2$N-), 2.59 (t, *J* = 7.4 Hz, 4H, 2 × N*CH*$_2$CH$_2$CH$_3$), 2.05-2.00 (m, 2H, 3-*O*-CH$_2$*CH*$_2$CH$_2$N-), 1.56 (sextet, *J* = 6.8 Hz, 4H, 2 × NCH$_2$*CH*$_2$CH$_3$), 0.86 (t, *J* = 7.2 Hz, 6H, 2 × NCH$_2$CH$_2$*CH*$_3$); ^{13}C NMR (75 MHz, CDCl$_3$): δ 174.2 (C-4), 164.1 (C-7), 161.1 (C-5), 158.9 (C-8a), 152.7 C-16a), 149.9 (C-19), 149.6 (C-20), 145.5 (C-2), 143.8 (C-12a), 140.3 (C-3), 128.5 (C-17), 124.2 (C-14), 122.5 (C-15), 120.3 (C-22), 117.3 (C-13), 117.2 (C-16), 111.5 (C-21), 110.3 (C-18), 109.5 (C-4a), 96.0 (C-6), 92.5 (C-8), 78.9 (C-10), 76.4 (C-11), 70.3 (3-O*CH*$_2$), 61.6 (C-23), 56.5 (OCH$_3$), 56.2 (OCH$_3$), 56.1 (OCH$_3$), 55.9 (OCH$_3$), 55.4 (3-*O*-CH$_2$CH$_2$*CH*$_2$N-), 51.0 (N*CH*$_2$CH$_2$CH$_3$), 26.7 (3-*O*-CH$_2$*CH*$_2$CH$_2$N-), 18.7 (NCH$_2$*CH*$_2$CH$_3$), 11.8 (NCH$_2$CH$_2$*CH*$_3$); HRMS-ESI *m/z* [M+H]$^+$ calcd for $C_{37}H_{46}NO_{10}$: 664.3122, found: 664.3118.

3-O-(N,N-dipropylamino)butyl-5,7,20-O-trimethyl-2,3-dehydrosilybin (**27**). 59% yield, yellow solid, mp. 80-81 ^{o}C. IR (film) ν_{max}: 3415, 2956, 1625, 1607, 1507, 1462, 1433 cm^{-1}; ^{1}H NMR (300 MHz, CDCl$_3$): δ 7.75 (d, *J* = 10.2 Hz, 1H, H-15), 7.73 (s, 1H, H-13), 7.04 (d, *J* = 8.4 Hz, 1H, H-16), 7.03 (d, *J* = 8.4 Hz, 1H, H-22), 6.97 (s, 1H, H-18), 6.92 (d, *J* = 8.1 Hz, 1H, H-21), 6.46 (s, 1H, H-8), 6.32 (s, 1H, H-6), 5.04 (d, *J* = 8.1 Hz, 1H, H-11), 4.15-4.12 (m, 1H, H-10), 4.05-3.99 (m, 2H, 3-O*CH*$_2$), 3.94 (s, 3H, OCH$_3$), 3.91 (s, 6H, 2 × OCH$_3$), 3.86 (s, 3H, OCH$_3$), 3.88-3.82 (overlapped, 1H, H-23), 3.57 (dd, *J* = 12.3, 3.3 Hz, 1H, H-23), 2.55 (t, *J* = 5.7 Hz, 2H, 3-*O*-CH$_2$CH$_2$CH$_2$*CH*$_2$N-), 2.44 (t, *J* = 7.5 Hz, 4H, 2 × N*CH*$_2$CH$_2$CH$_3$), 1.80-1.56 (m, 4H, 2 × NCH$_2$*CH*$_2$CH$_3$), 1.52-1.43 (m, 4H, 3-*O*-CH$_2$*CH*$_2$*CH*$_2$CH$_2$N-), 0.85 (t, *J* = 7.2 Hz, 6H, 2 × NCH$_2$CH$_2$*CH*$_3$); ^{13}C NMR (75 MHz, CDCl$_3$): δ 174.8 (C-4), 164.5 (C-7), 161.6 (C-5), 159.4 (C-8a), 152.9 (C-16a), 150.4 (C-19), 150.0 (C-20), 145.7 (C-2), 144.2 (C-12a), 141.1 (C-3), 129.0 (C-17), 125.0 (C-14), 123.1 (C-15), 120.8 (C-22), 117.7 (C-13), 117.6 (C-16), 111.9 (C-21), 110.7 (C-18), 110.1 (C-4a), 96.4 (C-6), 92.9 (C-8), 79.3 (C-10), 76.9 (C-11), 72.7 (3-O*CH*$_2$), 62.2 (C-23), 57.0 (OCH$_3$), 56.7 (OCH$_3$), 56.6 (OCH$_3$), 56.4 (OCH$_3$), 56.2 (3-*O*-CH$_2$CH$_2$CH$_2$*CH*$_2$N-), 54.1 (N*CH*$_2$CH$_2$CH$_3$), 28.7 (3-*O*-CH$_2$*CH*$_2$CH$_2$CH$_2$N-), 23.3 (3-*O*-CH$_2$CH$_2$*CH*$_2$CH$_2$N-), 20.1 (NCH$_2$*CH*$_2$CH$_3$), 12.5 (NCH$_2$CH$_2$*CH*$_3$); HRMS-ESI *m/z* [M+H]$^+$ calcd for $C_{38}H_{48}NO_{10}$: 678.3279, found: 678.3275.

3-O-(N,N-Dipropylamino)pentyl-5,7,20-O-trimethyl-2,3-dehydrosilybin (**28**). 60% yield, white solid, mp. 94.0-94.5 ^{o}C. IR (film) ν_{max}: 3388, 2936, 2873, 2838, 1624, 1605, 578, 1490, 1462 cm^{-1}; ^{1}H NMR (300 MHz, CDCl$_3$): δ 7.73 (d, *J* = 7.2 Hz, 1H, H-15), 7.72 (s, 1H, H-13), 7.06 (d, *J* = 9.3 Hz, 2H, H-16 & H-22), 6.99 (s, 1H, H-18), 6.93 (d, *J* = 8.4 Hz, 1H, H-21), 6.47 (s, 1H, H-8), 6.33 (s, 1H, H-6), 5.07 (d, *J* = 7.8 Hz, 1H, H-11), 4.16 (d, *J* = 7.8 Hz, 1H, H-10), 4.03 (t, *J* = 6.0 Hz, 2H, 3-O*CH*$_2$), 3.94 (s, 3H, OCH$_3$), 3.91 (s, 6H, 2 × OCH$_3$), 3.87 (s, 3H, OCH$_3$), 3.87-3.83 (overlapped, 1H, H-23), 3.67 (dd, *J* = 12.3, 3.0 Hz, 1H, H-23), 2.68-2.51 (overlapped, 6H, 3-*O*-CH$_2$CH$_2$CH$_2$CH$_2$*CH*$_2$N- & 2 × N*CH*$_2$CH$_2$CH$_3$), 1.75 (quin, *J* = 6.9 Hz, 2H, 3-*O*-CH$_2$*CH*$_2$CH$_2$CH$_2$CH$_2$N-), 1.60-1.50 (m, 6H, 3-*O*-CH$_2$CH$_2$CH$_2$*CH*$_2$CH$_2$N- & 2 × NCH$_2$*CH*$_2$CH$_3$), 1.43 (quin, *J* = 6.9 Hz, 2H 3-*O*-CH$_2$CH$_2$*CH*$_2$CH$_2$CH$_2$N-), 0.90 (t, *J* = 7.5 Hz, 6H, 2 × NCH$_2$CH$_2$*CH*$_3$); ^{13}C NMR (75 MHz, CDCl$_3$): δ 174.1 (C-4), 163.9 (C-7), 161.0 (C-5), 158.8 (C-8a), 152.3 (C-16a), 149.7 (C-19), 149.4 (C-20), 145.1 (C-2), 143.6 (C-12a), 140.4 (C-3), 128.4 (C-17), 124.4 (C-14), 122.4 (C-15), 120.1 (C-22), 117.2 (C-13), 116.9 (C-16), 111.3 (C-21), 110.2 (C-18), 109.5 (C-4a), 95.8 (C-6), 92.3 (C-8), 78.6 (C-10), 76.2 (C-11), 72.0 (3-O*CH*$_2$), 61.5 (C-23), 56.4 (OCH$_3$), 56.1 (OCH$_3$), 56.0 (OCH$_3$), 55.7 (OCH$_3$), 55.2 (3-*O*-CH$_2$CH$_2$CH$_2$CH$_2$*CH*$_2$N-), 53.2 (N*CH*$_2$CH$_2$CH$_3$), 29.7 (3-*O*-

$CH_2\mathit{CH_2}CH_2CH_2CH_2N$-), 23.8 (3-*O*-$CH_2CH_2CH_2\mathit{CH_2}CH_2N$-), 18.8 ($NCH_2\mathit{CH_2}CH_3$), 18.7 (3-*O*-$CH_2CH_2\mathit{CH_2}CH_2CH_2N$-). 11.7 ($NCH_2CH_2\mathit{CH_3}$); HRMS-ESI *m/z* $[M+H]^+$ calcd for $C_{39}H_{50}NO_{10}$: 692.3435, found: 692.3436.

3-O-(3-(N,N-Dibutylamino)propyl)-5,7,20-O-trimethyl-2,3-dehydrosilybin (**29**). 79% yield, yellow wax. IR (film) ν_{max}: 3352, 2957, 2933, 2872, 2838, 1624, 1604, 1578, 1505, 1491, 1457 cm^{-1}; ^1H NMR (300 MHz, $CDCl_3$): δ 7.67 (d, *J* = 7.2 Hz, 1H, H-15), 7.65 (s, 1H, H-13), 7.04 (d, *J* = 8.7 Hz, 1H, H-16), 6.98 (d, *J* = 7.2 Hz, 1H, H-22), 6.96 (s, 1H, H-18), 6.89 (d, *J* = 8.1 Hz, 1H, H-21), 6.45 (s, 1H, H-8), 6.31 (s, 1H, H-6), 5.02 (d, *J* = 8.1 Hz, 1H, H-11), 4.12 (d, *J* = 7.8 Hz, 1H, H-10), 4.05-3.95 (m, 2H, 3-$O\mathit{CH_2}$), 3.92 (s, 3H, OCH_3), 3.89 (s, 3H, OCH_3), 3.88 (s, 3H, OCH_3), 3.84 (s, 3H, OCH_3), 3.84-3.81 (overlapped, 1H, H-23), 3.55 (dd, *J* = 12.6, 3.3 Hz, 1H, H-23), 3.04 (t, *J* = 6.9 Hz, 2H, 3-*O*-$CH_2CH_2\mathit{CH_2}N$-), 2.72 (t, *J* = 7.8 Hz, 4H, 2 × $N\mathit{CH_2}CH_2CH_2CH_3$), 2.13-2.05 (m, 2H, 3-*O*-$CH_2\mathit{CH_2}CH_2N$-), 1.62-1.51 (m, 4H, 2 × $NCH_2\mathit{CH_2}CH_2CH_3$), 1.30 (quin, *J* = 7.2 Hz, 4H, 2 × $NCH_2CH_2\mathit{CH_2}CH_3$), 0.89 (t, *J* = 7.5 Hz, 6H, 2 × $NCH_2CH_2CH_2\mathit{CH_3}$); ^{13}C NMR (75 MHz, $CDCl_3$): δ 174.2 (C-4), 164.2 (C-7), 161.0 (C-5), 158.9 (C-8a), 152.8 (C-16a), 149.9 (C-19), 149.5 (C-20), 145.5 (C-2), 143.9 (C-12a), 140.2 (C-3), 128.5 (C-17), 124.1 (C-14), 122.5 (C-15), 120.3 (C-22), 117.3 (C-13), 117.2 (C-16), 111.4 (C-21), 110.3 (C-18), 109.4 (C-4a), 96.0 (C-6), 92.5 (C-8), 78.9 (C-10), 76.4 (C-11), 70.1 (3-$O\mathit{CH_2}$), 61.6 (C-23), 56.6 (OCH_3), 56.2 (OCH_3), 56.1 (OCH_3), 55.9 (OCH_3), 53.3 (3-*O*-$CH_2CH_2\mathit{CH_2}N$-), 51.1 ($N\mathit{CH_2}CH_2CH_2CH_3$), 27.1 ($NCH_2\mathit{CH_2}CH_2CH_3$), 26.5 (3-*O*-$CH_2\mathit{CH_2}CH_2N$-), 20.6 ($NCH_2CH_2\mathit{CH_2}CH_3$), 14.0 ($NCH_2CH_2CH_2\mathit{CH_3}$); HRMS-ESI *m/z* $[M+H]^+$ calcd for $C_{39}H_{50}NO_{10}$: 692.3435, found 692.3427.

3-O-(N,N-dibutylamino)butyl-5,7,20-O-trimethyl-2,3-dehydrosilybin (**30**). 74% yield, yellow wax. IR (film) ν_{max}: 3397, 2931, 2870, 1624, 1605, 1579, 1506, 1460 cm^{-1}; ^1H NMR (300 MHz, $CDCl_3$): δ 7.75 (dd, *J* = 9.9, 1.8 Hz, 1H, H-15), 7.73 (s, 1H, H-13), 7.05 (d, *J* = 8.4 Hz, 1H, H-16), 7.03 (d, *J* = 8.1 Hz, 1H, H-22), 6.97 (s, 1H, H-18), 6.92 (d, *J* = 8.1 Hz, 1H, H-21), 6.46 (d, *J* = 1.8 Hz, 1H, H-8), 6.32 (d, *J* = 2.1 Hz, 1H, H-6), 5.05 (d, *J* = 8.1 Hz, 1H, H-11), 4.16-4.12 (m, 1H, H-10), 4.02 (t, *J* = 5.7 Hz, 2H, 3-$O\mathit{CH_2}$), 3.94 (s 3H, OCH_3), 3.91 (s, 3H, OCH_3), 3.90 (s, 3H, OCH_3), 3.86 (s, 3H, OCH_3), 3.86-3.81 (overlapped, 1H, H-23), 3.58 (dd, *J* = 12.6, 3.9 Hz, 1H, H-23), 2.59 (t, *J* = 7.5 Hz, 2H, 3-*O*-$CH_2CH_2CH_2\mathit{CH_2}N$-), 2.51 (t, *J* = 7.5 Hz, 4H, 2 × $N\mathit{CH_2}CH_2CH_2CH_3$), 1.78-1.60 (m, 4H, 2 × $NCH_2\mathit{CH_2}CH_2CH_3$), 1.50-1.40 (m, 4H, 3-*O*-$CH_2\mathit{CH_2}\mathit{CH_2}CH_2N$-), 1.33-1.21 (m, 4H, 2 × $NCH_2CH_2\mathit{CH_2}CH_3$), 0.89 (t, *J* = 7.2 Hz, 6H, 2 × $NCH_2CH_2CH_2\mathit{CH_3}$). ^{13}C NMR (75 MHz, $CDCl_3$): δ 174.1 (C-4), 163.9 (C-7), 161.0 (C-5), 158.8 (C-8a), 152.3 (C-16a), 149.7 (C-19), 149.4 (C-20), 145.1 (C-2), 143.6 (C-12a), 140.4 (C-3), 128.4 (C-17), 124.4 (C-14), 122.4 (C-15), 120.2 (C-22), 117.1 (C-13), 117.0 (C-16), 111.3 (C-21), 110.1 (C-18), 109.4 (C-4a), 96.8 (C-6), 92.3 (C-8), 78.6 (C-10), 76.2 (C-11), 72.0 (3-$O\mathit{CH_2}$), 61.5 (C-23), 56.4 (OCH_3), 56.0 (OCH_3), 55.9 (OCH_3), 55.7 (OCH_3), 53.3 (3-*O*-$CH_2CH_2CH_2\mathit{CH_2}N$-), 53.2 ($N\mathit{CH_2}CH_2CH_2CH_3$), 28.1 ($NCH_2\mathit{CH_2}CH_2CH_3$), 22.5 ($NCH_2CH_2\mathit{CH_2}CH_3$), 20.6 ($NCH_2CH_2\mathit{CH_2}CH_3$), 14.0 ($NCH_2CH_2CH_2\mathit{CH_3}$); HRMS-ESI *m/z* $[M+H]^+$ calcd for $C_{40}H_{52}NO_{10}$: 706.3592, found: 706.3591.

3-O-(N,N-Dibutylamino)pentyl-5,7,20-O-trimethyl-2,3-dehydrosilybin (**31**). 85% yield, yellow wax. IR (film) ν_{max}: 3398, 2931, 1625, 1606, 1506, 1462 cm^{-1}; ^1H NMR (300 MHz, $CDCl_3$): δ 7.72 (dd, *J* = 8.1, 2.1 Hz, 1H, H-15), 7.71 (s, 1H, H-13), 7.04 (d, *J* = 8.1 Hz, 2H, H-16 & H-22), 6.98 (d, *J* = 1.8 Hz, 1H, H-18), 6.91 (d, *J* = 8.1 Hz, 1H, H-21), 6.46 (d, *J* = 2.1 Hz, 1H, H-8), 6.31 (d, *J* = 2.1 Hz, 1H, H-6), 5.06 (d, *J* = 8.1 Hz, 1H, H-11), 4.17-4.13 (m, 1H, H-10), 4.02 (t, *J* = 6.3 Hz, 2H, 3-$O\mathit{CH_2}$), 3.93 (s, 3H, OCH_3), 3.91 (s, 3H, OCH_3), 3.90 (s, 3H, OCH_3), 3.86 (s, 3H, OCH_3), 3.86-3.81 (overlapped, 1H, H-11), 3.58 (dd, *J* = 12.3, 3.9 Hz, 1H, H-10), 2.54 (t, *J* = 7.5 Hz, 6H, 3-*O*-$CH_2CH_2CH_2CH_2\mathit{CH_2}N$- & 2 × $N\mathit{CH_2}CH_2CH_2CH_3$), 1.74 (quin, *J* = 7.5 Hz, 2H, 3-*O*-$CH_2\mathit{CH_2}CH_2CH_2CH_2N$-), 1.51-1.25 (m, 12H, 3-*O*-$CH_2CH_2\mathit{CH_2}\mathit{CH_2}CH_2N$- & 2 × $NCH_2\mathit{CH_2}\mathit{CH_2}CH_3$), 0.90 (t, *J* = 7.2 Hz, 6H, 2 × $NCH_2CH_2CH_2\mathit{CH_3}$); ^{13}C NMR (75 MHz, $CDCl_3$): δ 174.8 (C-4), 164.5 (C-7), 161.6 (C-5), 159.4 (C-8a), 153.0 (C-16a), 150.3 (C-19), 150.0 (C-20), 145.8 (C-2), 144.2 (C-12a), 141.1 (C-3), 129.1 (C-17), 124.9 (C-14), 123.0 (C-15), 120.8 (C-22), 117.8 (C-13), 117.6 (C-16), 111.9 (C-21), 110.8 (C-18), 110.0 (C-4a), 96.4 (C-6), 92.9 (C-8), 79.3 (C-10), 76.8 (C-11), 72.7 (3-$O\mathit{CH_2}$), 62.1 (C-23), 57.0 (OCH_3), 56.7 (OCH_3), 56.6 (OCH_3), 56.4 (OCH_3), 53.9 (3-*O*-$CH_2CH_2CH_2CH_2\mathit{CH_2}N$-), 53.8 ($N\mathit{CH_2}CH_2CH_2CH_3$), 30.5 (3-*O*-$CH_2\mathit{CH_2}CH_2CH_2CH_2N$-), 28.3 (3-*O*-$CH_2CH_2CH_2\mathit{CH_2}CH_2N$-), 25.7 (3-*O*-$CH_2CH_2\mathit{CH_2}CH_2CH_2N$-), 24.5 ($NCH_2\mathit{CH_2}CH_2CH_3$), 21.2 ($NCH_2CH_2\mathit{CH_2}CH_3$), 14.6 ($NCH_2CH_2CH_2\mathit{CH_3}$); HRMS-ESI *m/z* $[M+H]^+$ calcd for $C_{41}H_{54}NO_{10}$: 720.3748, found: 720.3741.

3-O-Pyrrolidinopropyl-5,7,20-O-trimethyl-2,3-dehydrosilybin (**32**). 84% yield, yellow oil. IR (film) ν_{max}: 3361, 2935, 2879, 2837, 1668, 1624, 1604, 1578, 1517, 1505, 1459 cm^{-1}; ^1H NMR (300 MHz, $CDCl_3$): δ 7.69 (s, 1H, H-13), 7.68 (dd, *J* = 7.8, 2.1 Hz, 1H, H-15), 7.06-7.01 (overlapped, 2H, H-16 & H-22), 6.96 (d, *J* = 1.5 Hz, 1H, H-18), 6.91 (d, *J* = 8.4 Hz, 1H, H-21), 6.45 (d, *J* = 2.1 Hz, 1H, H-8), 6.32 (d, *J* = 2.1 Hz, 1H, H-6), 5.03 (d, *J* = 8.4 Hz, 1H, H-11), 4.13 (dt, *J* = 7.8, 3.3 Hz, 1H, H-10), 4.07-3.96 (m, 2H, 3-OCH_2), 3.93 (s, 3H, OCH_3), 3.91 (s, 3H, OCH_3), 3.90 (s, 3H, OCH_3), 3.85 (s, 3H，OCH_3), 3.85-3.81 (overlapped, 1H, H-23), 3.56 (dd, *J* = 12.3, 3.6 Hz, 1H, H-23), 3.05 (t, *J* = 7.8 Hz, 2H, 3-*O*-$CH_2CH_2CH_2$N-), 2.95-2.83 (m, 4H, 2 × CH_2 from pyrrolidino), 2.16-2.00 (m, 2H, 3-*O*-$CH_2CH_2CH_2$N-), 1.92 – 1.83 (m, 4H, 2 × CH_2 from pyrrolidino); ^{13}C NMR (75 MHz, $CDCl_3$): δ 174.2 (C-4), 164.2 (C-7), 161.1 (C-5), 159.0 (C-8a), 152.8 (C-16a), 149.9 (C-19), 149.6 (C-20), 145.5 (C-2), 143.8 (C-12a), 140.2 (C-3), 128.5 (C-17), 124.2 (C-14), 122.4 (C-15), 120.4 (C-22), 117.3 (C-13), 117.3 (C-16), 111.5 (C-21), 110.4 (C-18), 109.5 (C-4a), 96.0 (C-6), 92.5 (C-8), 78.9 (C-10), 76.4 (C-11), 70.0 (3-OCH_2), 61.6 (C-23), 56.6 (OCH_3), 56.2 (OCH_3), 56.1 (OCH_3), 55.9 (OCH_3), 53.7 (3-*O*-$CH_2CH_2CH_2$N-), 53.3 (CH_2 from pyrrolidino), 28.2 (3-*O*-$CH_2CH_2CH_2$N-), 23.6 (CH_2 from pyrrolidino); HRMS-ESI *m/z* $[M+H]^+$ calcd for $C_{35}H_{40}NO_{10}$: 634.2652, found: 634.2648.

3-O-Pyrrolidinobutyl-5,7,20-O-trimethyl-2,3-dehydrosilybin (**33**). 69% yield, yellow solid, mp. 109-110 °C. IR (film) ν_{max}: 3398, 2936, 1625, 1606, 1507, 1458, 1433 cm^{-1}; ^1H NMR (300 MHz, $CDCl_3$): δ 7.70 (s, 1H, H-13), 7.69 (d, *J* = 5.4 Hz, 1H, H-15), 7.04 (d, *J* = 9.0 Hz, 1H, H-16)), 7.03 (d, *J* = 8.4 Hz, 1H, H-22), 6.97 (s, 1H, H-18), 6.91 (d, *J* = 8.1 Hz, 1H, H-21), 6.45 (s, 1H, H-8), 6.31 (s, 1H, H-6), 5.05 (d, *J* = 8.1 Hz, 1H, H-11), 4.15-4.12 (m, 1H, H-10), 4.00-3.90 (overlapped, 2H, 3-OCH_2), 3.93 (s, 3H, OCH_3), 3.90 (s, 6H, 2 × OCH_3), 3.85 (s, 3H, OCH_3), 3.85-3.81 (overlapped, 1H, H-23), 3.56 (dd, *J* = 12.6, 3.6 Hz, 1H, H-23), 3.35-3.19 (m, 2H, 3-*O*-$CH_2CH_2CH_2CH_2$N-), 2.88-2.71 (m, 4H, 2 × CH_2 from pyrrolidino), 1.95-1.76 (m, 8H, 3-*O*-$CH_2CH_2CH_2CH_2$N- & 2 × CH_2 from pyrrolidino); ^{13}C NMR (75 MHz, $CDCl_3$): δ 174.8 (C-4), 164.6 (C-7), 161.5 (C-5), 159.4 (C-8a), 153.2 (C-16a), 150.3 (C-19), 150.0 (C-20), 145.9 (C-2), 144.2 (C-12a), 140.9 (C-3), 129.1 (C-17), 124.8 (C-14), 123.0 (C-15), 120.8 (C-22), 117.74 (C-13), 117.70 (C-16), 111.9 (C-21), 110.8 (C-18), 110.0 (C-4a), 96.4 (C-6), 93.0 (C-8), 79.3 (C-10), 76.8 (C-11), 72.2 (3-OCH_2), 62.0 (C-23), 57.0 (OCH_3), 56.7 (OCH_3), 56.6 (OCH_3), 56.4 (OCH_3), 56.3 (3-*O*-$CH_2CH_2CH_2CH_2$N-), 54.2 (2 × CH_2 from pyrrolidino), 28.4 (3-*O*-$CH_2CH_2CH_2CH_2$N-), 24.8 (3-*O*-$CH_2CH_2CH_2CH_2$N-), 24.0 (2 × CH_2 from pyrrolidino); HRMS-ESI *m/z* $[M+H]^+$ calcd for $C_{36}H_{42}NO_{10}$: 648.2809, found: 648.2806.

3-O-Pyrrolidinopentyl-5,7,20-O-trimethyl-2,3-dehydrosilybin (**34**). 77% yield, reddish wax. IR (film) ν_{max}: 3348, 2935, 2874, 2838, 2802, 1667, 1623, 1604, 1578, 1517, 1505, 1460 cm^{-1}; ^1H NMR (300 MHz, $CDCl_3$): δ 7.70 (s, 1H, H-13), 7.69 (dd, *J* = 7.3, 2.0 Hz, 1H, H-15), 7.03 (d, *J* = 9.3 Hz, 2H, H-16 & H-22), 6.98 (d, *J* = 1.3 Hz, 1H, H-18), 6.90 (d, *J* = 8.3 Hz, 1H, H-21), 6.45 (d, *J* = 2.1 Hz, 1H, H-8), 6.31 (d, *J* = 2.1 Hz, 1H H-6), 5.06 (d, *J* =7.9 Hz, 1H, H-11), 4.16-4.13 (m, 1H, H-10), 4.03-3.96 (m, 2H, 3-OCH_2), 3.93 (s, 3H, OCH_3), 3.90 (s, 3H, OCH_3), 3.89 (s, 3H, OCH_3), 3.85 (s, 3H, OCH_3), 3.85-3.81 (overlapped, 1H, H-23), 3.57 (dd, *J* = 12.5, 3.8 Hz, 1H, H-23), 2.74 (t, *J* = 6.6 Hz, 4H, 2 × CH_2 from pyrrolidino), 2.58 (t, *J* = 7.9 Hz, 2H, 3-*O*-$CH_2CH_2CH_2CH_2CH_2$N-), 1.90-1.81 (m, 4H, 2 × CH_2 from pyrrolidino), 1.76-1.55 (m, 4H, 3-*O*-$CH_2CH_2CH_2CH_2CH_2$N-), 1.42 (quin, *J* = 7.8 Hz, 2H, 3-*O*-$CH_2CH_2CH_2CH_2CH_2$N-); ^{13}C NMR (75 MHz, $CDCl_3$): δ 174.1 (C-4), 163.9 (C-7), 161.0 (C-5), 158.8 (C-8a), 152.4 (C-16a), 149.7 (C-19), 149.4 (C-20), 145.2 (C-2), 143.5 (C-12a), 140.5 (C-3), 128.6 (C-17), 124.3 (C-14), 122.4 (C-15), 120.1 (C-22), 117.2 (C-13), 116.9 (C-16), 111.3 (C-21), 110.2 (C-18), 109.4 (C-4a), 95.8 (C-6), 92.3 (C-8), 78.7 (C-10), 76.1 (C-11), 72.1 (3-OCH_2), 61.4 (C-23), 56.4 (OCH_3), 56.0 (OCH_3), 55.9 (OCH_3), 55.8 (OCH_3), 55.7 (3-*O*-$CH_2CH_2CH_2CH_2CH_2$N-), 53.5 (CH_2 from pyrrolidino), 29.8 (3-*O*-$CH_2CH_2CH_2CH_2CH_2$N-), 27.1 (3-*O*-$CH_2CH_2CH_2CH_2CH_2$N-), 23.9 (CH_2 from pyrrolidino), 23.4 (3-*O*-$CH_2CH_2CH_2CH_2CH_2$N-); HRMS-ESI *m/z* $[M+H]^+$ calcd for $C_{37}H_{44}NO_{10}$: 662.2965, found: 662.2970.

3-O-Piperidinopropyl-5,7,20-O-trimethyl-2,3-dehydrosilybin (**35**). 51% yield, yellow wax. IR (film) ν_{max}: 3380, 2934, 2853, 1702, 1626, 1606, 1578, 1518, 1506, 1460 cm^{-1}; ^1H NMR (300 MHz, $CDCl_3$): δ 7.70 (s, 1H, H-13), 7.69 (d, *J* = 7.2 Hz, 1H, H-15), 7.06 (d, *J* = 9.0 Hz, 1H, H-16), 7.03 (d, *J* = 8.0 Hz, 1H, H-22), 6.97 (s, 1H, H-18), 6.92 (d, *J* = 8.2 Hz, 1H, H-21), 6.46 (d, *J* = 1.6 Hz, 1H, H-8), 6.32 (d, *J* = 1.7 Hz, 1H, H-6), 5.03 (d, *J* = 8.1 Hz, 1H, H-11), 4.14 (dd, *J* = 11.2, 3.2 Hz, 1H, H-10), 4.08-3.97 (m, 2H, 3-OCH_2), 3.94 (s, 3H, OCH_3), 3.92 (s, 3H, OCH_3), 3.91 (s, 3H, OCH_3), 3.86 (s, 3H, OCH_3), 3.86-3.82 (overlapped, 1H, H-23), 3.57 (dd, *J* =

12.5, 3.7 Hz, 1H, H-23), 2.94-2.83 (m, 2H, 3-*O*-$CH_2CH_2CH_2$N-), 2.76-2.63 (m, 4H, 2 × NCH_2 from piperidino), 2.12-2.08 (m, 2H, 3-*O*-$CH_2CH_2CH_2$N-), 1.79-1.76 (m, 4H, 2 × CH_2 from piperidino), 1.54-1.43 (m, 2H, CH_2 from piperidino); ^{13}C NMR (75 MHz, $CDCl_3$): δ 174.1 (C-4), 164.0 (C-7), 160.9 (C-5), 158.8 (C-8a), 152.6 (C-16a), 149.8 (C-19), 149.4 (C-20), 145.3 (C-2), 143.7 (C-12a), 140.1 (C-3), 128.3 (C-17), 124.0 (C-14), 122.3 (C-15), 120.2 (C-22), 117.2 (C-13), 117.2 (C-16), 111.3 (C-21), 110.2 (C-18), 109.3 (C-4a), 95.9 (C-6), 92.4 (C-8), 78.7 (C-10), 76.3 (C-11), 70.1 (3-OCH_2), 61.5 (C-23), 56.4 (OCH_3), 56.1 (OCH_3), 56.0 (OCH_3), 55.77 (OCH_3), 55.76 (3-*O*-$CH_2CH_2CH_2$N-), 53.9 (NCH_2 from piperidino), 26.2 (3-*O*-$CH_2CH_2CH_2$N-), 24.3 (CH_2 from piperidino), 23.4 (CH_2 from piperidino); HRMS-ESI *m/z* [M+H]$^+$ calcd for $C_{36}H_{42}NO_{10}$: 648.2809, found: 648.2807.

3-O-Piperidinobutyl-5,7,20-O-trimethyl-2,3-dehydrosilybin (**36**). 78% yield, yellow solid, mp. 91-92 ^{o}C. IR (film) ν_{max}: 3392, 2937, 2837, 1625, 1606, 1506, 1458 cm^{-1}; ^{1}H NMR (300 MHz, $CDCl_3$): δ 7.70-7.65 (overlapped, 2H, H-13 & H-15), 7.02 (d, *J* = 9.0 Hz, 2H, H-16 & H-22), 6.97 (s, 1H, H-18), 6.90 (d, *J* = 8.1 Hz, 1H, H-21), 6.44 (s, 1H, H-8), 6.30 (s, 1H, H-6), 5.03 (d, *J* = 7.8 Hz, 1H, H-11), 4.15-4.10 (m, 1H, H-10), 3.97 (t, *J* = 5.7 Hz, 2H, 3-OCH_2), 3.92 (s, 3H, OCH_3), 3.90 (s, 3H OCH_3), 3.89 (s, 3H, OCH_3), 3.84 (s, 3H, OCH_3), 3.84-3.81 (overlapped, 1H, H-23), 3.56 (dd, *J* = 12.3, 3.6 Hz, 1H, H-23), 2.70-2.42 (overlapped, 6H, 3-*O*-$CH_2CH_2CH_2CH_2$N- & 2 × NCH_2 from piperidino), 1.74-1.64 (m, 8H, 3-*O*-$CH_2CH_2CH_2CH_2$N- & 2 × CH_2 from piperidino), 1.52-1.36 (m, 2H, CH_2 from piperidino); ^{13}C NMR (75 MHz, $CDCl_3$): δ 174.8 (C-4), 164.6 (C-7), 161.5 (C-5), 159.4 (C-8a), 153.0 (C-16a), 150.3 (C-19), 150.0 (C-20), 145.9 (C-2), 144.2 (C-12a), 141.0 (C-3), 129.1 (C-17), 124.8 (C-14), 123.0 (C-15), 120.8 (C-22), 117.71 (C-13), 117.65 (C-16), 111.9 (C-21), 110.8 (C-18), 110.0 (C-4a), 96.4 (C-6), 93.0 (C-8), 79.3 (C-10), 76.8 (C-11), 72.4 (3-OCH_2), 62.0 (C-23), 59.1 (OCH_3), 57.0 (OCH_3), 56.7 (OCH_3), 56.6 (OCH_3), 56.4 (3-*O*-$CH_2CH_2CH_2CH_2$N-), 54.5 (NCH_2 from piperidino), 28.6 (3-*O*-$CH_2CH_2CH_2CH_2$N-), 25.4 (3-*O*-$CH_2CH_2CH_2CH_2$N-), 24.3 (CH_2 from piperidino), 22.9 (CH_2 from piperidino); HRMS-ESI *m/z* [M+H]$^+$ calcd for $C_{37}H_{44}NO_{10}$: 662.2966, found: 662.2959.

3.10.18. *3-O-Piperidinopentyl-5,7,20-O-trimethyl-2,3-dehydrosilybin* (**37**). 91% yield, white solid, mp. 89.5-90.0 ^{o}C. IR (film) ν_{max}: 3404, 2935, 2858, 1624, 1605, 1578, 1538, 1517, 1490, 1462 cm^{-1}; ^{1}H NMR (300 MHz, $CDCl_3$): δ 7.71 (s, 1H, H-13), 7.70 (d, *J* = 6.5 Hz, 1H, H-15), 7.03 (d, *J* = 8. 2 Hz, 2H, H-16 & H-22), 6.98 (s, 1H, H-18), 6.92 (d, *J* = 8.1 Hz, 1H, H-21), 6.45 (s, 1H, H-8), 6.31 (s, 1H, H-6), 5.06 (d, *J* = 7.9 Hz, 1H, H-11), 4.16-4.13 (m, 1H, H-10), 4.03-3.97 (m, 2H, 3-OCH_2), 3.93 (s, 3H, OCH_3), 3.90 (s, 6H, 2 × OCH_3), 3.85 (s, 3H, OCH_3), 3.85-3.82 (overlapped, 1H, H-23), 3.58 (dd, *J* = 11.8, 2.1 Hz, 1H, H-23), 2.59-2.45 (m, 4H, 2 × NCH_2 from piperidino), 2.41 (t, *J* = 7.1 Hz, 2H, 3-*O*-$CH_2CH_2CH_2CH_2CH_2$N-), 1.76-1.62 (m, 6H, 3-*O*-$CH_2CH_2CH_2CH_2CH_2$N- & 2 × CH_2 from piperidino), 1.60-1.52 (m, 2H, 3-*O*-$CH_2CH_2CH_2CH_2CH_2$N-), 1.51-1.37 (m, 4H, CH_2 from piperidino & 3-*O*-$CH_2CH_2CH_2CH_2CH_2$N-); ^{13}C NMR (75 MHz, $CDCl_3$): δ 174.3 (C-4), 164.0 (C-7), 161.1 (C-5), 158.9 (C-8a), 152.5 (C-16a), 149.9 (C-19), 149.6 (C-20), 145.3 (C-2), 143.7 (C-12a), 140.6 (C-3), 128.7 (C-17), 124.5 (C-14), 122.6 (C-15), 120.3 (C-22), 117.4 (C-13), 117.1 (C-16), 111.5 (C-21), 110.3 (C-18), 109.6 (C-4a), 95.9 (C-6), 92.5 (C-8), 78.8 (C-10), 76.3 (C-11), 72.3 (3-OCH_2), 61.6 (C-23), 58.9 (OCH_3), 56.5 (OCH_3), 56.2 (OCH_3), 56.1 (OCH_3), 55.9 (3-*O*-$CH_2CH_2CH_2CH_2CH_2$N-), 54.1 (NCH_2 from piperidino), 30.0 (3-*O*-$CH_2CH_2CH_2CH_2CH_2$N-), 25.6 (3-*O*-$CH_2CH_2CH_2CH_2CH_2$N-), 25.0 (3-*O*-$CH_2CH_2CH_2CH_2CH_2$N-), 24.2 (CH_2 from piperidino), 23.9 (CH_2 from piperidino); HRMS-ESI *m/z* [M+H]$^+$ calcd for $C_{38}H_{46}NO_{10}$: 676.3122, found: 676.3117.

3-O-Morpholinopropyl-5,7,20-O-trimethyl-2,3-dehydrosilybin (**38**). 54% yield, yellow wax. IR (film) ν_{max}: 3379, 2937, 2838, 1625, 1606, 1518, 1506, 1493, 1462 cm^{-1}; ^{1}H NMR (300 MHz, $CDCl_3$): δ 7.69 (s, 1H, H-13), 7.68 (d, *J* = 8.6 Hz, 1H, H-15), 7.07 (d, *J* = 8.9 Hz, 1H, H-16), 7.04 (d, *J* = 10.8 Hz, 1H, H-22), 6.97 (s, 1H, H-18), 6.92 (d, *J* = 8.2 Hz, 1H, H-21), 6.48 (s, 1H, H-8), 6.34 (s, 1H, H-6), 5.03 (d, *J* = 8.3 Hz, 1H, H-11), 4.16-4.14 (m, 1H, H-10), 4.02-3.90 (overlapped, 2H, 3-OCH_2), 3.94 (s, 3H, OCH_3), 3.91 (s, 6H, 2 × OCH_3), 3.87 (s, 3H, OCH_3), 3.94-3.87 (overlapped, 4H, 2 × OCH_2 from morpholino), 3.87-3.83 (overlapped, 1H, H-23), 3.58 (dd, *J* = 12.2, 2.9 Hz, 1H, H-23), 3.24-3.10 (m, 2H, 3-*O*-$CH_2CH_2CH_2$N-), 3.03-2.83 (m, 4H, 2 × NCH_2 from morpholino), 2.29-2.13 (m, 2H, 3-*O*-$CH_2CH_2CH_2$N-); ^{13}C NMR (75 MHz, $CDCl_3$): δ 174.2 (C-4), 164.3 (C-7), 161.1 (C-5), 159.0 (C-8a), 153.0 (C-16a), 150.0 (C-19), 149.6 (C-20), 145.6 (C-2), 143.9 (C-12a), 140.1 (C-3), 128.4 (C-17), 124.0 (C-14), 122.4 (C-15), 120.4 (C-22), 117.4 (C-13), 117.3 (C-16), 111.5 (C-21), 110.4 (C-18), 109.4 (C-4a), 96.1 (C-6), 92.6 (C-

8), 78.9 (C-10), 76.5 (C-11), 69.6 (3-O*CH*$_2$), 65.0 (O*CH*$_2$ from morpholino), 61.7 (C-23), 56.6 (OCH_3), 56.2 (OCH_3), 56.2 (OCH_3), 56.1 (OCH_3), 56.0 (3-*O*-CH_2CH_2*CH*$_2$N-), 52.9 (N*CH*$_2$ from morpholino), 25.6 (3-*O*-CH_2*CH*$_2$$CH_2$N-); HRMS-ESI *m/z* $[M+H]^+$ calcd for $C_{35}H_{40}NO_{11}$: 650.2601, found: 650.2602.

3-O-Morpholinobutyl-5,7,20-O-trimethyl-2,3-dehydrosilybin (**39**). 90% yield, yellow wax. IR (film) ν_{max}: 3415, 2938, 1624, 1605, 1579, 1491 cm^{-1}; ^{1}H NMR (300 MHz, $CDCl_3$): δ 7.73 (d, *J* = 6.9 Hz, 1H, H-15), 7.72 (s, 1H, H-13), 7.02 (d, *J* = 9.0 Hz, 2H, H-16 & H-22), 6.96 (s, 1H, H-18), 6.91 (d, *J* = 8.4 Hz, 1H, H-21), 6.45 (d, *J* = 1.5 Hz, 1H, H-8), 6.31 (s, 1H, H-6), 5.03 (d, *J* = 8.1 Hz, 1H, H-11), 4.13-4.10 (m, 1H, H-10), 4.07-4.01 (m, 2H, 3-O*CH*$_2$), 3.93 (s, 3H, OCH_3), 3.91 (s, 3H, OCH_3), 3.90 (s, 3H, OCH_3), 3.85 (s, 3H, OCH_3), 3.85-3.82 (overlapped, 1H, H-23), 3.68 (t, *J* = 4.5 Hz, 4H, 2 × O*CH*$_2$ from morpholino), 3.56 (dd, *J* = 12.3, 3.6 Hz, H-23), 2.42-2.35 (overlapped, 6H, 3-*O*-$CH_2CH_2CH_2$*CH*$_2$N- & 2 × N*CH*$_2$ from morpholino), 1.77-1.62 (m, 4H, 3-*O*-CH_2*CH*$_2$*CH*$_2$$CH_2$N-); ^{13}C NMR (75 MHz, $CDCl_3$): δ 174.8 (C-4), 164.5 (C-7), 161.6 (C-5), 159.4 (C-8a), 152.8 (C-16a), 150.4 (C-19), 150.0 (C-20), 145.8 (C-2), 144.2 (C-12a), 141.1 (C-3), 129.0 (C-17), 125.0 (C-14), 123.0 (C-15), 120.8 (C-22), 117.8 (C-13), 117.6 (C-16), 111.9 (C-21), 110.7 (C-18), 110.0 (C-4a), 96.4 (C-6), 92.9 (C-8), 79.3 (C-10), 76.9 (C-11), 72.6 (3-O*CH*$_2$), 67.4 (O*CH*$_2$ from morpholino), 62.2 (C-23), 59.3 (OCH_3), 57.0 (OCH_3), 56.7 (OCH_3), 56.6 (OCH_3), 56.4 (3-*O*-$CH_2CH_2CH_2$*CH*$_2$N-), 54.1 (N*CH*$_2$ from morpholino), 28.7 (3-*O*-CH_2*CH*$_2$$CH_2CH_2$N-), 23.3 (3-*O*-$CH_2CH_2$*CH*$_2$$CH_2$N-); HRMS-ESI *m/z* $[M+H]^+$ calcd for $C_{36}H_{42}NO_{11}$: 664.2758, found: 664.2757.

3-O-Morpholinopentyl-5,7,20-O-trimethyl-2,3-dehydrosilybin (**40**). 77% yield, light yellow wax. IR (film) ν_{max}: 3397, 2936, 1625, 1603, 1505, 1456, 1428 cm^{-1}; ^{1}H NMR (300 MHz, $CDCl_3$): δ 7.72-7.69 (overlapped, 2H, H-15 & H-13), 7.02 (dd, *J* = 8.4, 1.8 Hz, 1H, H-16), 7.01 (d, *J* = 9.3 Hz, 1H, H-22), 6.97 (s, 1H, H-18), 6.90 (d, *J* = 8.1 Hz, 1H, H-21), 6.44 (d, *J* = 2.1 Hz, 1H, H-8), 6.30 (d, *J* = 2.1 Hz, 1H, H-6), 5.03 (d, *J* = 8.1 Hz, 1H, H-11), 4.12 (dt, *J* = 8.1, 4.8 Hz, 1H, H-10), 4.01-3.98 (m, 2H, 3-O*CH*$_2$), 3.92 (s, 3H, OCH_3), 3.90 (s, 3H, OCH_3), 3.89 (s, 3H, OCH_3), 3.84 (s, 3H, OCH_3), 3.84-3.80 (overlapped, 1H, H-23), 3.72 (t, *J* = 4.5 Hz, 4H, 2 × O*CH*$_2$ from morpholino), 3.56 (dd, *J* = 12.6, 3.9 Hz, 1H, H-23), 2.47 (br,s, 4H, 2 × N*CH*$_2$ from morpholino), 2.35 (t, *J* = 7.2 Hz, 2H, 3-*O*-$CH_2CH_2CH_2CH_2$*CH*$_2$N-), 1.73 (quin, *J* = 6.9 Hz, 2H, 3-*O*-CH_2*CH*$_2$$CH_2CH_2CH_2$N-), 1.53-1.23 (m, 4H, 3-*O*-CH_2CH_2*CH*$_2$*CH*$_2$$CH_2$N-). ^{13}C NMR (75 MHz, $CDCl_3$): δ 174.1 (C-4), 163.9 (C-7), 160.9 (C-5), 158.7 (C-8a), 152.2 (C-16a), 149.7 (C-19), 149.4 (C-20), 145.1 (C-2), 143.6 (C-12a), 140.5 (C-3), 128.4 (C-17), 124.4 (C-14), 122.4 (C-15), 120.2 (C-22), 117.1 (C-13), 116.9 (C-16), 111.3 (C-21), 110.2 (C-18), 109.4 (C-4a), 95.7 (C-6), 92.3 (C-8), 78.7 (C-10), 76.2 (C-11), 72.0 (3-O*CH*$_2$), 66.5 (O*CH*$_2$ from morpholino), 61.5 (C-23), 58.9 (OCH_3), 56.3 (OCH_3), 56.0 (OCH_3), 55.9 (OCH_3), 55.7 (3-*O*-$CH_2CH_2CH_2CH_2$*CH*$_2$N-), 53.4 (N*CH*$_2$ from morpholino), 29.9 (3-*O*-CH_2*CH*$_2$$CH_2CH_2CH_2$N-), 25.7 (3-*O*-$CH_2CH_2CH_2$*CH*$_2$$CH_2$N-), 23.8 (3-*O*-$CH_2CH_2$*CH*$_2$$CH_2CH_2$N-); HRMS-ESI *m/z* $[M+H]^+$ calcd for $C_{37}H_{44}NO_{11}$: 678.2914, found: 678.2915.

3-O-(Piperazin-1-yl)propyl-5,7,20-O-trimethyl-2,3-dehydrosilybin (**41**). 74% yield, white solid, mp. 158-159 °C. IR (film) ν_{max}: 3347, 3207, 2937, 2836, 1623, 1604, 1578, 1517, 1490, 1458 cm^{-1}; ^{1}H NMR (300 MHz, $CDCl_3$): δ 7.67 (s, 1H, H-13), 7.66 (d, *J* = 8.1 Hz, 1H, H-15), 7.02 (d, *J* = 8.1 Hz, 2H, H-16 & H-22), 6.97 (s, 1H, H-18), 6.90 (d, *J* = 8.1 Hz, 1H, H-21), 6.44 (s, 1H, H-8), 6.31 (s, 1H H-6), 5.73 (br.s, 1H, NH), 5.04 (d, *J* = 7.8 Hz, 1H, H-11), 4.13 (d, *J* = 7.5 Hz, 1H, H-10), 4.04 (t, *J* = 5.4 Hz, 2H, 3-O*CH*$_2$), 3.92 (s, 3H, OCH_3), 3.90 (s, 3H, OCH_3), 3.89 (s, 3H, OCH_3), 3.85 (s, 3H, OCH_3), 3.85-3.82 (overlapped, 1H, H-23), 3.55 (dd, *J* = 12.3, 3.0 Hz, 1H H-23), 2.97 (br.s, 4H, 2 × N*CH*$_2$ from piperazine), 2.49 (br.s., 6H, 3-*O*-CH_2CH_2*CH*$_2$N- & 2 × N*CH*$_2$ from piperazine), 1.90-1.79 (m, 2H, 3-*O*-CH_2*CH*$_2$$CH_2$N-); ^{13}C NMR (75 MHz, $CDCl_3$): δ 174.1 (C-4), 163.9 (C-7), 160.9 (C-5), 158.8 (C-8a), 152.5 (C-16a), 149.7 (C-19), 149.3 (C-20), 145.3 (C-2), 143.5 (C-12a), 140.3 (C-3), 128.5 (C-17), 124.1 (C-14), 122.5 (C-15), 120.2 (C-22), 117.3 (C-13), 116.9 (C-16), 111.3 (C-21), 110.2 (C-18), 109.4 (C-4a), 95.8 (C-6), 92.3 (C-8), 78.7 (C-10), 76.1 (C-11), 70.4 (3-O*CH*$_2$), 61.2 (C-23), 56.4 (OCH_3), 56.0 (OCH_3), 55.9 (OCH_3), 55.8 (OCH_3), 55.3 (3-*O*-CH_2CH_2*CH*$_2$N-), 51.7 (N*CH*$_2$ from piperazine), 44.2 (N*CH*$_2$ from piperazine), 27.2 (3-*O*-CH_2*CH*$_2$$CH_2$N-); HRMS-ESI *m/z* $[M+H]^+$ calcd for $C_{35}H_{41}N_2O_{10}$: 649.2761, found: 649.2764.

3-O-(Piperazin-1-yl)butyl-5,7,20-O-trimethyl-2,3-dehydrosilybin (**42**). 68% yield, yellow wax. IR (film) ν_{max}: 3379, 2934, 1625, 1604, 1505, 1456 cm^{-1}; ^{1}H NMR (300 MHz, $CDCl_3$): δ 7.68 (d, *J* = 7.8 Hz, 1H, H-15), 7.66 (s, 1H, H-13), 7.03 (d, *J* = 7.8 Hz, 2H, H-16 & H-22), 6.99 (s, 1H, H-18), 6.91 (d, *J* = 8.1 Hz, 1H, H-21),

6.45 (s, 1H, H-8), 6.32 (s, 1H, H-6), 5.06 (d, *J* = 7.8 Hz, 1H, H-11), 4.17-4.09 (m, 1H, H-10), 4.09-3.99 (m, 2H, 3-O*CH*$_2$), 3.93 (s, 3H, OCH$_3$), 3.90 (s, 3H, OCH$_3$), 3.89 (s, 3H, OCH$_3$), 3.86 (s, 3H, OCH$_3$), 3.85-3.79 (overlapped, 1H, H-23), 3.56 (d, *J* = 11.1 Hz, 1H, H-23), 3.11-2.99 (m, 2H, 3-*O*-CH$_2$CH$_2$CH$_2$*CH*$_2$N-), 2.61-2.43 (m, 4H, 2 × N*CH*$_2$ from piperazine), 2.39-2.26 (m, 4H, 2 × N*CH*$_2$ from piperazine), 1.74-1.47 (m, 4H, 3-*O*-CH$_2$*CH*$_2$*CH*$_2$CH$_2$N-); ^{13}C NMR (75 MHz, CDCl$_3$): δ 174.8 (C-4), 164.5 (C-7), 161.6 (C-5), 159.4 (C-8a), 153.2 (C-16a), 150.3 (C-19), 150.0 (C-20), 145.8 (C-2), 144.2 (C-12a), 141.0 (C-3), 129.1 (C-17), 124.9 (C-14), 123.3 (C-15), 120.8 (C-22), 117.9 C-13), 117.5 (C-16), 111.9 (C-21), 110.8 (C-18), 110.0 (C-4a), 96.4 (C-6), 92.9 (C-8), 79.4 (C-10), 76.7 (C-11), 72.7 (3-O*CH*$_2$), 61.8 (C-23), 58.6 (OCH$_3$), 57.0 (OCH$_3$), 56.7 (OCH$_3$), 56.6 (OCH$_3$), 56.4 (3-*O*-CH$_2$CH$_2$CH$_2$*CH*$_2$N-),, 52.0 (N*CH*$_2$ from piperazine), 44.9 (N*CH*$_2$ from piperazine), 28.4 (3-*O*-CH$_2$*CH*$_2$CH$_2$CH$_2$N-), 23.7 (3-*O*-CH$_2$CH$_2$*CH*$_2$CH$_2$N-); HRMS-ESI *m*/*z* [M+H]$^+$ calcd for $C_{36}H_{43}N_2O_{10}$: 663.2918, found: 663.2912.

3-O-(Piperazin-1-yl)pentyl-5,7,20-O-trimethyl-2,3-dehydrosilybin (**43**). 98% yield, light yellow wax. IR (film) ν_{max}: 3365, 3003, 2937, 2861, 2836, 1667, 1623, 1604, 1578, 1517, 1491, 1459 cm^{-1}; ^{1}H NMR (300 MHz, CDCl$_3$): δ 7.72 (d, *J* = 8.1 Hz, 1H, H-15), 7.67 (s, 1H, H-13), 7.04 (d, *J* = 8.1 Hz, 2H, H-16 & H-22), 6.98 (s, 1H, H-18), 6.91 (d, *J* = 8.1 Hz, 1H, H-21), 6.46 (s, 1H, H-8), 6.32 (s, 1H, H-6), 5.05 (d, *J* = 7.8 Hz, 1H, H-11), 4.12 (d, *J* = 7.2 Hz, 1H, H-10), 4.02 (t, *J* = 4.5 Hz, 2H, 3-O*CH*$_2$), 3.93 (s, 6H, 2 × OCH$_3$), 3.91 (s, 6H, 2 × OCH$_3$), 3.91-3.83 (overlapped, 1H, H-23), 3.56 (d, *J* = 12.3 Hz, 1H, H-23), 3.28-2.98 (m, 4H, 2 × N*CH*$_2$ from piperazine), 2.62-2.50 (m, 4H, 2 × N*CH*$_2$ from piperazine), 2.41-2.25 (m, 2H, 3-*O*-CH$_2$CH$_2$CH$_2$CH$_2$*CH*$_2$N-), 1.78-1.60 (m, 2H, 3-*O*-CH$_2$*CH*$_2$CH$_2$CH$_2$CH$_2$N-), 1.50-1.33 (m, 4H, 3-*O*-CH$_2$CH$_2$*CH*$_2$*CH*$_2$CH$_2$N-); ^{13}C NMR (75 MHz, CDCl$_3$): δ 174.3 (C-4), 164.1 (C-7), 161.1 (C-5), 159.0 (C-8a), 152.7 (C-16a), 149.9 (C-19), 149.6 (C-20), 145.3 (C-2), 143.8 (C-12a), 140.6 (C-3), 128.7 (C-17), 124.5 (C-14), 122.8 (C-15), 120.4 (C-22), 117.3 (C-13), 117.0 (C-16), 111.5 (C-21), 110.4 (C-18), 109.6 (C-4a), 95.9 (C-6), 92.5 (C-8), 79.0 (C-10), 76.4 (C-11), 72.2 (3-O*CH*$_2$), 61.3 (C-23), 58.5 (OCH$_3$), 56.5 (OCH$_3$), 56.2 (OCH$_3$), 56.1 (OCH$_3$), 55.9 (3-*O*-CH$_2$CH$_2$CH$_2$CH$_2$*CH*$_2$N-), 51.0 (N*CH*$_2$ from piperazine), 43.9 (N*CH*$_2$ from piperazine), 30.0 (3-*O*-CH$_2$*CH*$_2$CH$_2$CH$_2$CH$_2$N-), 26.2 (3-*O*-CH$_2$CH$_2$CH$_2$*CH*$_2$CH$_2$N-), 24.1 (3-*O*-CH$_2$CH$_2$*CH*$_2$CH$_2$CH$_2$N-); HRMS-ESI *m*/*z* [M+H]$^+$ calcd for $C_{37}H_{45}N_2O_{10}$: 677.3074, found: 677.3064.

3-O-(4-Methylpiperazin-1-yl)propyl-5,7,20-O-trimethyl-2,3-dehydrosilybin (**44**). 75% yield, yellow wax. IR (film) ν_{max}: 3398, 3002, 2937, 2879, 2836, 2806, 1625, 1606, 1578, 1517, 1506, 1491, 1460 cm^{-1}; ^{1}H NMR (300 MHz, CDCl$_3$): δ 7.70 (s, 1H, H-13), 7.68 (dd, *J* = 8.7, 2.1 Hz, 1H, H-15), 7.03 (d, *J* = 8.4 Hz, 2H, H-16 & H-22), 6.97 (d, *J* = 1.5 Hz, 1H, H-18), 6.91 (d, *J* = 8.2 Hz, 1H, H-21), 6.45 (d, *J* = 2.1 Hz, 1H, H-8), 6.31 (d, *J* = 2.1 Hz, 1H, H-6), 5.04 (d, *J* = 8.1 Hz, 1H, H-11), 4.17-4.12 (m, 1H, H-10), 4.10-4.04 (m, 2H, 3-O*CH*$_2$), 3.93 (s, 3H, OCH$_3$), 3.91 (s, 3H, OCH$_3$), 3.90 (s, 3H, OCH$_3$), 3.86 (s, 3H, OCH$_3$), 3.85-3.82 (overlapped, 1H, H-23), 3.58 (dd, *J* = 12.3, 3.9 Hz, 1H, H-23), 2.62-2.47 (overlapped, 10H, 4 × N*CH*$_2$ from 4-methylpiperazine and 3-*O*-CH$_2$CH$_2$*CH*$_2$N-), 2.32 (s, 3H, *N*CH$_3$), 1.93 (quin, *J* = 6.6 Hz, 2H, 3-*O*-CH$_2$*CH*$_2$CH$_2$N-); ^{13}C NMR (75 MHz, CDCl$_3$): δ 174.0 (C-4), 163.9 (C-7), 161.0 (C-5), 158.8 (C-8a), 152.3 (C-16a), 149.7 (C-19), 149.4 (C-20), 145.2 (C-2), 143.6 (C-12a), 140.3 (C-3), 128.5 (C-17), 124.2 (C-14), 122.4 (C-15), 120.2 (C-22), 117.3 (C-13), 117.0 (C-16), 111.3 (C-21), 110.2 (C-18), 109.4 (C-4a), 95.8 (C-6), 92.3 (C-8), 78.7 (C-10), 76.2 (C-11), 70.4 (3-O*CH*$_2$), 61.4 (C-23), 56.4 (OCH$_3$), 56.1 (OCH$_3$), 56.0 (OCH$_3$), 55.7 (OCH$_3$), 55.2 (3-*O*-CH$_2$CH$_2$*CH*$_2$N-), 54.3 (N*CH*$_2$ from 4-methylpiperazine), 52.3 (N*CH*$_2$ from 4-methylpiperazine), 45.4 (*N*CH$_3$), 27.2 (3-*O*-CH$_2$*CH*$_2$CH$_2$N-); HRMS-ESI *m*/*z* [M+H]$^+$ calcd for $C_{36}H_{43}N_2O_{10}$ 663.2918, found: 663.2914.

3-O-(4-Methylpiperazin-1-yl)pentyl-5,7,20-O-trimethyl-2,3-dehydrosilybin (**45**). 93% yield, light yellow wax. IR (film) ν_{max}: 3384, 2941, 2838, 1624, 1606, 1579, 1517, 1506, 1491, 1463 cm^{-1}; ^{1}H NMR (300 MHz, CDCl$_3$): δ 7.72 (dd, *J* = 7.7, 2.1 Hz, 1H, H-15), 7.70 (s, 1H, H-13), 7.05-7.01 (overlapped, 2H, H-16 & H-22), 6.98 (d, *J* = 1.8 Hz, 1H, H-18), 6.91 (d, *J* = 8.3 Hz, 1H, H-21), 6.46 (d, *J* = 2.2 Hz, 1H, H-8), 6.31 (d, *J* = 2.2 Hz, 1H, H-6), 5.06 (d, *J* = 8.1 Hz, 1H, H-11), 4.13 (dt, *J* = 7.9, 3.1 Hz, 1H, H-10), 4.02 (t, *J* = 6.4 Hz, 2H, 3-O*CH*$_2$), 3.93 (s, 3H, OCH$_3$), 3.91 (s, 3H, OCH$_3$), 3.90 (s, 3H, OCH$_3$), 3.86 (s, 3H, OCH$_3$), 3.85 (dd, *J* = 12.5, 2.6 Hz, 1H, H-23), 3.56 (dd, *J* = 12.5, 3.8 Hz, 1H, H-23), 2.89-2.70 (overlapped, 4H, 2 × N*CH*$_2$ from 4-methylpiperazine), 2.63-2.45 (m, 6H, 2 × N*CH*$_2$ from 4-methylpiperazine and 3-*O*-CH$_2$CH$_2$CH$_2$CH$_2$*CH*$_2$N-), 2.32 (s, 3H, *N*CH$_3$), 1.71 (quin, *J* = 6.3 Hz, 2H, 3-*O*-CH$_2$*CH*$_2$CH$_2$CH$_2$CH$_2$N-), 1.51-1.35 (m, 4H, 3-*O*-CH$_2$CH$_2$*CH*$_2$*CH*$_2$CH$_2$N-); ^{13}C NMR (75 MHz, CDCl$_3$): δ 174.1 (C-4), 163.9 (C-7), 161.0 (C-5), 158.8 (C-8a), 152.3 (C-16a), 149.7 (C-19), 149.4 (C-20), 145.1 (C-2),

143.6 (C-12a), 140.5 (C-3), 128.5 (C-17), 124.4 (C-14), 122.5 (C-15), 120.2 (C-22), 117.2 (C-13), 116.9 (C-16), 111.3 (C-21), 110.2 (C-18), 109.5 (C-4a), 95.8 (C-6), 92.3 (C-8), 78.7 (C-10), 76.1 (C-11), 72.2 (3-O*C*H_2), 61.4 (C-23), 58.4 (OCH_3), 56.4 (OCH_3), 56.1 (OCH_3), 56.0 (OCH_3), 55.7 (3-*O*-$CH_2CH_2CH_2CH_2$*C*H_2N-), 54.5 (N*C*H_2 from 4-methylpiperazine), 52.5 (N*C*H_2 from 4-methylpiperazine), 45.6 (*N*CH_3), 29.9 (3-*O*-$CH_2CH_2CH_2$*C*H_2CH_2N-), 26.1 (3-*O*-CH_2*C*$H_2CH_2CH_2CH_2$N-), 23.9 (3-*O*-CH_2CH_2*C*$H_2CH_2CH_2$N-); HRMS-ESI m/z $[M+H]^+$ calcd for $C_{38}H_{47}N_2O_{10}$: 691.3231, found 691.3233.

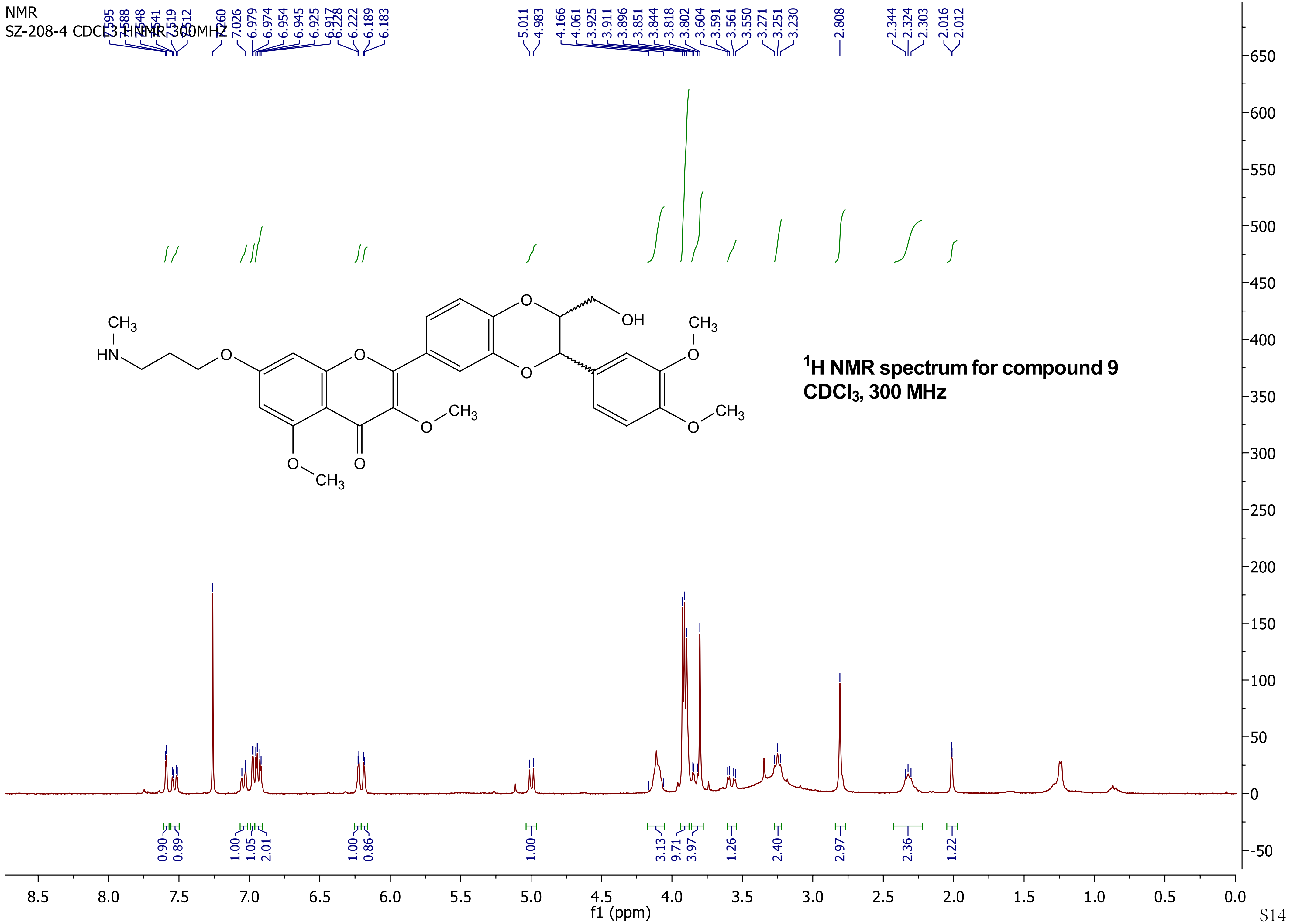
NMR
SZ-208-4 CDCl3 HNMR 300MHZ
1H NMR spectrum for compound 9
CDCl3, 300 MHz
f1 (ppm)

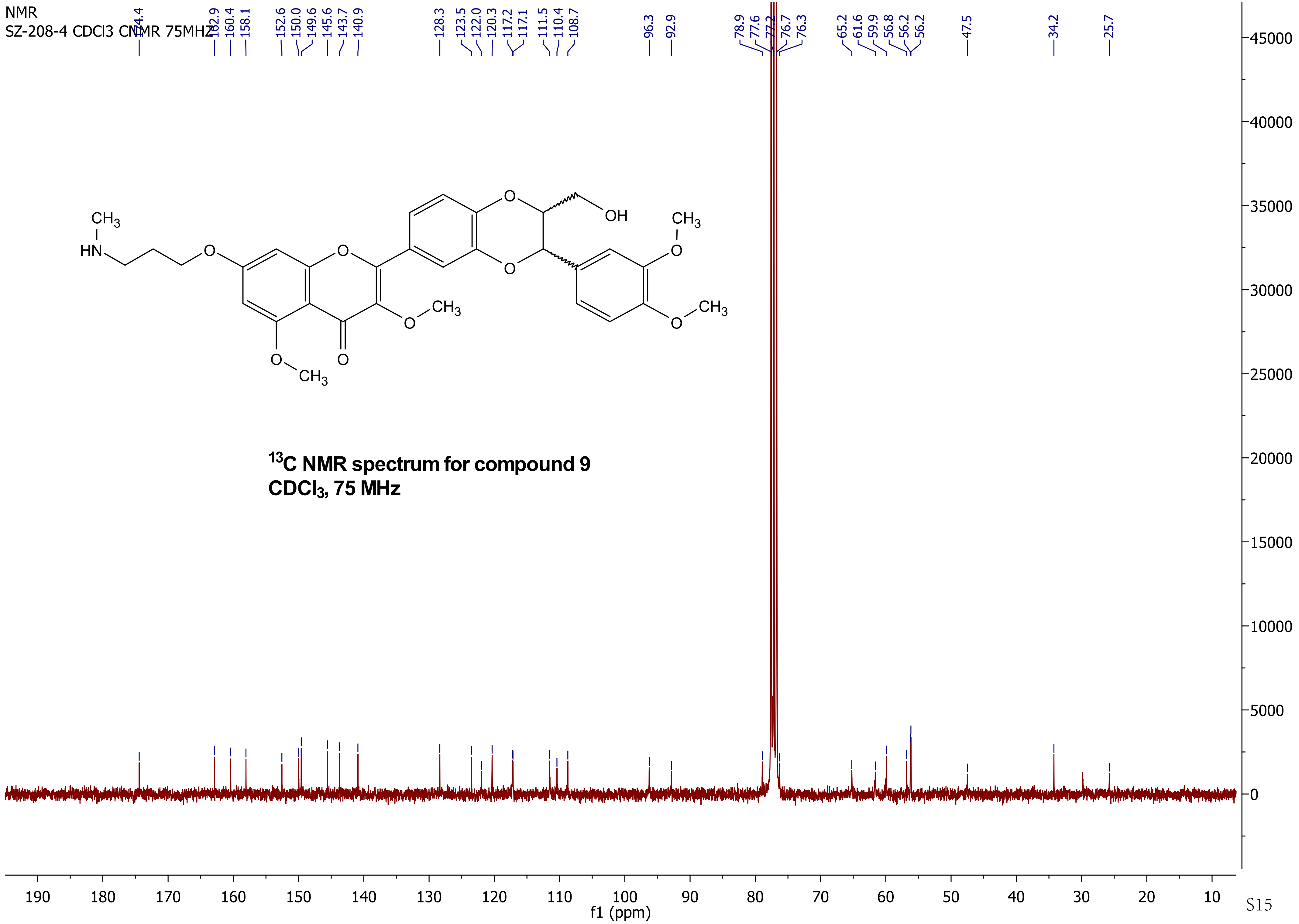

^{13}C NMR spectrum for compound 9
$CDCl_3$, 75 MHz

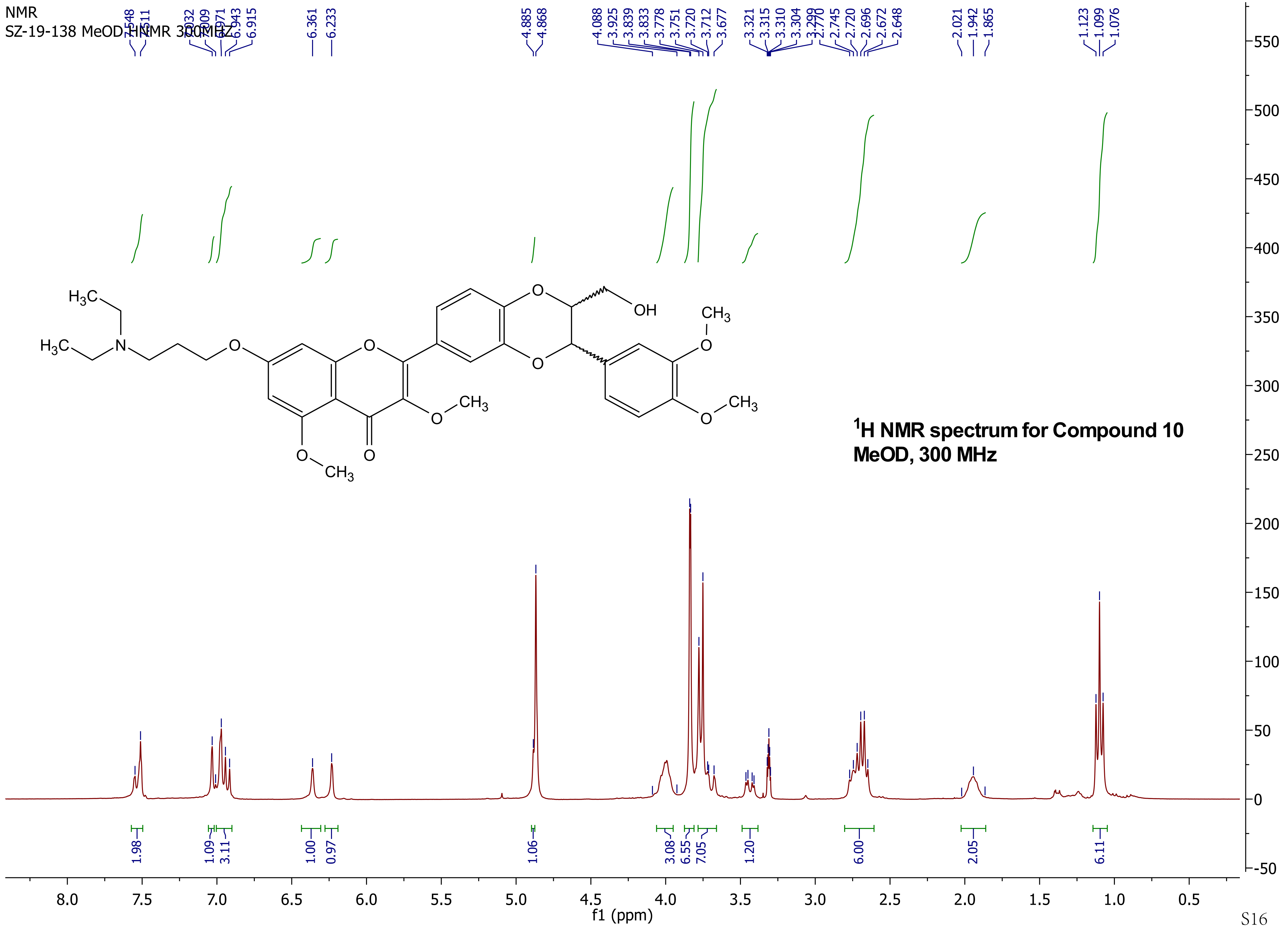

NMR
SZ-19-138 MeOD HNMR 300MHZ
7.548
7.511
7.032
7.009
6.971
6.943
6.915
6.361
6.233
4.885
4.868
4.088
3.925
3.839
3.833
3.778
3.751
3.720
3.712
3.677
3.321
3.315
3.310
3.304
3.299
2.770
2.745
2.720
2.696
2.672
2.648
2.021
1.942
1.865
1.123
1.099
1.076
H_3C
N
O
OH
CH_3
^{1}H NMR spectrum for Compound 10
MeOD, 300 MHz
1.98
1.09
3.11
1.00
0.97
1.06
3.08
6.55
7.05
1.20
6.00
2.05
6.11
f1 (ppm)

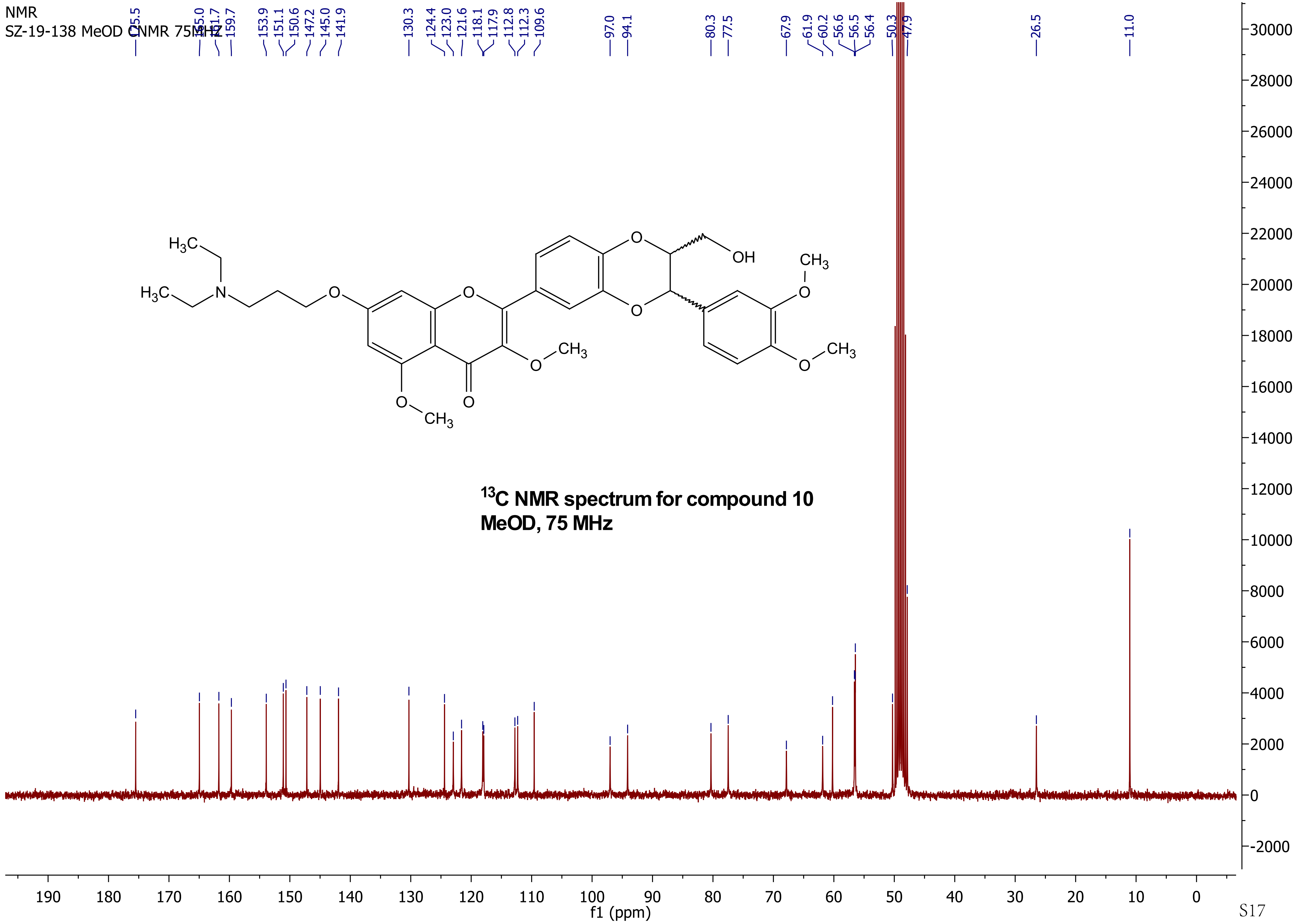
NMR
SZ-19-138 MeOD CNMR 75MHz
175.5 165.0 161.7 159.7 153.9 151.1 150.6 147.2 145.0 141.9 130.3 124.4 123.0 121.6 118.1 117.9 112.8 112.3 109.6 97.0 94.1 80.3 77.5 67.9 61.9 60.2 56.6 56.5 56.4 50.3 47.9 26.5 11.0
^{13}C NMR spectrum for compound 10
MeOD, 75 MHz
f1 (ppm)

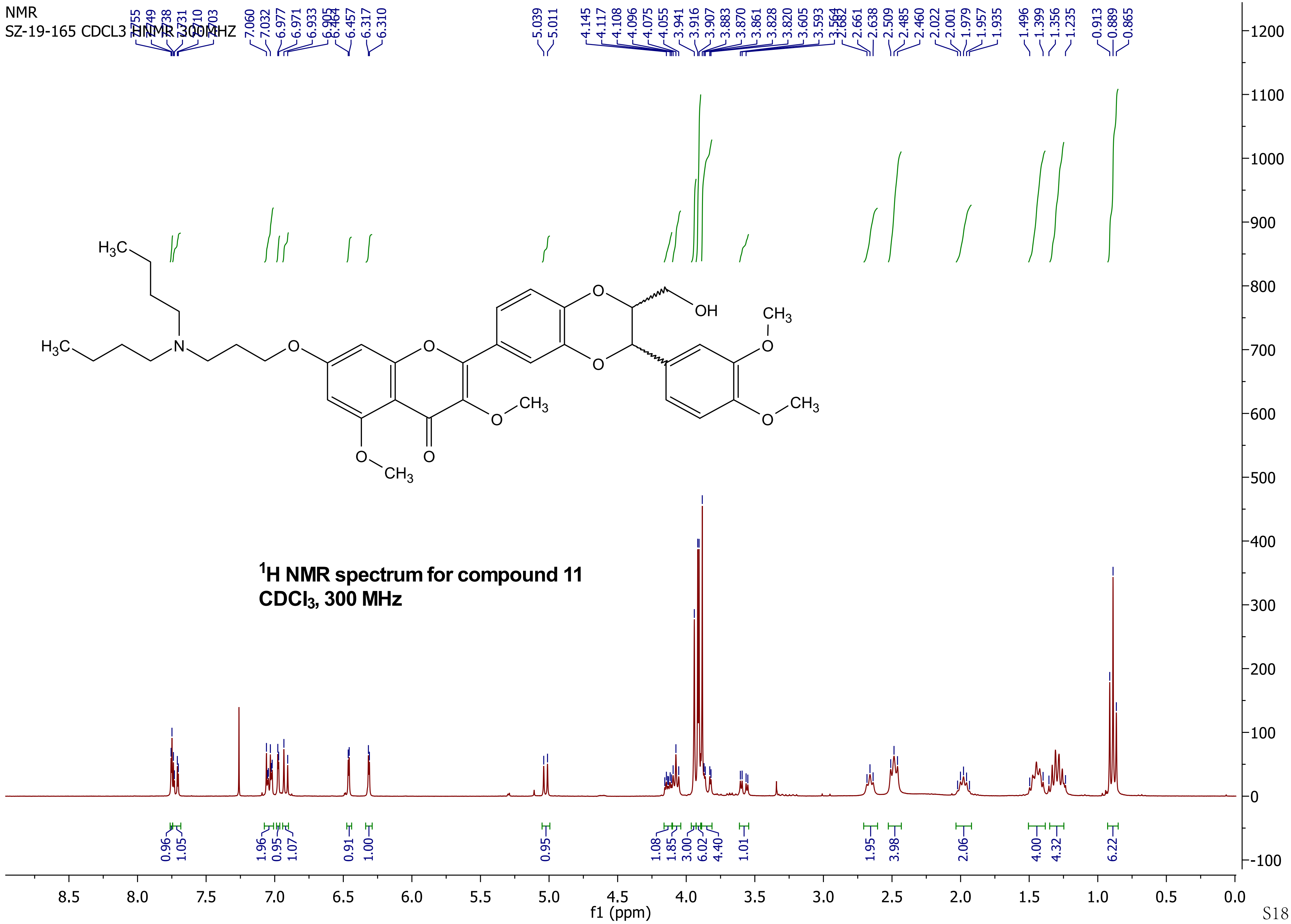

NMR
SZ-19-165 CDCL3 HNMR 300MHZ
1H NMR spectrum for compound 11
CDCl3, 300 MHz
f1 (ppm)

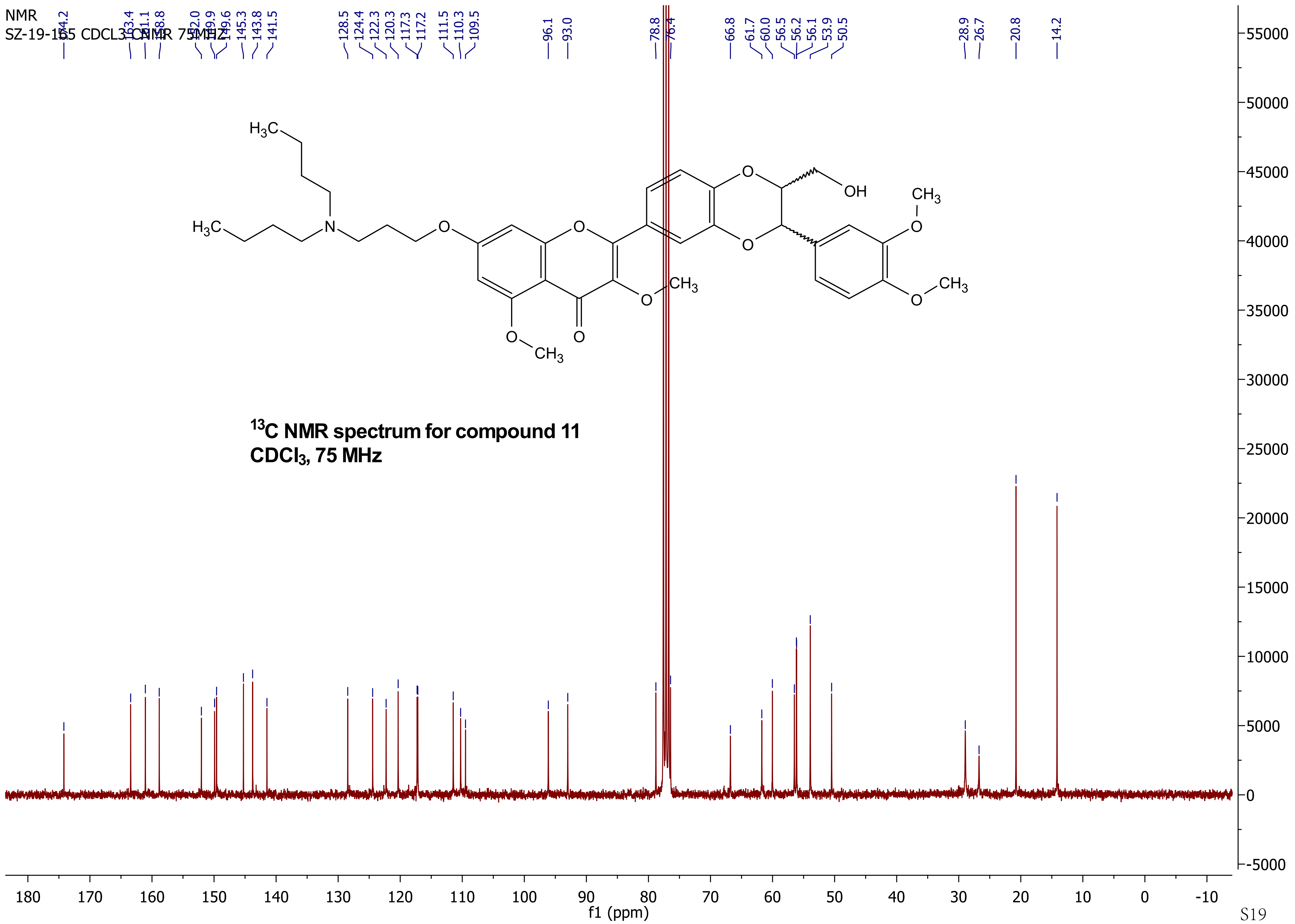
NMR
SZ-19-155 CDCL3 CNMR 75MHZ
174.2
163.4
161.1
158.8
152.0
149.9
149.6
145.3
143.8
141.5
128.5
124.4
122.3
120.3
117.3
117.2
111.5
110.3
109.5
96.1
93.0
78.8
76.4
66.8
61.7
60.0
56.5
56.2
56.1
53.9
50.5
28.9
26.7
20.8
14.2
H3C
H3C
N
O
O
O
O
O
O
O
OH
CH3
O
CH3
O
CH3
O
CH3
^{13}C NMR spectrum for compound 11
CDCl3, 75 MHz
f1 (ppm)

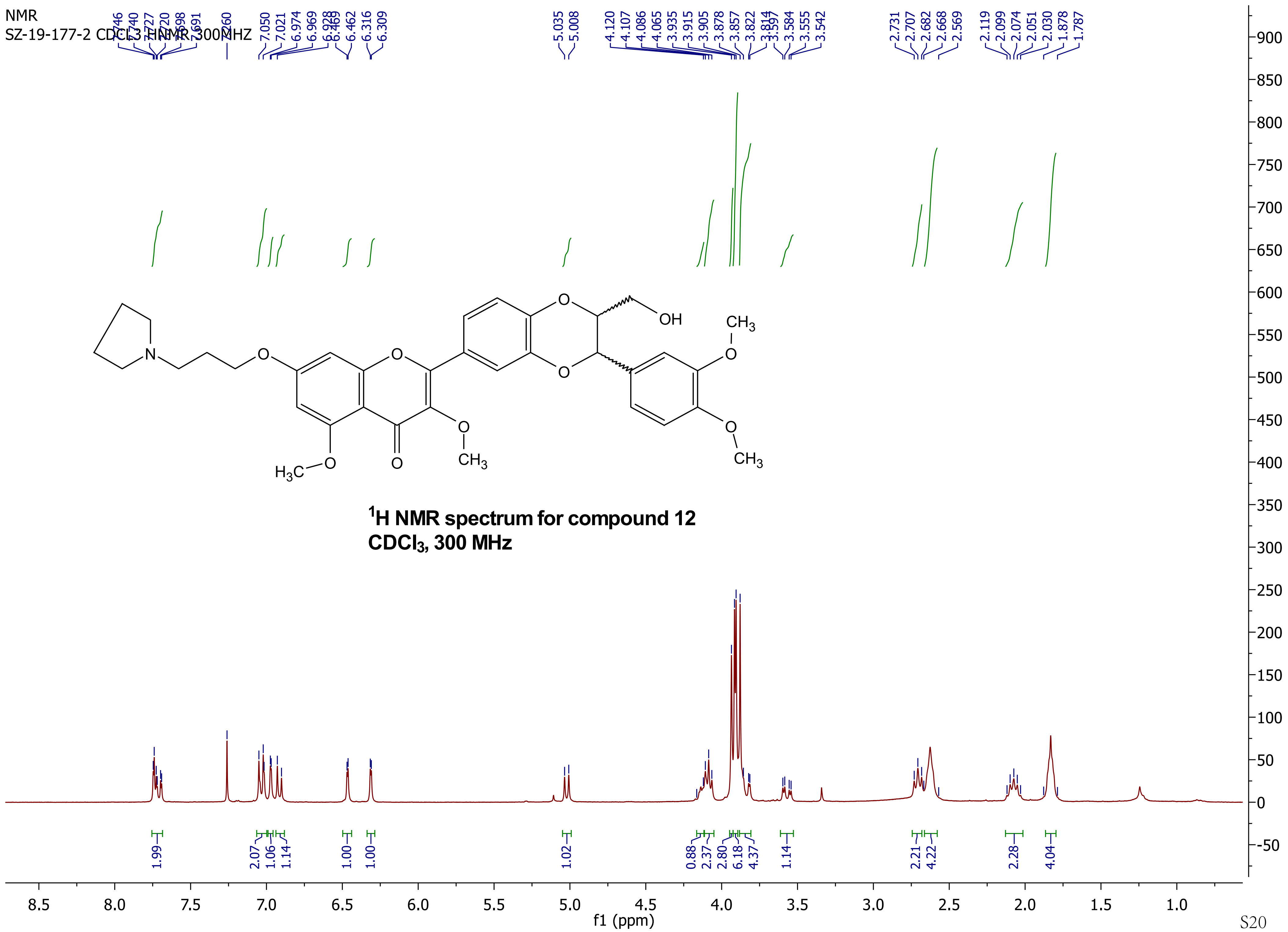

^{1}H NMR spectrum for compound 12
$CDCl_3$, 300 MHz

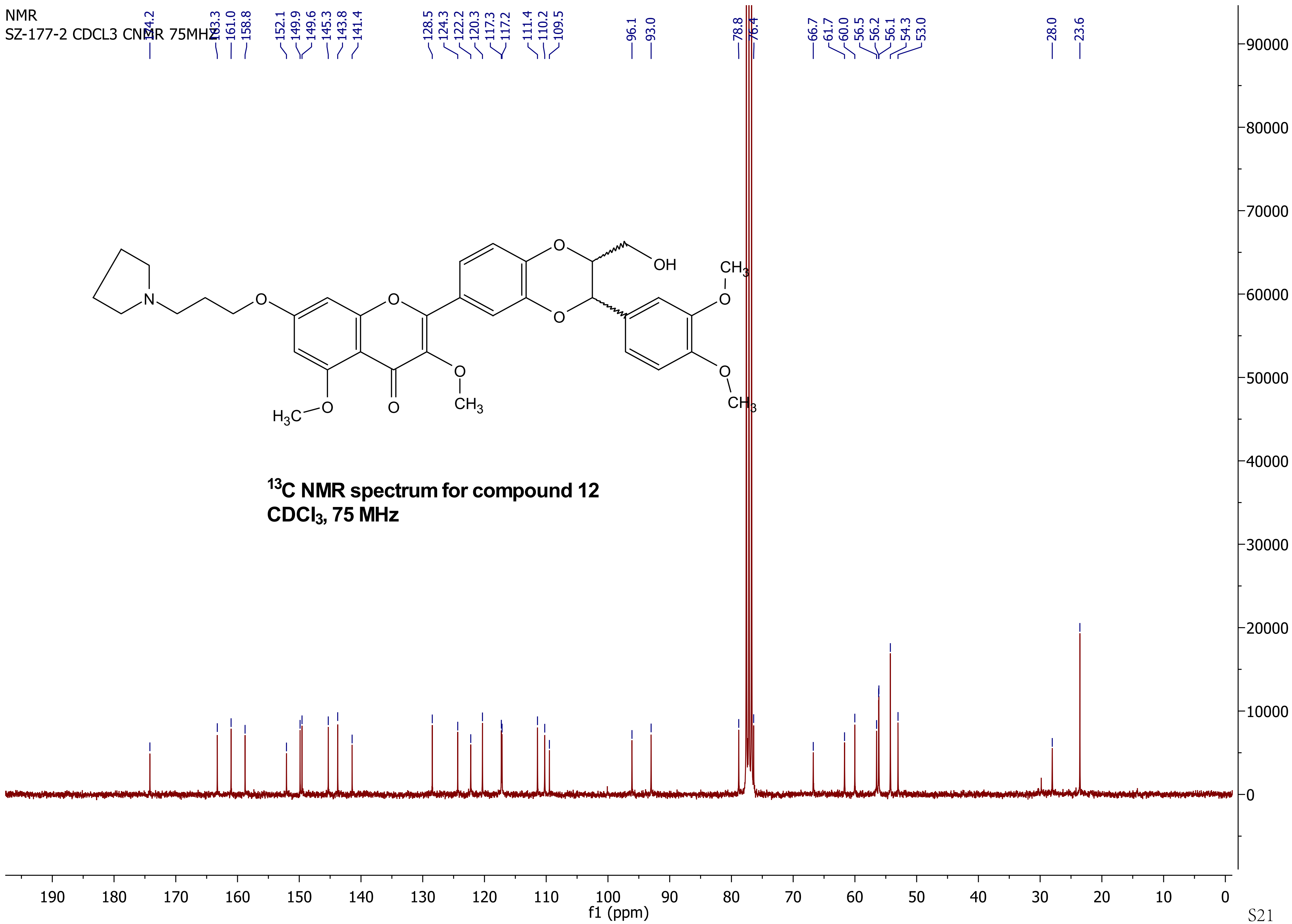

^{13}C NMR spectrum for compound 12
$CDCl_3$, 75 MHz

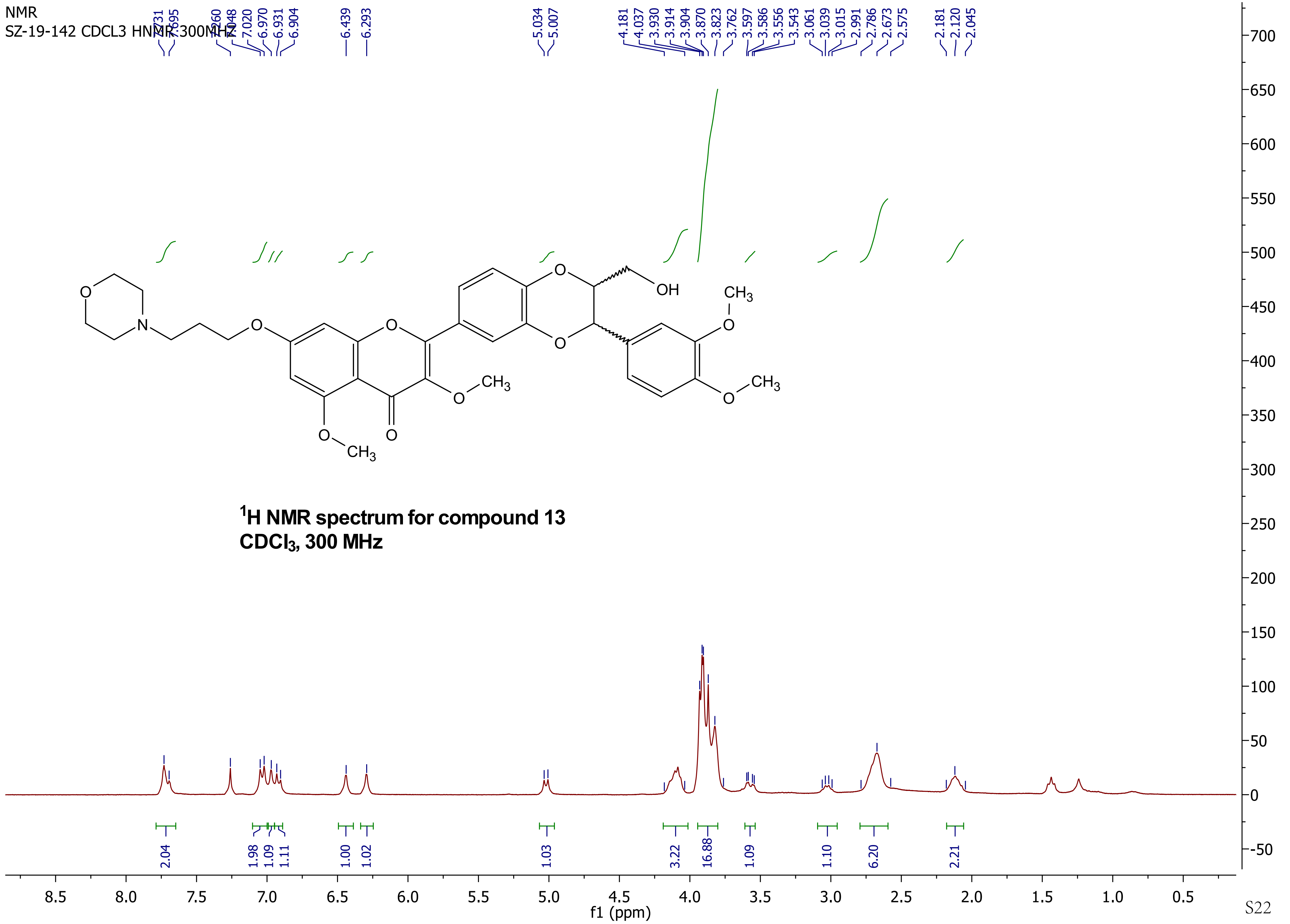

^{1}H NMR spectrum for compound 13
$CDCl_3$, 300 MHz

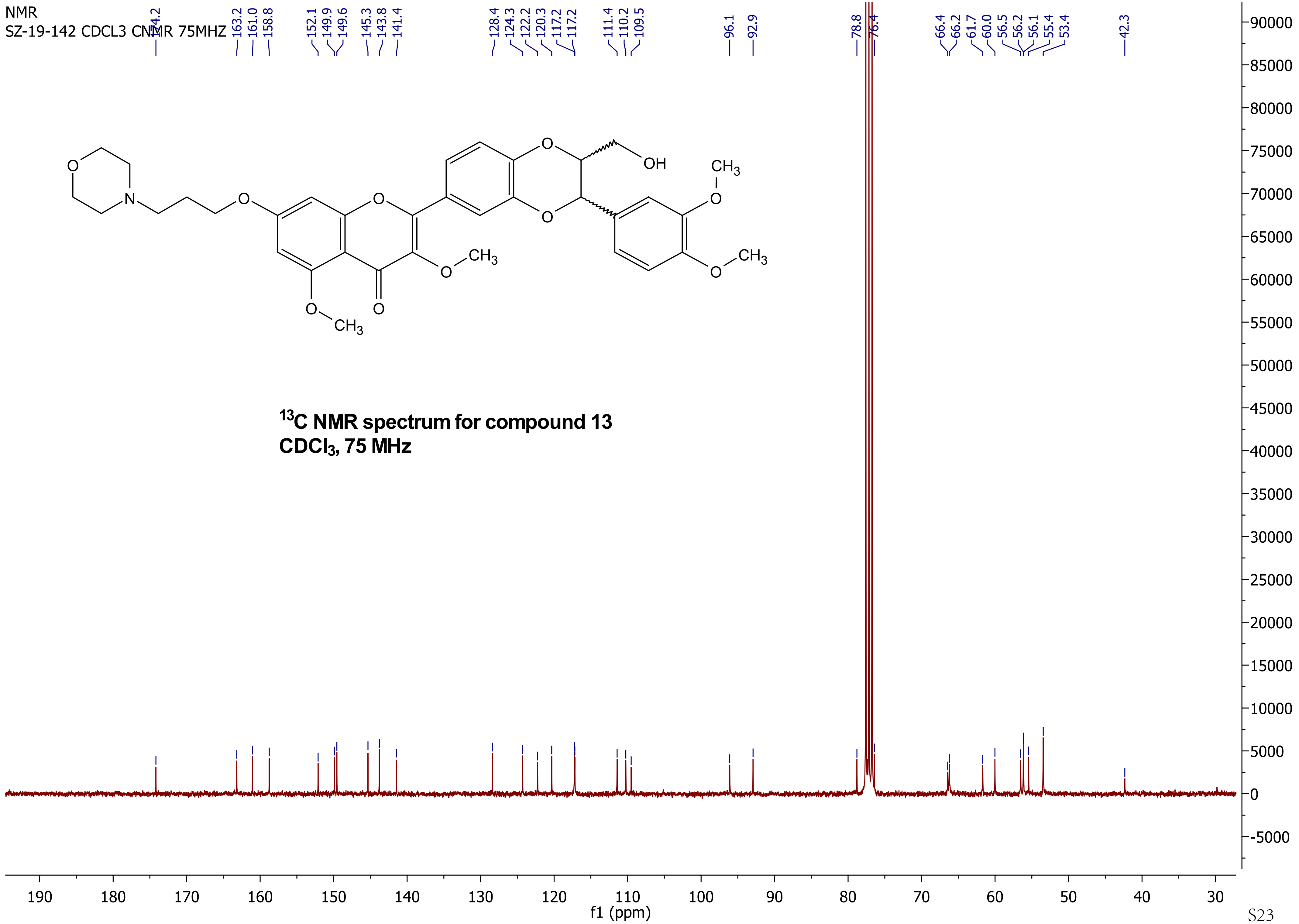
NMR
SZ-19-142 CDCL3 CNMR 75MHZ
174.2
163.2
161.0
158.8
152.1
149.9
149.6
145.3
143.8
141.4
128.4
124.3
122.2
120.3
117.2
117.2
111.4
110.2
109.5
96.1
92.9
78.8
76.4
66.4
66.2
61.7
60.0
56.5
56.2
56.1
55.4
53.4
42.3
O
N
O
O
O
O
O
OH
CH3
O
CH3
O
O
CH3
O
CH3
13C NMR spectrum for compound 13
CDCl3, 75 MHz
f1 (ppm)

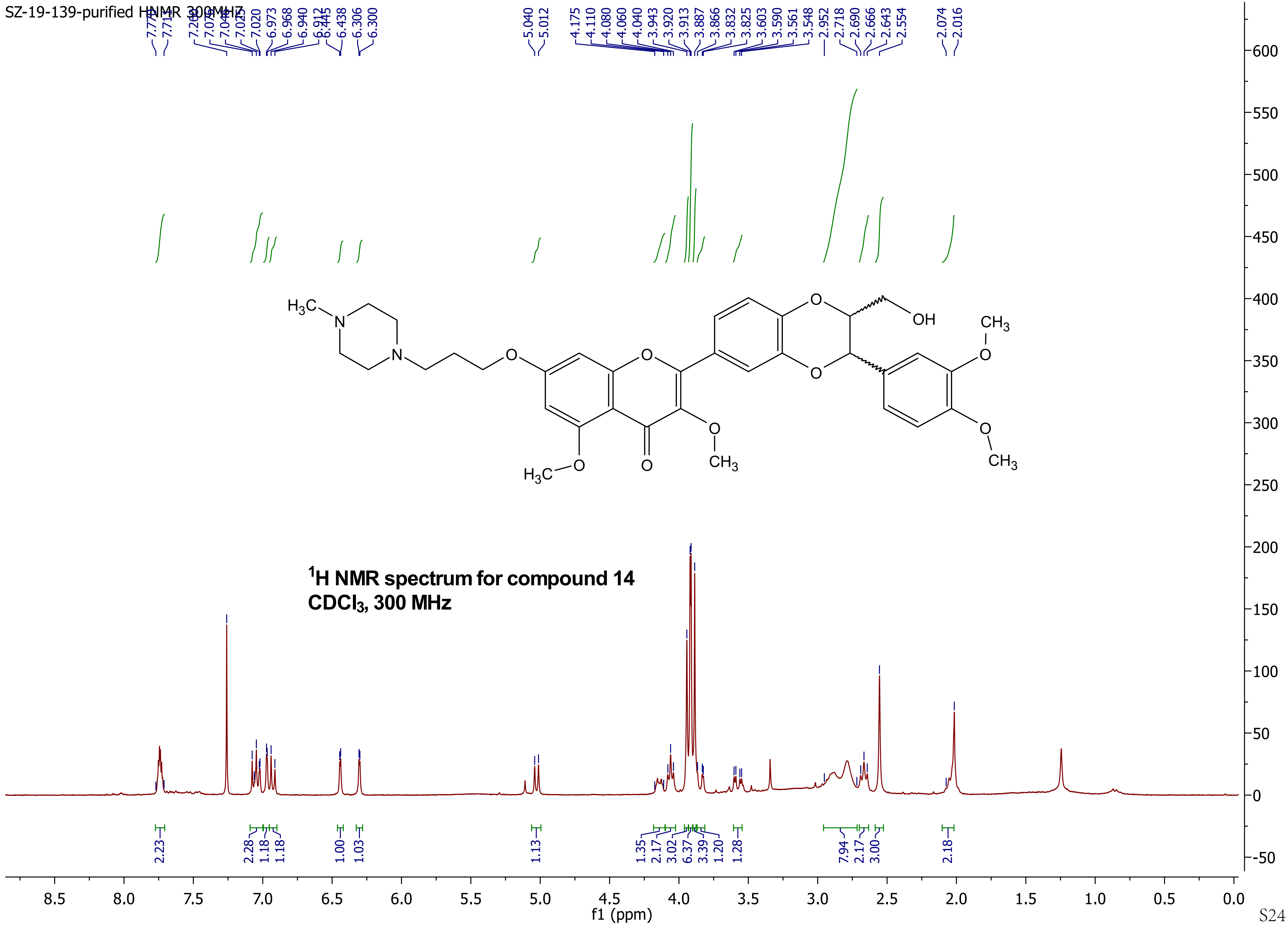

SZ-19-139-purified HNMR 300MHz
¹H NMR spectrum for compound 14
CDCl₃, 300 MHz
f1 (ppm)

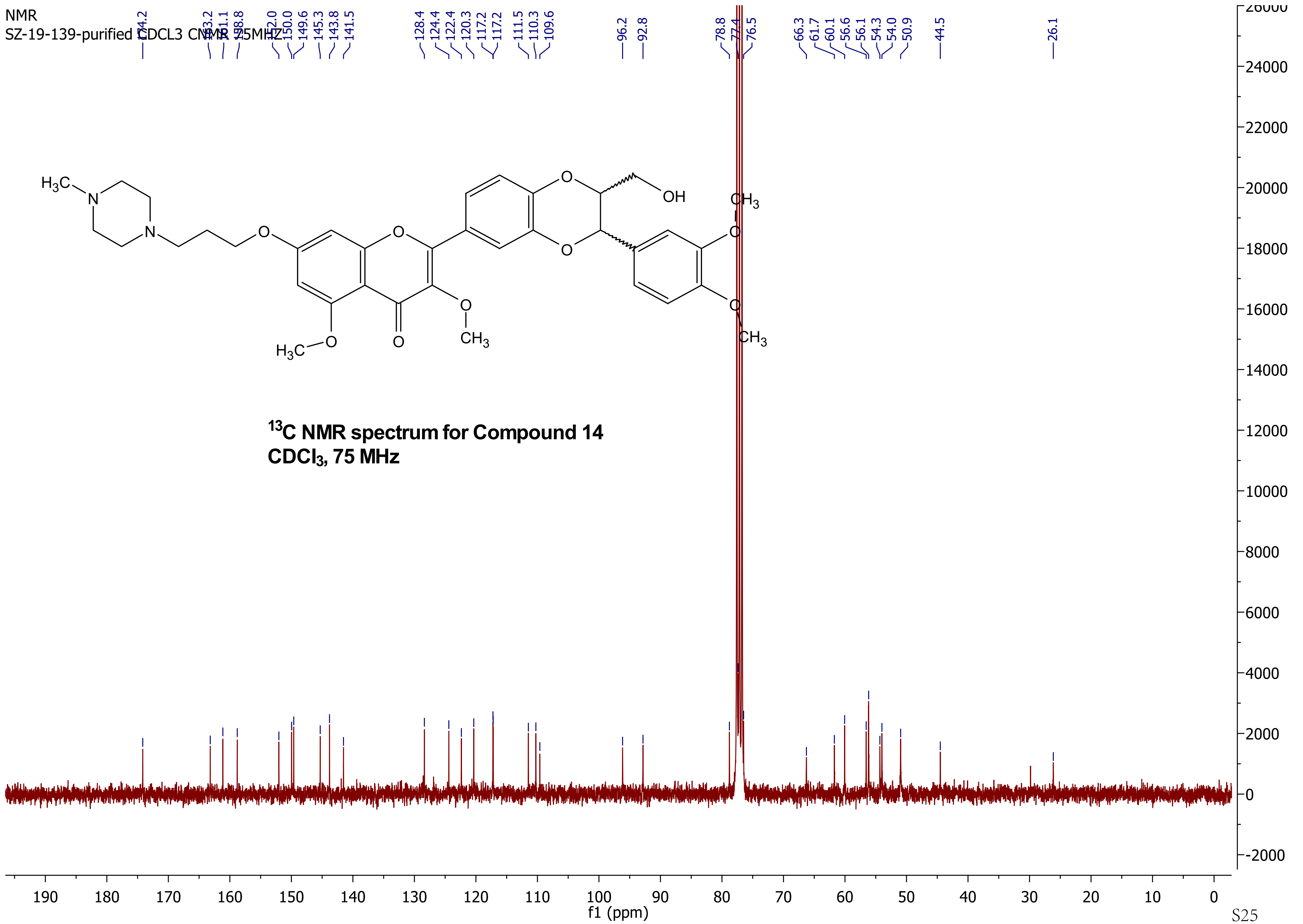
NMR
SZ-19-139-purified CDCL3 CNMR 75MHZ
163.2
161.1
158.8
152.0
150.0
149.6
145.3
143.8
141.5
128.4
124.4
122.4
120.3
117.2
117.2
111.5
110.3
109.6
96.2
92.8
78.8
77.4
76.5
66.3
61.7
60.1
56.6
56.1
54.3
54.0
50.9
44.5
26.1
H3C
N
N
O
O
O
O
O
OH
CH3
O
O
CH3
H3C
O
O
CH3
13C NMR spectrum for Compound 14
CDCl3, 75 MHz
f1 (ppm)

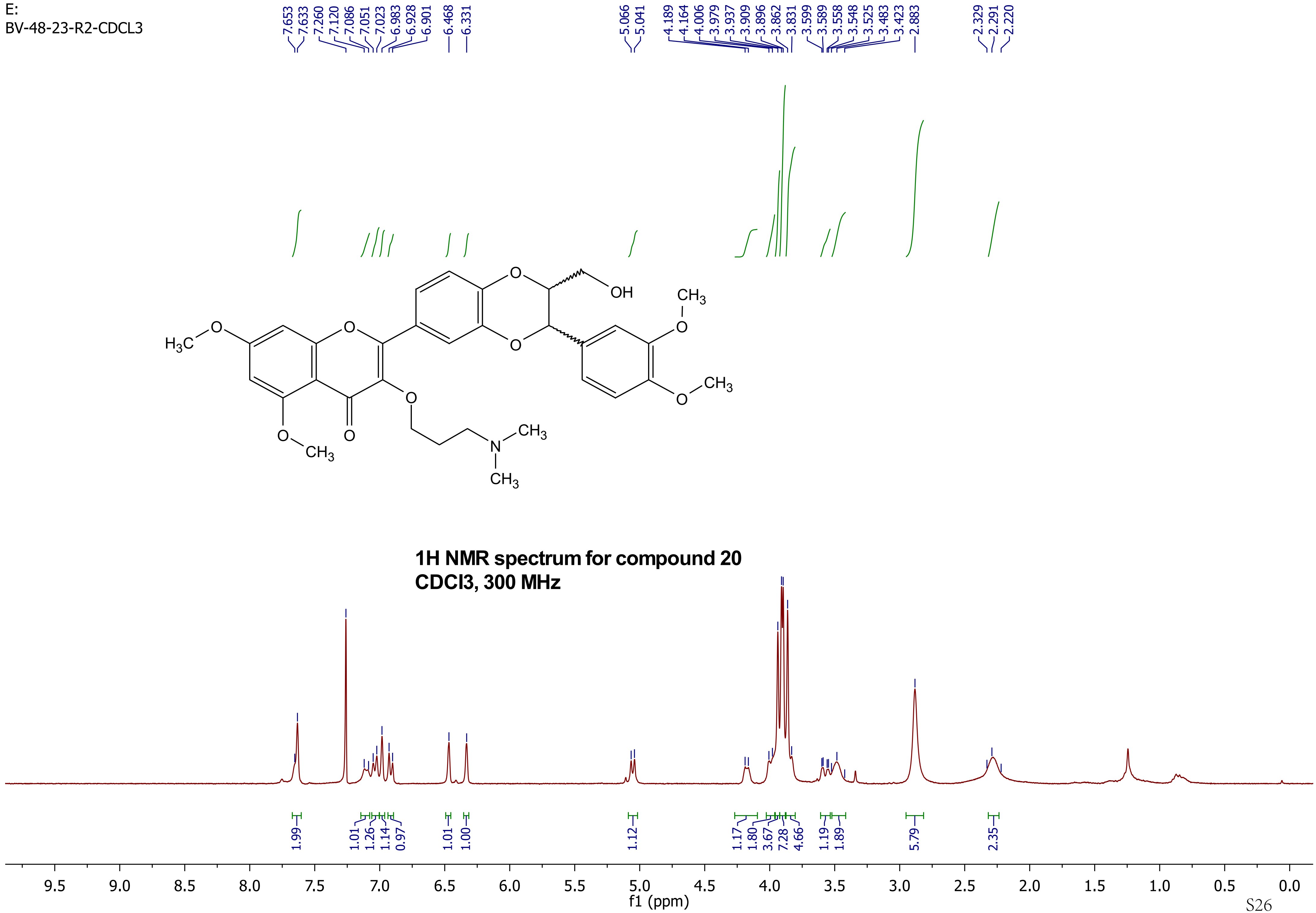
E:
BV-48-23-R2-CDCL3
7.653
7.633
7.260
7.120
7.086
7.051
7.023
6.983
6.928
6.901
6.468
6.331
5.066
5.041
4.189
4.164
4.006
3.979
3.937
3.909
3.896
3.862
3.831
3.599
3.589
3.558
3.548
3.525
3.483
3.423
2.883
2.329
2.291
2.220
H3C
O
O
O
O
OH
O
CH3
O
O
CH3
O
O
N
CH3
CH3
CH3
O
CH3
1H NMR spectrum for compound 20
CDCl3, 300 MHz
1.99
1.01
1.26
1.14
0.97
1.01
1.00
1.12
1.17
1.80
3.67
7.28
4.66
1.19
1.89
5.79
2.35
9.5
9.0
8.5
8.0
7.5
7.0
6.5
6.0
5.5
5.0
4.5
4.0
3.5
3.0
2.5
2.0
1.5
1.0
0.5
0.0
f1 (ppm)

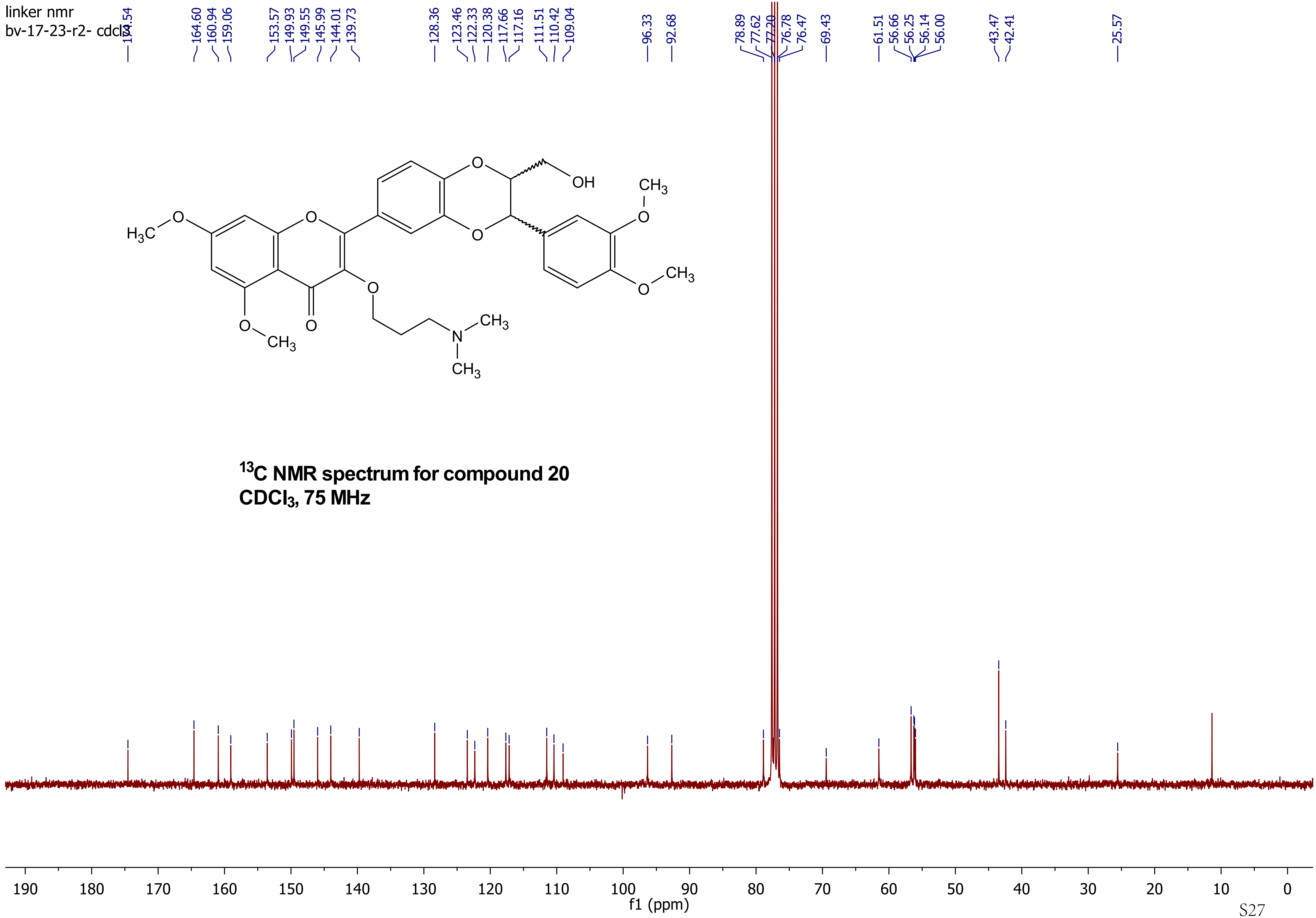

^{13}C NMR spectrum for compound 20
$CDCl_3$, 75 MHz

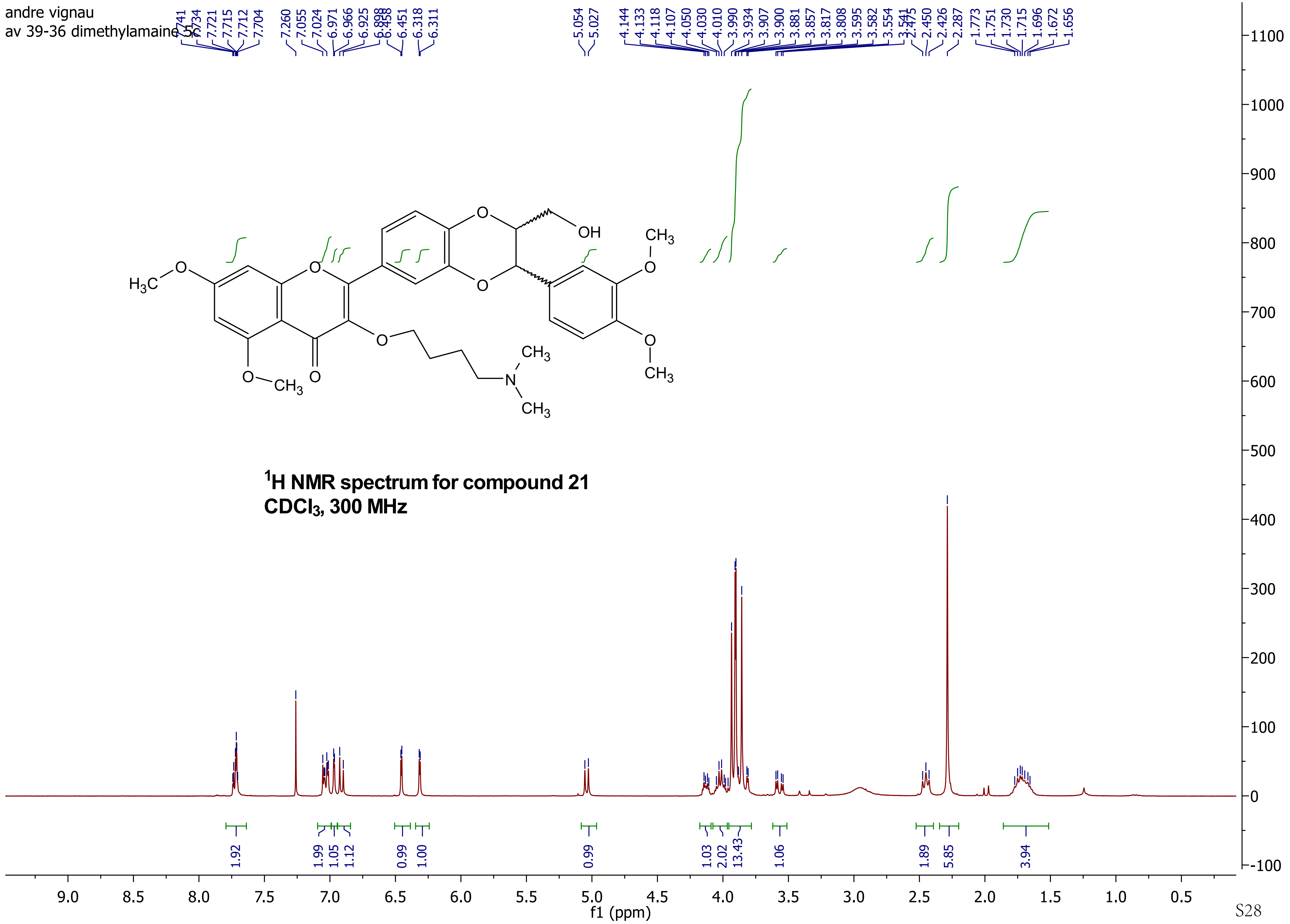

andre vignau
av 39-36 dimethylamaine 5c
7.741 7.734 7.721 7.715 7.712 7.704 7.260 7.055 7.024 6.971 6.966 6.925 6.898 6.458 6.451 6.318 6.311
5.054 5.027
4.144 4.133 4.118 4.107 4.050 4.030 4.010 3.990 3.934 3.907 3.900 3.881 3.857 3.817 3.808 3.595 3.582 3.554 3.541 2.475 2.450 2.426 2.287 1.773 1.751 1.730 1.715 1.696 1.672 1.656
OH
CH3
O
H3C
O
CH3
N
CH3
CH3
1H NMR spectrum for compound 21
CDCl3, 300 MHz
1.92 1.99 1.05 1.12 0.99 1.00 0.99 1.03 2.02 13.43 1.06 1.89 5.85 3.94
9.0 8.5 8.0 7.5 7.0 6.5 6.0 5.5 5.0 4.5 4.0 3.5 3.0 2.5 2.0 1.5 1.0 0.5
f1 (ppm)
1100 1000 900 800 700 600 500 400 300 200 100 0 -100

andre vignau
av 39-36 dimethylamine 5c c13

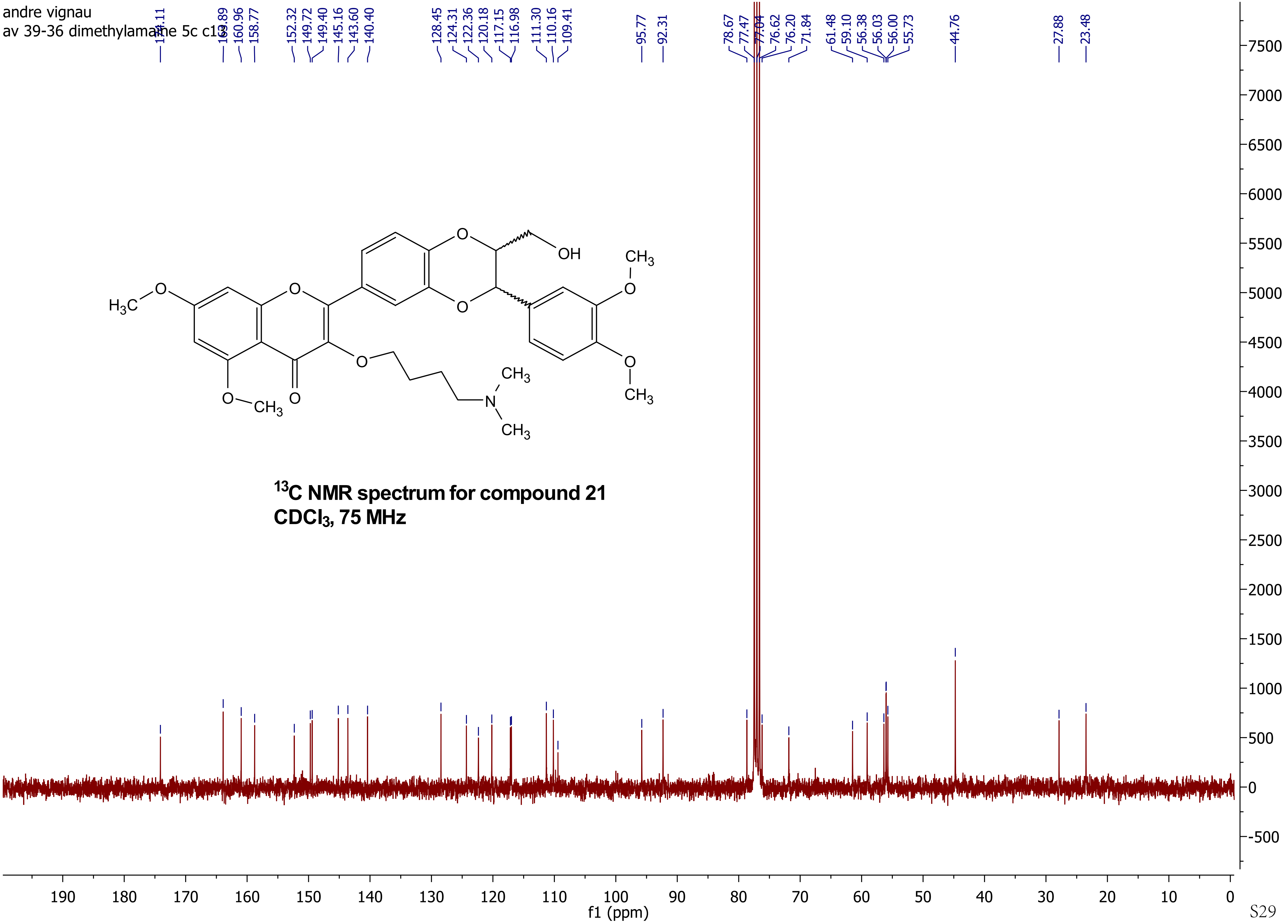

^{13}C NMR spectrum for compound 21
$CDCl_3$, 75 MHz

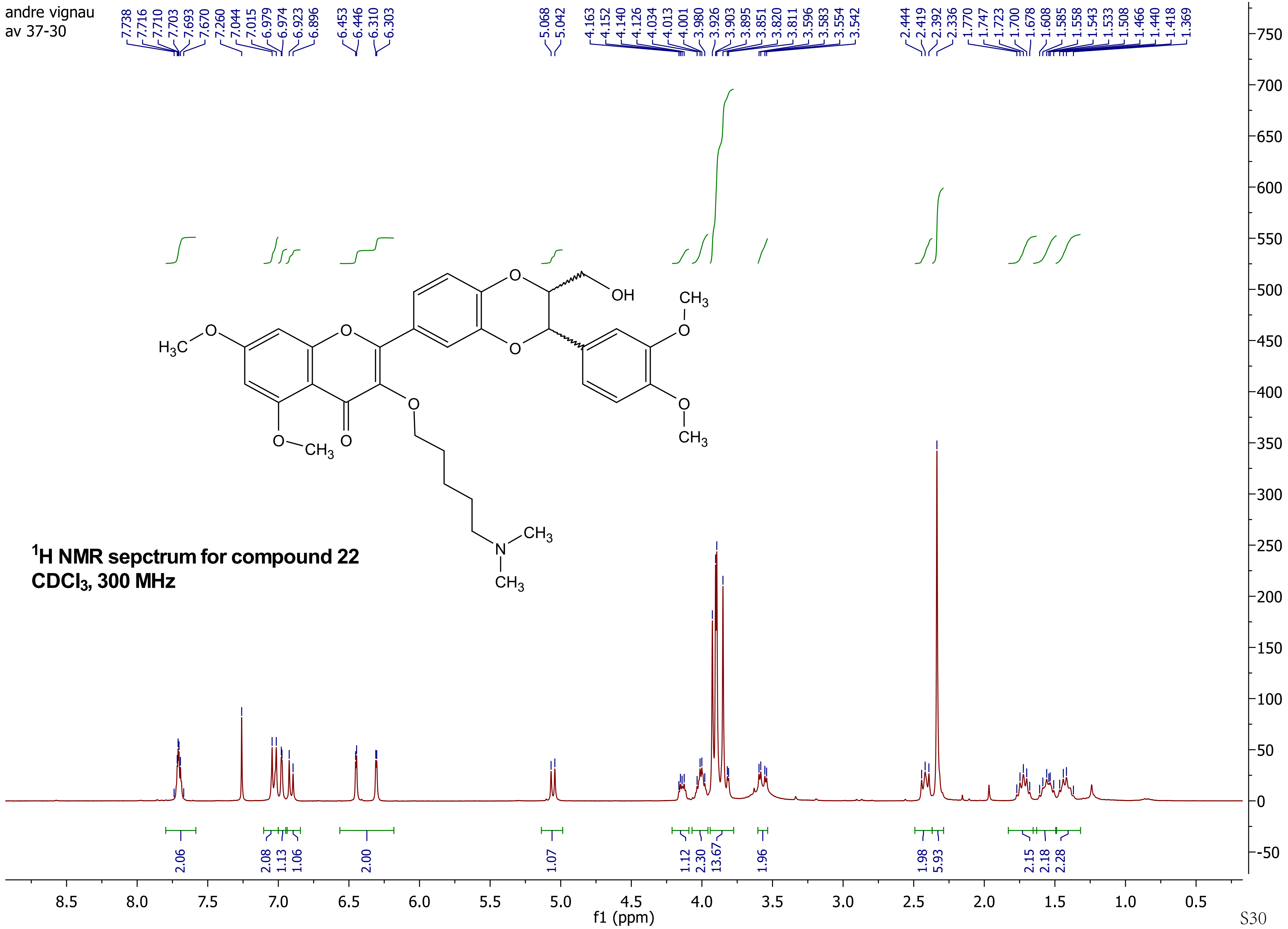
andre vignau
av 37-30
7.738 7.716 7.710 7.703 7.693 7.670 7.260 7.044 7.015 6.979 6.974 6.923 6.896 6.453 6.446 6.310 6.303
5.068 5.042
4.163 4.152 4.140 4.126 4.034 4.013 4.001 3.980 3.926 3.903 3.895 3.851 3.820 3.811 3.596 3.583 3.554 3.542
2.444 2.419 2.392 2.336 1.770 1.747 1.723 1.700 1.678 1.608 1.585 1.558 1.543 1.533 1.508 1.466 1.440 1.418 1.369
OH
CH3
O
H3C
O
O
O
O
O
O
O
CH3
O
CH3
N
CH3
CH3
1H NMR sepctrum for compound 22
CDCl3, 300 MHz
2.06
2.08 1.13 1.06
2.00
1.07
1.12 2.30 13.67
1.96
1.98 5.93
2.15 2.18 2.28
8.5 8.0 7.5 7.0 6.5 6.0 5.5 5.0 4.5 4.0 3.5 3.0 2.5 2.0 1.5 1.0 0.5
f1 (ppm)
750 700 650 600 550 500 450 400 350 300 250 200 150 100 50 0 -50

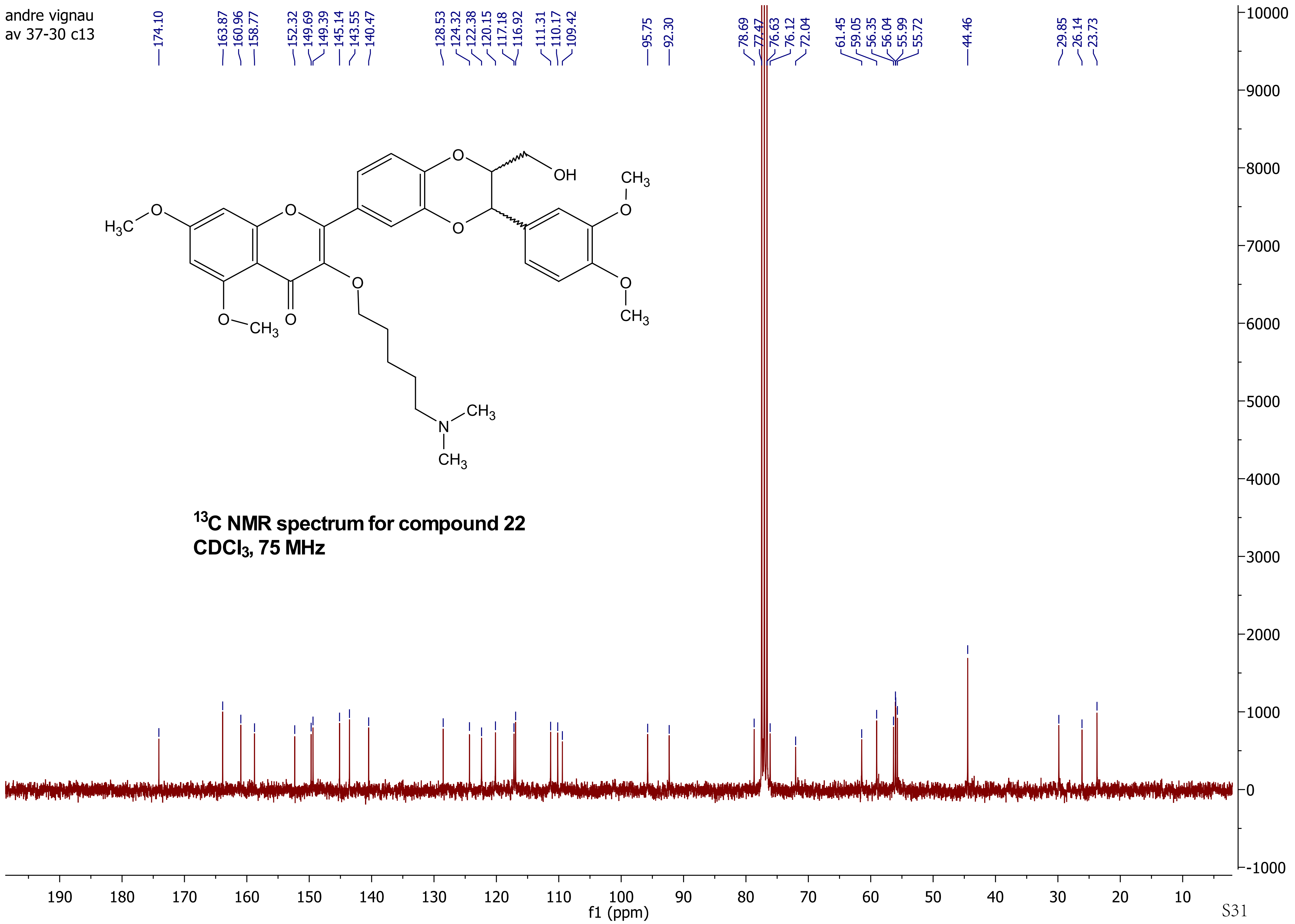
andre vignau
av 37-30 c13
174.10
163.87
160.96
158.77
152.32
149.69
149.39
145.14
143.55
140.47
128.53
124.32
122.38
120.15
117.18
116.92
111.31
110.17
109.42
95.75
92.30
78.69
77.47
76.63
76.12
72.04
61.45
59.05
56.35
56.04
55.99
55.72
44.46
29.85
26.14
23.73
13C NMR spectrum for compound 22
CDCl3, 75 MHz
f1 (ppm)

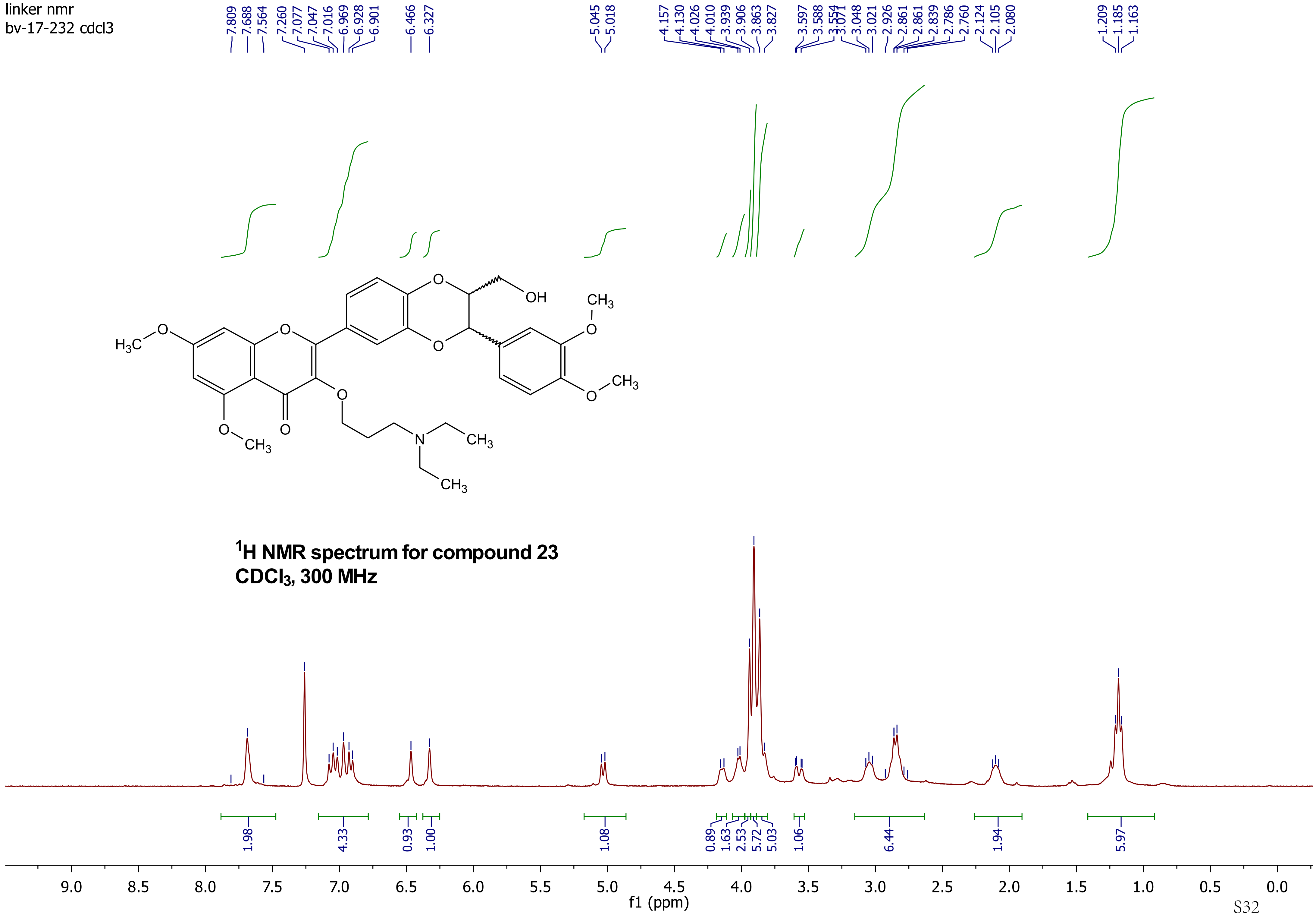

^{1}H NMR spectrum for compound 23
$CDCl_3$, 300 MHz

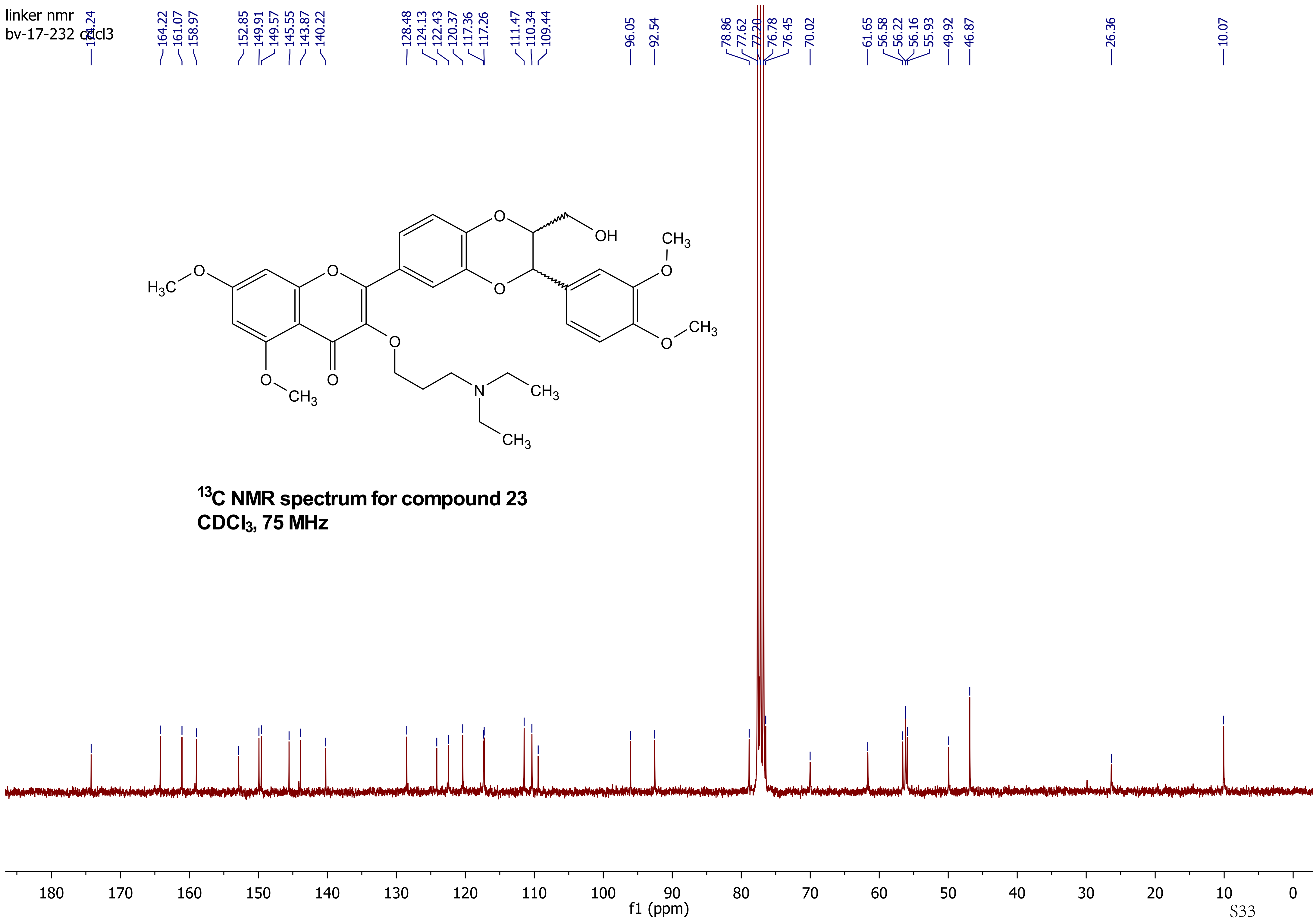
linker nmr
bv-17-232 cdcl3
174.24
164.22
161.07
158.97
152.85
149.91
149.57
145.55
143.87
140.22
128.48
124.13
122.43
120.37
117.36
117.26
111.47
110.34
109.44
96.05
92.54
78.86
77.62
77.20
76.78
76.45
70.02
61.65
56.58
56.22
56.16
55.93
49.92
46.87
26.36
10.07
OH
CH_3
O
H_3C
N
^{13}C NMR spectrum for compound 23
$CDCl_3$, 75 MHz
180 170 160 150 140 130 120 110 100 90 80 70 60 50 40 30 20 10 0
f1 (ppm)

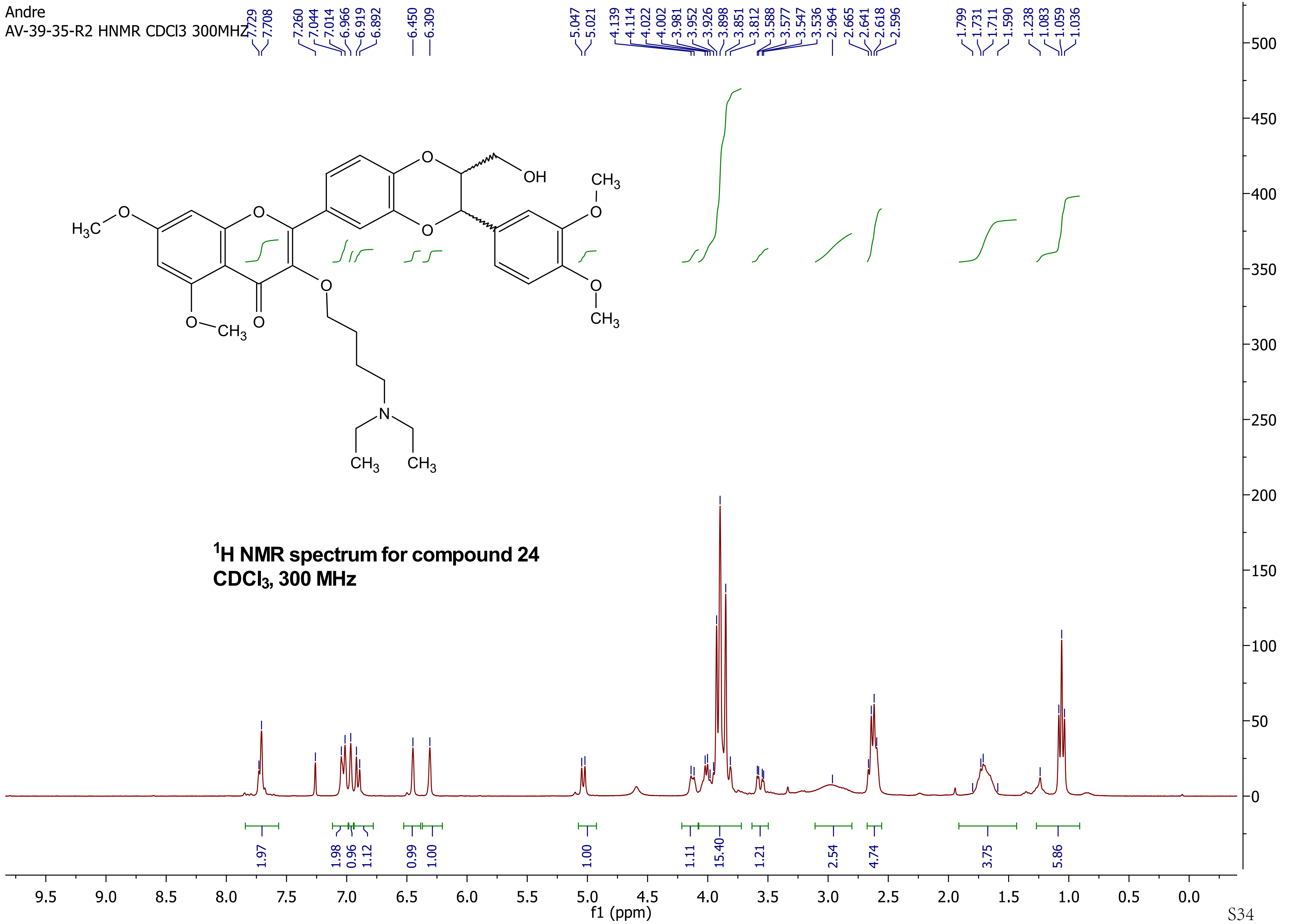

^{1}H NMR spectrum for compound 24
$CDCl_3$, 300 MHz

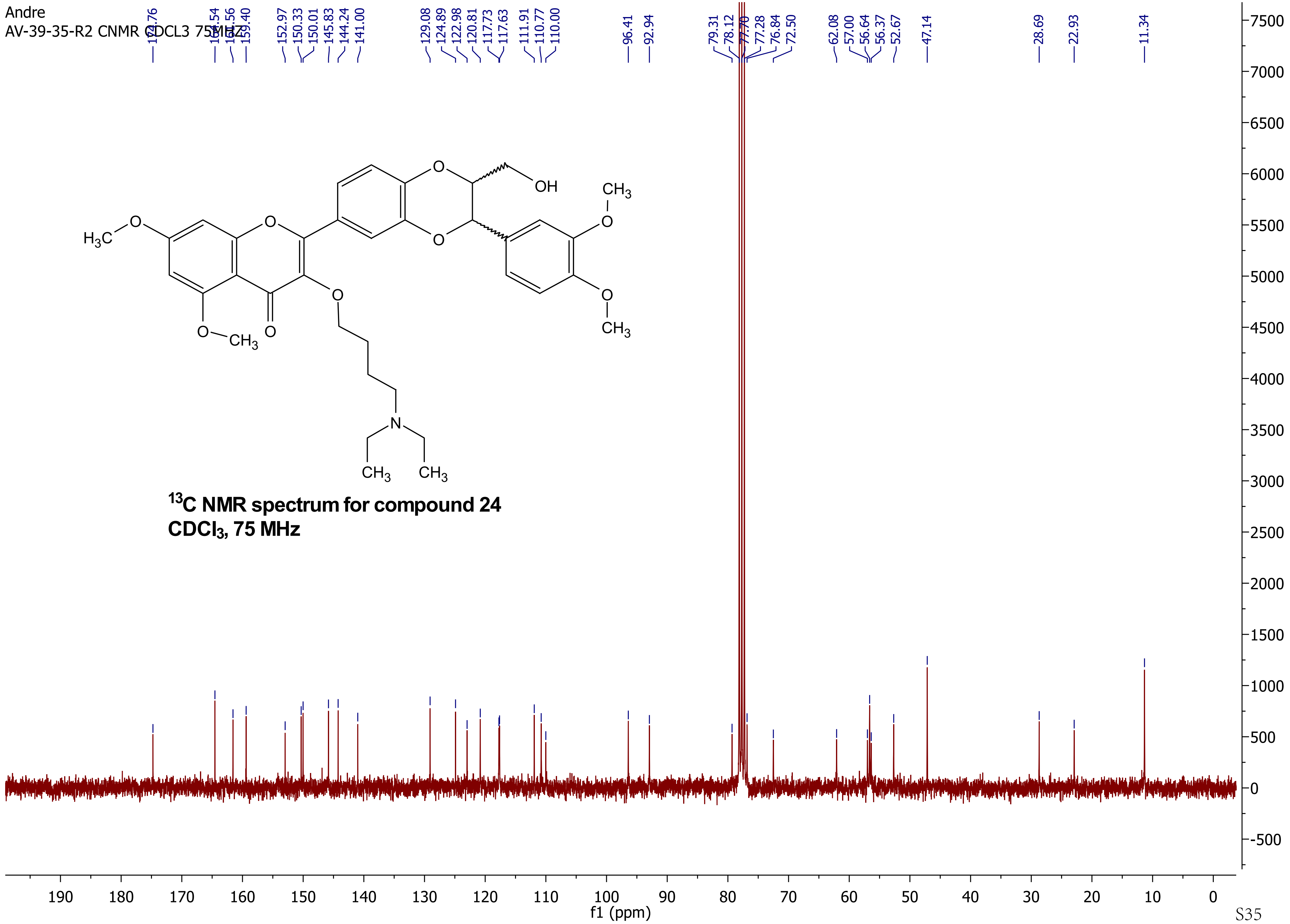

^{13}C NMR spectrum for compound 24
$CDCl_3$, 75 MHz

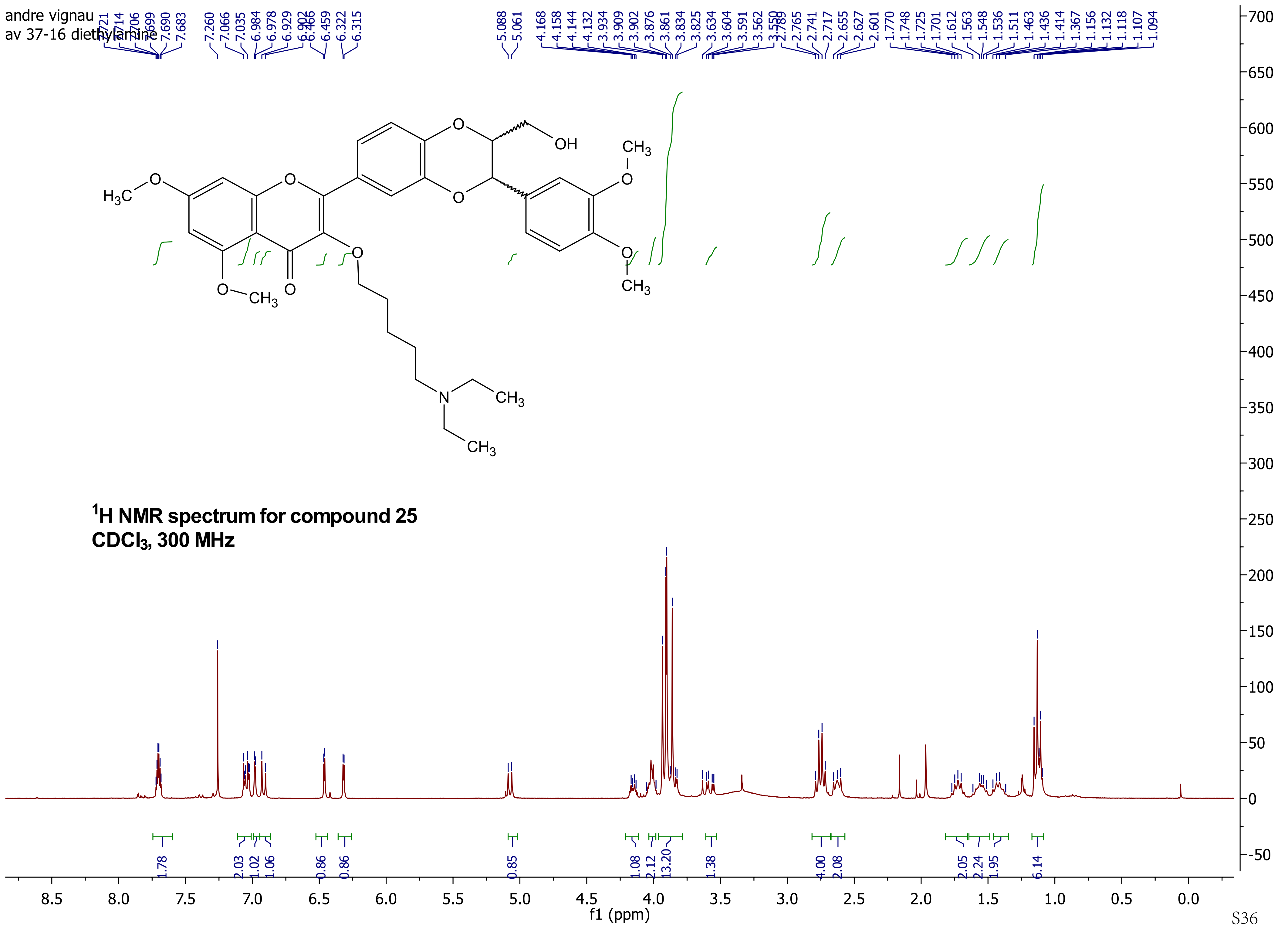
andre vignau
av 37-16 diethylamine
OH
H_3C
CH_3
¹H NMR spectrum for compound 25
CDCl3, 300 MHz
f1 (ppm)

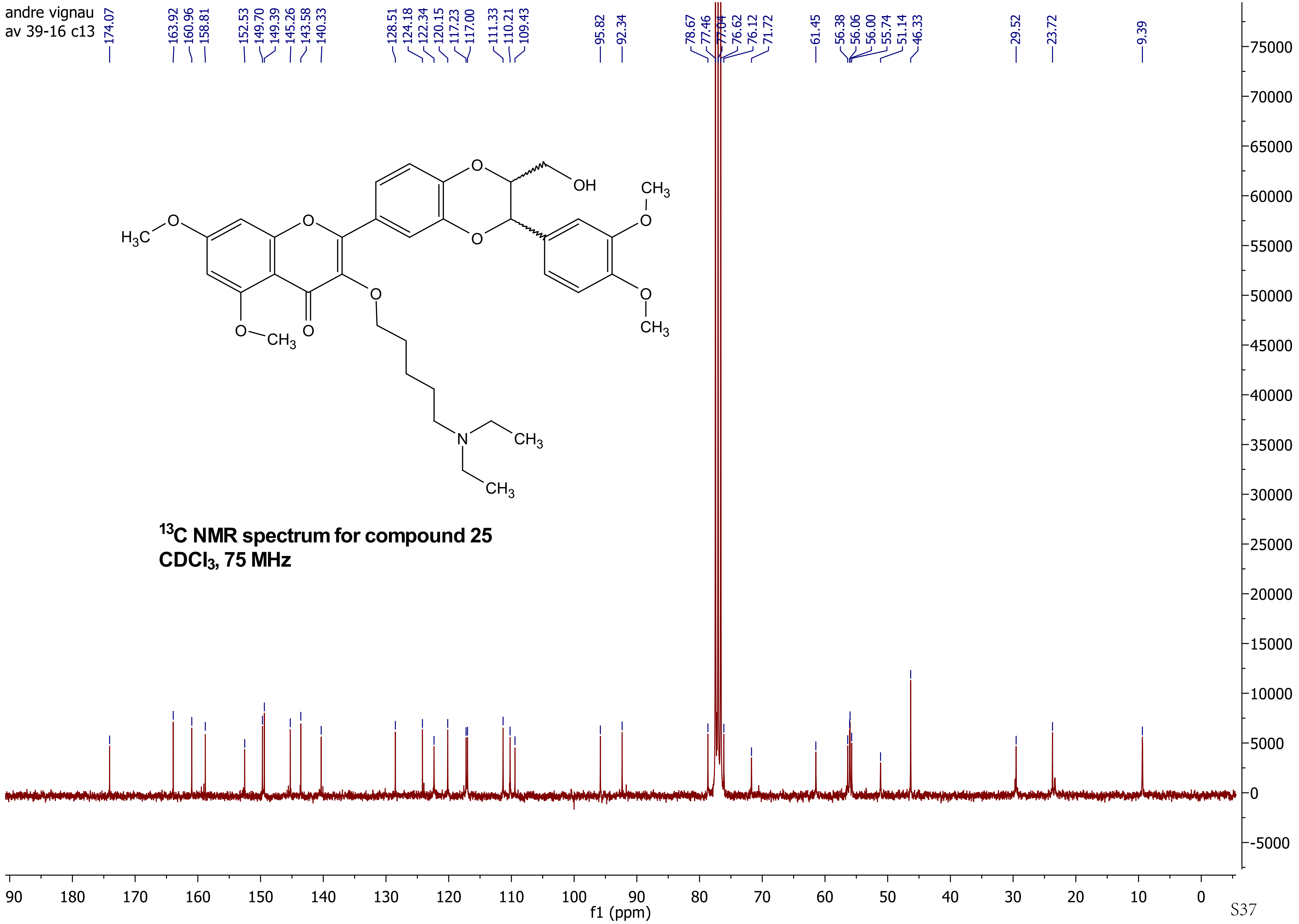
andre vignau
av 39-16 c13
174.07
163.92
160.96
158.81
152.53
149.70
149.39
145.26
143.58
140.33
128.51
124.18
122.34
120.15
117.23
117.00
111.33
110.21
109.43
95.82
92.34
78.67
77.46
77.04
76.62
76.12
71.72
61.45
56.38
56.06
56.00
55.74
51.14
46.33
29.52
23.72
9.39
OH
CH3
O
H3C
O
O
O
O
O
O
O
CH3
O
CH3
N
CH3
CH3
13C NMR spectrum for compound 25
CDCl3, 75 MHz
f1 (ppm)

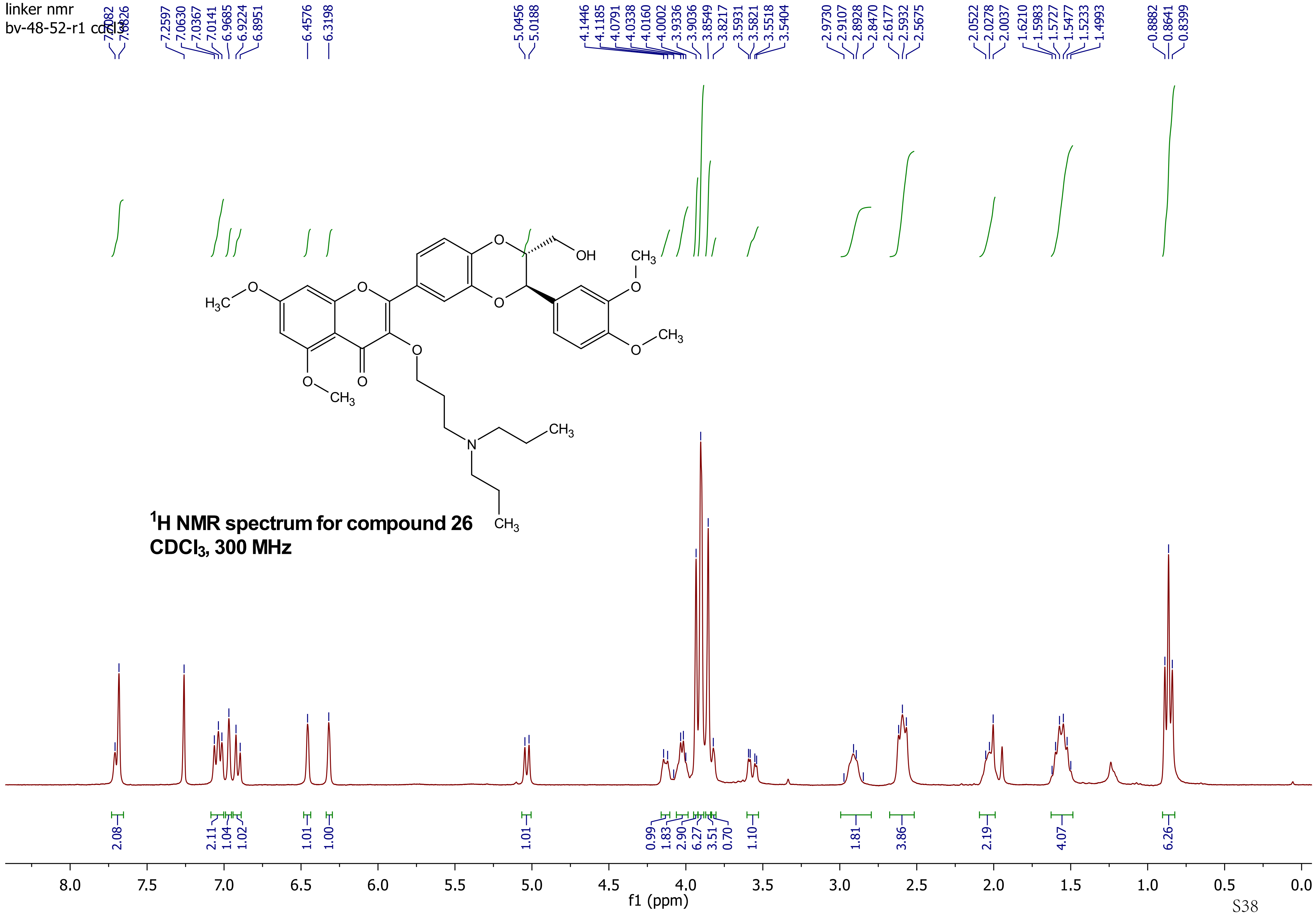

^{1}H NMR spectrum for compound 26
$CDCl_3$, 300 MHz

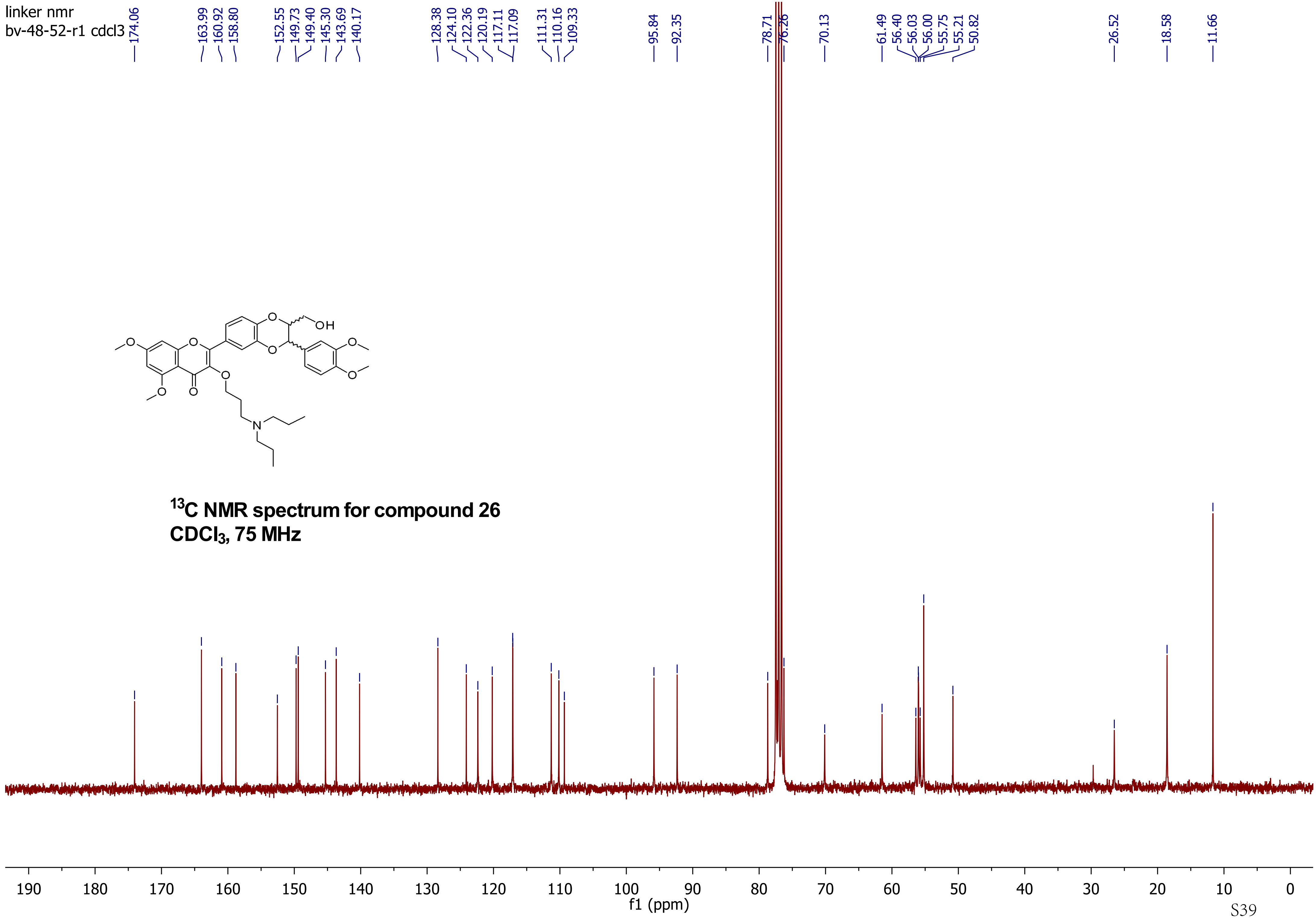

^{13}C NMR spectrum for compound 26
$CDCl_3$, 75 MHz

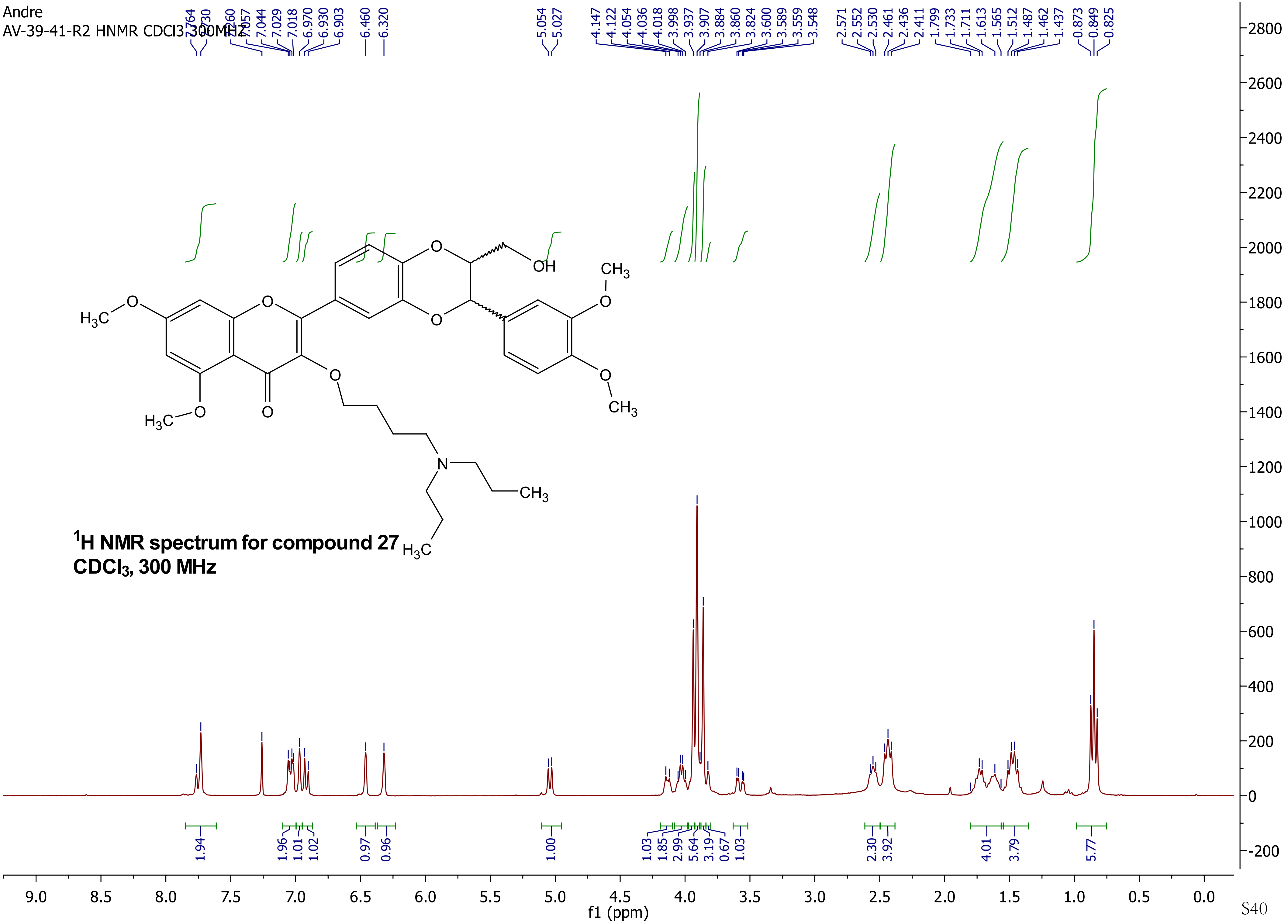

^{1}H NMR spectrum for compound 27
$CDCl_3$, 300 MHz

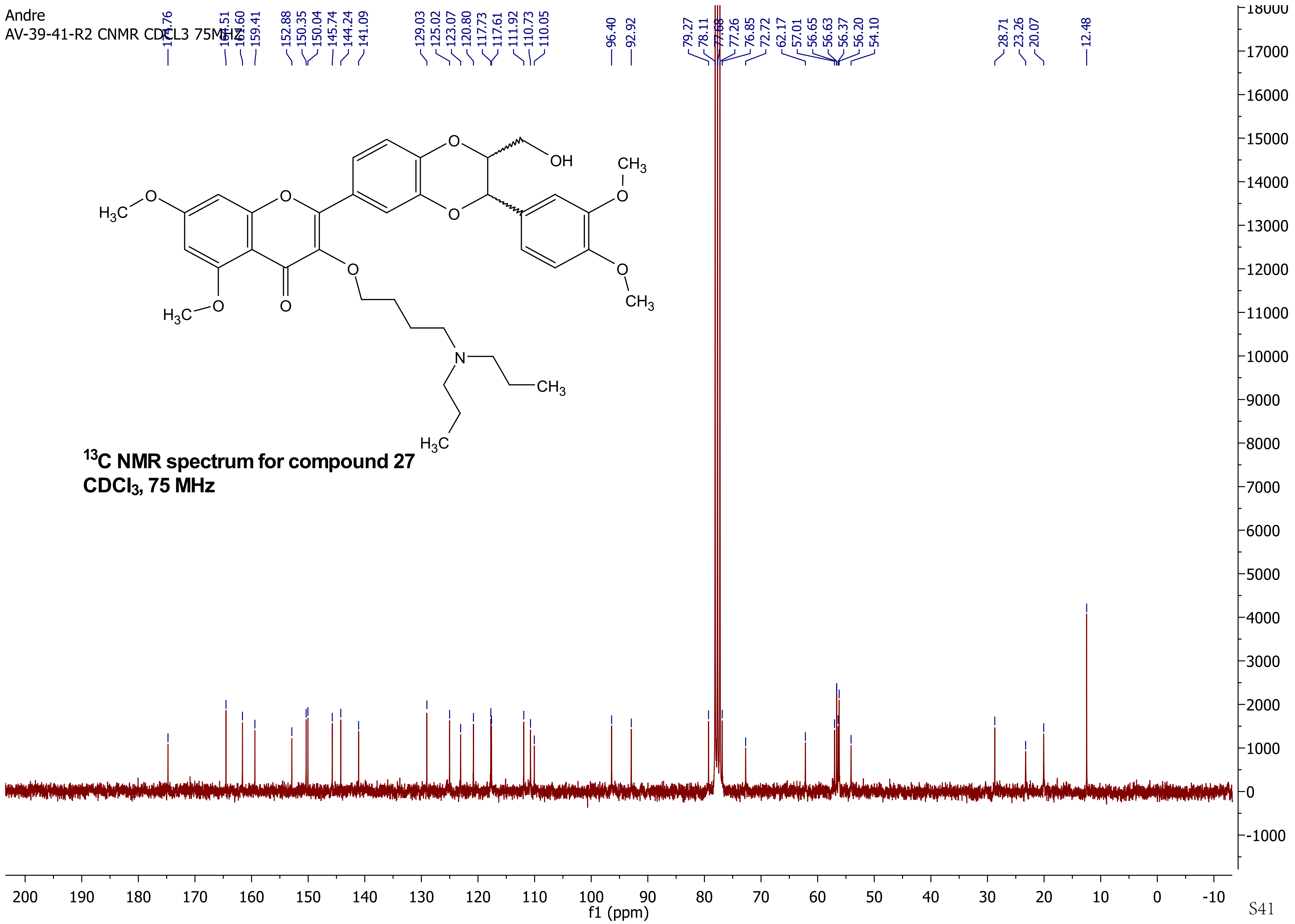

^{13}C NMR spectrum for compound 27
$CDCl_3$, 75 MHz

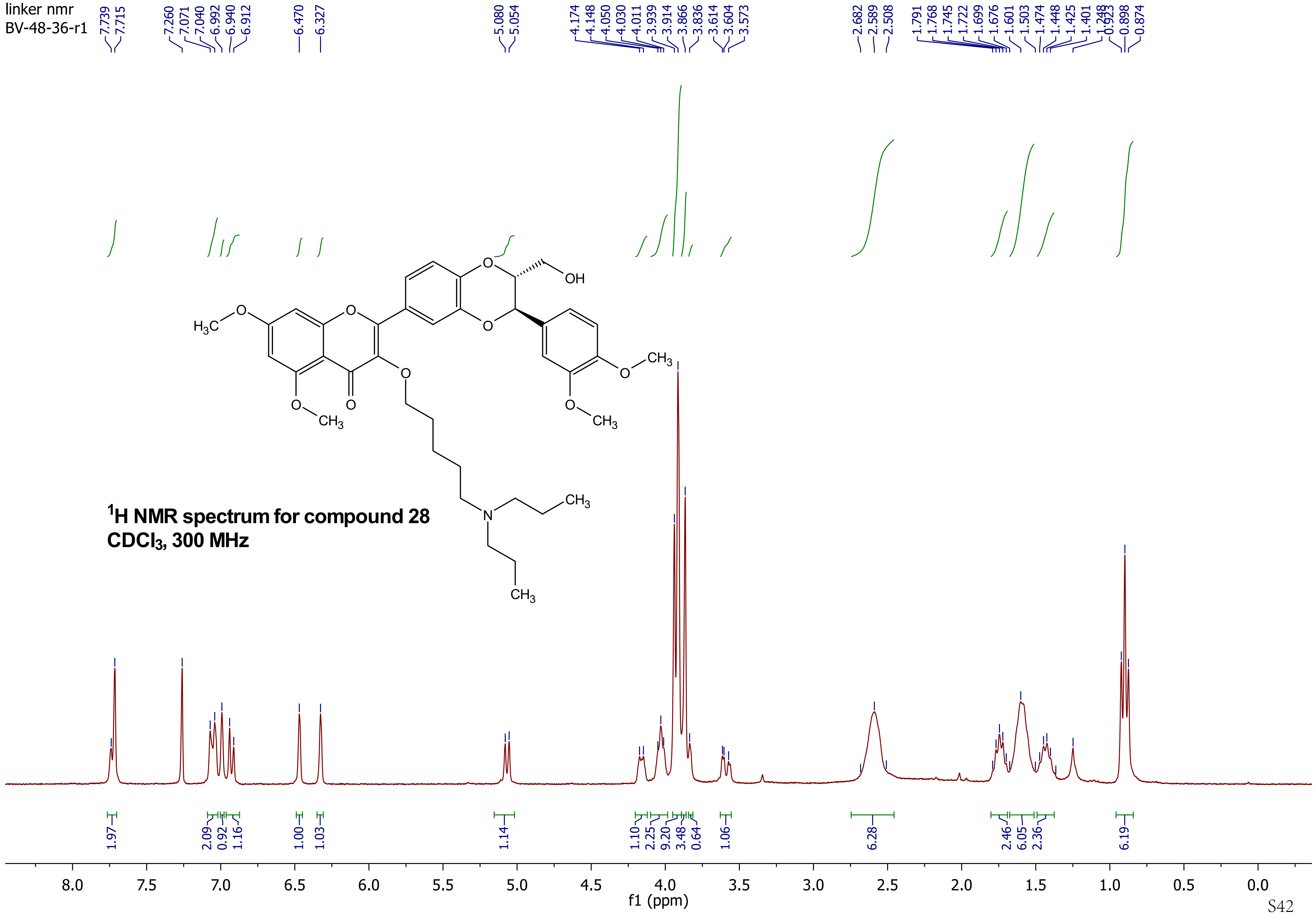

^1H NMR spectrum for compound 28
$CDCl_3$, 300 MHz

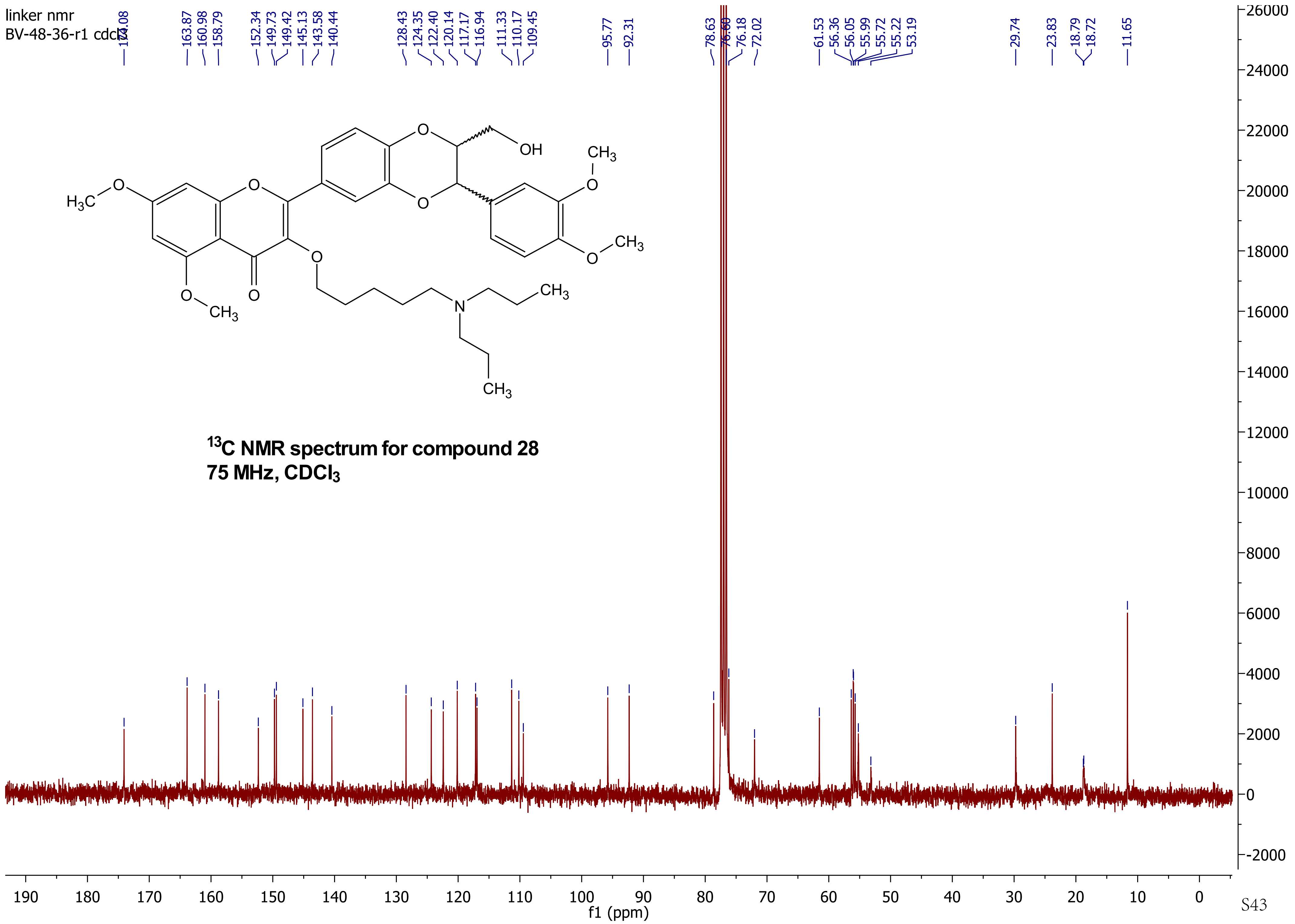
linker nmr
BV-48-36-r1 cdcl3
174.08
163.87
160.98
158.79
152.34
149.73
149.42
145.13
143.58
140.44
128.43
124.35
122.40
120.14
117.17
116.94
111.33
110.17
109.45
95.77
92.31
78.63
76.60
76.18
72.02
61.53
56.36
56.05
55.99
55.72
55.22
53.19
29.74
23.83
18.79
18.72
11.65
OH
CH3
O
H3C
N
^{13}C NMR spectrum for compound 28
75 MHz, $CDCl_3$
f1 (ppm)

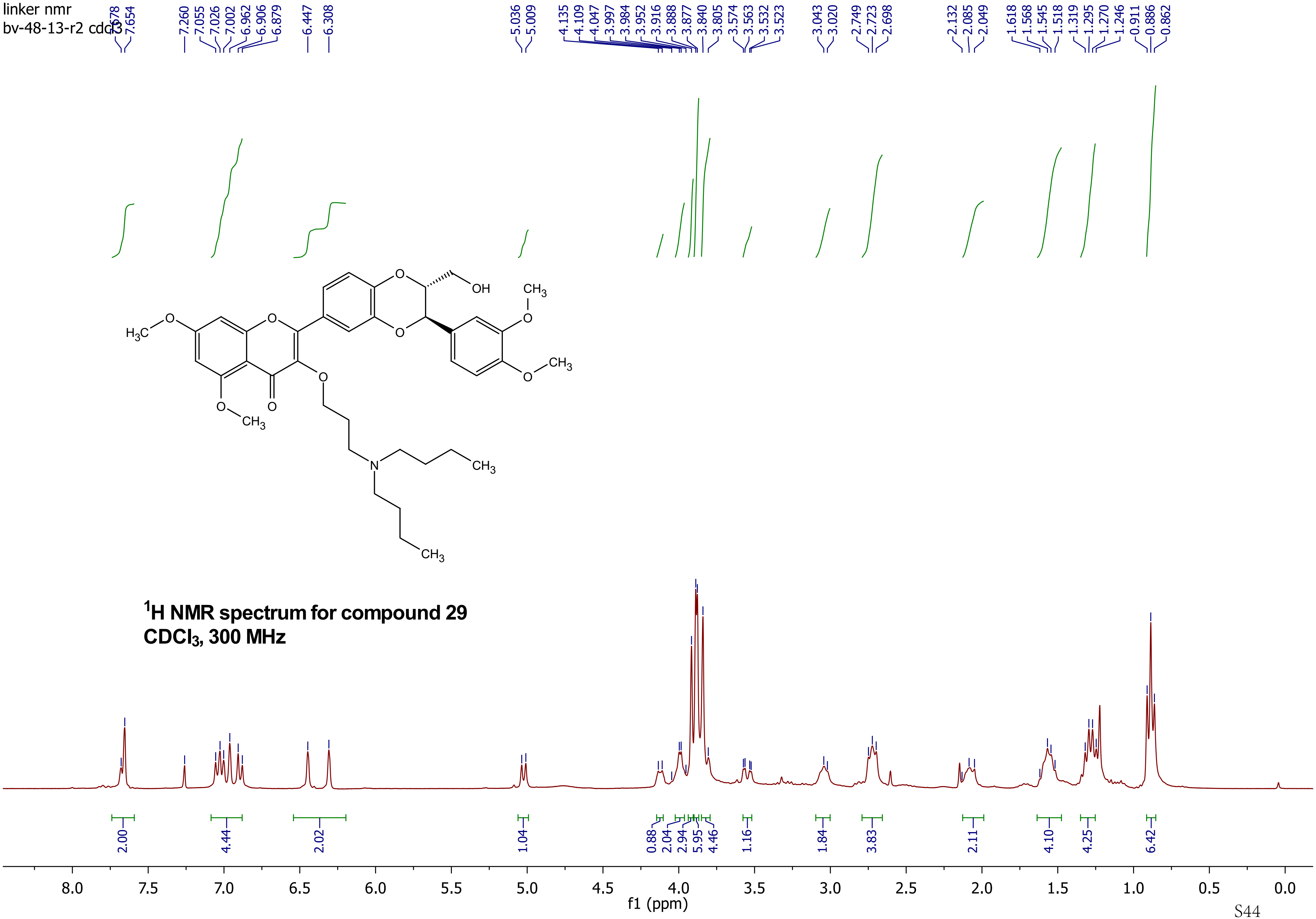

^{1}H NMR spectrum for compound 29
$CDCl_3$, 300 MHz

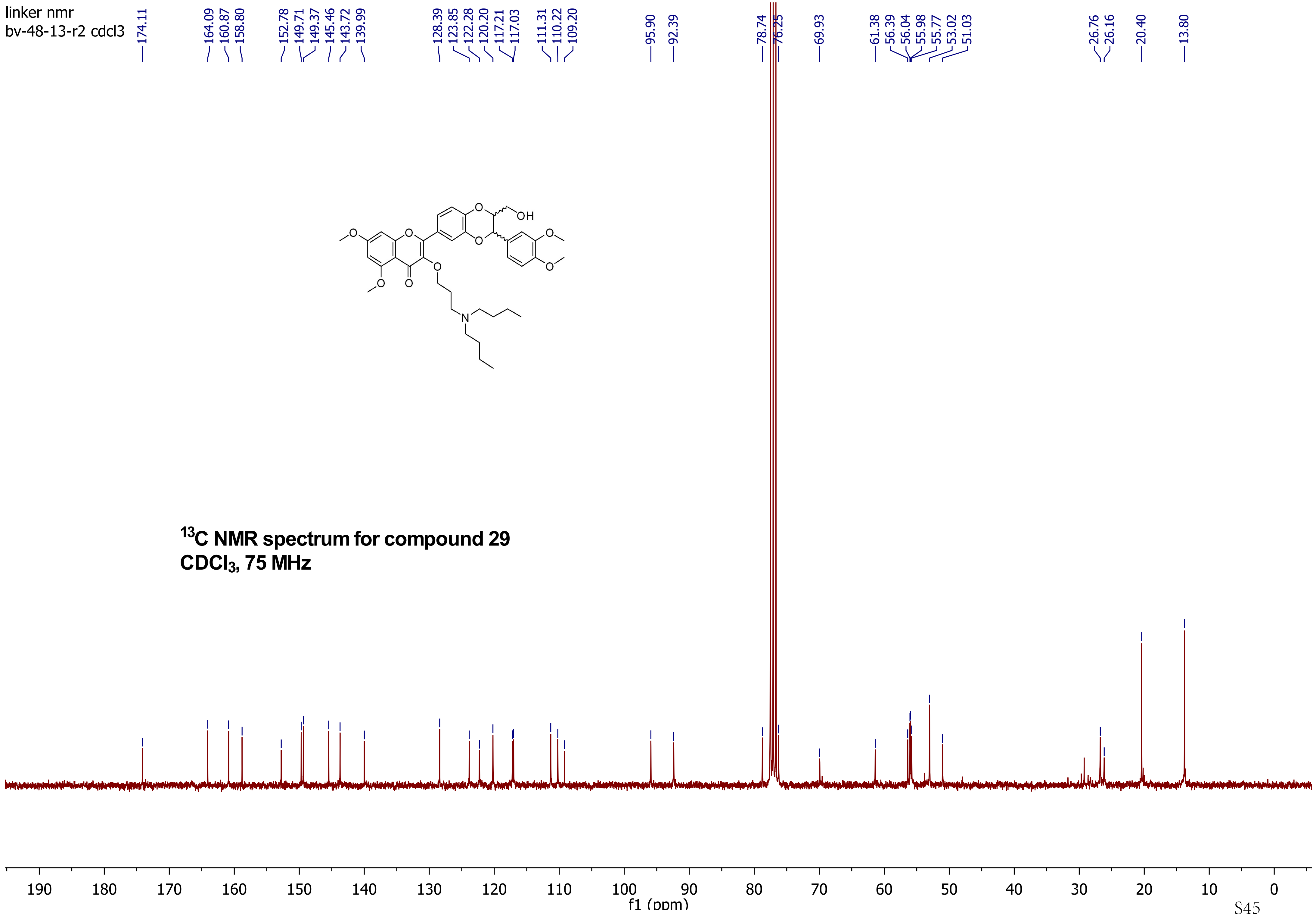

^{13}C NMR spectrum for compound 29
$CDCl_3$, 75 MHz

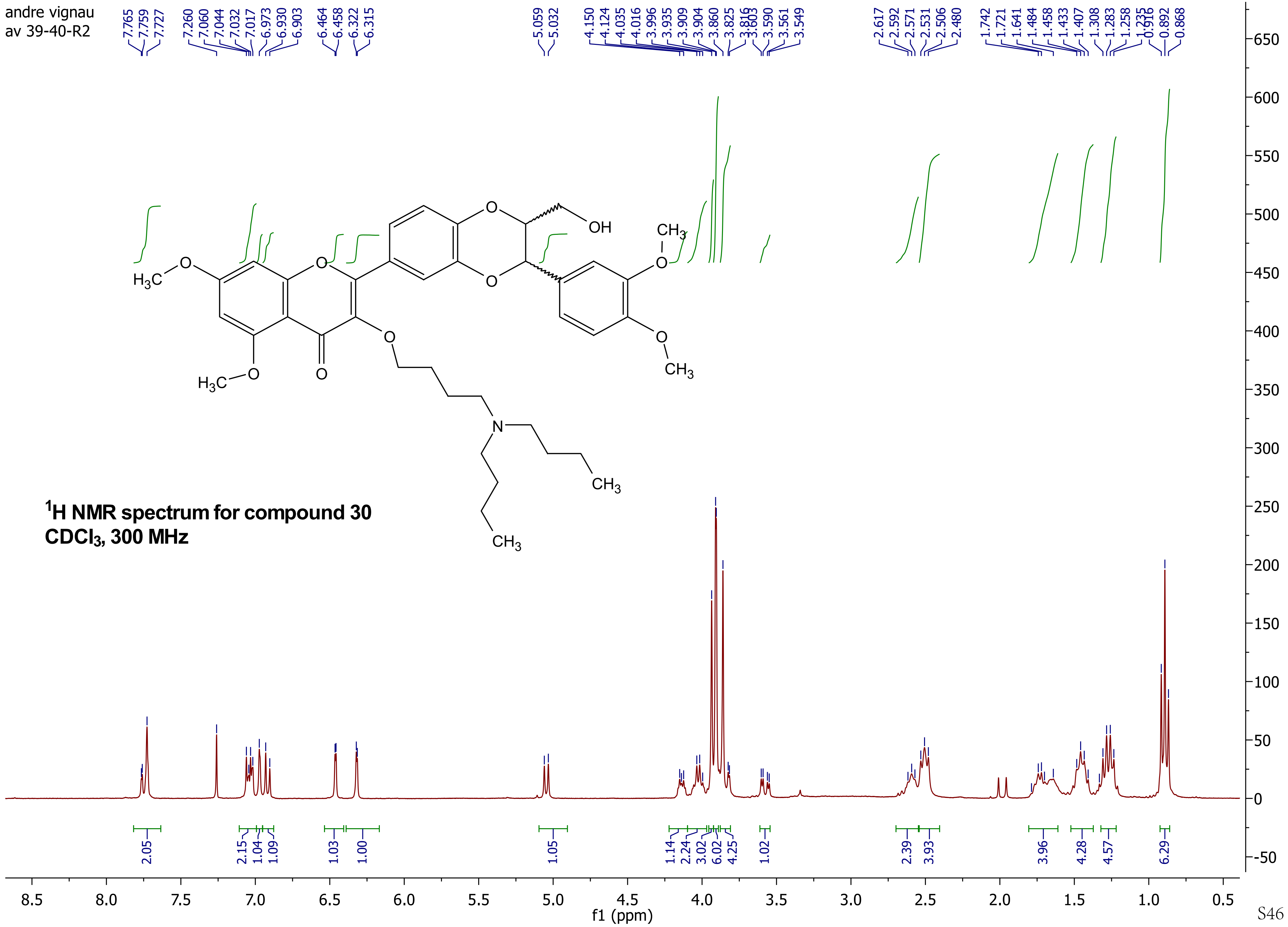
andre vignau
av 39-40-R2
7.765
7.759
7.727
7.260
7.060
7.044
7.032
7.017
6.973
6.930
6.903
6.464
6.458
6.322
6.315
5.059
5.032
4.150
4.124
4.035
4.016
3.996
3.935
3.909
3.904
3.860
3.825
3.816
3.603
3.590
3.561
3.549
2.617
2.592
2.571
2.531
2.506
2.480
1.742
1.721
1.641
1.484
1.458
1.433
1.407
1.308
1.283
1.258
1.235
0.916
0.892
0.868
OH
CH3
O
H3C
N
1H NMR spectrum for compound 30
CDCl3, 300 MHz
2.05
2.15
1.04
1.09
1.03
1.00
1.05
1.14
2.24
3.02
6.02
4.25
1.02
2.39
3.93
3.96
4.28
4.57
6.29
f1 (ppm)

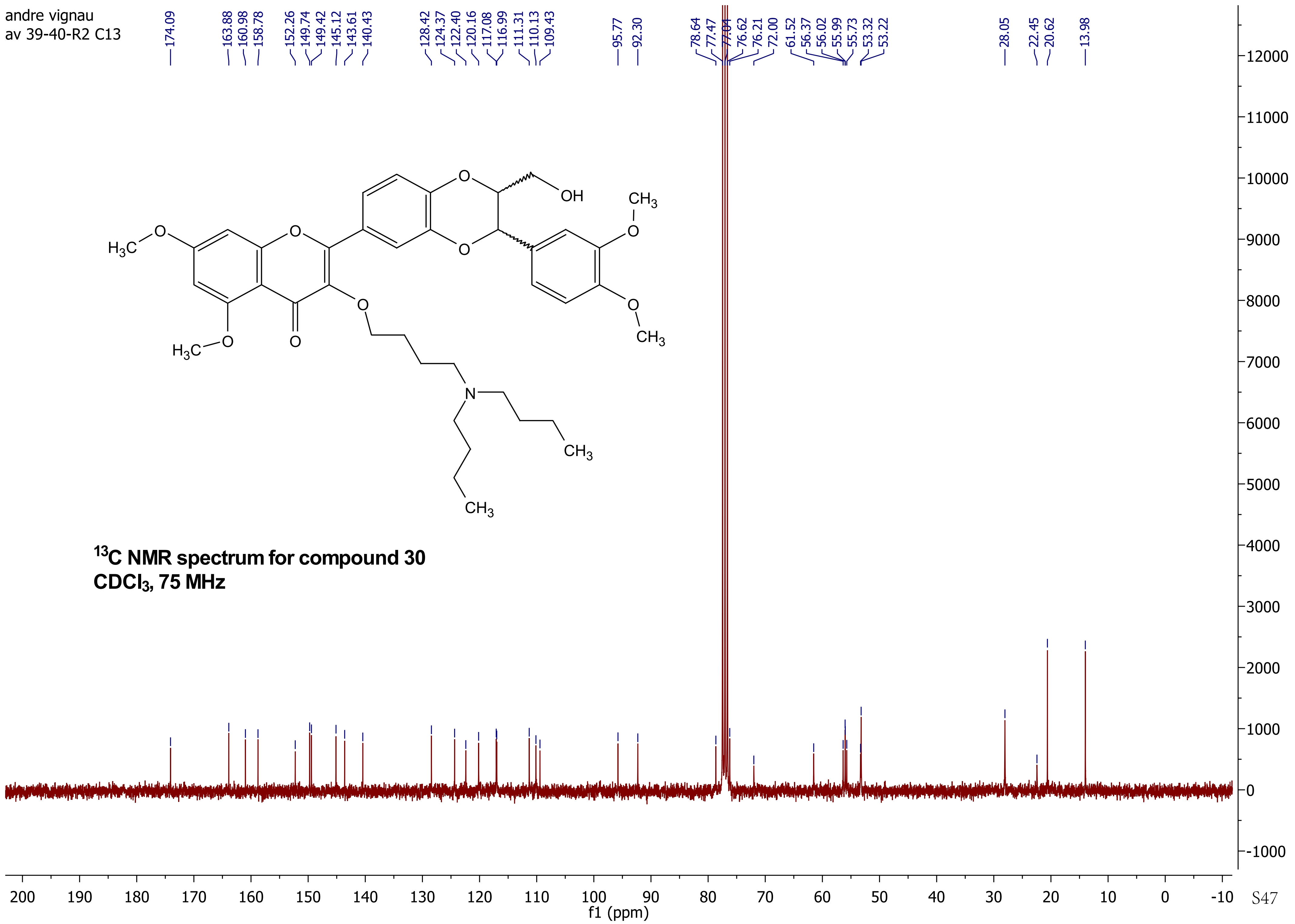

^{13}C NMR spectrum for compound 30
$CDCl_3$, 75 MHz

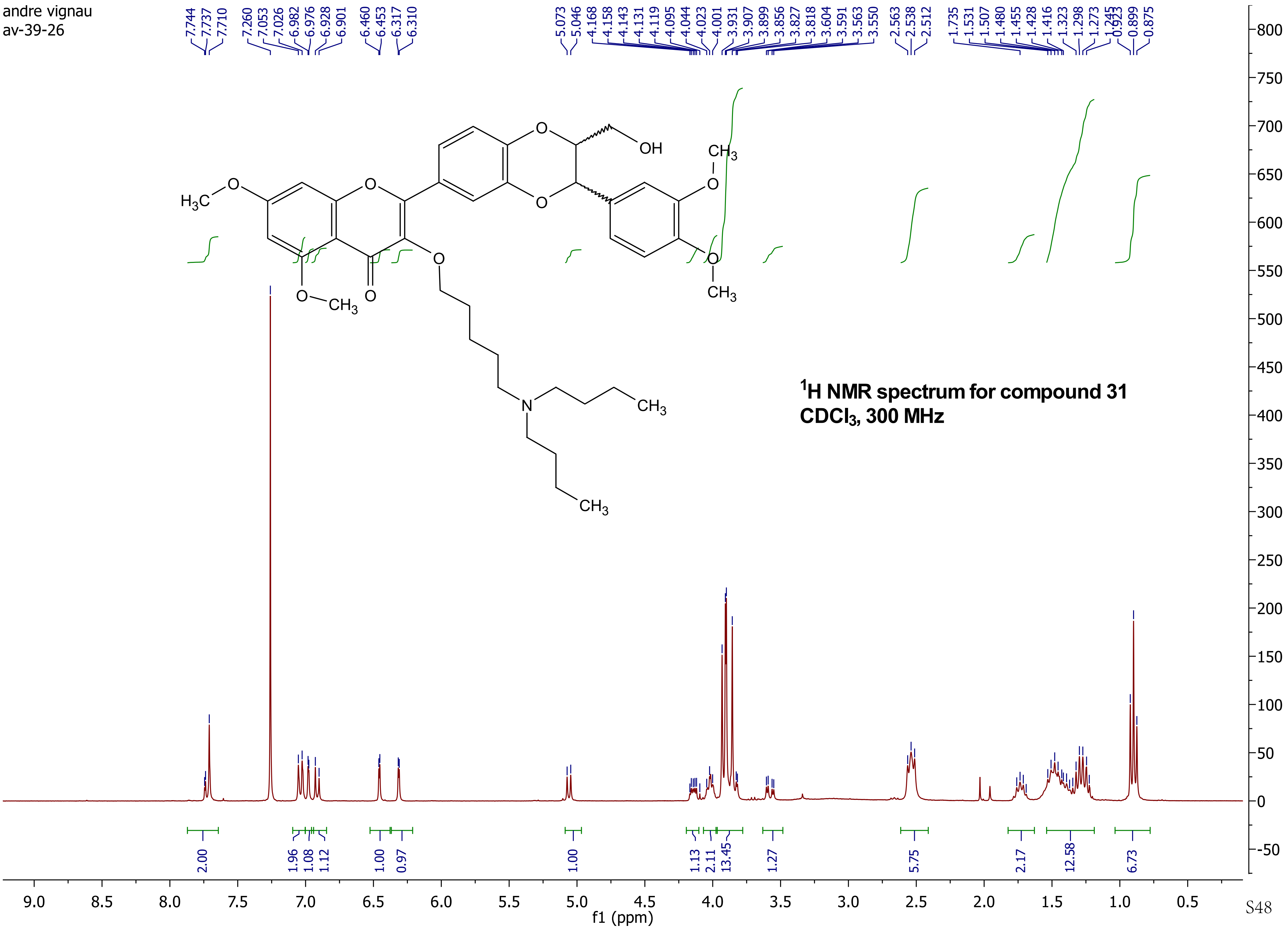
andre vignau
av-39-26
1H NMR spectrum for compound 31
CDCl3, 300 MHz
f1 (ppm)
OH
CH3
H3C
O
N

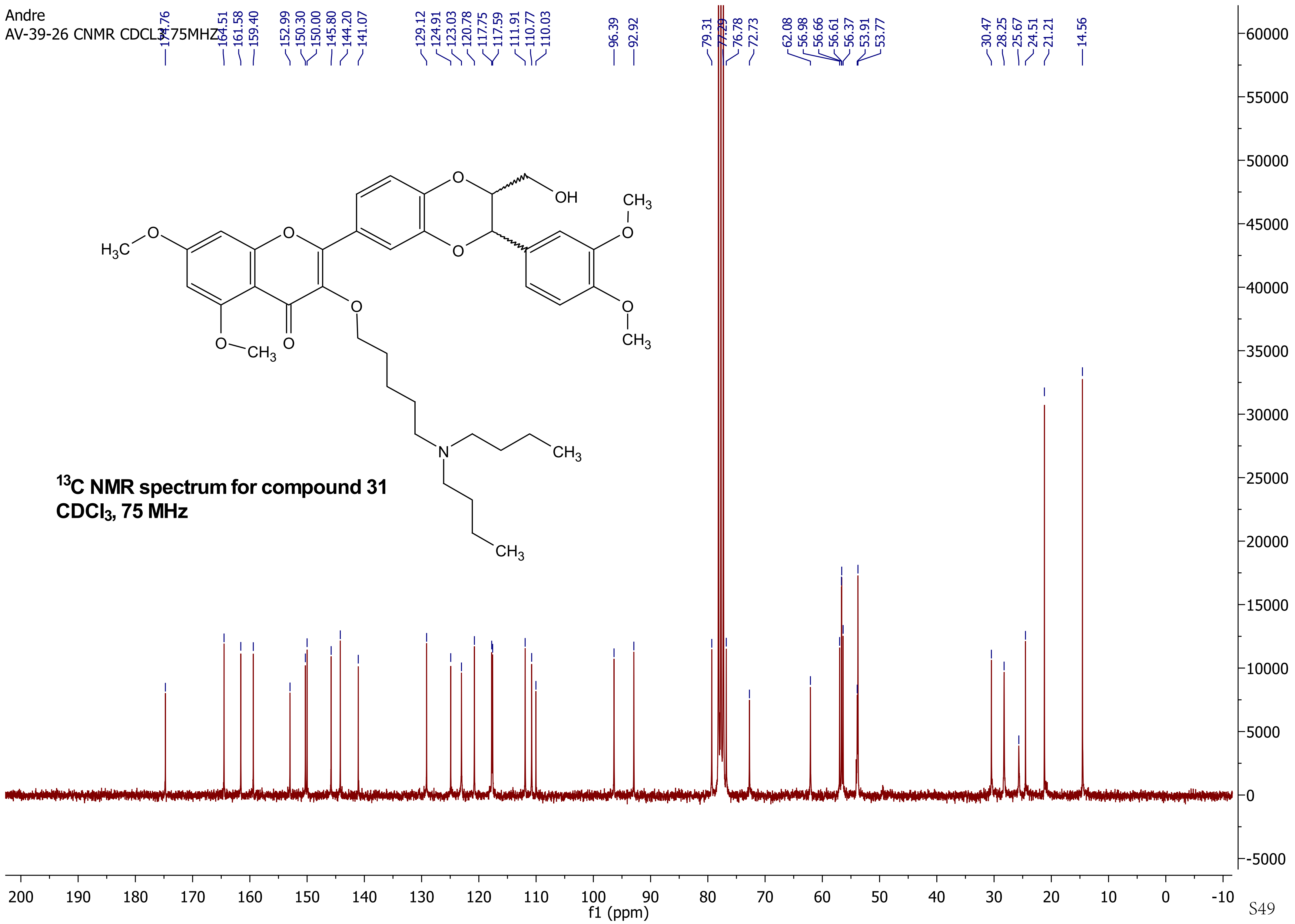
Andre
AV-39-26 CNMR CDCL3 75MHZ
174.76
164.51
161.58
159.40
152.99
150.30
150.00
145.80
144.20
141.07
129.12
124.91
123.03
120.78
117.75
117.59
111.91
110.77
110.03
96.39
92.92
79.31
77.29
76.78
72.73
62.08
56.98
56.66
56.61
56.37
53.91
53.77
30.47
28.25
25.67
24.51
21.21
14.56
OH
CH3
O
H3C
O
CH3
N
CH3
CH3
13C NMR spectrum for compound 31
CDCl3, 75 MHz
f1 (ppm)

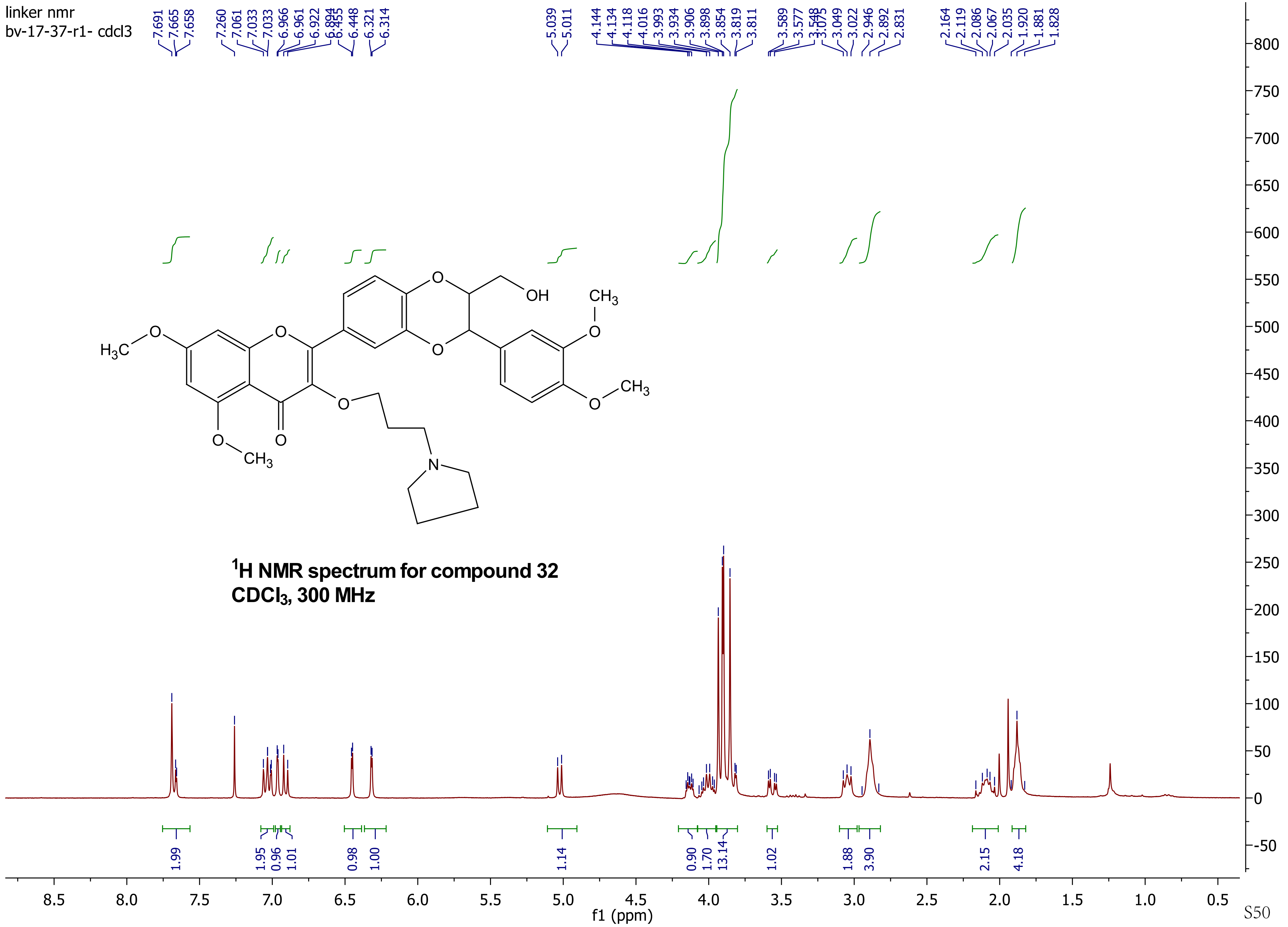
linker nmr
bv-17-37-r1- cdcl3
1H NMR spectrum for compound 32
CDCl3, 300 MHz
f1 (ppm)

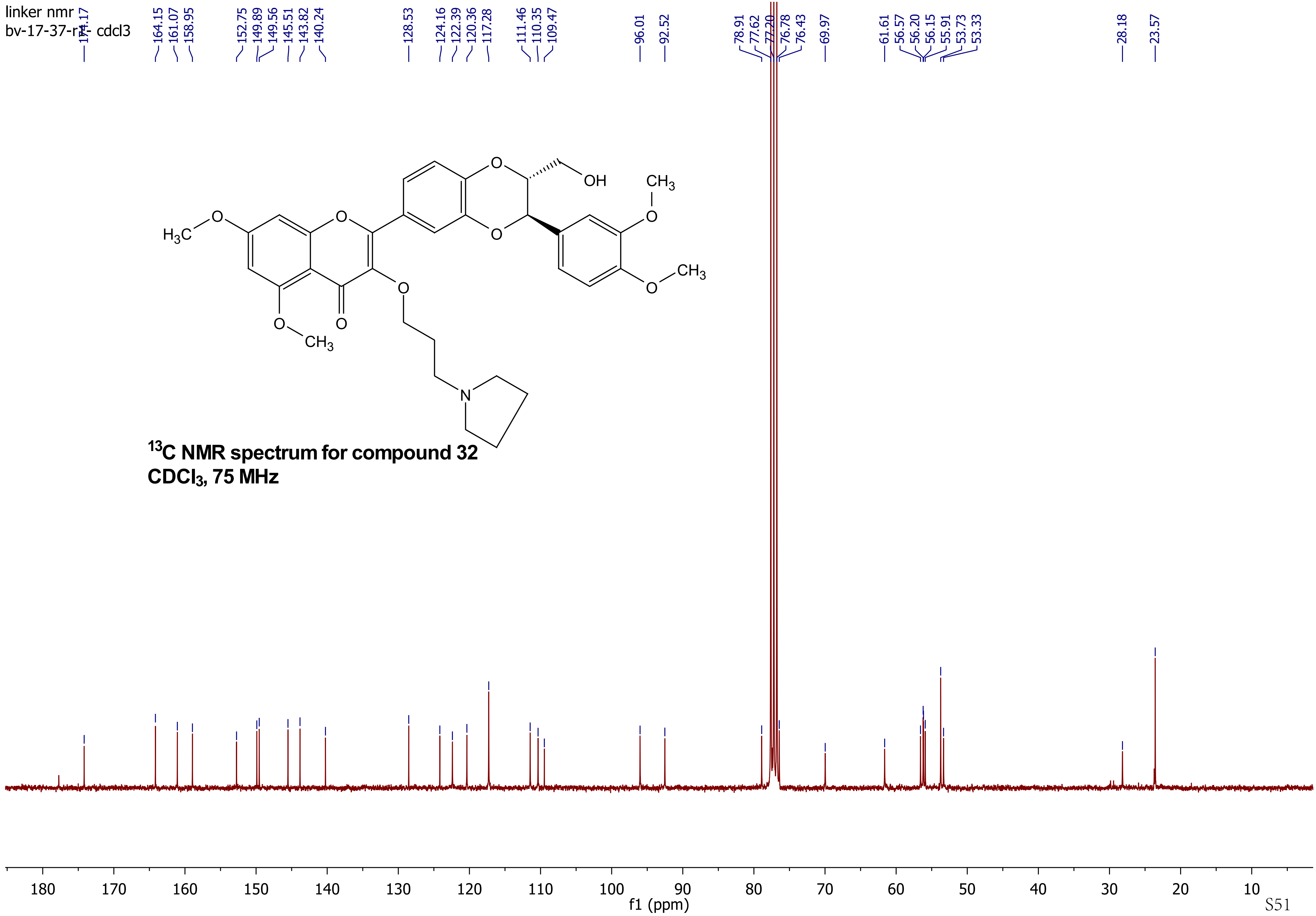

^{13}C NMR spectrum for compound 32
$CDCl_3$, 75 MHz

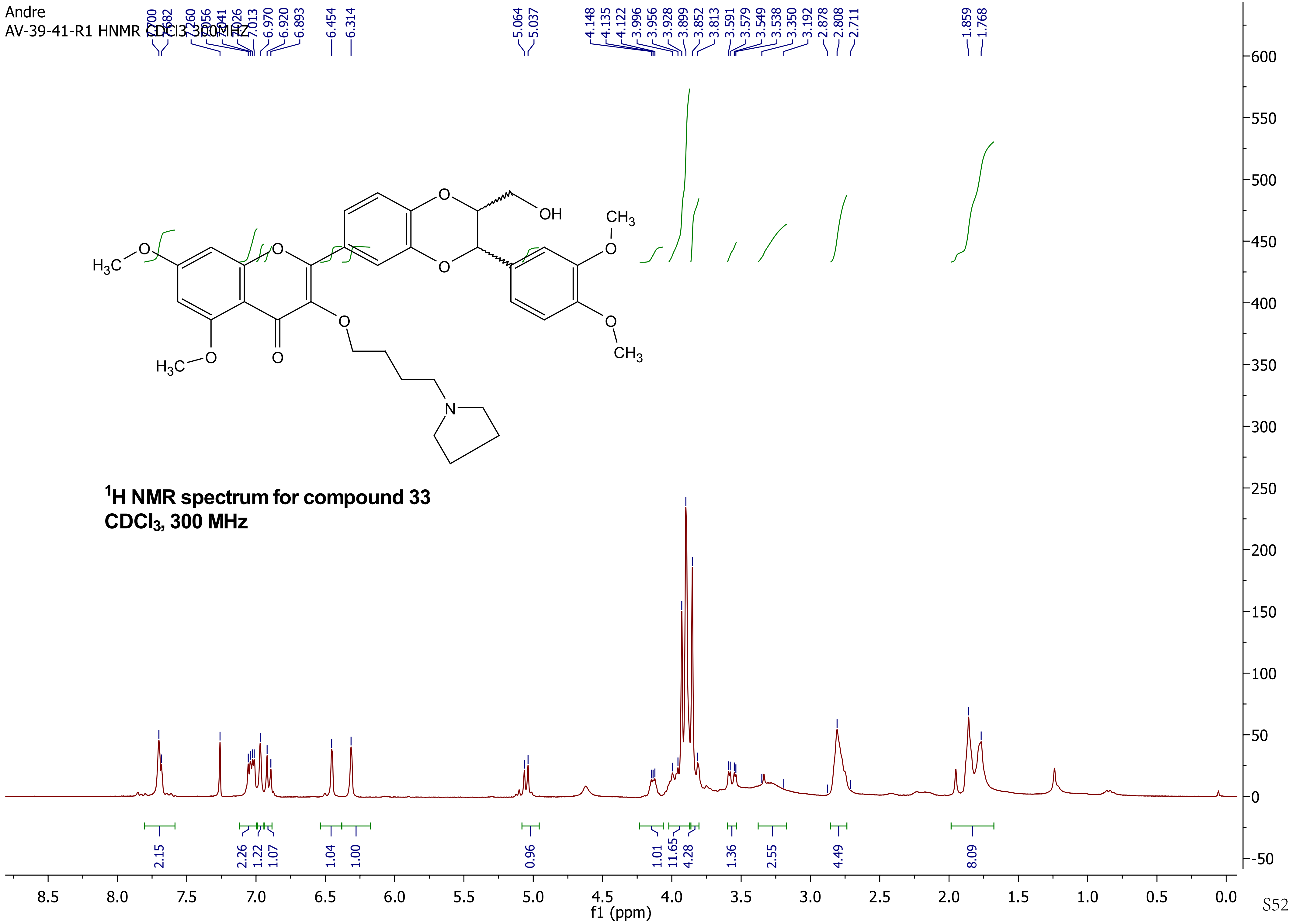
Andre
AV-39-41-R1 HNMR CDCl3 300MHZ
6.970
6.920
6.893
6.454
6.314
5.064
5.037
4.148
4.135
4.122
3.996
3.956
3.928
3.899
3.852
3.813
3.591
3.579
3.549
3.538
3.350
3.192
2.878
2.808
2.711
1.859
1.768
OH
CH_3
O
H_3C
N
^{1}H NMR spectrum for compound 33
$CDCl_3$, 300 MHz
2.15
2.26
1.22
1.07
1.04
1.00
0.96
1.01
11.65
4.28
1.36
2.55
4.49
8.09
f1 (ppm)

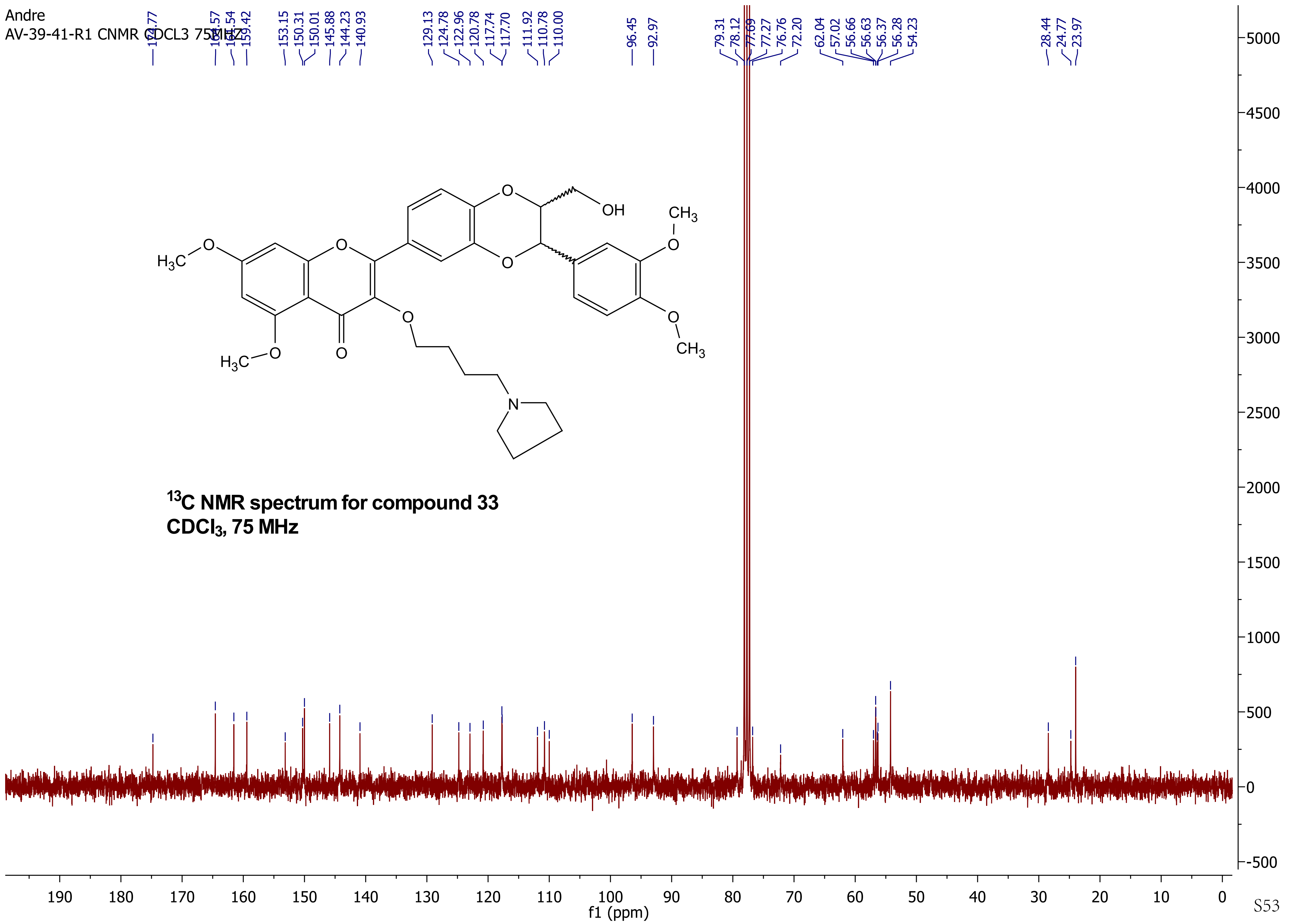

^{13}C NMR spectrum for compound 33
$CDCl_3$, 75 MHz

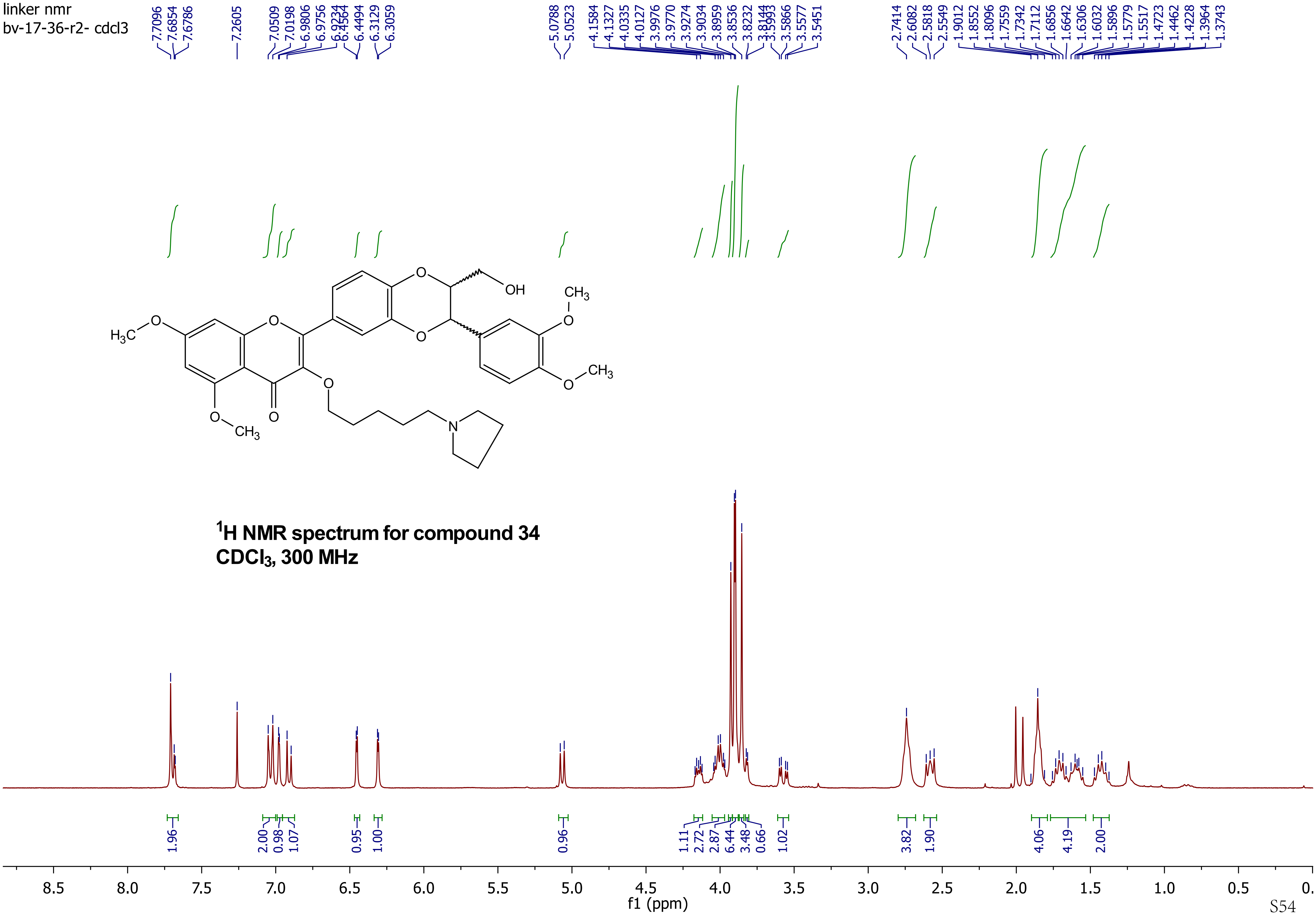

^{1}H NMR spectrum for compound 34
$CDCl_3$, 300 MHz

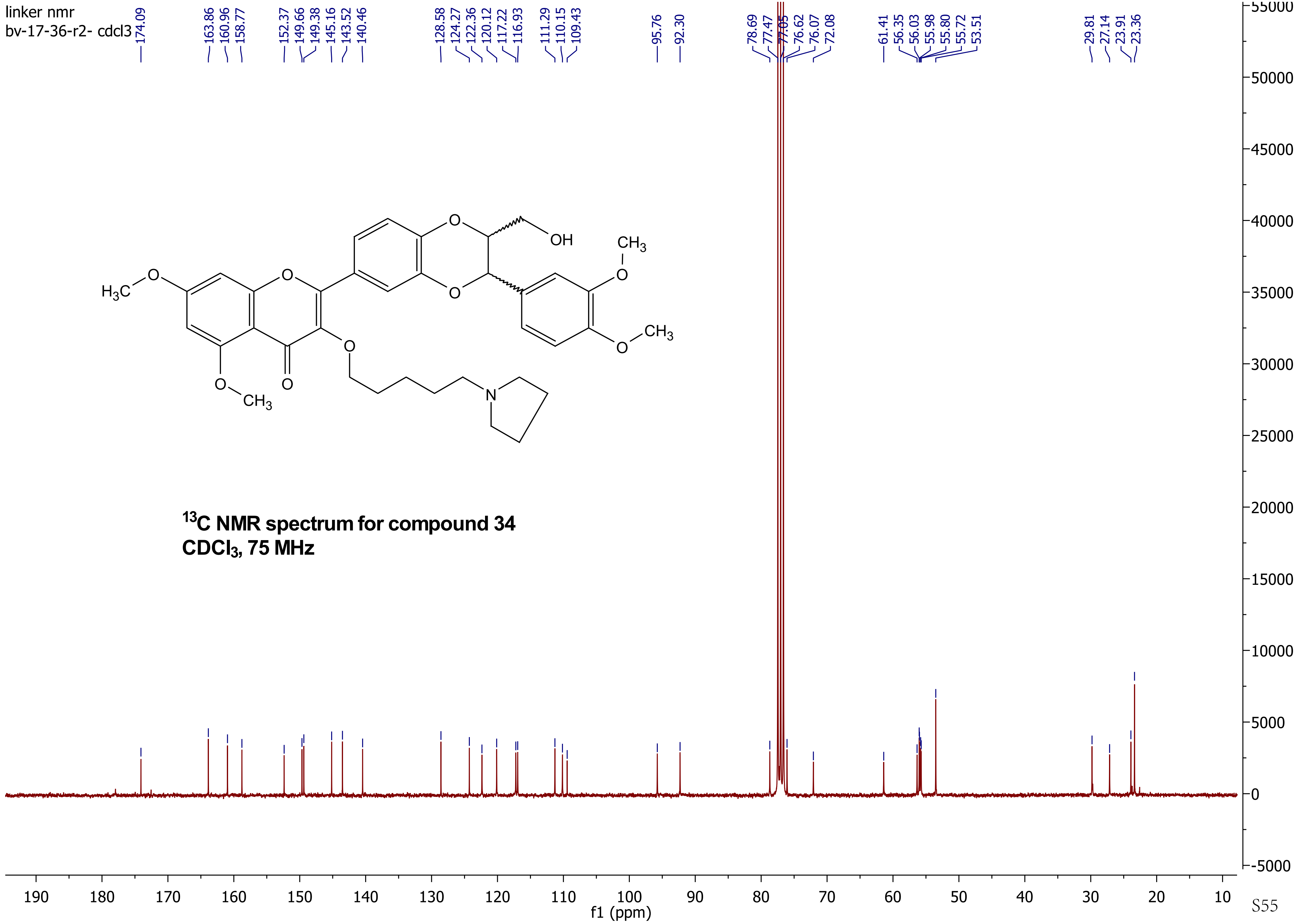

^{13}C NMR spectrum for compound 34
$CDCl_3$, 75 MHz

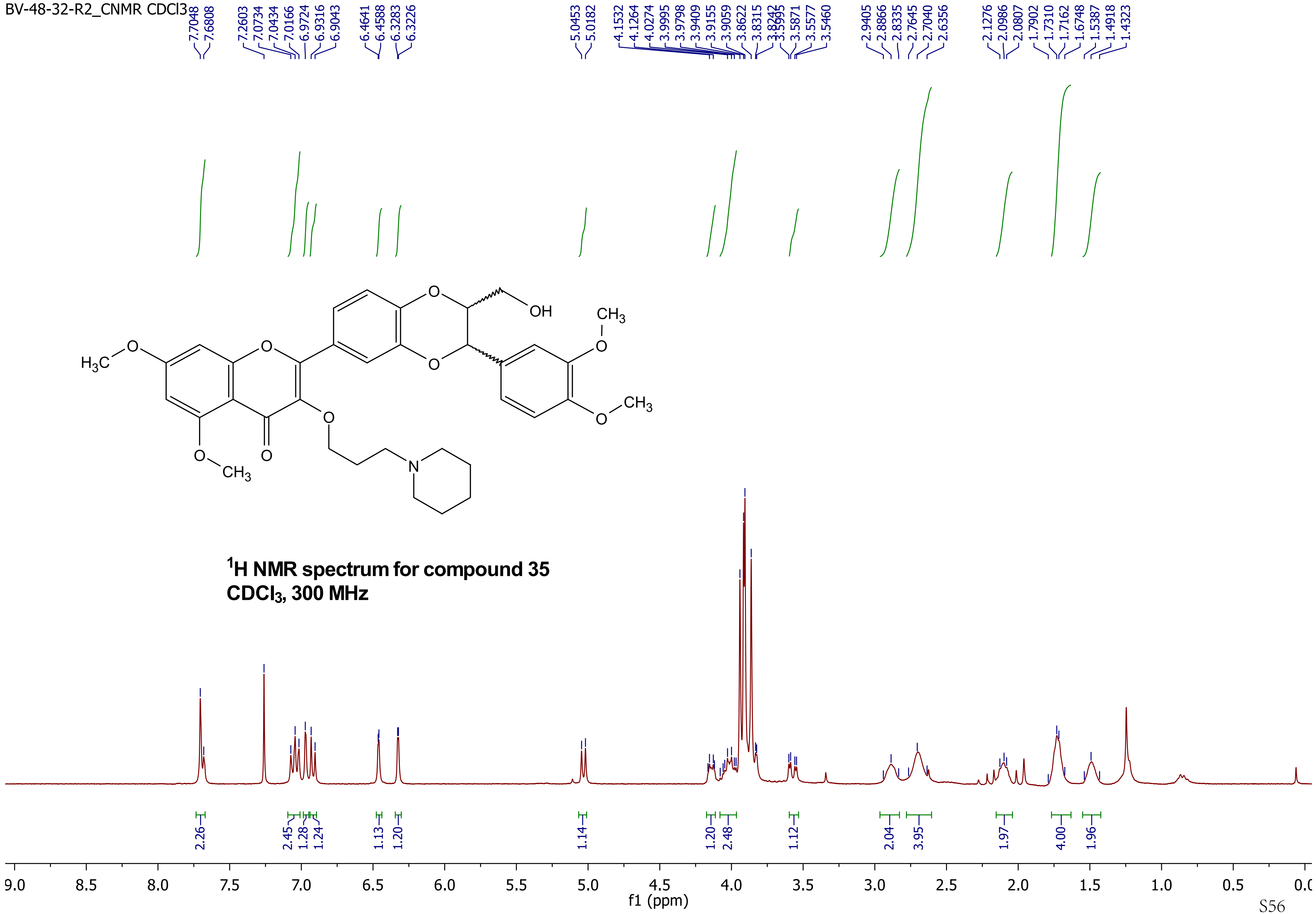
BV-48-32-R2_CNMR CDCl3
7.7048
7.6808
7.2603
7.0734
7.0434
7.0166
6.9724
6.9316
6.9043
6.4641
6.4588
6.3283
6.3226
5.0453
5.0182
4.1532
4.1264
4.0274
3.9995
3.9798
3.9409
3.9155
3.9059
3.8622
3.8315
3.5871
3.5577
3.5460
2.9405
2.8866
2.8335
2.7645
2.7040
2.6356
2.1276
2.0986
2.0807
1.7902
1.7310
1.7162
1.6748
1.5387
1.4918
1.4323
OH
CH3
O
H3C
N
CH3
^{1}H NMR spectrum for compound 35
CDCl3, 300 MHz
2.26
2.45
1.28
1.24
1.13
1.20
1.14
1.20
2.48
1.12
2.04
3.95
1.97
4.00
1.96
f1 (ppm)

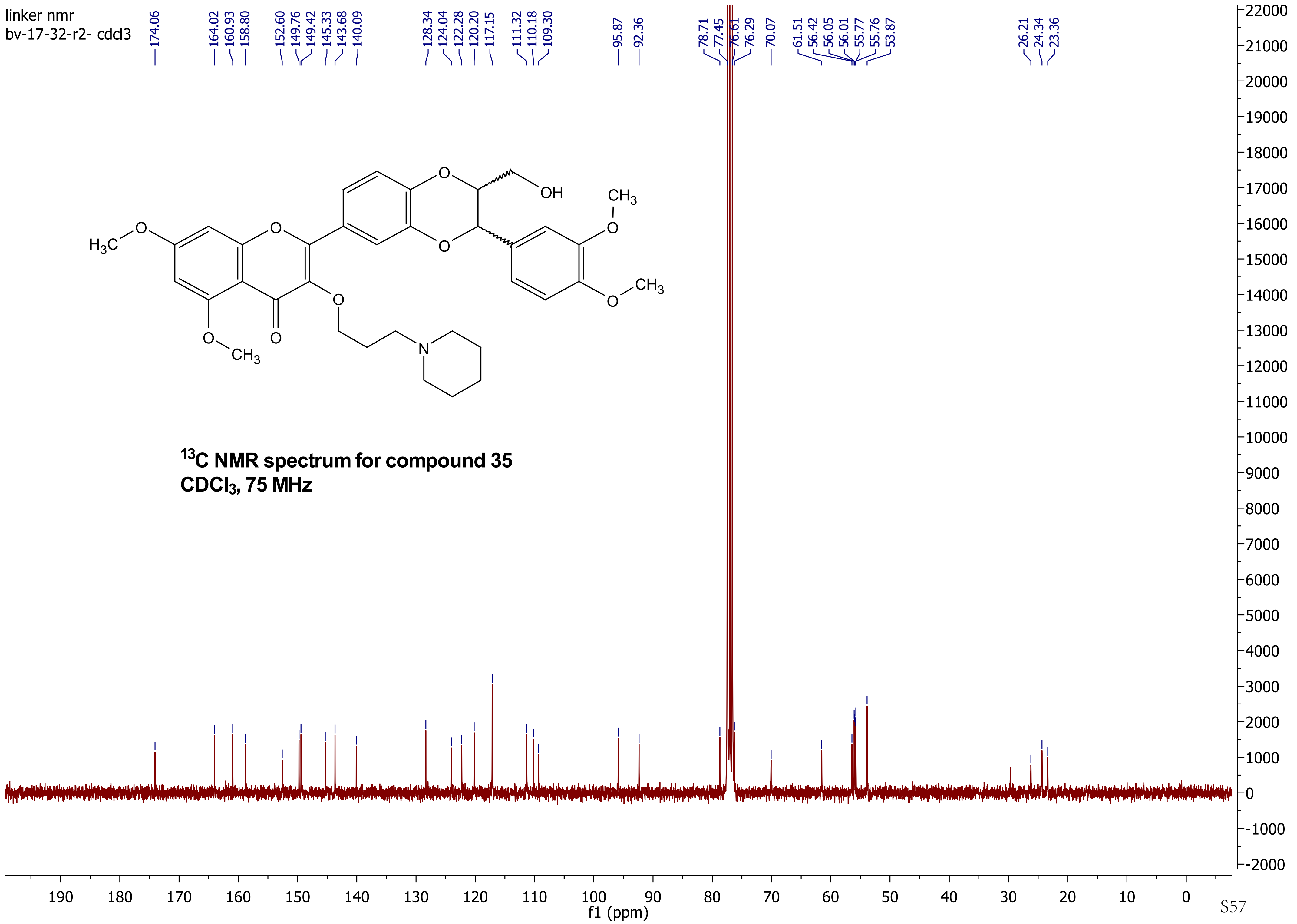

^{13}C NMR spectrum for compound 35
$CDCl_3$, 75 MHz

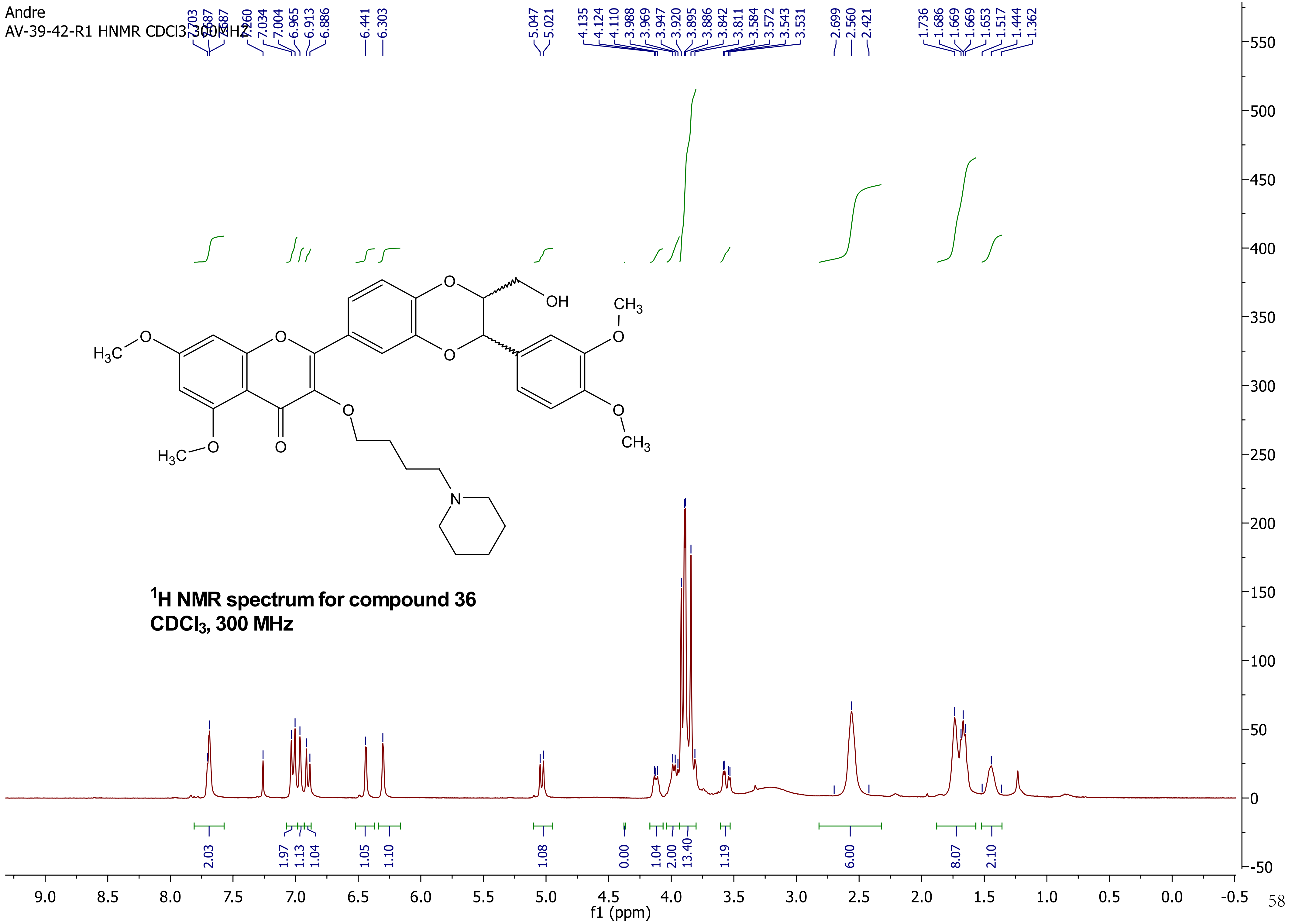

^{1}H NMR spectrum for compound 36
$CDCl_3$, 300 MHz

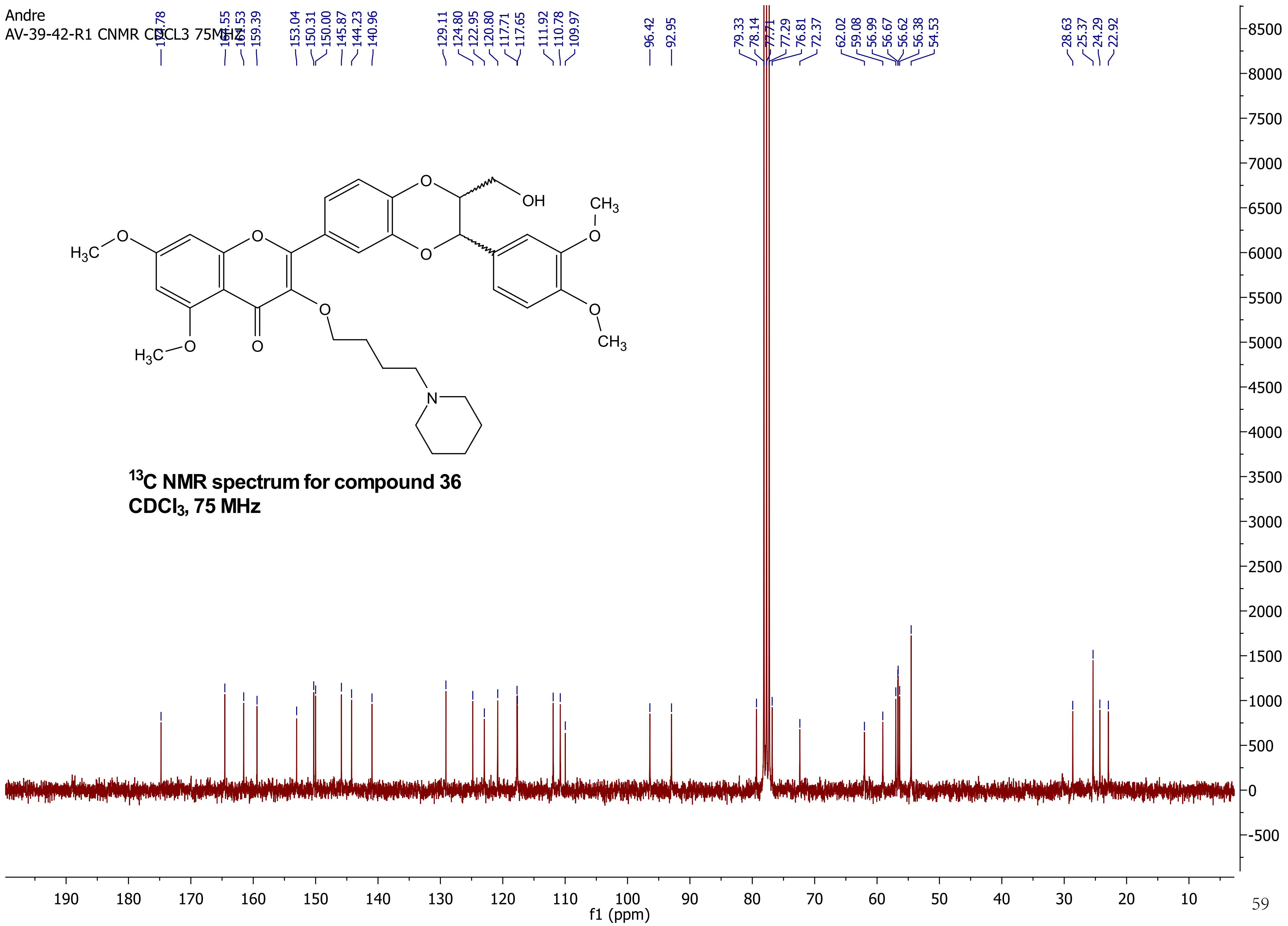

^{13}C NMR spectrum for compound 36
$CDCl_3$, 75 MHz

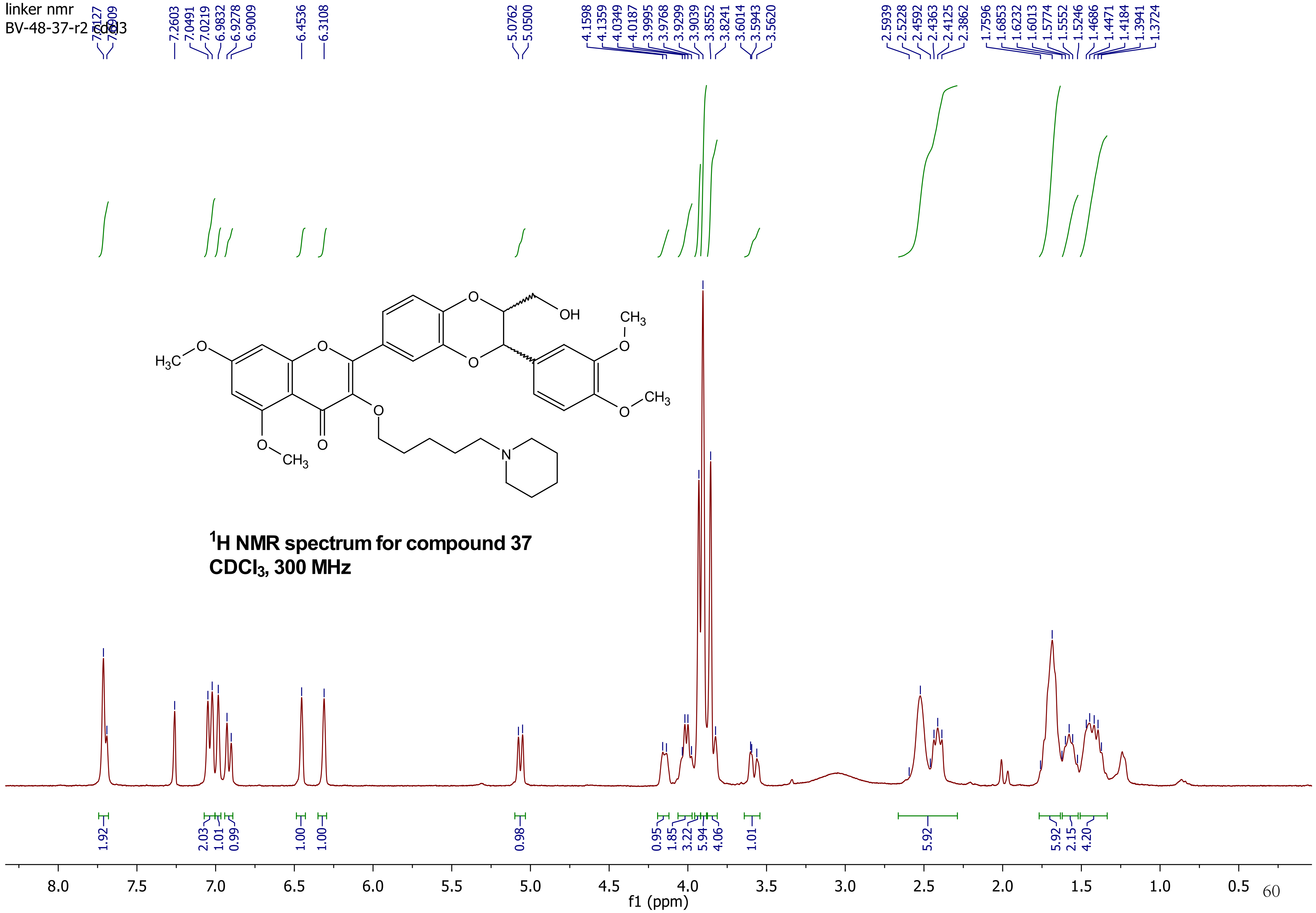

^{1}H NMR spectrum for compound 37
$CDCl_3$, 300 MHz

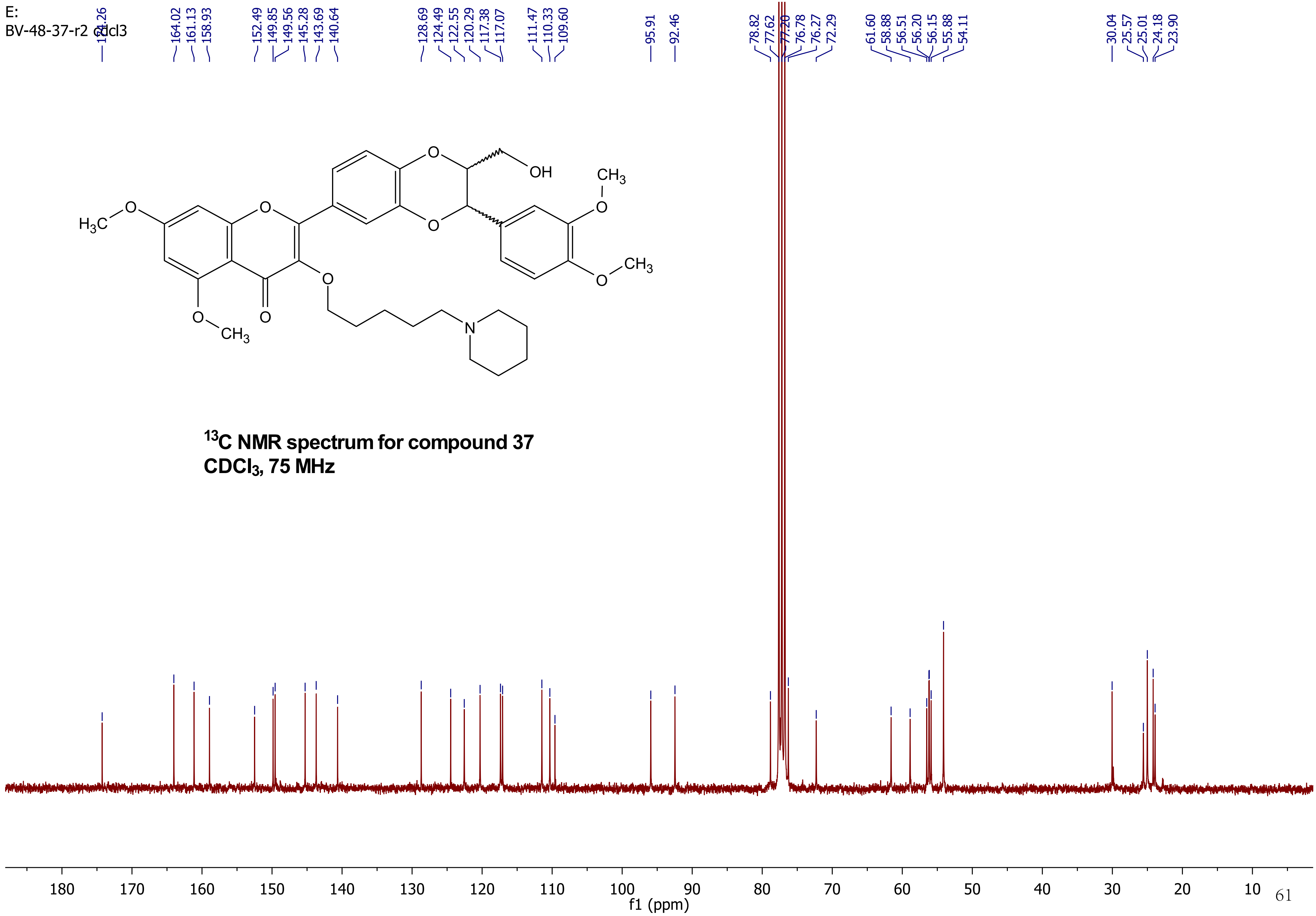

^{13}C NMR spectrum for compound 37
$CDCl_3$, 75 MHz

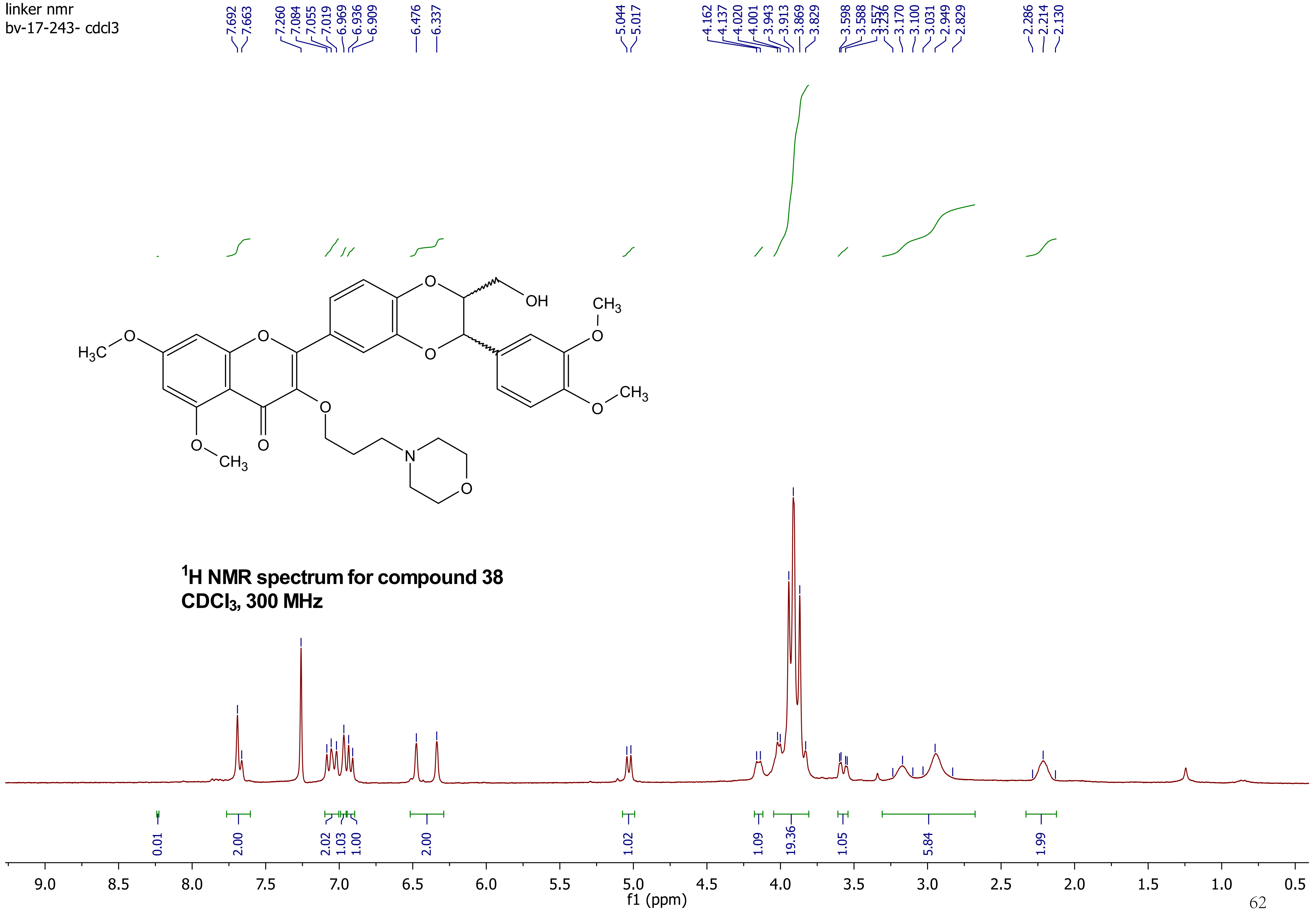

^{1}H NMR spectrum for compound 38
$CDCl_3$, 300 MHz

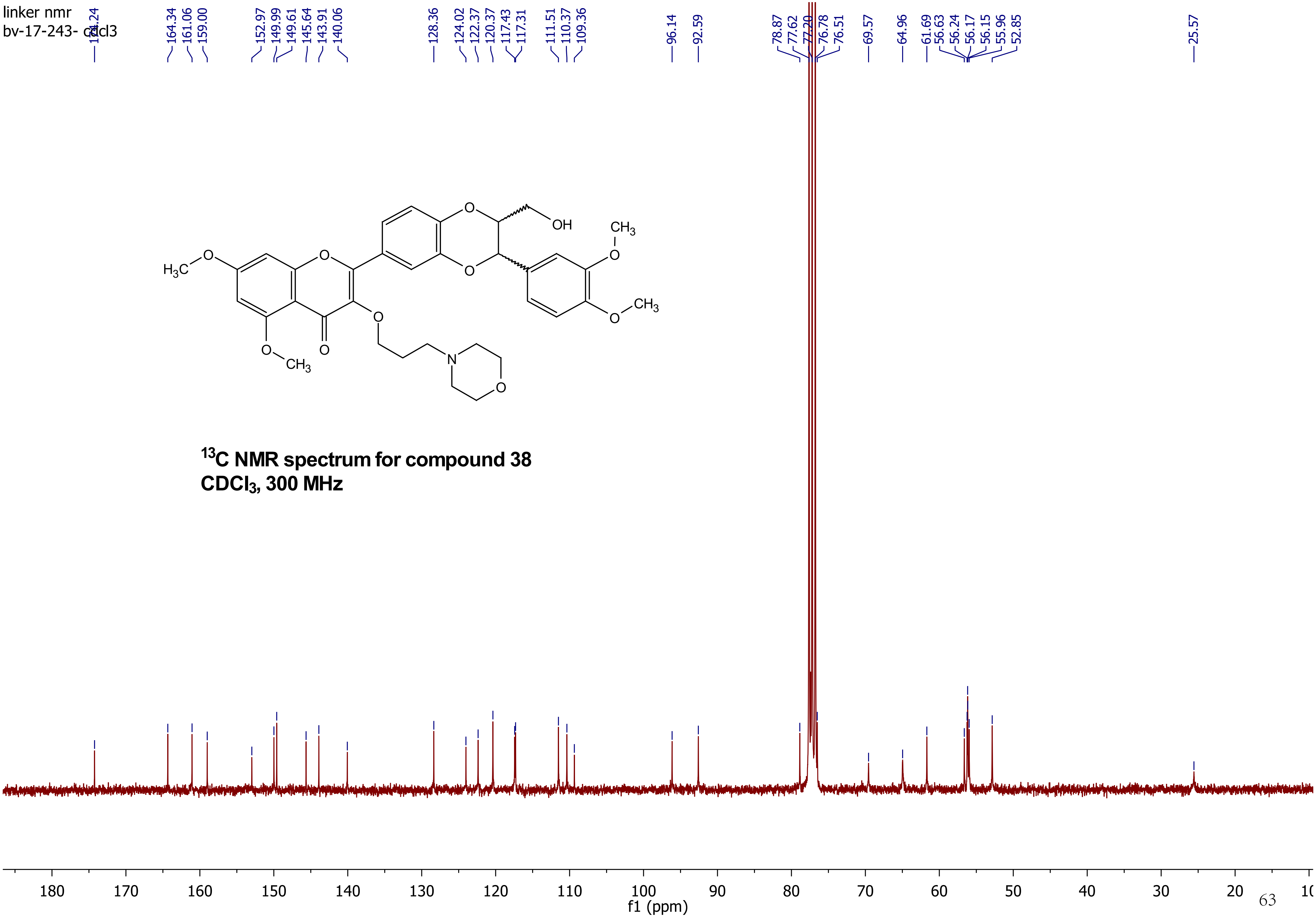

^{13}C NMR spectrum for compound 38
$CDCl_3$, 300 MHz

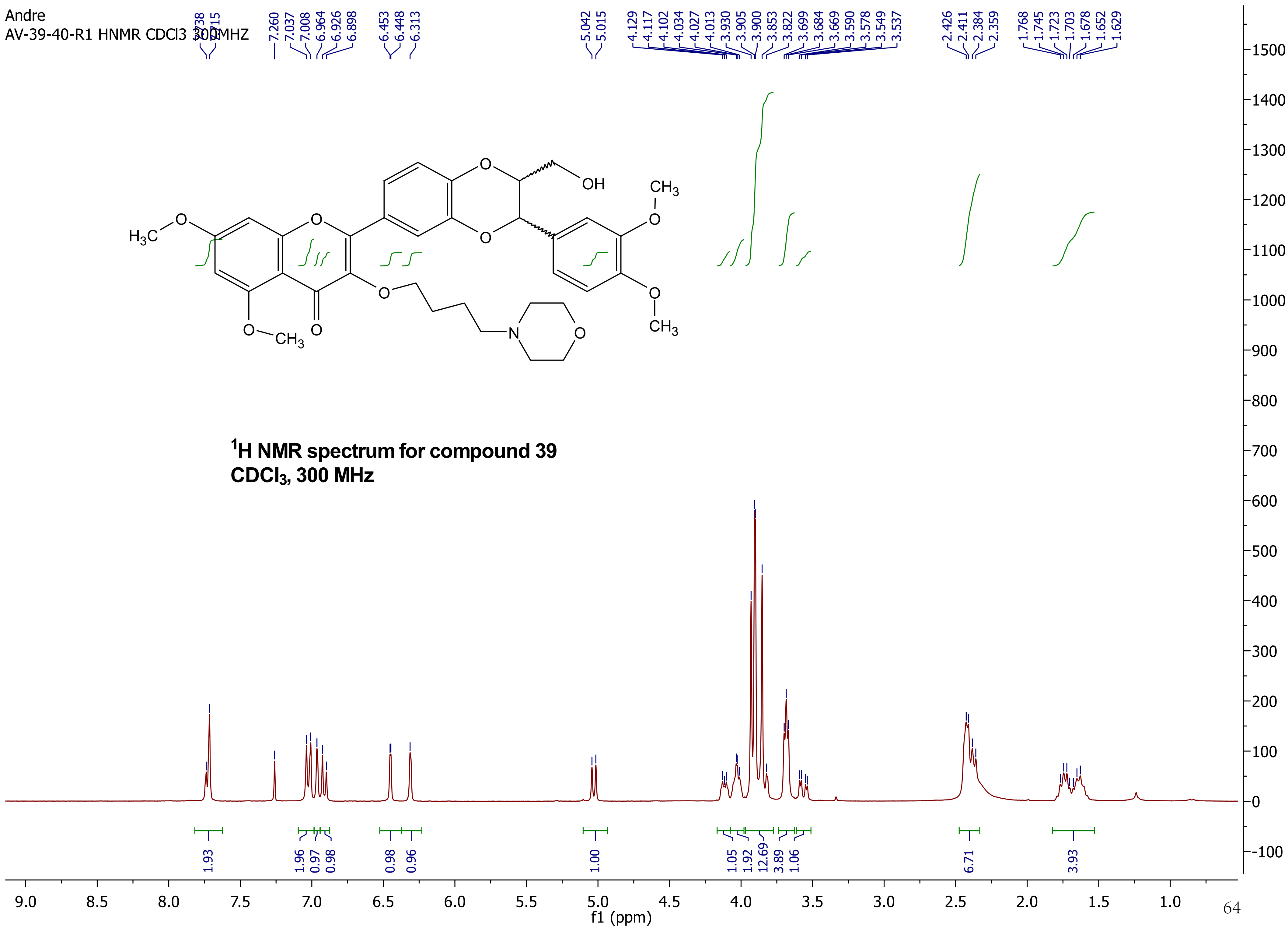
Andre
AV-39-40-R1 HNMR CDCl3 300MHZ
7.738
7.715
7.260
7.037
7.008
6.964
6.926
6.898
6.453
6.448
6.313
5.042
5.015
4.129
4.117
4.102
4.034
4.027
4.013
3.930
3.905
3.900
3.853
3.822
3.699
3.684
3.669
3.590
3.578
3.549
3.537
2.426
2.411
2.384
2.359
1.768
1.745
1.723
1.703
1.678
1.652
1.629
H3C
O
O
O
O
OH
CH3
O
O
O
CH3
O
CH3
O
O
N
O
1H NMR spectrum for compound 39
CDCl3, 300 MHz
1.93
1.96
0.97
0.98
0.98
0.96
1.00
1.05
1.92
12.69
3.89
1.06
6.71
3.93
f1 (ppm)

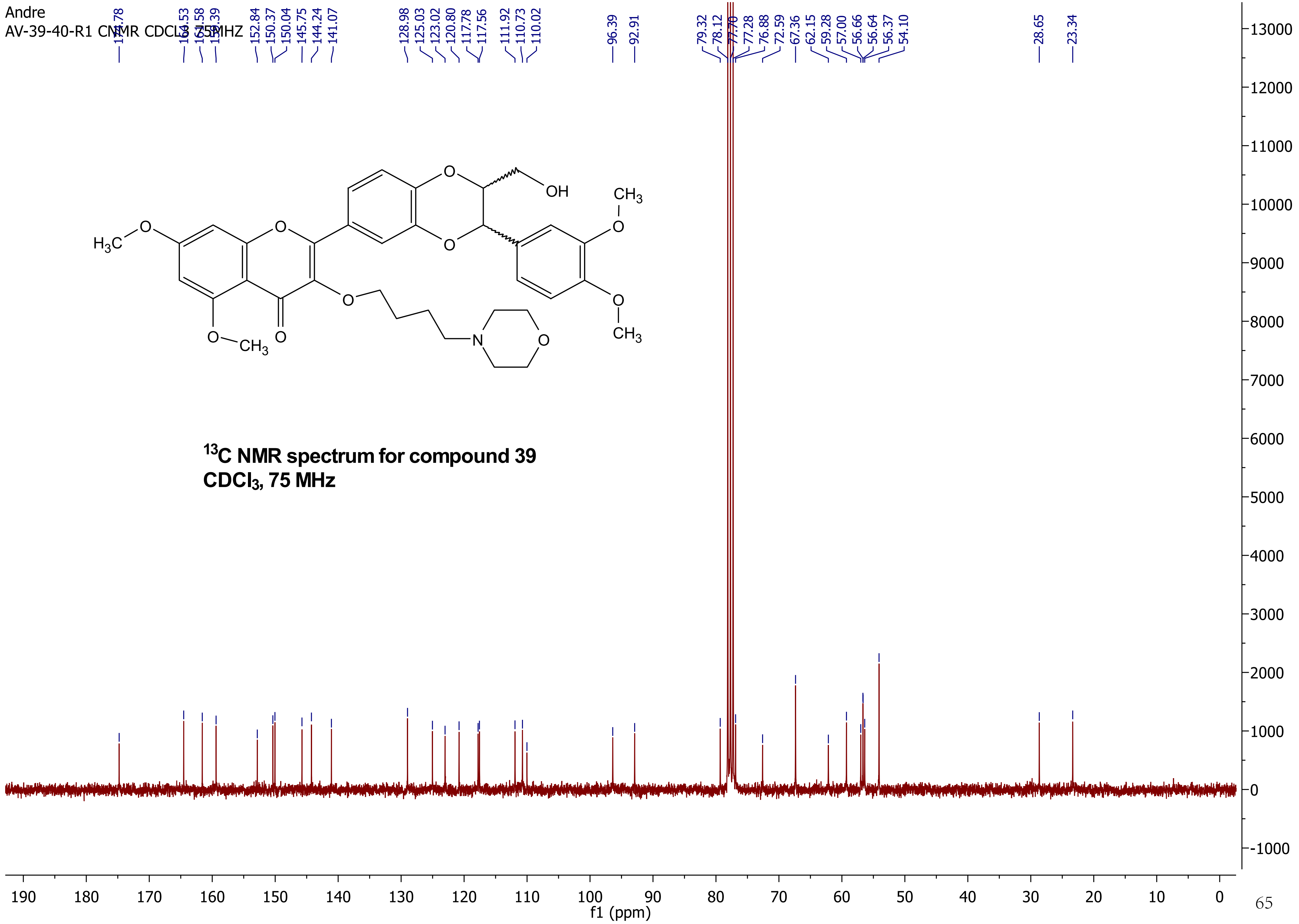

^{13}C NMR spectrum for compound 39
$CDCl_3$, 75 MHz

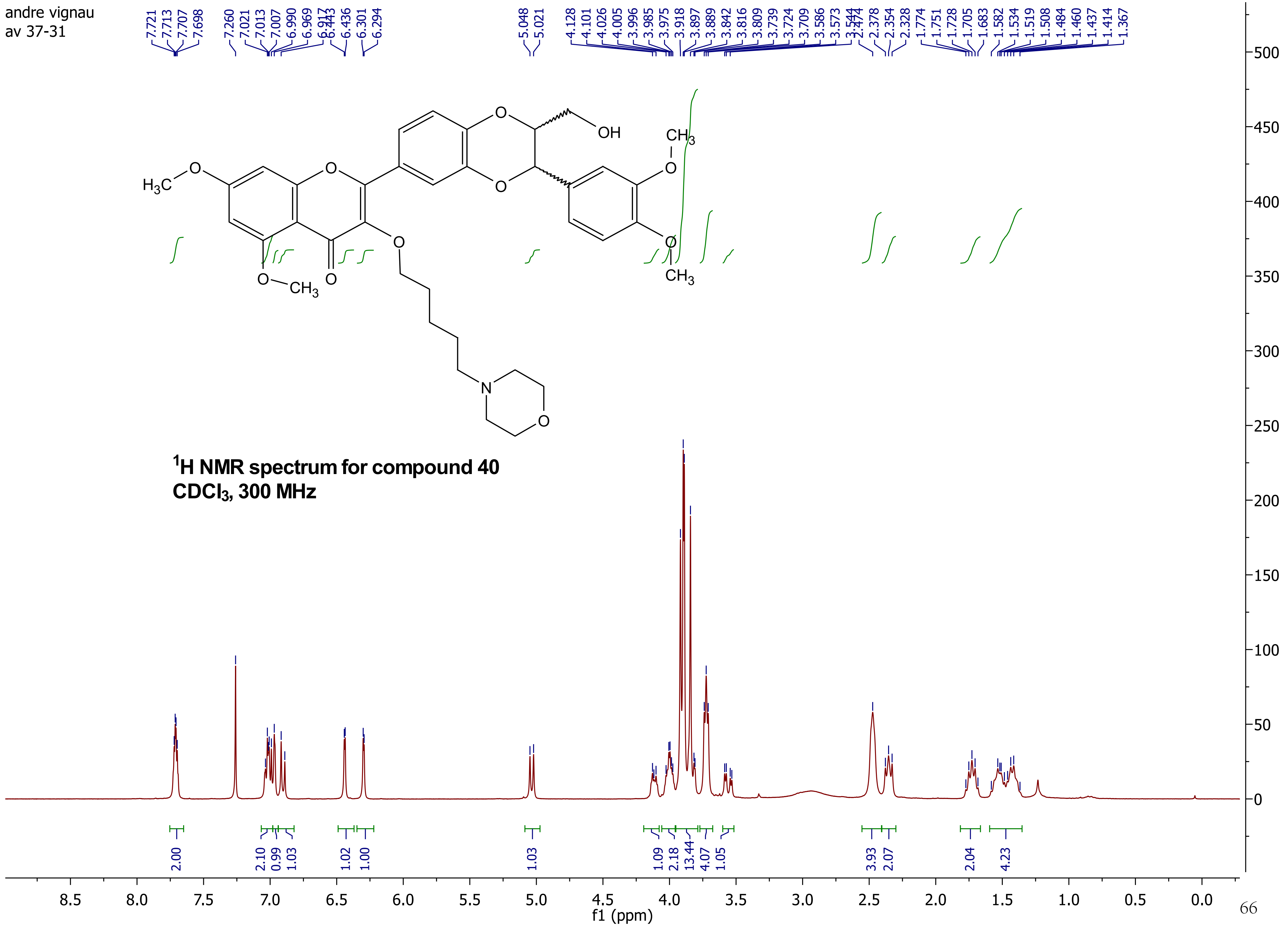
andre vignau
av 37-31
1H NMR spectrum for compound 40
CDCl3, 300 MHz
f1 (ppm)

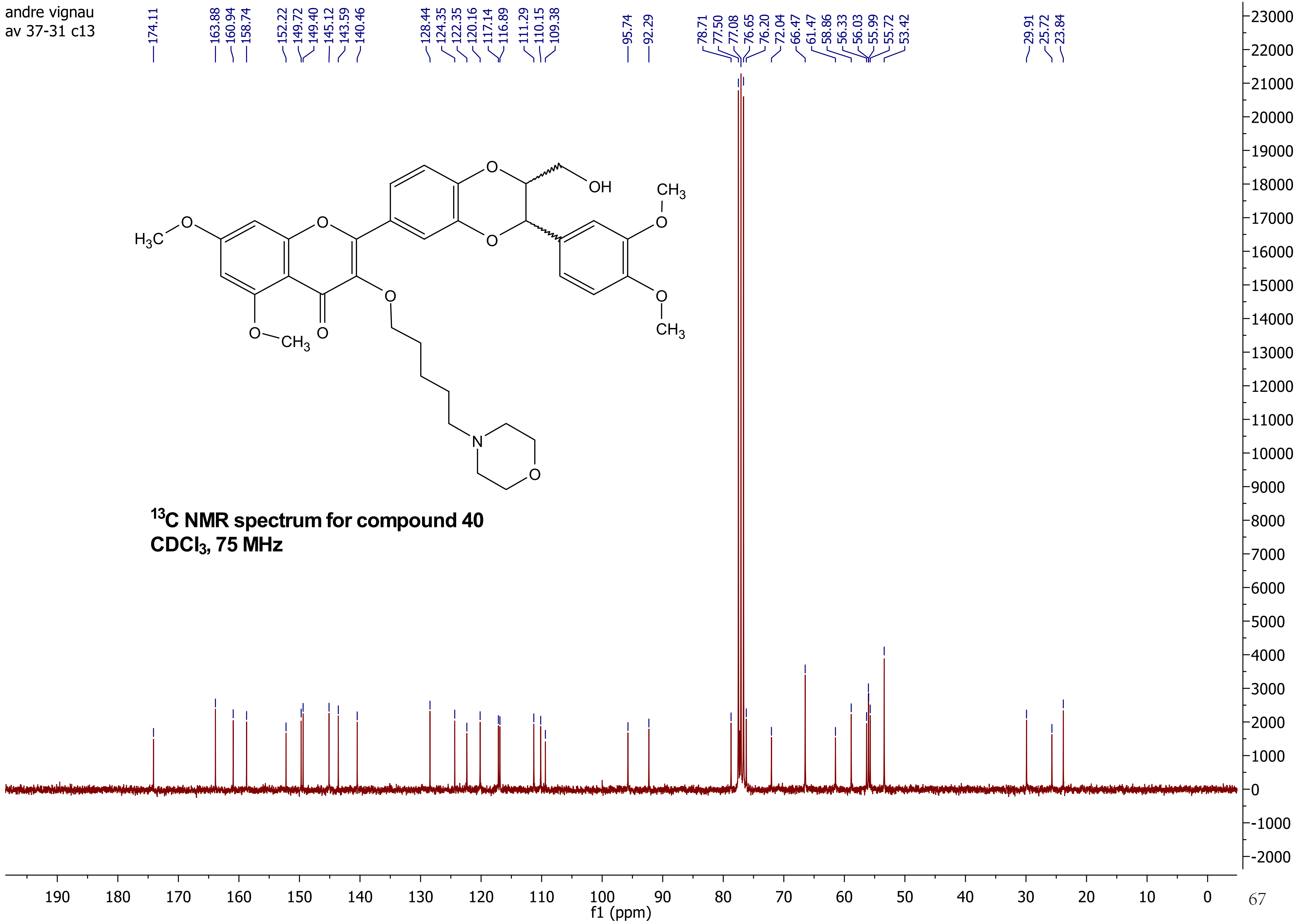

^{13}C NMR spectrum for compound 40
$CDCl_3$, 75 MHz

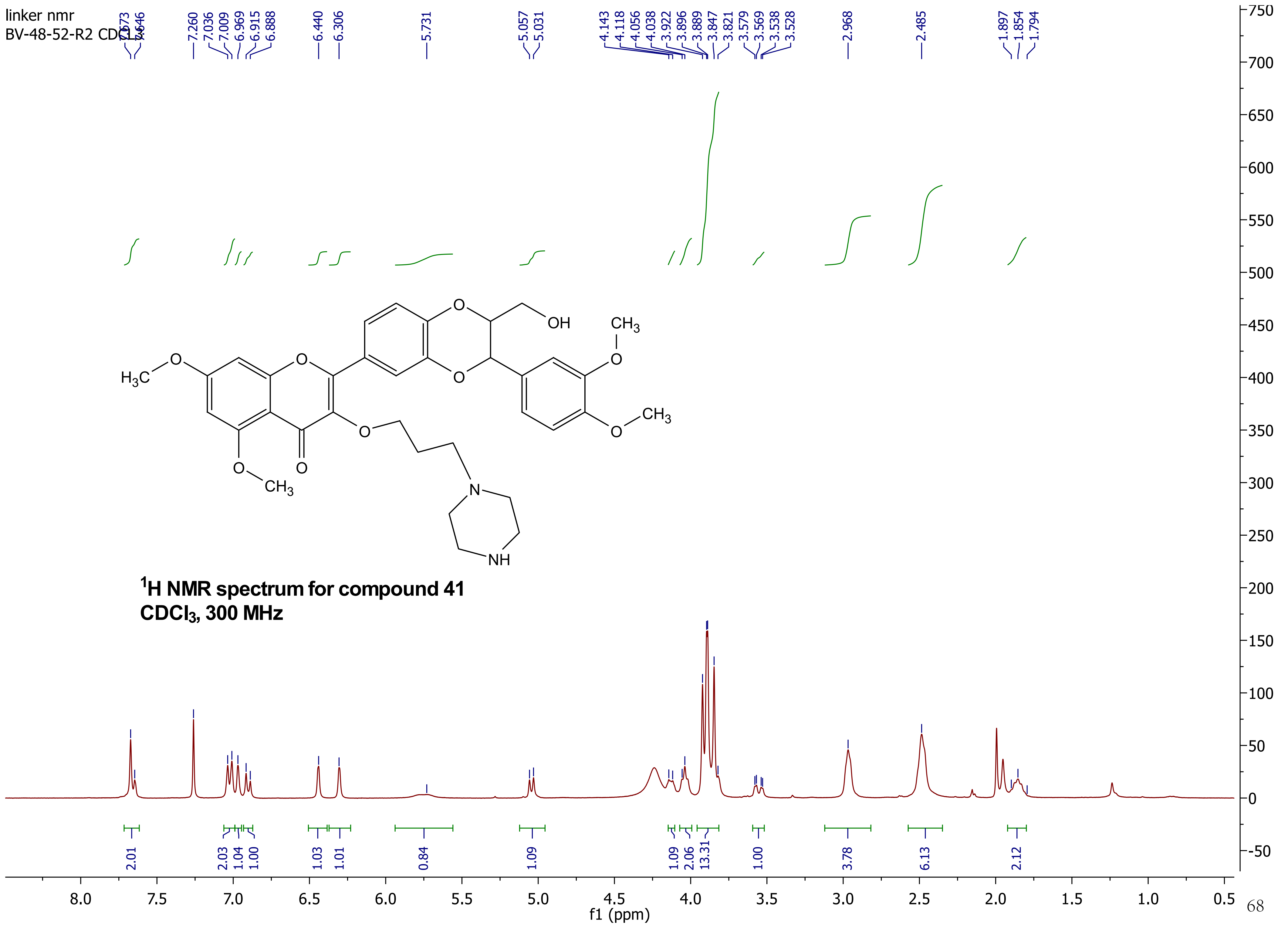

^{1}H NMR spectrum for compound 41
$CDCl_3$, 300 MHz

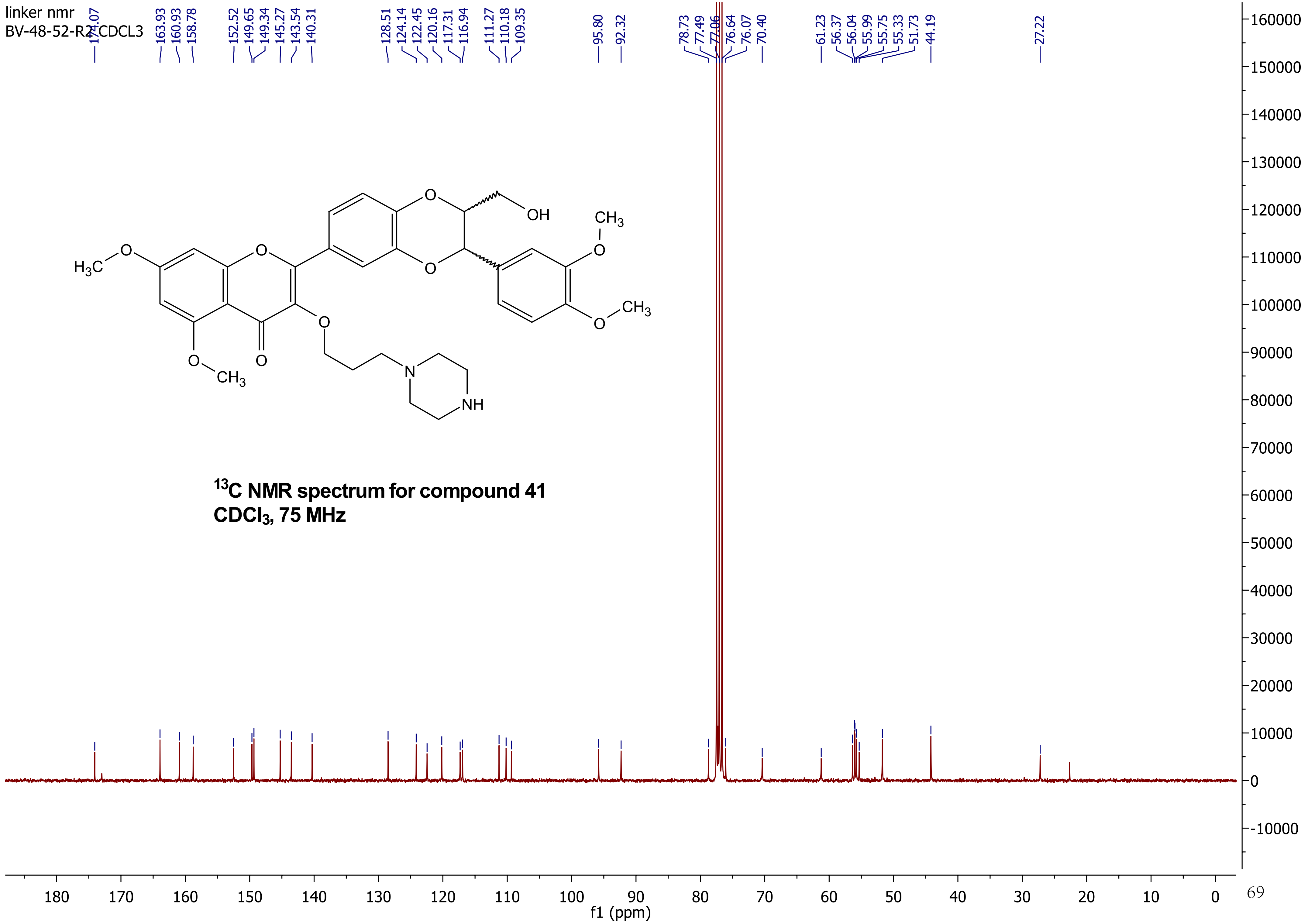

^{13}C NMR spectrum for compound 41
$CDCl_3$, 75 MHz

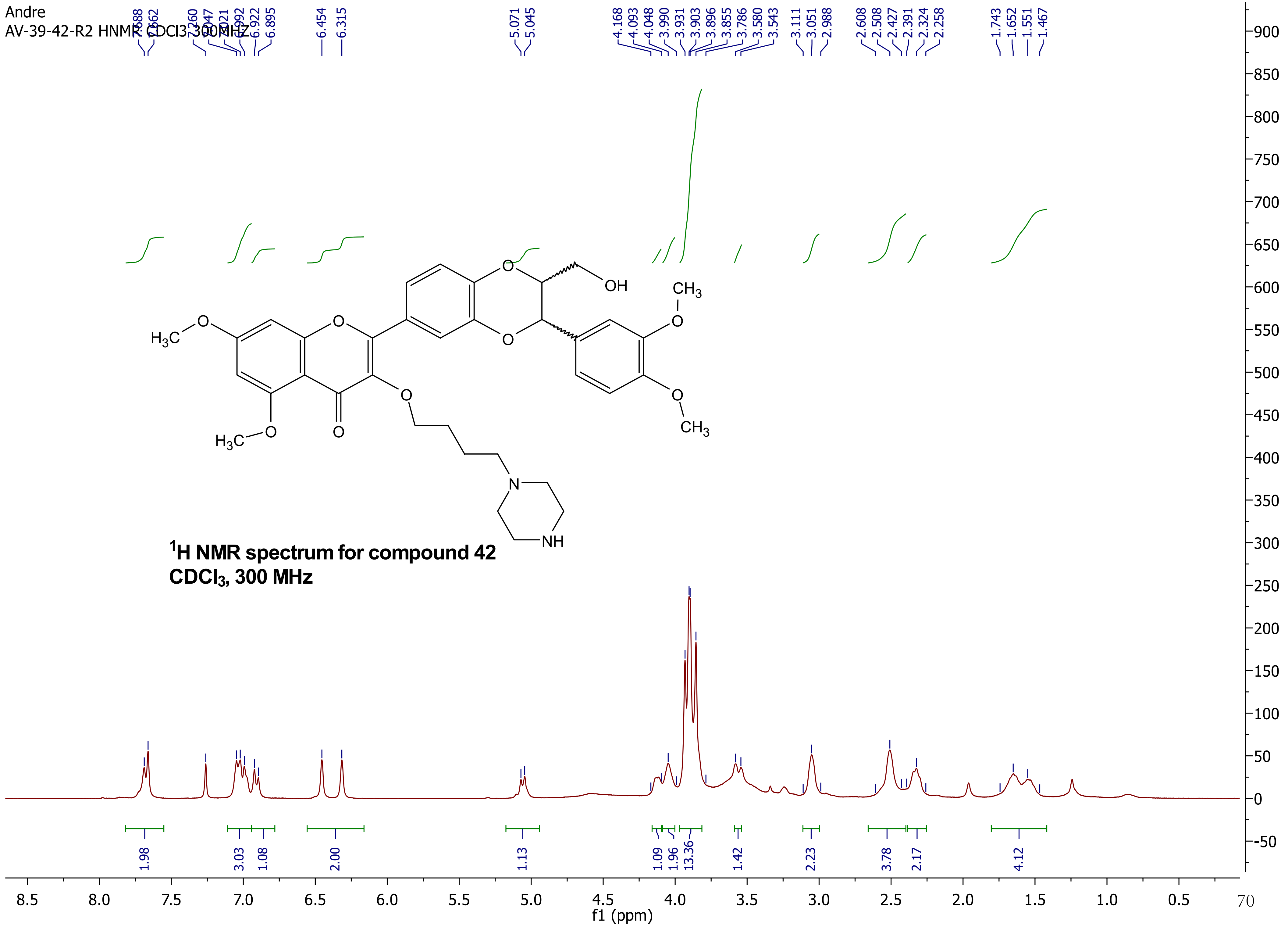
Andre
AV-39-42-R2 HNMR CDCl3, 300MHZ
7.688
7.662
7.260
7.047
7.021
6.992
6.922
6.895
6.454
6.315
5.071
5.045
4.168
4.093
4.048
3.990
3.931
3.903
3.896
3.855
3.786
3.580
3.543
3.111
3.051
2.988
2.608
2.508
2.427
2.391
2.324
2.258
1.743
1.652
1.551
1.467
H3C
O
O
O
O
OH
CH3
O
O
CH3
H3C
O
O
O
N
NH
1H NMR spectrum for compound 42
CDCl3, 300 MHz
1.98
3.03
1.08
2.00
1.13
1.09
1.96
13.36
1.42
2.23
3.78
2.17
4.12
f1 (ppm)

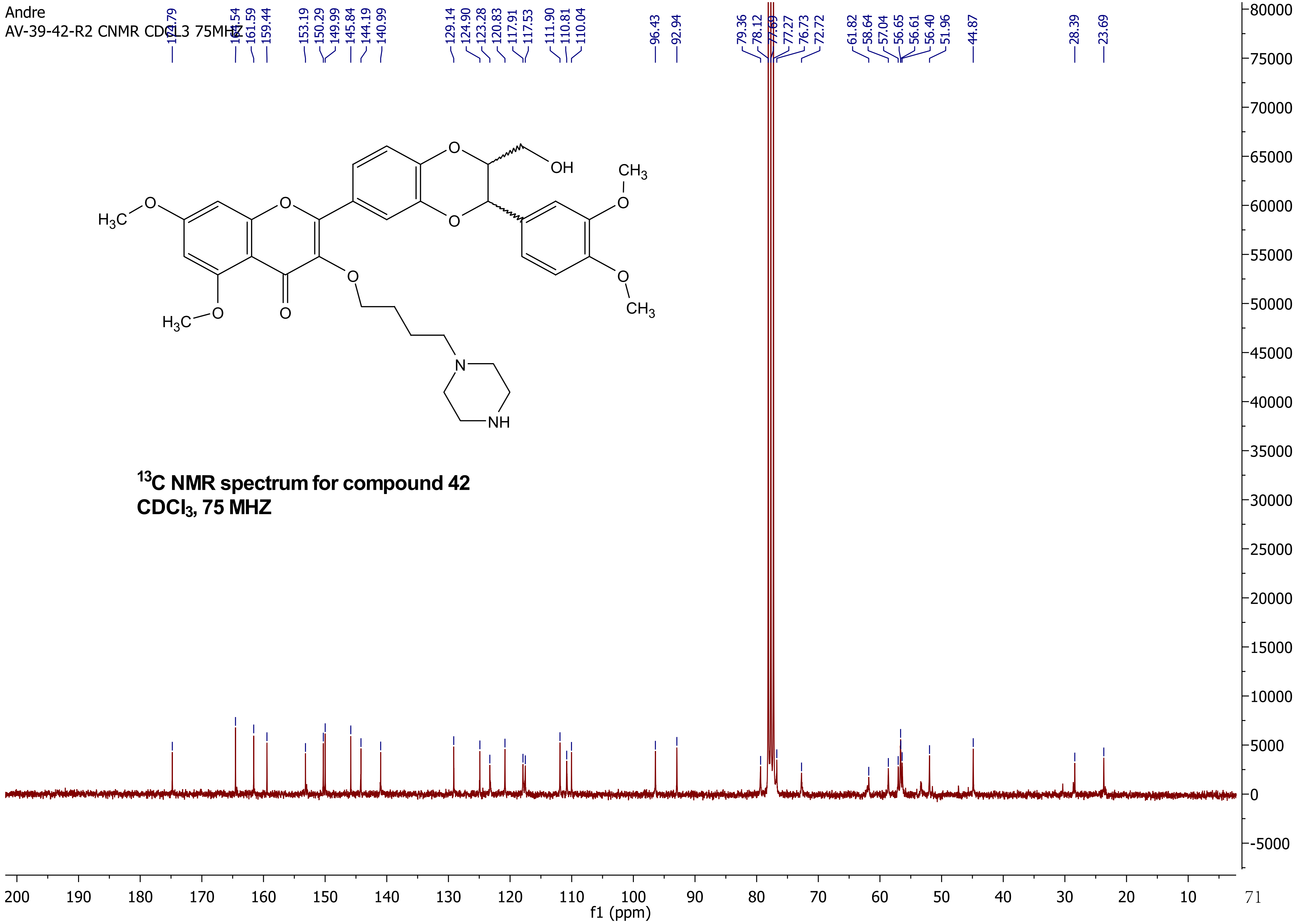

^{13}C NMR spectrum for compound 42
$CDCl_3$, 75 MHZ

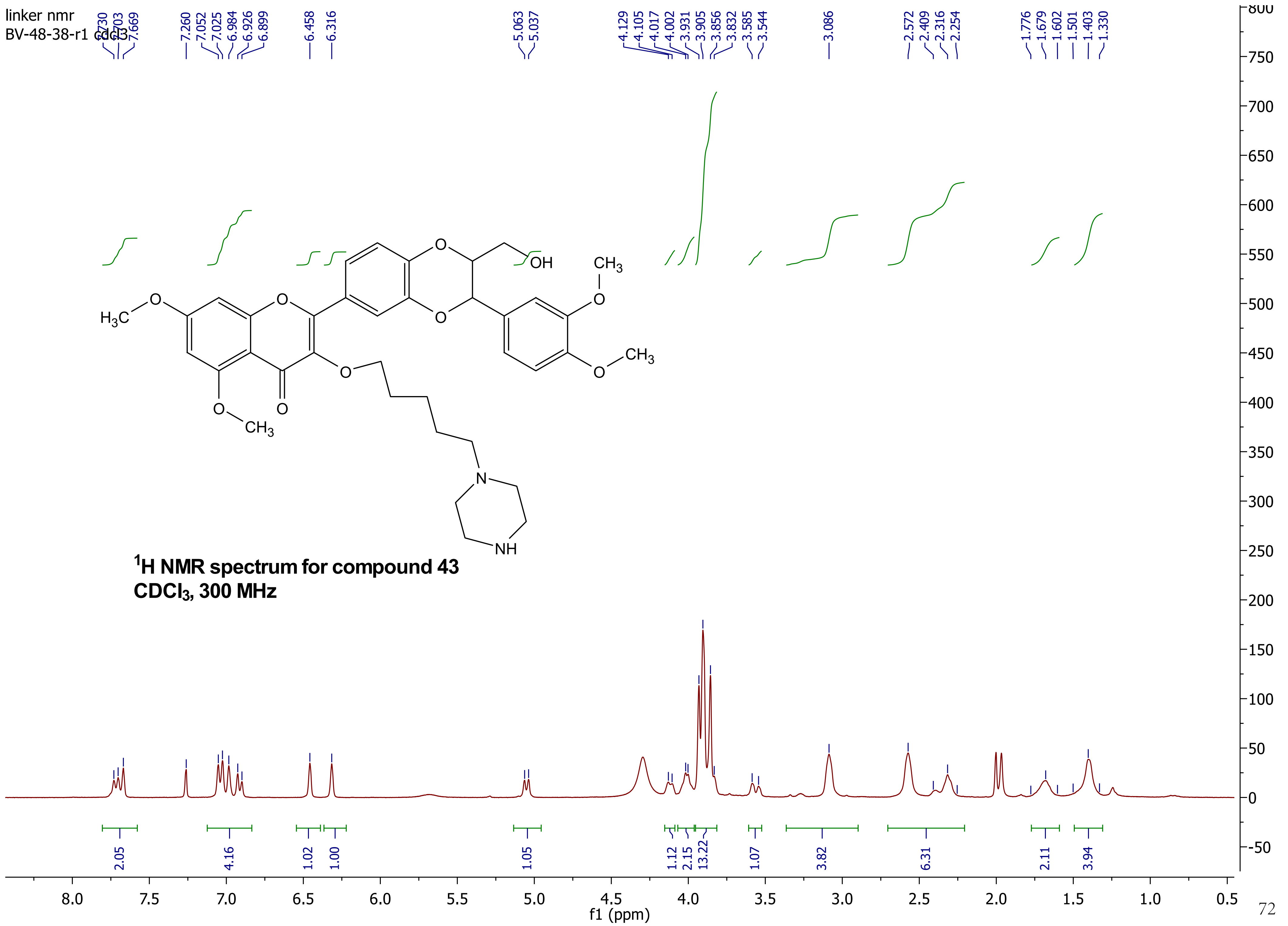

^{1}H NMR spectrum for compound 43
$CDCl_3$, 300 MHz

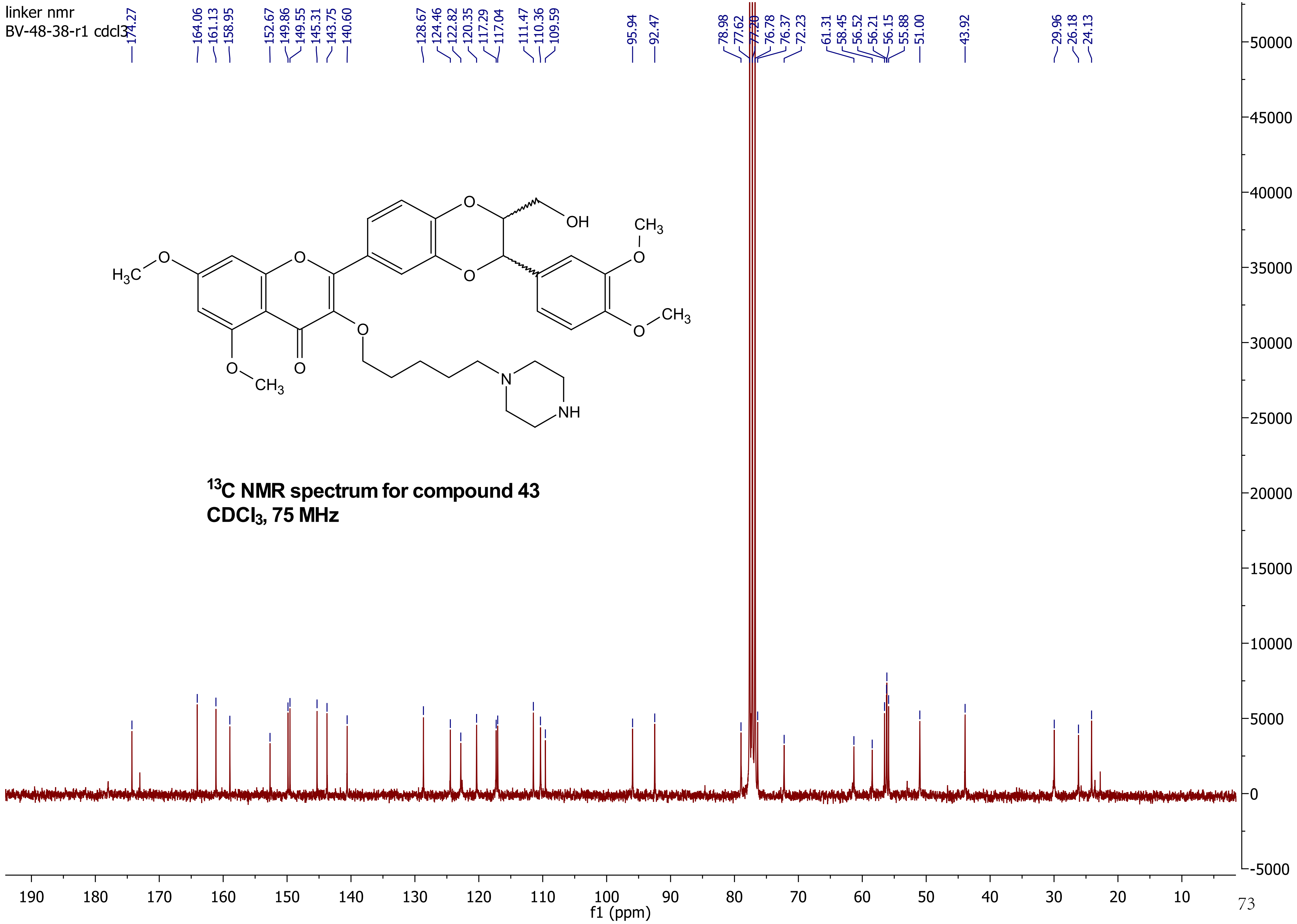
linker nmr
BV-48-38-r1 cdcl3
174.27
164.06
161.13
158.95
152.67
149.86
149.55
145.31
143.75
140.60
128.67
124.46
122.82
120.35
117.29
117.04
111.47
110.36
109.59
95.94
92.47
78.98
77.62
77.20
76.78
76.37
72.23
61.31
58.45
56.52
56.21
56.15
55.88
51.00
43.92
29.96
26.18
24.13
H_3C
O
OH
CH_3
NH
N
^{13}C NMR spectrum for compound 43
$CDCl_3$, 75 MHz
f1 (ppm)

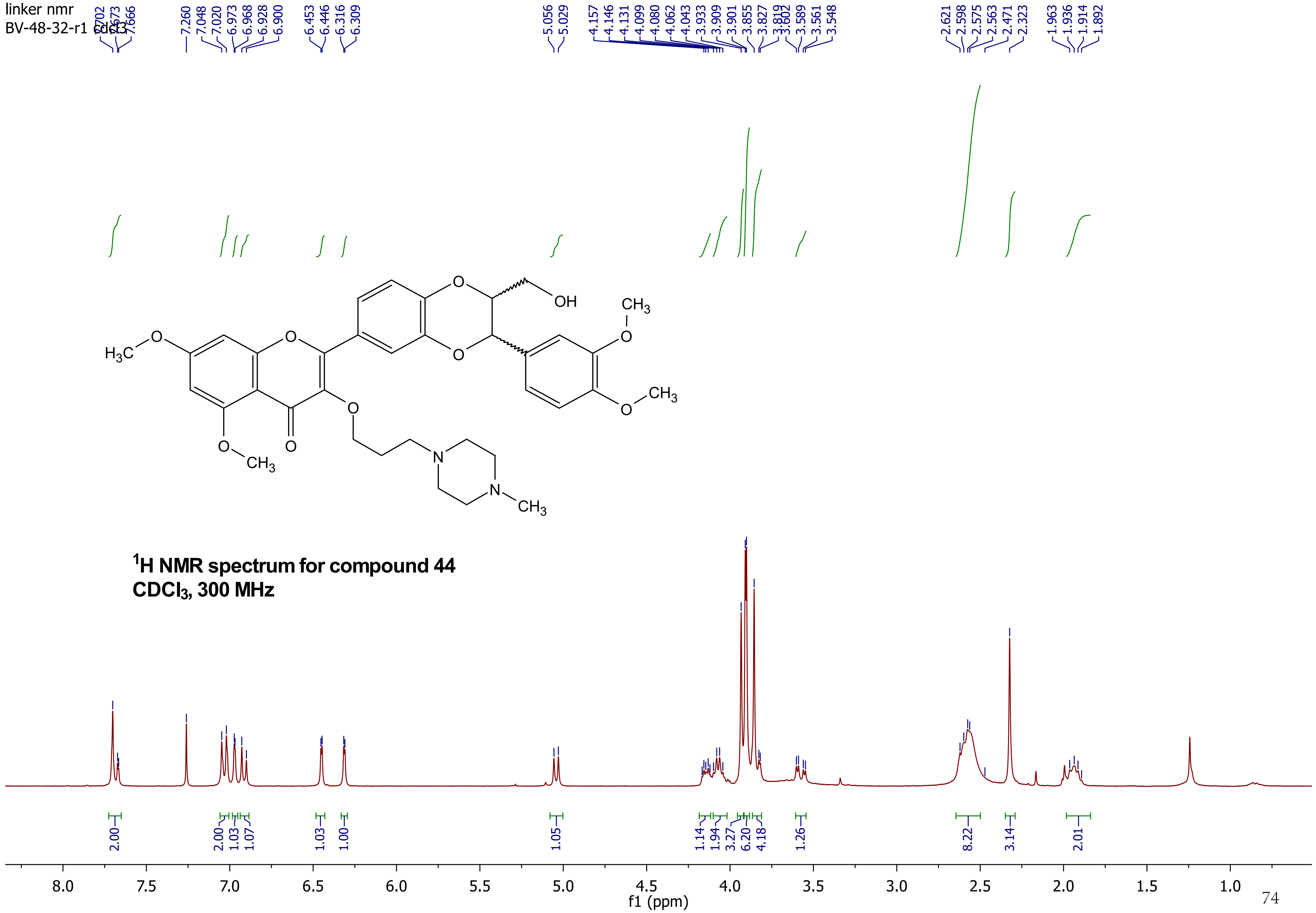

^{1}H NMR spectrum for compound 44
$CDCl_3$, 300 MHz

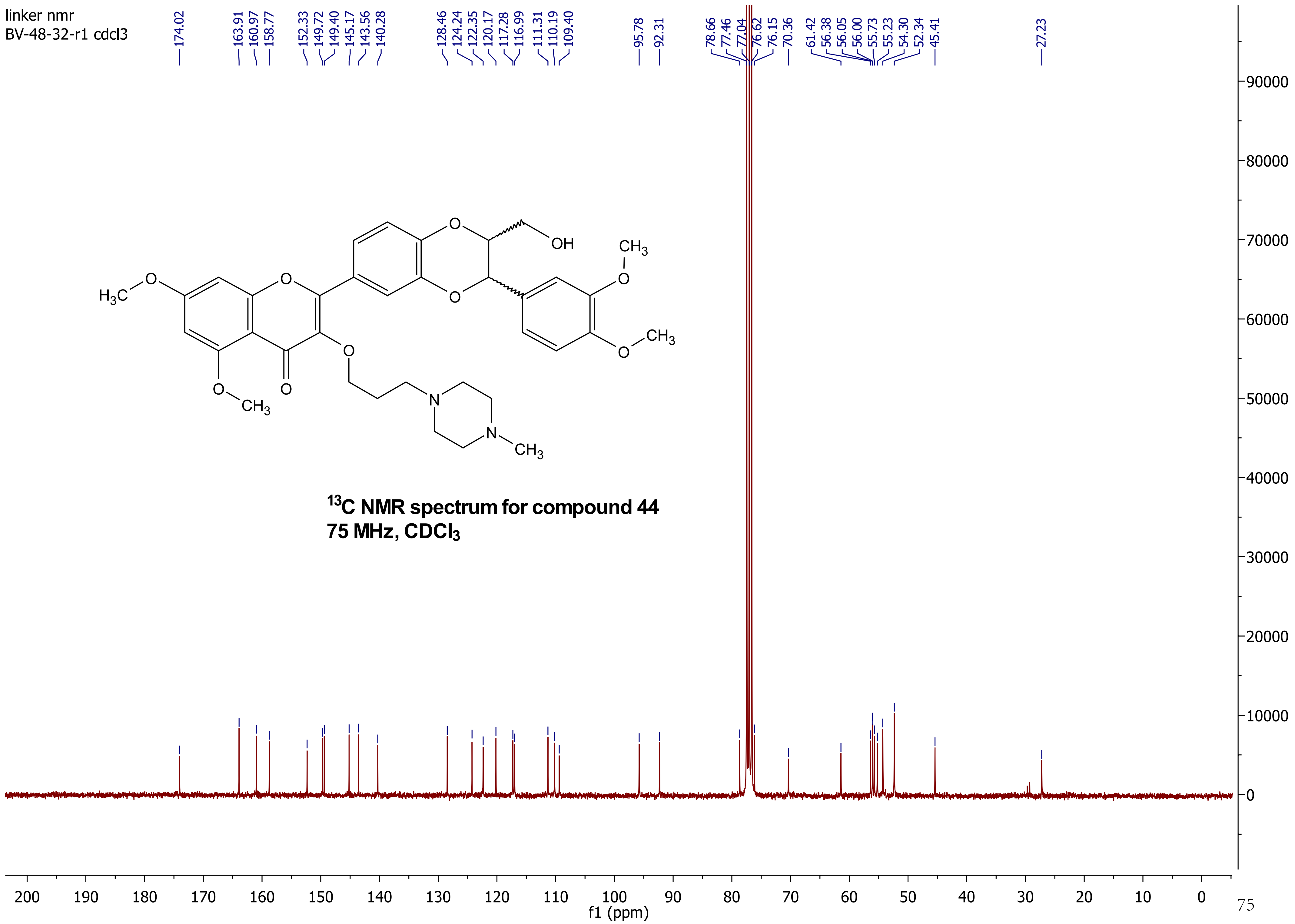

^{13}C NMR spectrum for compound 44
75 MHz, $CDCl_3$

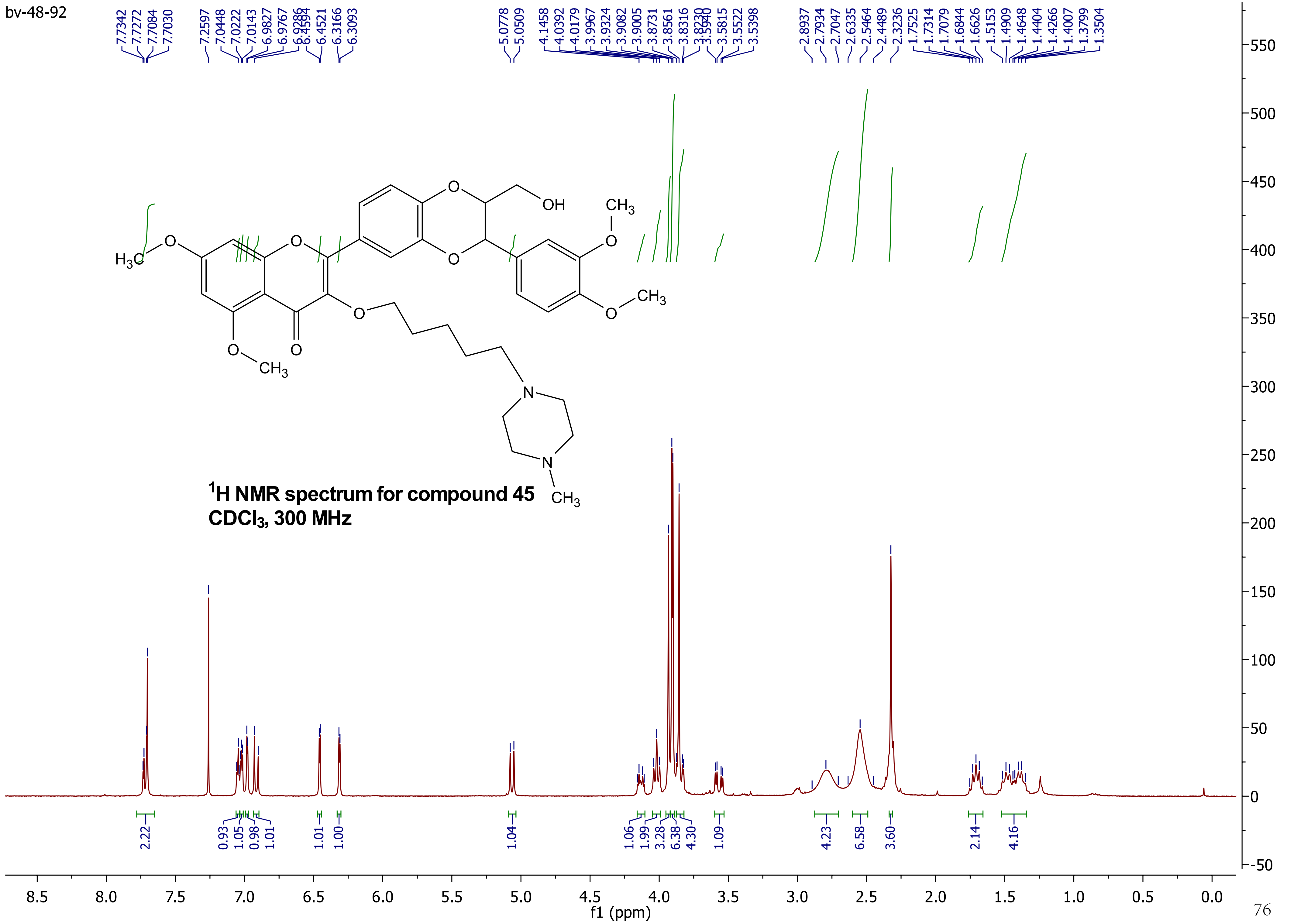

bv-48-92
¹H NMR spectrum for compound 45
CDCl3, 300 MHz
f1 (ppm)

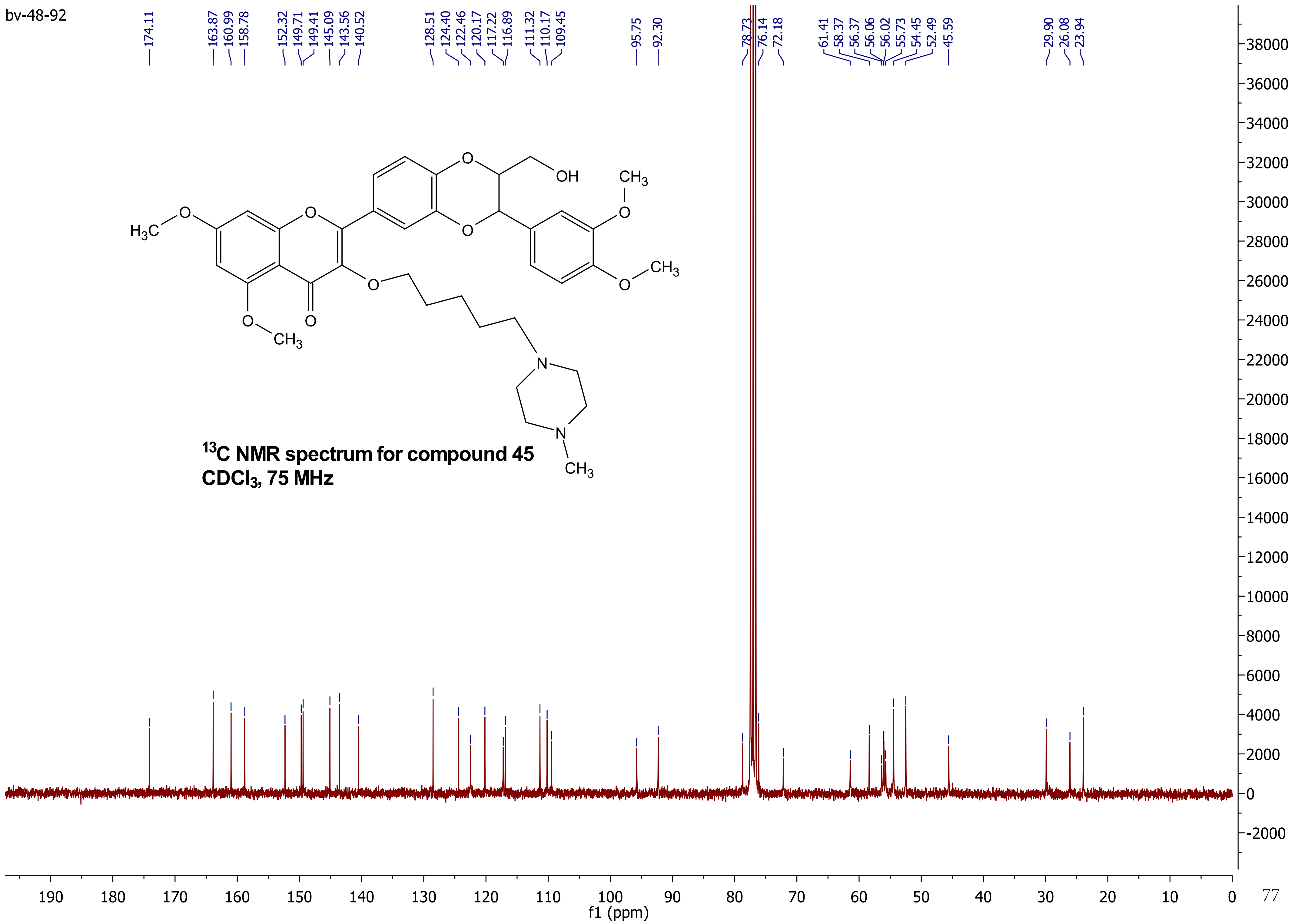

bv-48-92
174.11
163.87
160.99
158.78
152.32
149.71
149.41
145.09
143.56
140.52
128.51
124.40
122.46
120.17
117.22
116.89
111.32
110.17
109.45
95.75
92.30
78.73
76.14
72.18
61.41
58.37
56.37
56.06
56.02
55.73
54.45
52.49
45.59
29.90
26.08
23.94
13C NMR spectrum for compound 45
CDCl3, 75 MHz
f1 (ppm)
